# Elucidating the fundamental fibrotic processes driving abdominal adhesion formation

**DOI:** 10.1038/s41467-020-17883-1

**Published:** 2020-08-13

**Authors:** Deshka S. Foster, Clement D. Marshall, Gunsagar S. Gulati, Malini S. Chinta, Alan Nguyen, Ankit Salhotra, R. Ellen Jones, Austin Burcham, Tristan Lerbs, Lu Cui, Megan E. King, Ashley L. Titan, R. Chase Ransom, Anoop Manjunath, Michael S. Hu, Charles P. Blackshear, Shamik Mascharak, Alessandra L. Moore, Jeffrey A. Norton, Cindy J. Kin, Andrew A. Shelton, Michael Januszyk, Geoffrey C. Gurtner, Gerlinde Wernig, Michael T. Longaker

**Affiliations:** 1 Hagey Laboratory for Pediatric Regenerative Medicine, Division of Plastic and Reconstructive Surgery, Stanford University School of Medicine, Stanford, CA 94305 USA; 2 Department of Surgery, Stanford University School of Medicine, Stanford, CA 94305 USA; 3 Institute for Stem Cell Biology and Regenerative Medicine, Stanford University School of Medicine, Stanford, CA 94305 USA

**Keywords:** Mechanisms of disease, Gastrointestinal diseases

## Abstract

Adhesions are fibrotic scars that form between abdominal organs following surgery or infection, and may cause bowel obstruction, chronic pain, or infertility. Our understanding of adhesion biology is limited, which explains the paucity of anti-adhesion treatments. Here we present a systematic analysis of mouse and human adhesion tissues. First, we show that adhesions derive primarily from the visceral peritoneum, consistent with our clinical experience that adhesions form primarily following laparotomy rather than laparoscopy. Second, adhesions are formed by poly-clonal proliferating tissue-resident fibroblasts. Third, using single cell RNA-sequencing, we identify heterogeneity among adhesion fibroblasts, which is more pronounced at early timepoints. Fourth, *JUN* promotes adhesion formation and results in upregulation of *PDGFRA* expression. With *JUN* suppression, adhesion formation is diminished. Our findings support *JUN* as a therapeutic target to prevent adhesions. An anti-*JUN* therapy that could be applied intra-operatively to prevent adhesion formation could dramatically improve the lives of surgical patients.

## Abstract (web summary)

Abdominal adhesions are a common cause of bowel obstruction, but knowledge regarding adhesion biology and anti-adhesion therapies remains limited. Here the authors report a systematic analysis of mouse and human adhesion tissues demonstrating that visceral fibroblast JUN and associated PDGFRA expression promote adhesions, and JUN suppression can prevent adhesion formation.

## Introduction

The peritoneum is a two-layered tissue consisting of a single-cell covering and underlying connective tissue, which lines the abdominal cavity and covers intra-abdominal organs including the gastrointestinal tract^1^. When there is injury (e.g., a surgical procedure) or infection (e.g., diverticulitis), adhesions form connecting the parietal peritoneum to the visceral peritoneum of intra-abdominal organs, or connecting intra-abdominal organs to one another^2,3^. Adhesions are the number one cause of small bowel obstruction, and can also cause infertility or chronic pain, and complicate subsequent operations^4,5^. Adhesions form postoperatively in 50–90% of all open abdominal operations and as such, represent an enormous clinical problem impacting hundreds of millions of patients each year. Despite the magnitude of adhesions, there are currently no effective treatments to prevent adhesion formation.

Literature addressing the evaluation and management of adhesions is overall limited. Recent research exploring fibrosis in a variety of organ systems showed that *JUN* signaling is paramount in fibrogenesis. *JUN* signals via several known fibrosis-related pathways, including *VEGF*, *FGFR*, *PDGFR*, and *TGFBR*^6^. Inhibitors of these well-known, downstream, fibrosis-associated pathways have shown some effect at modulating adhesion formation in mice, for example the MEK inhibitor trametinib^7^, but it has remained unknown what the upstream transcriptional regulator of adhesion pathology might be. Furthermore, abdominal adhesion fibroblasts have yet to be characterized at the single-cell level to appreciate the functional heterogeneity involved.

In this study, we investigate the origin of adhesion-forming cells, and show using in vivo models that adhesions derive primarily from the visceral peritoneum. This is in line with our clinical observation that adhesions are most severe following open abdominal surgical procedures that involve manipulation of the bowel (Supplementary Fig. 1a–c). Using bulk and single-cell RNA-seq, we explore patterns of gene expression and heterogeneity among abdominal adhesion fibroblasts derived from mouse and human tissue specimens. These data suggest that *JUN* is a transcriptional master regulator of fibroblasts in the context of abdominal adhesions. Further, we show that *JUN* signals via *JAK*-*STAT* and epithelial-mesenchymal transition (EMT) pathways, and results in upregulation of PDGFRA expression among adhesion fibroblasts. With in vivo *JUN* suppression, adhesion formation is dramatically decreased. Application of *JUN* knockdown to primary human adhesion fibroblasts, significantly reduces profibrotic signaling, proliferation, and collagen production. Our findings suggest that an anti-*JUN* therapy might be effective to prevent adhesions clinically.

## Results

### *JUN* promotes adhesions and upregulates PDGFRA expression

**Fig. 1:**
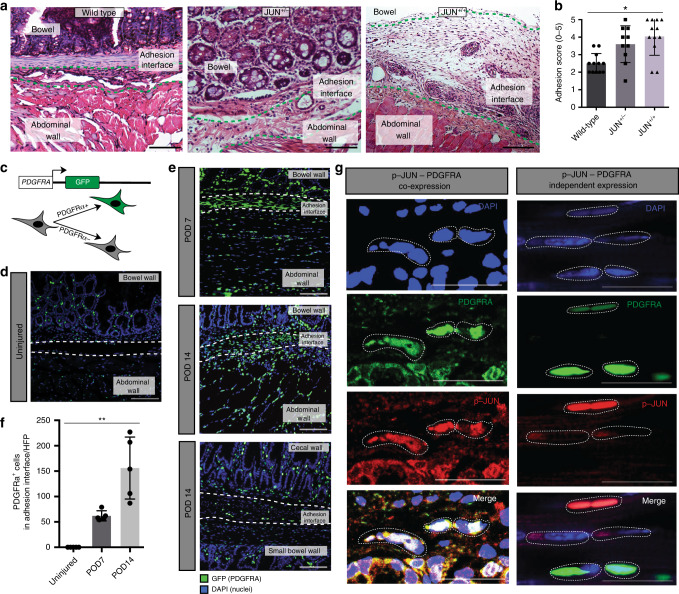
*JUN* promotes adhesions and upregulates PDGFRA expression. **a** Representative samples of hematoxylin and eosin (H&E) stained abdominal adhesion tissue specimen from *JUN*^+/+^ (right panel), *JUN*^+/−^ (middle panel) and wild-type mice (left panel). Green dotted lines outline adhesion interface, structures as labeled in figure. *n* > 10 biological replicates. Scale bars, 100 μm. **b** Application of an objective histologic adhesion rating score by blinded pathologists (based on to the gross score used by Tsai et al. (2018)^19^ and the histologic score used by Linsky et al., (1987)^36^) quantifies relative adhesion severity in wild-type, *JUN*^+/−^, and *JUN*^+/+^ mouse specimens. *n* = 10 biological replicates. **c** Schematic of the PDGFRA^GFP^ mouse construct. **d** Fluorescent imaging of PDGFRA^GFP^ mouse uninjured (control) visceral and parietal peritoneum. Structures as labelled in figure, white dotted lines outline area of potential adhesion interface, POD postoperative day. *n* = 10 biological replicates. Scale bar, 100 um. **e** Fluorescent imaging of PDGFRA^GFP^ mouse adhesion tissue at POD 7 (top panel), POD 14 (middle panel—visceral-parietal adhesion, bottom panel—visceral-visceral adhesion). Structures as labelled in figure, white dotted lines outline adhesion interface. GFP green fluorescent protein. *n* > 5 biological replicates. Scale bars, 100 μm. **f** Quantitation of GFP+(PDGFRA^GFP^) cells per high power field (HPF) in the adhesion interface in mouse adhesions (from Fig. 1d, e). *n* = 5 biological replicates. **g** Immunofluorescent staining of representative samples shows PDGFRA and phospho (p)-JUN colocalization (left panels) and independent expression (right panels) within the adhesion interface. Individual panels at top, merge in bottom row, white dotted lines highlight cells of interest. Scale bars, 25 μm. Data and error bars represent means ± standard deviation (SD). *P = 0.0009 (one-way Anova), **P = 0.0001 (one-way Anova). Source data are provided as a Source Data file.

*JUN* is a member of the Activator Protein-1 (AP-1) transcription factor complex, which has conserved function in mice and humans, and was recently found to promote fibrotic disease in the lung, skin, bone marrow, kidney, liver, pancreas, and heart^6^. To explore if *JUN* might also promote abdominal adhesion formation, we examined JUN expression in an established model for mouse adhesions^8^. This surgical model relies on abrasive injury to both the visceral and parietal peritoneum and results in the formation of dense adhesions, which are maintained over the life span of the mice (Supplementary Fig. 2a, b). We found that JUN expression is upregulated in adhesion tissue (Supplementary Fig. 3a—left panels) compared with control peritoneum in wild-type mice (Supplementary Fig. 3a—right panels). Using a flp-in tetO c-jun (*JUN*) mouse, *JUN* expression results in significantly increased adhesion formation (Fig. 1a, b) compared with wild-type mice (Fig. 1a, b, Supplementary Fig. 2a, b).

*JUN* produces downstream signaling through several known fibrosis-related pathways^6^. To explore *JUN* signaling in the context of adhesions, we isolated mouse adhesion fibroblasts via fluorescence activated cell sorting (FACS) using an unbiased approach involving lineage-labeling of non-fibroblast cells^9^. We screened the isolated fibroblasts for expression of fibrosis-relevant markers, and found that PDGFRA, along with activated-fibroblast markers including a smooth muscle actin (ASMA), vimentin (VIM), and collagen 1 (COL1), are strongly expressed by mouse adhesion fibroblasts (Supplementary Fig. 3b—quantitation at right). PDGFRA is a transmembrane receptor tyrosine kinase and fibroblast marker in the dermis, and is a known promotor of systemic fibrosis^10–12^.

To validate PDGFRA expression in adhesion-forming fibroblasts, we created adhesions in PDGFRA^GFP^ mice (Fig. 1c)^13^. JUN is also expressed in abdominal adhesions in these tissues (Supplementary Fig. 3c). Fluorescent imaging of uninjured bowel and abdominal wall shows PDGFRA-expressing cells scattered throughout both structures in a pattern typical for tissue-resident fibroblasts (Fig. 1d). Seven days after surgery, PDGFRA-expressing cells are numerous along the adhesion interface (Fig. 1e—top panel). At postoperative day (POD) 14, PDGFRA-expressing cells increase in the adhesion interface (Fig. 1e—middle and bottom panels, Fig. 1f), suggesting that this cell population is a primary contributor to adhesions.

Mouse adhesion fibroblasts also express fibroblast specific protein-1 (FSP1) (Supplementary Fig. 3b), which labels fibroblasts in lung and liver fibrosis^14,15^. FSP1 expression upregulates *JAK2/STAT5* signaling in adventitial fibroblasts^16^. We found that FSP1 expression correlated with JUN expression (mean 76% of JUN+-fibroblasts, SD 2.9) (Supplementary Fig. 4a, Supplementary Fig. 3d—top row). PDGFRA expression captures the majority of the JUN+-adhesion fibroblasts (mean 90.6% of phospho-JUN+/FSP1+ cells, SD 2.1) (Fig. 1g—left panels, Supplementary Fig. 4a, Supplementary Fig. 3d—top row), although there are also minor populations of fibroblasts that express PDGFRA and JUN independently (Fig. 1g—right panels), indicating heterogeneity among the fibroblasts responsible for adhesions. ASMA expression, known to identify activated fibroblasts, is similar to PDGFRA expression (Supplementary Fig. 3d—second row). We explored expression of other fibrosis-associated fibroblast markers including podoplanin (PDPN) and CD10, which were found to be relatively less expressed in mouse adhesions (Supplementary Fig. 3d—bottom rows). As such, adhesion fibroblasts can be characterized by expression of JUN, PDGFRA, ASMA, and to a lesser extent, FSP1, in mice.

IL6, STAT3, and STAT5 are also expressed in adhesion tissue. IL6 is a known mediator of inflammation and fibrosis in the liver^17^. STAT5 expression is central to myelo- and lympho-proliferative disease^18^ and is upregulated in other fibrotic pathologies including bleomycin-induced pulmonary fibrosis. Following peritoneal injury with vessel damage in the context of abdominal surgery or intra-abdominal infection, platelets are recruited to the site and immediately release acute phase factors such as IL6 and PDGF. We hypothesize that this results in the preliminary activation of *JUN*, which can then autoamplify, signal via *STAT3* and *STAT5*, and stimulate fibroblast production of IL6 and related factors, ultimately driving a chronic fibrotic state in adhesion cells.

We found that in our model of abdominal adhesions, mesothelin (MSLN) was expressed by a portion of the JUN+ fibroblast population (Supplementary Fig. 5a). This is consistent with a “hypoxic button” model of parietal peritoneal fibrosis, which found MSLN to be strongly expressed in the parietal peritoneum^19^. As such, we wanted to determine if JUN expression might also be upregulated in that model. Using the hypoxic button model, we found that JUN expression is strongly induced and correlates with prominent MSLN expression, suggesting that JUN may also be responsible for fibrosis in that context (Supplementary Fig. 5b).

Previous research exploring ischemic fibrosis in the parietal peritoneum suggested elevated expression of Wilms tumor antigen 1 (WT1)^19^. We found that this protein does not contribute significantly to adhesion tissue using our model in endogenous WT1-expressing mice (WT1^Cre^::ROSA26^mTmG^) (Supplementary Fig. 5c—top panels). Similarly, Engrailed-1 (EN1)-lineage fibroblasts, which are the predominant scar forming fibroblast in the dorsal dermis of skin^20^, are only rarely present in the parietal peritoneum using endogenous EN1-expressing mice (EN1^Cre^::ROSA26^mTmG^), are not found in the visceral peritoneum, and do not contribute to adhesions (Supplementary Fig. 5c—bottom panels).

### Local fibroblasts proliferate polyclonally to form adhesions

**Fig. 2:**
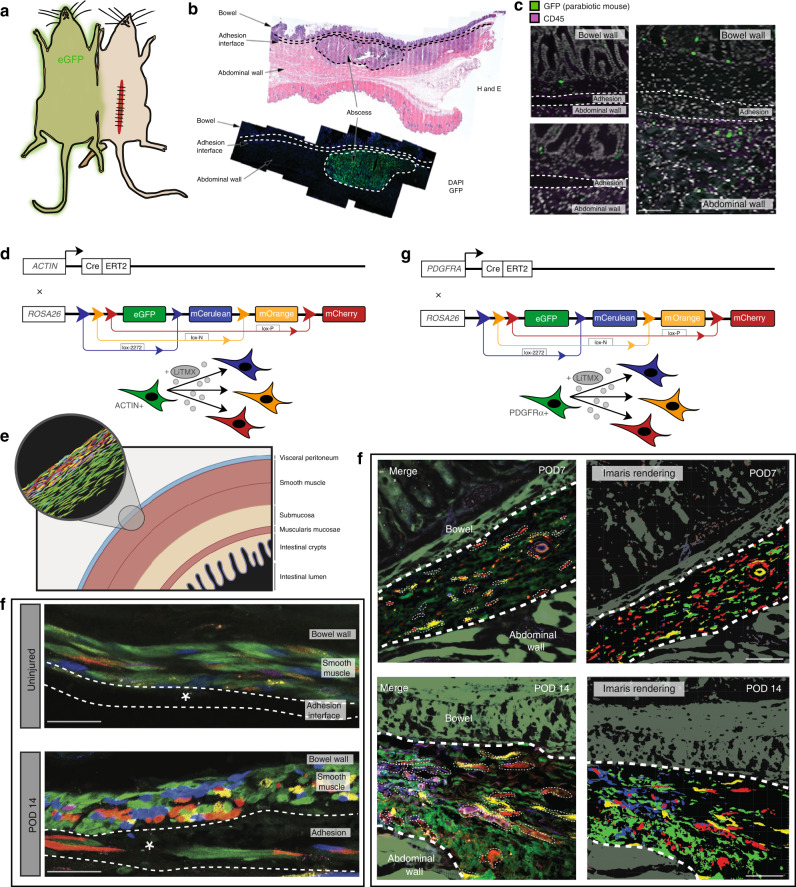
Local fibroblasts proliferate polyclonally to form adhesions. **a** Parabiosis schematic. **b** H&E and IF data showing the presence of an abscess identified along the abdominal wall adjacent to the adhesion interface in a mouse parabiont, which is strongly GFP+ (green fluorescent protein) secondary to the presence of circulating immune cells in the abscess. **c** No GFP+ cells were identified in the adhesion interface. Green (GFP) represents circulating cells, purple (CD45) stains for immune cells. *n* = 3 biological replicates. Scale bars, 50 μm. **d** Schematic of the Actin^CreER^::ROSA26^VT2/GK3^ Rainbow mouse construct. **e** Schematic showing Actin^CreER^::ROSA26^VT2/GK3^ Rainbow mice locally induced with activated tamoxifen liposomes (LiTMX) at time of adhesion formation, using a published protocol^21^, ERT2 estrogen receptor T2. **f** Actin^CreER^::ROSA26^VT2/GK3^ Rainbow mouse uninjured control (top panel) and adhesion tissue harvested at POD 14 (bottom panel). Clonal proliferation of fibroblasts are visualized along the adhesion interface. Representative samples, structures as labelled in figures, white dotted lines outline adhesion, white asterisk marks adhesion interface, confocal imaging. *n* = 5 biological replicates. Scale bars, 25 μm. **g** Schematic of the PDGFRA^CreER^::ROSA26^VT2/GK3^ Rainbow mouse construct. **h** Confocal imaging of representative PDGFRA^CreER^::ROSA26^VT2/GK3^ Rainbow mouse adhesion samples showing cellular clonality in the adhesion interface at postoperative day (POD) 7 (top panels, Imaris rendering at right), and POD 14 (bottom panels, Imaris rendering at right). Structures as labelled in figures, thick white dotted lines outline adhesion interface, thin white dotted lines outline individual clones, confocal imaging. *n* = 5 biological replicates. Scale bars, 50 μm.

We hypothesized that there are two possible sources for adhesion-forming cells: local tissue-resident cells activated in the context of abdominal surgery, or systemic circulating cells recruited to the site following injury. To investigate this, we created adhesions in wild-type mice parabiosed to eGFP (ACTIN^GFP^) mice (Fig. 2a—left panel). At POD 14, we found histologically that there was no significant contribution from circulating mesenchymal cells (GFP+) to the adhesion interface (Fig. 2c), while a dense contribution of GFP+ cells was seen in an abscess in the abdominal wall which formed nearby the adhesion interface in one of the samples, further validating our model (Fig. 2b). These data indicate that the major source of adhesion-forming cells is local tissue.

Our previous work has demonstrated polyclonal proliferation of tissue-resident cells involved in wound healing, suggesting the presence of local progenitor-type cells that are activated in response to injury^21^. To determine whether adhesions are formed by polyclonal proliferation of local cells, we created abdominal adhesions in Rainbow mice (ACTIN^CreER^::ROSA26^VT2/GK3^) (Fig. 2d). The Rainbow mouse expresses an inducible fluorescent reporter. After induction, cells expressing the Cre driver of interest express one of four colors (eGFP, mCerulean, mCherry, and mOrange) and all subsequent progeny cells express the same color as the initial parent cell^22^. We induced the peritoneum of ACTIN^CreER^::ROSA26^VT2/GK3^ mice during adhesion formation (Fig. 2e) using locally applied 4-hydroxytamoxifen liposomes, which permits precise labelling of tissue-resident cells^21^. Compared with uninjured peritoneum (Fig. 2f—top panel), polyclonal expansion of Rainbow cells was observed extending along the adhesion interface (Fig. 2f—bottom panel) at POD 14. We confirmed the identity of the clonal cells as adhesion-forming fibroblasts by inducing adhesions in Rainbow mice using an inducible PDGFRA^CreER^ driver (PDGFRA^CreER^::ROSA26^VT2/GK3^) (Fig. 2g). Polyclonal proliferation of PDGFRA+ Rainbow fibroblasts is seen along the adhesion interface at POD 7 (Fig. 2h—top panels), and these clones expand at POD 14 (Fig. 2h—bottom panels). These data show that adhesions arise from tissue-resident, progenitor-type fibroblasts that proliferate polyclonally in response to injury.

### Adhesion fibroblasts derive primarily from the viscera

**Fig. 3:**
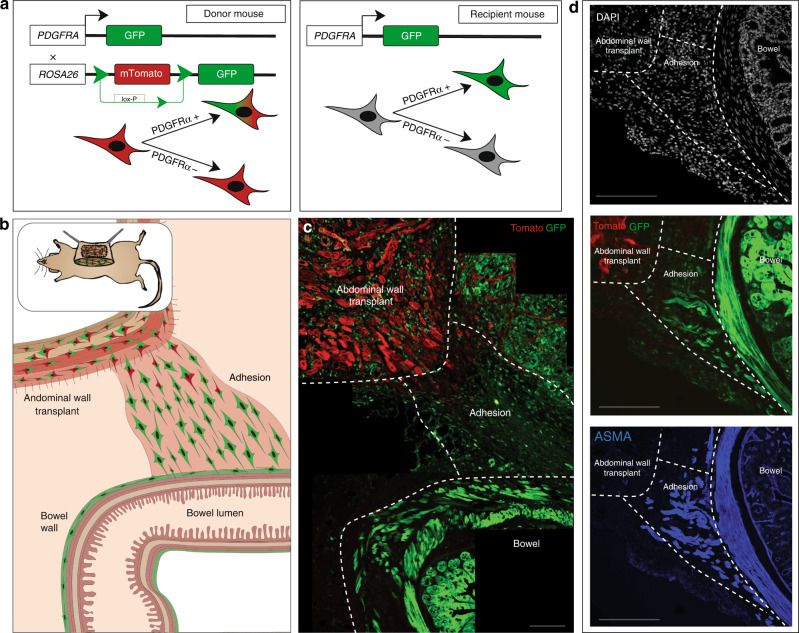
Adhesion fibroblasts derive primarily from the viscera. **a** Schematics of the PDGFRA^GFP^::ROSA26^mTmG^ mouse “donor” model (left panel) and PDGFRA^GFP^ “recipient” model (right panel). **b** Schematic for abdominal wall transplant model using abdominal wall from PDGFRA^GFP^::ROSA26^mTmG^ mice, transplanted into PDGFRA^GFP^ mice, followed by adhesions surgery. **c** Confocal imaging of representative abdominal wall transplant model mouse adhesion tissue shows a prominence of GFP+ cells within the adhesion interface, relative to GFP-mTomato+ cells, harvested at POD 14. White dotted lines mark structures as labelled in images, confocal imaging. *n* = 5 biological replicates. Scale bar, 100 μm. **d** Confocal imaging of the abdominal wall transplant model representative tissue shows PDGFRA/ASMA co-expression (PDGFRA labelled with GFP using the mTmG mouse model, IF staining for ASMA) among the cells migrating from the visceral peritoneum into the adhesion interface. White dotted lines mark structures as labelled in images, confocal imaging. *n* = 5 biological replicates. Scale bars, 100 μm.

Clinically, adhesion formation is most pronounced after open laparotomy during which the bowel is manipulated, rather than following laparoscopy (which often only injures the parietal peritoneum) (Supplementary Data Fig. 1a–c). This supports the idea that the visceral (bowel wall) peritoneum might be the primary contributor to adhesion formation. To determine whether cells from the visceral or parietal (abdominal wall) peritoneum are more active in adhesion formation, we designed an abdominal wall transplant procedure. The abdominal wall (full thickness muscular layer and parietal peritoneum) of PDGFRA^GFP^::ROSA26^mTmG^ mice, in which all cells express membrane (m) Tomato, and PDGFRA+ cells express both m-Tomato and GFP (Fig. 3a—left panel), was excised and rapidly transplanted to the abdominal wall of PDGFRA^GFP^ mice, in which PDGFRA+ cells express GFP (Fig. 3a—right panel, Supplementary Fig. 6a—left panel). Adhesions were then created between the native bowel and the transplanted wall (Fig. 3b, Supplementary Fig. 6a—right panel). At POD 14, the vast majority of fibroblasts in the adhesion interface were GFP+, derived from the visceral rather than parietal peritoneum (Fig. 3c). ASMA expression was found to correlate closely with PDGFRA expression in this context (Fig. 3d). In summary, abdominal adhesions are derived primarily from the visceral peritoneum, supporting the well-known clinical observation.

### Adhesion fibroblasts upregulate EMT and show heterogeneity

**Fig. 4:**
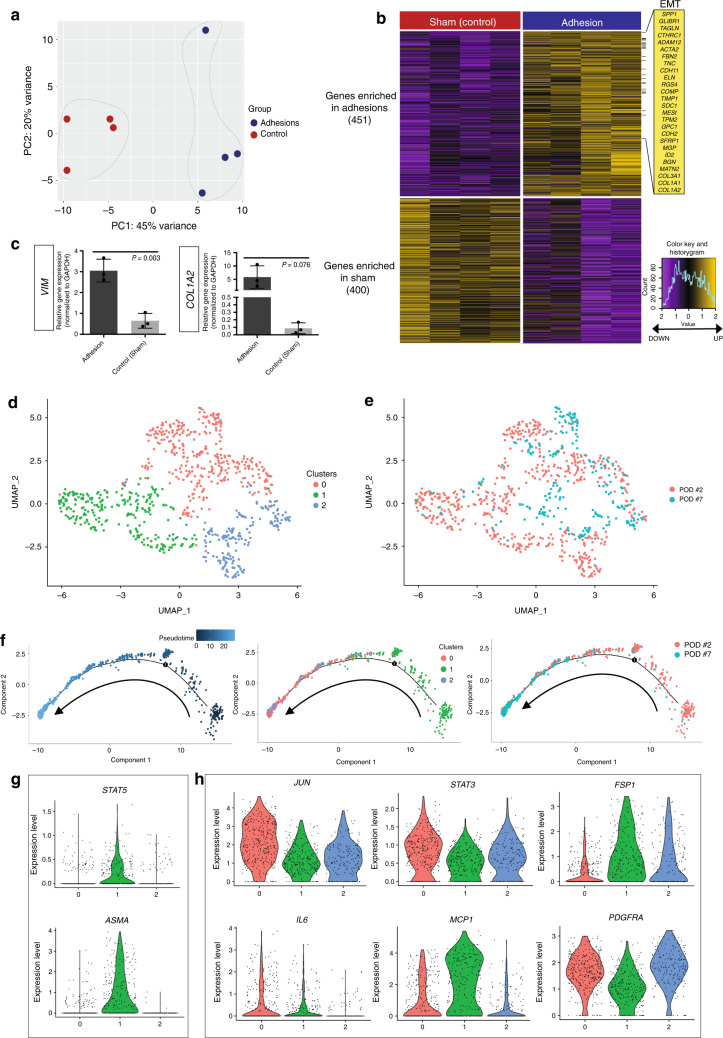
Adhesion fibroblasts upregulate EMT and show heterogeneity. **a** Principal component analysis (PCA) plot comparing bulk RNA-seq gene expression for adhesion (*n* = 4) and control peritoneum (*n* = 4) FACS-isolated mouse fibroblasts. Colors as labelled, variances noted on plot. **b** Heatmap of mouse adhesion-forming fibroblast bulk RNA-seq data shows significant differential gene expression between adhesion and control peritoneum (sham surgery) cohorts. Upregulated EMT-pathway genes noted at right. Gene enrichment as noted in figure, color key, and histogram at far right. **c** Quantitation of qPCR for vimentin (*VIM*) and collagen 1a2 (*COL1A2*) shows upregulation of gene expression in the context of mouse abdominal adhesions. Data and error bars represent means ± SD; *P*-values noted in figure, unpaired two-tailed *t*-test. *n* = 3 replicates per condition, datapoints represent average of technical replicates. **d** Uniform manifold approximation and projection (UMAP) plot showing single-cell (sc) RNA-seq data from mouse adhesion fibroblasts FACS-isolated using an unbiased, lineage-negative sort strategy at POD 2 (*n* = 4) and POD 7 (*n* = 4) following adhesion induction. Three unique clusters of fibroblasts are identified. Colors as labelled in the figure panel. **e** UMAP plot showing distribution of mouse scRNA-seq fibroblasts in terms of harvest timepoint relative to the clusters in panel **d**. Cells isolated at both timepoints are represented in all clusters. Colors as labelled in the figure panel. **f** Pseudotime analysis (Monocle 2) of mouse scRNA-seq data: Pseudotime analysis (left panel), representation of scRNA-seq clusters across the pseudotime analysis shows a clear progression from cluster 1 to clusters 0 and 2 (middle panel) and relative to timepoints, the cells follow a logical time progression that mirrors the pseudotime with the largest representation of POD 2 cells in cluster 1 and more POD 7 cells in clusters 0 and 2 (right panel). Arrows indicated direction of pseudotime progression. **g** Violin plots showing expression of *STAT5* and *ASMA* within the scRNA-seq data. Colors and numbering on *x*-axis match cluster colors assigned in panel **d. h** Additional violin plots showing expression of *JUN*, *STAT3*, *FSP1*, *IL6*, *MCP1*, and *PDGFRA* relative to the scRNA-seq data clusters seen in panel **d**. Colors and numbering on *x*-axis match cluster colors assigned in panel **d**. Source data are provided as a Source Data file.

Next, we examined FACS-isolated mouse adhesion fibroblast gene expression using bulk RNA-seq (Supplementary Fig. 7a, b, Supplementary Dataset 1). Principal component analysis (PCA) showed clear separation between adhesion and control (sham surgery) transcriptomes (Fig. 4a). We then compared gene expression profiles of adhesion and control specimens. Using DESeq2, we identified 451 genes that were significantly enriched in mouse adhesion fibroblasts and 400 that were significantly enriched in sham surgery control cells (false discover rate [FDR] <0.01; Fig. 4b). Genes upregulated in mouse adhesion fibroblasts are known to be involved in fibroblast activation and fibrosis, including *ACTA2* (*ASMA*), tenascin C (*TNC*), *COL1A1*, *COL1A2*, and *COL3A1*.

We next performed gene set enrichment analysis (GSEA) using the Molecular Signatures Database (MSigDb) to identify expression programs associated with mouse adhesion fibroblasts. One of the most significantly enriched molecular signatures in mouse adhesion fibroblasts was the epithelial-mesenchymal transition (EMT) pathway (Supplementary Fig. 8a). EMT is associated with cell proliferation in the setting of neoplasia as well as tissue fibrosis in a variety of organ systems including hepatic and pulmonary fibrosis^23,24^. Specific genes of interest in this pathway relating to organ fibrosis include osteopontin (*SPP1*), periostin (*POSTN*), *TIMP1*, cartilage oligomeric matrix protein/thrombospondin-5 (*COMP*), *TNC*, and N-cadherin (*CDH2*, *CD325*) (Fig. 4b). *SPP1* is an established *JUN* target gene and has specifically been associated with *JUN*-mediated hepatic fibrosis^23^. *COMP*, a non-collagen ECM protein, and *TNC* are both upregulated in the context of pulmonary fibrosis, which *JUN* signaling also mediates^24,25^. *JUN* is an important transcription factor in cardiac fibrosis^26^, in which *COMP* is upregulated. *COMP* is also known to be highly induced in skin fibrosis and specifically involved in collagen secretion^27^. As such, these upregulated EMT-pathway genes represent likely targets by which *JUN*+/*PDGFRA*+ fibroblasts induce adhesions.

Other fibrosis-associated gene sets enriched in mouse adhesion fibroblasts include ‘Regulation of Response to Wound Healing’, ‘Positive Regulation of Cell Proliferation’, and ‘Positive Regulation of Cell Differentiation’ (Supplementary Fig. 8a). Furthermore, hypergeometric test of gene ontology (GO) terms revealed enrichment of cytokine production regulation, extracellular matrix (ECM) organization, collagen fibril organization, endopeptidase activity, ECM structural constituents, growth factor binding, and metallopeptidase activity in mouse adhesion fibroblasts (Supplementary Fig. 8b). qPCR assay confirmed upregulation of profibrotic genes in adhesion-forming fibroblasts including *VIM* and *COL1A2* (Fig. 4c).

To explore fibroblast heterogeneity in the context of abdominal adhesions, we FACS-isolated mouse adhesion fibroblasts using the aforementioned, unbiased, lineage-negative based sorting strategy and conducted single-cell (sc) RNA-seq. Adhesion fibroblasts from each of two timepoints (POD 2 and POD 7) were analyzed using the 10x Genomics platform. Our findings demonstrate three unique clusters among pooled fibroblasts (Fig. 4d), with heterogeneous gene expression observed among fibroblasts from each timepoint (Fig. 4e, Supplementary Fig. 9a, b). The heterogeneity appears greater among the fibroblasts from POD 2 compared to POD 7 (Fig. 4e).

Next, we predicted differentiation states using CytoTRACE, a computational tool that orders single cells by relative developmental potential based on transcriptional diversity^28^. Our analysis identified a clear lineage trajectory stemming from cluster 1—represented primarily by POD 2 adhesion fibroblasts (Supplementary Fig. 9c). In addition, we found that the top 200 genes associated with less differentiation in two independent datasets, mouse lung fibroblast and human mesoderm development, also align with these results (Supplementary Fig. 9d). Next, we applied pseudotime analysis to further characterize the comparative properties of the identified fibroblast subpopulations^29^. In line with the CytoTRACE results, we observe a clear pseudotime trajectory from cluster 1 with significant differences observed between the timepoints evaluated—POD 2 versus POD 7—in the mouse abdominal adhesion formation process (Fig. 4f). We explored the specific gene expression changes involved with this trajectory and found that JUN is activated early at POD 2 (cluster 1) and expression is maintained throughout, suggesting that there may be a persistent, JUN+, profibrotic state obtained for adhesion fibroblasts once activated (Fig. 4h, Supplementary Fig. 9e). *STAT5* and *ASMA* are expressed primarily in cluster 1 in parallel with *JUN* activation (Fig. 4g). *STAT3*, *FSP1*, and *MCP1*, are strongly expressed by fibroblasts in all clusters, particularly cluster 1, supporting these as prominent factors in the signaling pathways by which *JUN* signaling promotes fibrosis (Fig. 4h, Supplementary Fig. 9e, f). Interestingly, *PDGFRA* expression appears activated alongside *JUN* by cells in cluster 1 and shows progressively increasing expression through clusters 0 and 2, supporting our protein findings implicating a role for PDGFRA+ fibroblasts in adhesion progression (Fig. 4h, Supplementary Fig. 9e, f).

Gene Ontology (GO) enrichment analysis of the mouse scRNA-seq data revealed enrichment of acute phase factors primarily in cluster 1 such as cellular response to oxidative stress, cell migration, as well as angiogenesis and vascular development. All clusters show enrichment for extracellular matrix organization, but this is highest in cluster 0, whereas cluster 2 shows most enrichment for chemokine-mediated signaling and regulation of apoptotic signaling (Supplementary Fig. 10a, b). Taken together these analyses characterize the properties of the putative subpopulations identified on single-cell gene expression analysis and support our conclusion that *JUN* is an early instigator of abdominal adhesion formation that signals through several impactful profibrotic pathways.

### *JUN* is an early promotor of abdominal adhesion fibrosis

**Fig. 5:**
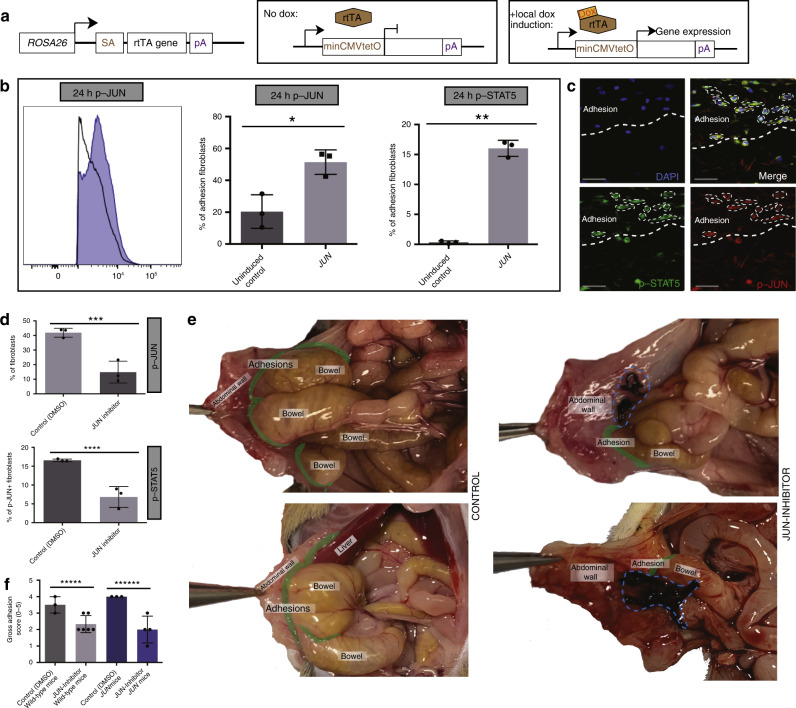
*JUN* is an early promotor of abdominal adhesion fibrosis. **a** Schematic illustrating the targeting construct used in the doxycycline (dox)-inducible *JUN* mouse model. In this construct, *rt*TA is expressed at the endogenous ROSA26 promotor. With dox induction, rtTA undergoes nuclear translocation to activate the Tet-responsive element (minCMV-tet(o)) driving expression of *JUN*. SA splice acceptor, pA poly(A) sequence. **b** Representative phospho-flow-cytometry analysis for phosphorylated (phospho-) JUN (left and middle panels), and phospho-STAT5 (right panel) expression in abdominal adhesion fibroblasts isolated from *JUN* mice 24 h after adhesion surgery (with local induction with doxycycline at the time of adhesion formation) compared with vehicle control. *n* = 3 biological replicates. **c** Immunofluorescent assessment of phospho-JUN and phospho-STAT5 co-expression in mouse abdominal adhesion tissue. Thick white dotted line indicates edge of adhesions interface, co-expressing cells highlighted with thin white dotted lines. *n* = 3 biological replicates. Scale bars, 25 μm. **d** Quantitation of phospho-flow-cytometry analysis showing a significant decrease in phospho-JUN (top panel) and phospho-STAT5 (bottom panel) expression with application of JUN inhibitor versus vehicle control in *JUN* mice at 24 h. *n* = 3 biological replicates per condition, datapoints represent averages of technical replicates. **e** Representative gross images of mouse adhesions at POD 3 treated with (vehicle, DMSO) control (left panels) versus JUN inhibitor (right panels). Adhesion interface highlighted in green, structures as indicated in figures, black sutures (circled with blue dotted line) visible on inhibitor specimens are nidus for adhesion formation (these are not visible on control specimens as they are covered with bowel that is adhesed to the abdominal sidewall). *n* ≥ 3 biological replicates per condition per treatment. **f** Gross assessment (using an adhesion severity grading score established by Tsai et al. 2018^19^) of adhesion severity following in vivo inhibition of JUN using JUN inhibitor (T-5224) versus vehicle control in *JUN* (*JUN* expression induced with doxycycline in all *JUN* mice used) and wild-type mice at POD 3. *n* ≥ 3 biological replicates per condition per treatment. Data and error bars represent mean ± SD. *P = 0.01, **P = 0.0001, ***P = 0.004, ****P = 0.003, *****P = 0.01, ******P = 0.009, unpaired two-tailed *t-*test. Source data are provided as a Source Data file.

These results support that *JUN* activation might promote profibrotic pathway signaling in adhesions. To functionally validate this hypothesis, we conducted adhesion surgery on *JUN* mice (Fig. 5a), induced with doxycycline administered locally at the time of adhesion surgery. As early as 24h postoperatively, adhesion fibroblasts from induced *JUN* mice expressed significantly increased levels of phosphorylated (phospho-) JUN compared with vehicle control (Fig. 5b—left and middle planels, Supplementary Fig. 11a). Phospho-JUN+ adhesion fibroblasts from induced *JUN* mice also expressed significantly increased phospho-STAT5 compared with control (Fig. 5b—right panel, Supplementary Fig. 11a), indicating this as a signaling pathway through which *JUN* promotes fibrosis. Co-expression of phospho-JUN and phospho-STAT5 were confirmed histologically (Fig. 5c). JUN expression remains elevated in adhesion tissues from *JUN* mice at POD 3 and 10 (Supplementary Fig. 11b). These findings were further validated at the tissue level. Immunofluorescent staining of mouse adhesion specimens over time shows that JUN expression is activated very early following injury (surgery), followed closely by increases in STAT3 and STAT5 expression (similar to our transcript level findings), as well as IL6, and then PDGFRA expression is activated with JUN and increases more gradually over time (Supplementary Fig. 12a, b). While JUN signaling appears to have a dramatic effect on the recruitment and activities of fibroblasts in the context of abdominal adhesions, although JUN is expressed by other cells (for example, immune cells), JUN expression does not affect the number of CD45+ cells in the adhesion interface (comparing tissues from PDGFRA^GFP^ (wild-type) and *JUN* (induced and control) mice, Supplementary Fig. 13a).

### Functional modulation of *JUN* regulates adhesion formation

**Fig. 6:**
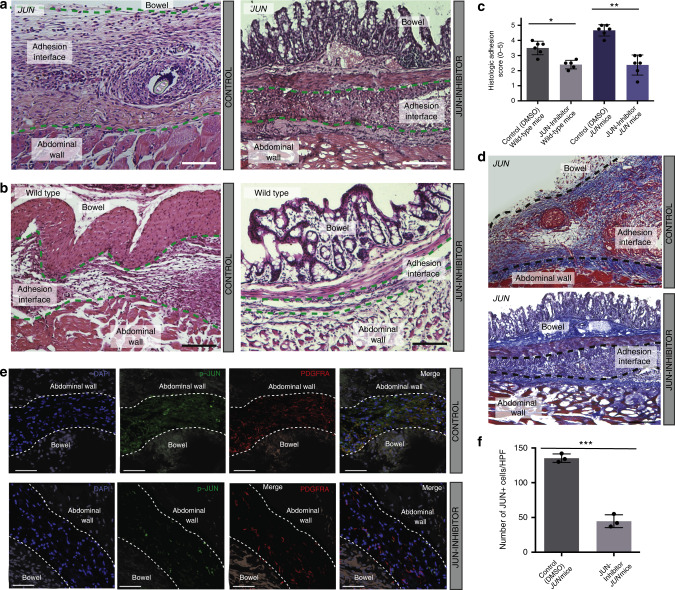
Functional modulation of *JUN* regulates adhesion formation. **a**, **b** Representative H&E sections for vehicle control (left panels) and JUN-inhibitor-treated (right panels), in *JUN* (JUN expression induced with doxycycline in all *JUN* mice used) (**a**) and wild-type (**b**) mice. Adhesion interfaces outlined with green dotted lines. Structures as labelled in figure. Scale bars, 100 μm. *n* = 5 biological replicates per condition per treatment. **c** Histologic scoring (as used in Fig. 1b) of adhesion tissue following in vivo inhibition of JUN using JUN inhibitor versus vehicle control in *JUN* (JUN expression induced with doxycycline in all *JUN* mice used) and wild-type mice. *n* = 5 biological replicates per condition per treatment. **d** Representative images of trichrome staining of *JUN* mouse vehicle control (top panel) and JUN-inhibitor-treated adhesion specimen (bottom panel). *JUN* expression induced with doxycycline in all *JUN* mice used. Adhesion interface outlined with black dotted lines. Structures as labelled in figure. *n* = 5 biological replicates per treatment. Conditions and structures at noted in figure panels. Scale bars, 100 μm. **e** Representative figures showing IF staining for phospho-JUN and PDGFRA in adhesion tissue following in vivo inhibition of JUN (using JUN inhibitor, T-5224) in *JUN* (*JUN* expression induced with doxycycline in all *JUN* mice used) mice. Conditions and structures at noted in figure. Scale bars, 50 μm. **f** Quantification of p-JUN+ cells in **e**. HPF high power field. *n* = 3 biological replicates assessed per treatment condition, datapoints represent average of multiple measures per replicate. Data and error bars represent mean ± SD. *P = 0.001, **P = 0.0001, ***P = 0.0001, unpaired two-tailed *t*-test. Source data are provided as a Source Data file.

Given these findings, we wondered if functional modulation of *JUN* signaling may affect adhesion formation. T-5224 is a selective, small molecule AP-1 inhibitor that has been explored in a variety of fibrotic pathologies and shown to block AP-1-induced early and late cytokine responses^30^. When *JUN* signaling in mouse adhesion fibroblasts was suppressed using T-5224 (JUN Inhibitor), phospho-JUN and phospho-STAT5 expression were significantly reduced in vitro using freshly isolated mouse adhesion fibroblasts (Supplementary Fig. 14a). When this inhibitor was applied intra-abdominally in vivo (in wild-type and *JUN* mice), phospho-JUN and phospho-STAT5 expression were significantly decreased (Fig. 5d, Supplementary Fig. 14b). Grossly, we saw a dramatic decrease in adhesion formation with application of this JUN inhibitor (Fig. 5e, f). Histologically, the adhesions were significantly thinner and less fibrotic with JUN inhibition (Fig. 6a–d). Co-suppression of JUN and PDGFRA were confirmed in this model, and decreased JUN expression with JUN-inhibitor application was significant at the tissue level (Fig. 6e, f).

### Human adhesions recapitulate biology and gene expression

**Fig. 7:**
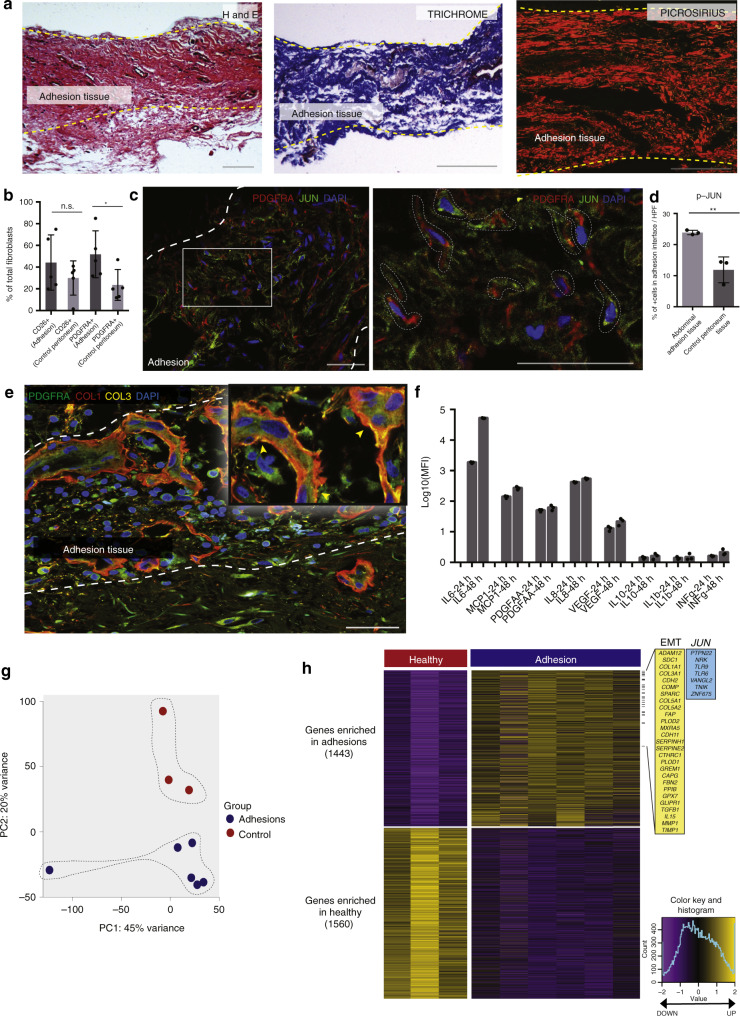
Human adhesions recapitulate biology and gene expression. **a** Representative human abdominal adhesion tissue (*n* = 24 human adhesion specimens) histology (H&E—left, trichrome—middle, picrosirius red—right). Adhesion interface bounded by yellow dotted lines and labeled. Scale bars, 100 μm. **b** On unbiased-FACS analysis, PDGFRA expression is significantly upregulated in human abdominal adhesion fibroblasts, compared with control peritoneum. CD26 expression is also upregulated, although not significantly. Conditions as labelled in figure. *n* = 5 biological replicates per condition. **c** Representative IF staining of human abdominal adhesion tissue for JUN and PDGFRA, right panel is zoom of indicated region in white. Adhesion interface outlined with thick white dotted lines (left panel), colocalization of PDGFRA and JUN expression highlighted with thin white dotted lines (right panel). *n* = 3 biological replicates. Scale bars, 50 μm. **d** Quantitation of p-JUN+ cells in human abdominal adhesion tissue pictured in panel **c**, compared with human control peritoneum tissue. *n* = 3 biological replicates. **e** Representative IF staining of human abdominal adhesion tissue shows colocalization of PDGFRA with collagen 1 (COL1) and collagen 3 (COL3). Adhesion interface outlined with white dotted lines. Structures as labelled in figure. *n* = 5 biological replicates. Scale bar, 50 μm; ×10 zoom at right. **f** Quantitation of cytokine production (including IL6, MCP-1, PDGF-AA, and IL8) by fluorescent assessment of the cell supernatant from primary human abdominal adhesion fibroblasts in vitro, measured 24 and 48 h after isolation. Values normalized to cell-free media for each cytokine assessed. MFI median fluorescence intensity. Conditions as labelled in figure. *n* = 3 replicates analyzed per condition per timepoint. **g** PCA plot of human bulk RNA-seq data shows distinct clustering of human fibroblast specimens FACS-isolated from human abdominal adhesion (*n* = 6) and control peritoneal tissues (*n* = 3) (colors as indicated, variances noted on plot). **h** Heatmap of human adhesion-forming fibroblasts shows significant differential gene expression between conditions. Highly expressed EMT and *JUN* kinase GSEA pathway genes highlighted in yellow and blue panels, respectively, at right. Color key and histogram at far right. Data and error bars represent means ± SD.*P = 0.04, **P = 0.0078, unpaired two-tailed *t-*test. Source data are provided as a Source Data file.

Next, we explored these results in human abdominal adhesions. We collected 24 adhesion tissue specimens from patients with a history of one or more prior abdominal surgical procedure(s) (Supplementary Fig. 15a, Supplementary Table 1) and 10 control peritoneum specimens from patients who had not undergone prior surgery (Supplementary Fig. 15b, Supplementary Table 1). Human adhesions histologically resemble mouse adhesions on H&E (Fig. 7a—left panel). Trichrome staining shows prominent collagen throughout the adhesions (Fig. 7a—middle panel, Supplementary Fig. 15a), and picrosirius red staining shows mature collagen fibers with primarily linear organization (Fig. 7a—right panel). JUN is expressed in human adhesion tissue, similar to what is seen in mouse tissue (Fig. 7c, d).

We FACS-isolated fibroblasts from human adhesion and control tissues using the aforementioned unbiased, lineage-based approach (Supplementary Fig. 16a, b). Human adhesion fibroblasts strongly express VIM and COL1 on immunocytochemistry evaluation compared with control (Supplementary Fig. 16c). Similar to mice, we found that the vast majority (mean 84.8%, SD 7.1) of JUN+ human adhesion fibroblasts expressed FSP1 (Supplementary Fig. 17a, b—top panels). The vast majority of phospho-JUN+/FSP1+ also expressed PDGFRA (91%, SD 1.2) (Supplementary Fig. 17a). Although, similar to the results in mice, there was a small population of PDGFRA+ fibroblasts that did not express JUN, indicating heterogeneity within the human adhesion fibroblast population. A minor portion of JUN+ fibroblasts also expressed MSLN, similar to mice (Supplementary Fig. 17b—middle panels). We confirmed PDGFRA expression in human adhesion fibroblasts (Fig. 7b, c). PDGFRA expression also colocalized with ASMA (Supplementary Fig. 17b—bottom panels). As such, as in mice, human adhesion fibroblasts can be identified by expression of JUN, PDGFRA, ASMA, and FSP1.

At the tissue level, PDGFRA expression colocalizes with COL1 and COL3 expression, showing that PDGFRA+ fibroblasts are directly involved in extracellular matrix production during adhesion formation (Fig. 7e). We also analyzed the supernatant from freshly isolated human abdominal adhesion fibroblasts in terms of cytokine production and found that adhesion fibroblasts primarily secrete IL6, MCP1, PDGF-AA, and also IL8 (Fig. 7f). This assay validates that while IL6 is initially secreted by a variety of cell types in the acute phase response to injury when adhesions first form, adhesion fibroblasts produce their own IL6 as part of a profound, chronic, profibrotic state. IL6 is a direct pathway signaling factor downstream from *JUN*^31^, and PDGF-AA binds to fibroblast PDGFRA receptors stimulating cell proliferation, gene expression, and ECM production. MCP1 is also known to be secreted by fibroblasts in the context of fibrosis^32^.

Next, we explored human adhesion (Supplementary Table 2) versus control (Supplementary Table 2) fibroblast gene expression using bulk RNA-seq (Supplementary Dataset 2). PCA shows separate clustering of adhesion and control (uninjured peritoneum) transcriptomes, with 65% of the variance explained by the first two components (Fig. 7g). We then compared gene expression profiles of adhesion and control specimens. We identified 1443 genes that were significantly enriched in human adhesion fibroblasts and 1560 genes that were significantly enriched in control cells (FDR < 0.01; Fig. 7h). On GSEA, one of the most significantly enriched molecular signatures in human adhesion fibroblasts was the EMT pathway, similar to mouse fibroblasts (Supplementary Fig. 18a). Many of the genes noted in this pathway are associated with tissue fibrosis and were also found to be upregulated in our mouse RNA-seq data including *COMP*, *TIMP1*, *COL1A1*, and *COL3A1*. The *JUN* kinase (JNK) pathway was also significantly enriched in human adhesion fibroblasts (Fig. 7f, Supplementary Fig. 18a). Other significant gene sets associated with human adhesion fibroblasts include ‘Extracellular Matrix Structural Constituent’ and ‘Collagen Trimer’ (Supplementary Fig. 18a). Furthermore, GO terms revealed enrichment of extracellular structure and matrix organization, several chromosomal processes, as well as collagen metabolism and fibril organization (Supplementary Fig. 18b).

### Human adhesion fibroblasts are heterogeneous and *JUN* dependent

**Fig. 8:**
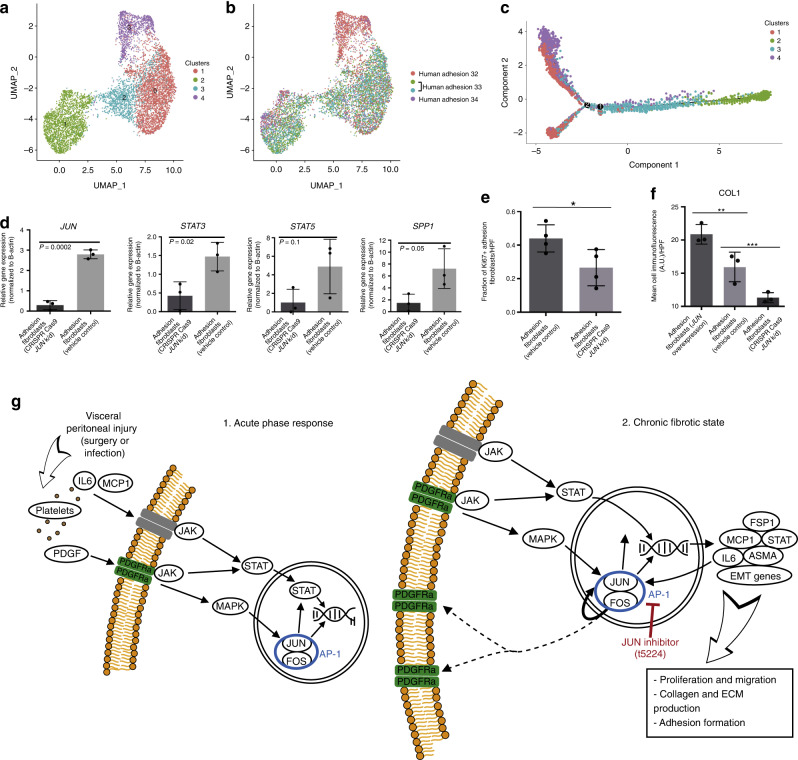
Human adhesion fibroblasts are heterogeneous and *JUN* dependent. **a** Uniform manifold approximation and projection (UMAP) plots showing single-cell (sc)RNA-seq data from human adhesion fibroblasts FACS-isolated using an unbiased, lineage-negative sort strategy from three unique human specimens. Four unique clusters are identified. **b** UMAP plot showing representation of individual patient samples (arbitrarily numbered) across the cluster presented in **1**. Human hashtag labels indicated at right, 2 hashtag antibodies were used for patient 33 as an internal control, as noted in the figure panel legend. **c** Pseudotime analysis of human abdominal adhesion fibroblast scRNA-seq data. Colors match cluster colors assigned in panel **a. d** Quantitation of qPCR analysis for *JUN*, *STAT5*, *STAT3*, and *SPP1* of vehicle control versus *JUN* CRISPR Cas9-knockdown human abdominal adhesions fibroblasts. *P*-values noted in figure. *n* = 3 replicates per condition, datapoints represent average of technical replicates. **e** Quantitation of Ki67 expression using ICC of primary human abdominal adhesions fibroblasts treated with CRISPR Cas9 *JUN* knockdown compared with vehicle control. *n* = 4 biological replicates assessed per condition. **f** Quantitation of collagen type 1 expression using ICC of primary human abdominal adhesions fibroblasts treated with virally mediated JUN overexpression, vehicle control, or CRISPR Cas9 *JUN* knockdown. *n* = 3 biological replicates assessed per condition. **g** Schematic (based on published KEGG pathways) displays proposed *JUN*-relevant signaling pathways identified in this study. The left panel shows the acute phase response following tissue injury by which *JUN* is initially activated. The right panel shows the chronic profibrotic state that is established in adhesion fibroblasts. Blue circle highlights AP-1, red indicates the role of JUN inhibitor. Colors as labelled in the figure. ECM extracellular matrix. Data and error bars represent means ± SD. HPF high power field. A.U. arbitrary units. *P = 0.04, **P = 0.03, ***P = 0.03, unpaired two-tailed *t*-test. Source data are provided as a Source Data file.

To explore heterogeneity among human adhesion fibroblasts at the transcriptional level, we isolated adhesion fibroblasts from three surgical patients (Supplementary Table 3) and conducted scRNA-seq using the 10x Genomics platform. Cells from individual patient specimens were labelled with hashtag oligos in order to explore any differences that might be seen between fibroblasts isolated from adhesions from different patients. The sequencing data show 4 distinct clusters of human adhesion fibroblasts based on gene expression (Fig. 8a, Supplementary Fig. 19a), with considerable heterogeneity noted among all fibroblasts (Supplementary Fig. 19b). This heterogeneity is not attributable to differences between individual patient specimens (Fig. 8b); in fact, cells from the three patient specimens analyzed are represented relatively evenly across the four clusters, suggesting that there is a common/shared adhesions gene expression phenotype across different patients (irrespective of differences in gender or reason for surgery, for example).

We also conducted CytoTRACE analysis on the human adhesion fibroblast scRNA-seq data, as with our mouse dataset above. Unlike in our mouse RNA-seq analysis, a clear pattern of differentiation was not identified in these human fibroblasts, suggesting that the cells we evaluated from human adhesion specimens existed in an established, profibrotic steady state. This is supported by the diverse phenotypic nature of these samples, which were collected from independent patients many months after abdominal surgery (range: 9–19 months postoperatively). The difference in time after surgery between patients did not appear to be a driver of transcriptional clustering, further supporting the concept of a common, chronic fibrosis state (Supplementary Fig. 19c). We also applied pseudotime analysis to compare the properties of the identified fibroblasts subpopulations (Supplementary Fig. 19d—left panel). Two branch points were identified (Fig. 8c), with representation of all specimens in all transcriptionally-defined clusters (Supplementary Fig. 19d—right panel), again suggesting that the differences in time after surgery between patients (range 9–19 months after initial surgery) was not a significant driver of subsequent meta-state distribution. These data support the conclusion that adhesion fibroblasts maintain a chronic, fibrosis state indefinitely following activation. This is in line with the clinical observation that once formed, abdominal adhesions persist indefinitely in patients.

Next we explored the expression of specific genes in relation to the clustering of our human adhesion fibroblast scRNA-seq data. *JUN* and *STAT3* are expressed throughout all clusters and by all patients. *FSP1* is strongly expressed by nearly all cells assessed, while *ASMA* expression is found throughout but highest in clusters 1 and 2, and *PDGFRA* expression is highest in clusters 0 and 3 (Supplemental Fig. 20a).

GO enrichment analysis of these human adhesion fibroblast scRNA-seq clusters revealed less enrichment of components involved in acute phase processes compared with our mouse data, and instead demonstrated comparative enrichment of oxidative phosphorylation and ATP metabolism. Such processes suggest that maintenance of the adhesion fibroblast profibrotic state may be associated with alterations in local metabolic programming. Focal adhesion and cell-substrate junction processes are also differentially enriched among all clusters, suggesting that the altered tissue mechanics found in fibrosis such as adhesions likely play a persistent role in meta-state regulation. Extracellular matrix and components are also strongly enriched throughout all clusters consistent with the fibrotic phenotype observed. *WNT* pathway signaling is also enriched particularly in cluster 1 (Supplementary Fig. 20b); *JUN* is a known transcription factor partner in non-canonical *WNT* pathway signaling^33^. Taken together these data support *JUN* as a primary mediator of fibrosis—which occurs through several downstream pathways—in abdominal adhesions in humans.

To functionally validate the role of *JUN* in human adhesion formation, CRISPR Cas9 was used to knockdown *JUN* expression in freshly isolated human abdominal adhesion fibroblasts. Knockdown was confirmed at the protein level (Supplementary Fig. 21a, b). qPCR assay of *JUN*-knockdown fibroblasts showed a decrease is expression of profibrotic genes including *JUN*, *STAT3*, *STAT5*, and *SPP1* (Fig. 8d). At the protein level, we found that CRISPR Cas9 *JUN* knockdown resulted in a significant decrease in human adhesion fibroblast proliferation (assessed via Ki67 immunofluorescence) compared with control—freshly isolated adhesion fibroblasts (Fig. 8e). To further validate these results, we applied virally mediated *JUN* overexpression to primary human adhesion fibroblasts. This caused significant upregulation of phospho-JUN expression compared with control or JUN inhibitor (validated in a fibroblast cell line, Supplementary Fig. 22a, b). Compared alongside vehicle control, primary human adhesion fibroblasts and those treated with CRISPR Cas9 *JUN*-knockdown collagen expression at the protein level was significantly elevated with *JUN* overexpression in primary human abdominal adhesions fibroblasts compared with control or CRISPR Cas9 *JUN* knockdown (Fig. 8f).

These human data are consistent with our mouse data, supporting that *JUN* is a key initiating factor for adhesion formation in human tissue. Taken together, these data support *JUN* as a transcriptional master regulator of fibroblasts. Mechanical injury in the form of abdominal surgery or intra-abdominal infection insights a local wound healing response that involves platelet aggregation at the site and associated PDGF and IL6 release. These factors initially precipitate *JUN* expression (Fig. 8g—left panel). Once induced, *JUN* signaling auto-amplifies, acting as a profibrotic transcription factor via several downstream signaling responses including *JAK*-*STAT* and EMT pathways. These fibroblasts take over production of IL6 and related factors resulting in a chronic, high-*JUN*, profibrotic state through which adhesions are maintained indefinitely (Fig. 8g—right panel). Suppression of *JUN* pathway signaling is sufficient to block this process and dramatically decrease adhesion formation.

## Discussion

In summary, abdominal adhesions constitute a major medical problem for millions of patients for which effective therapeutic options are needed. In this study, we systemically explored abdominal adhesion biology in vivo in parallel in mice and humans at the tissue, transcriptomic, and protein level. We found that *JUN* expression is an early promotor of abdominal adhesions, which upregulates signaling of several pathways known to result in fibrosis including *JAK*-*STAT*, EMT, as well as *PDGFRA* expression. In both mice and human tissue, we elucidated the identity of adhesion fibroblasts, which can be isolated based on expression of JUN, PDGFRA, ASMA, and FSP1.

Adhesions are formed by local fibroblasts and proliferate polyclonally following injury, suggesting that this clinical phenomenon involves progenitor-type cell activation. We developed a model for abdominal wall transplantation and show that the adhesion fibroblast population derives primarily from the visceral peritoneum, confirming our observation in clinical surgery that adhesions are most prominent after open abdominal surgery with manipulation of the bowel (rather than laparoscopy in which frequently only the parietal peritoneum is affected). One limitation associated with our abdominal wall transplant model is that the blood supply to the transplanted potion is interrupted with transplant, so the transplant is reliant on plasma imbibition during the first 48 h post-transplant, much like a skin graft.

In both mouse and human RNA-seq data, epithelial-mesenchymal transition (EMT) and *JUN* pathway signaling are significantly upregulated at the transcriptional level. On single-cell RNA-seq, adhesion fibroblasts from both mice and humans are heterogeneous, with three transcriptionally distinct fibroblast clusters in the mouse data and four in humans. On pseudotime analysis, validated at the protein level, *JUN* is expressed early in mouse tissues and maintained over time. In human adhesion fibroblasts harvested many months after surgery, *JUN* expression is ubiquitous and maintained indefinitely. Functionally, JUN inhibition significantly suppresses adhesion formation in vivo in mice, and *JUN* knockdown suppresses profibrotic gene and protein expression, as well as proliferation, in human adhesion fibroblasts in vitro. As such, modulation of local *JUN* expression shows potential toward clinically minimizing adhesion formation.

## Methods

### Animals

The following mouse strains were purchased from Jackson Laboratories: Black/6 (C57BL/6 J), ROSA26^mTmG^ (B6.129(Cg)-Gt(ROSA)26Sortm4(ACTB-tdTomato,-EGFP)Luo/J)^34^, PDGFRα^GFP^ (B6.129S4-Pdgfrαtm11EGFPSor/J), PDGFRα^CreERT2^ (B6N. Cg-Tg (Pdgfrα-cre/ERT)467 Dbe/J), eGFP (C57BL/6J-Tg(CAG-EGFP)10 sb/J), EN1^Cre^ (En1-tm2(cre)Wrst/J), Wt1^CreERT2^ (Wt1-tm2(cre/ERT2)Wtp/J), and ACTIN^CreERT2^ mice (Tg(CAG-cre/Esr1)5Amc/J). Rainbow mice (ROSA26^VT2/GK3^) were provided as a gift from the Weissman Laboratory, Stanford University School of Medicine. *JUN* mice (flp-in tetO-c-JUN) were provided by the Wernig Laboratory, Stanford University School of Medicine. Mice were housed at the Stanford University Comparative Medicine Pavilion (CMP) and Research Animal Facility (RAF). The facilities provided light- and temperature-regulated housing for all animals. Mice were given rodent chow and water ad libitum. A minimum sample size of three animals was used for all experiments (exact numbers for experiments are provided in the figure legends). Ten-week-old mice with appropriate genotypes for a given experiment were randomly allocated to the various experimental conditions. Healthy litter mates were used as controls. All experiments were carried out in accordance with the Stanford University Animal Care and Use Committee standards of care. This study complies with all relevant ethical regulations for research with research animals. All mouse experiments were conducted under the guidance and approval of Stanford University’s IACUC/APLAC.

### Mouse model for adhesions

A similar model for abdominal adhesion formation has been published by our laboratory^8^. In brief, mice were anesthetized with inhaled isoflurane (Henry Schein Animal Health,) at a concentration of 1–2% in oxygen at 3 L/min. Ophthalmic ointment (Puralube petrolatum, Dechra Veterinary Products) was applied to the cornea to prevent desiccation. Buprenorphine (Buprenex, Reckitt Benckiser Pharmaceuticals Inc.) was administered subcutaneously prior to the surgery at a dose of 0.1 mg/kg. The mice were placed in the supine position on a clean operating surface. A heating pad was used beneath the surgical field to keep the animals warm throughout the procedure. The abdomens were shaved, and the skin of the abdomen was sterilized with three applications of betadine followed by 70% ethanol. During the procedure, the respiratory rate of the animals was monitored, and the isoflurane was titrated accordingly. A vertical midline skin incision was made in the abdomen with sharp scissors. The underlying abdominal wall was then opened vertically. The cecum was located and exteriorized. The cecal wall was abraded gently with 150-grit sandpaper. The parietal peritoneum along the abdominal wall was similarly abraded with sandpaper and three, single, interrupted, 4–0, silk sutures were placed into the right abdominal sidewall, which served as a nidus for adhesion formation. The cecum was then placed anatomically adjacent to the right abdominal wall. The abdomen was briefly irrigated with warmed, sterile, normal saline. The irrigant was removed by blotting with a gauze sponge. The abdominal wall incision was closed with running 6–0 Monocryl suture, and the skin incision was closed with 6–0 nylon, horizontal mattress sutures. The animals were monitored during recovery from anesthesia. Additional buprenorphine was given every 6–12 h as needed for pain. For sham surgery, animals were treated in an identical manner; however, incisions were closed without manipulation of abdominal organs.

### Liposomal tamoxifen induction

Activated 4-hydroxytamoxifen liposomes (LiTMX) were prepared according to the protocol developed by Ransom et al., 2018^21^. In brief, a 90:10 mol:mol mixture of DMPC (1,2-dimyristoyl-*sn*-glycero-3-phosphocholine) and cholesterol (Avanti Polar Lipids) was desiccated under nitrogen gas followed by vacuum desiccation, sonicated in 1× PBS, and reconstituted by extrusion. Liposome size was characterized using NanoBrook Omni dynamic light scattering (DLS) instrument (Brookhaven). Liposomal vesicles were then incubated with 4-hydroxytamoxifen (Sigma–Aldrich) under nitrogen gas. LiTMX were applied locally to the visceral and parietal peritoneum at the site of interest for induction of Cre Recombinase at the time of adhesion surgery. The adhesion procedure was conducted otherwise as described above.

### Abdominal wall transplant

A 1 × 1 cm section of abdominal wall (full thickness muscular layer and parietal peritoneum) was harvested from PDGFRA^GFP^::ROSA26^mTmG^ mice under surgical conditions. The same size section of abdominal wall was excised from PDGFRA^GFP^ mice. The PDGFRA^GFP^::ROSA26^mTmG^ transplants were rapidly transplanted and sutured into place in the PDGFRA^GFP^ mice using 4–0 silk interrupted sutures. Adhesions were created as previously described and harvested at postoperative day (POD)14.

### In vivo *JUN* induction and suppression

Doxycycline (2 mg/mL) or T-5224 (Cayman Chem, 10uM) resuspended in DMSO were applied locally to adhesion sites at the time of adhesion surgery for *JUN* induction (in *JUN* mice) or suppression (in *JUN* and WT mice), respectively. Vehicle only was used for control.

### Parabiosis model

Parabiotic mouse pairs were created following a previously published protocol^35^. Briefly, both age- and sex-matched wild-type (WT, C57BL/6) and eGFP-labeled mice (C57BL/6-Tg(CAG^EGFP^)10 sb/J) were housed together for two weeks prior to parabiosis surgery. Mice were anesthetized; the sides of the mice were shaved and cleaned with betadyne and 70% ethanol as previously described. An incision was made from the base of the right foreleg to the base of the right hind leg on the WT mice and an identical incision was made on the left leg of the eGFP mice. The skin was sutured together. Peripheral blood chimerism was determined two weeks later via flow cytometry. Adhesion surgeries were then performed on the WT mice.

### Tissue processing and histology

Mouse and human adhesion and control tissues were fixed in 4% paraformaldehyde (Electron Microscopy Sciences) for 20 h at 4 °C and embedded into paraffin per standard protocols. For cryopreservation, specimens were placed in 30% sucrose (Sigma) until saturation at 4 °C following fixation, followed by OCT until saturation at 4 °C, and then embedded in OCT. Representative tissue specimens were stained with hematoxylin and eosin (H&E, Sigma–Aldrich), Picrosirius Red Stain (Abcam), or Masson’s trichrome (Sigma–Aldrich) per manufacturer’s protocols.

### Gross and histologic scoring of adhesion tissue

Both gross and histologic scoring systems were used to evaluate extent of adhesion formation in mice. Scoring of adhesion tissue from gross images was achieved using the adhesion scoring system previously described by Tsai et al.^19^. The histologic scoring system used was adapted from Tsai et al. and Linksy et al.^36^ and applied to H&E-stained specimens. In brief, a histologic adhesion score of 0 was assigned to histological specimens with no apparent adhesion between peritoneal surfaces. A score of 1 indicated ‘string-like’ adhesions or ≤10% of contact with adherent tissue. A score of 2 indicated noncontinuous adhesions, thicker than strings with ≤25% contact with adherent tissue. A score of 3 indicated noncontinuous but dense adhesion with ≤50% contact with adherent tissue. A score of 4 included specimens with continuous adhesion with multiple (≤75%) points of contact with adherent tissue. A score of 5 was characterized by dense (100%), continuous contact with adherent tissue.

### Cell culture

Fibroblasts were resuspended in media (DMEM supplemented with 10% FBS) and seeded into a plate (Falcon) coated with EmbryoMax™ ultrapure water with 0.1% gelatin (Millipore). All cells were maintained under sterile conditions in a humidified incubator under 5% CO2 at 37 °C. A phase-contrast microscope (Leica) was used to image cells.

### Immunocytochemistry (ICC)

Coverslips were coated with 1% Embryomax gelatin (EMD Millipore). Adhesion and control fibroblasts were seeded onto the coverslips. Once stuck, the cells were fixed, permeabilized with 0.5% Triton-X-100 (Sigma), and then incubated with 1X Powerblock (Biogenex). The cells were then stained with primary antibody at 4 °C overnight. The following day, the cells were washed with 0.1% Tween-20 (PBST; Sigma–Aldrich), then stained with secondary antibody, and incubated at room temperature for 1 h. The slides were then mounted using Prolong Gold Antifade Mountant with DAPI (Life Technologies).

### Immunofluorescence (IF)

Cryopreserved specimens were cryosectioned onto Superfrost Plus microscope slides (FisherSci). The sections were permeabilized with 0.5% Triton-X-100 (Sigma), and then incubated with 1X Powerblock (Biogenex). Primary antibodies were applied to tissue specimens for 1 h at room temperature, and then rinsed repeatedly. Secondary antibodies were applied for one hour at room temperature. The antibody incubation and washing steps were repeated if multiple proteins were stained for in one specimen section. Slides were then mounted using Prolong Gold Antifade Mountant with DAPI (Life Technologies). Antibodies used for ICC and IF included: Anti-phospho-JUN (Cell Signaling, S63 (54B3), lot: 7, used at 1:100), anti-JUN (Abcam, ab31419, lot: GR306615-18, used at 1:50), anti-aSMA (Abcam, ab32575, lot: GR282976-32, used at 1:100), anti-FSP-1/S100A4 (Abcam, ab41532 lot: GR3176834-1, used at 1:200), anti-COL3 (Abcam, ab7778, lot: GR3234897-1, used at 1:100), anti-COL1 (Abcam, ab34710, lot: GR3244041-2, used at 1:100), anti-MSLN (ABBiotec, 250519, lot: 15102712, used at 1:100), anti-CD26 (Abcam, ab222716, lot: GR3220836-1, used at 1:100), anti-vimentin (Abcam, ab11256, lot: GR236597-5, used at 1:20), anti-phospho-FAK (Thermo Fisher, 799255, lot: RG240925A, used at 1:100), anti-PDPN (Invitrogen, MA5-29742, lot: UB2724771, used at 1:250), anti-CD10 (Abcam, ab227640, lot: GR3227478-1, used at 1:100), anti-CD31 (Abcam, ab28364, lot: GR3247742-7, used at 1:50), anti-CD45 (Abcam, ab10558, lot: GR269008-1, used at 1:150), anti-phospho-Stat5 (Cell Signaling, 9314 S, used at 1:200), anti-PDGFRa (Abcam, ab203491, lot: GR3226597-1, used at 1:200), IgG Alexa-Fluor 488 (Invitrogen, A32731, lot: SH251139, used at 1:1000), IgG Alexa-Fluor 555 (Invitrogen, A32732, lot: SH251140, used at 1:1000), IgG Alexa-Fluor 647 (Invitrogen, A32733, lot: SI231745, used at 1:1000).

### Confocal imaging and analysis

Laser scanning confocal microscopy was performed using a Leica WLL TCS SP8 Confocal Laser Scanning Microscope (Leica Microsystems) located in the Cell Sciences Imaging Facility (Stanford University, Stanford, CA). The ×10, ×20, and ×40 objectives were used (×10 HC PL APO, air, N.A. 0.40; ×20 and ×40 HC PL APO IMM CORR CS2, H2O/Glycerol/oil, N.A. 0.75). Raw image stacks were imported into Fiji (Image-J, NIH) or Imaris (Bitplane) software for analysis. Fiji was used to make two-dimensional micrographs of the confocal data and to quantify fluorophore expression intensity. For analysis of clonality from Rainbow mouse tissue, surfaces were created for each color of the Rainbow construct expressed using the volume surface and thresholding tools in Imaris.

### Recruitment of human specimens

Human abdominal tissue specimens were obtained from patient’s undergoing abdominal surgery at the Stanford Hospital under Stanford University’s IRB approval. Tissue specimens included only tissues that would otherwise have been discarded. Inclusion criteria for patients were as follows: For all patients, patients must be over the age of 18, surgery must be elective, and there must be no evidence of active inflammation or infection at time of operation. For adhesion specimens—patients must have had at least one prior abdominal surgery, for control specimens—patients must have had no history of prior abdominal surgery. The patients were approached in the preoperative area by one of the manuscript authors. The aims of the study were discussed with the patient. Participation was entirely voluntary. Written, informed consent was obtained from the patient prior to surgery. Tissue specimens were collected by one of the authors on this manuscript from the primary surgeon in the operating room, placed directly into sterile saline, and kept on ice for transport. Tissue specimens were processed immediately. This study complies with all relevant ethical regulations for research with human participants.

### Sample preparation and FACS isolation

Abdominal tissue specimens from mouse or human specimens was minced on ice. The tissue was then digested for 60 min in a 37 °C water bath agitator in 2 mg/mL collagenase (collagenase type IV, ThermoFisher) digest buffer in Medium 199 (HyClone, GE Healthcare) consisting of 5% fetal bovine serum (Gibco FBS, ThermoFisher), DNase I (Worthington), Poloxamer 188 (Cat. P5556-100ML, Sigma), HEPES, and CaCl2. The digest was quenched with quench media (DMEM (Gibco DMEM, ThermoFisher) with 15% FBS), then centrifuged at 300 × *g* for 5 min at 4 °C, resuspended in quench media, and filtered through 100, 70, and 40 μm cell strainers (Falcon cell strainer, ThermoFisher). Red blood cell lysis was performed using Hybri-Max (Sigma) per the manufacturers protocol. Histopaque was performed using Histopaque-1119 (Sigma–Aldrich), per the manufacturers protocol.

Cells were counted and resuspended in FACS buffer. Primary antibodies were applied, and cells were stained in the dark with gentle agitation for 30 min. Cells were then washed thoroughly in FACS buffer. Staining with secondary antibodies was conducted in the same manner. Propidium iodine (PI, Thermofisher, Cat. P3566, lot: 1755970, 3 μg/mL) or DAPI (Thermofisher, Cat. 3571) were used as a viability marker. Fibroblasts were isolated using the FACS Aria II system. For RNA sequencing, cells were sorted into chilled lysing reagent under RNA/DNAse-free conditions (Trizol LS, ThermoFisher). Flow-cytometry plots shown are representative of at least three independent experiments.

Antibodies against the following cell surface markers primarily or secondarily conjugated to the same fluorophore were used for exclusion of “lineage” cells in mouse and human specimens in order to isolate fibroblasts in an unbiased manner: CD45, CD31, Ter119, Tie2, CD324, and CD326. This approach has been previously validated by our laboratory in other fibrotic pathologies^9,37^.

For phospho-specific flow-cytometry analysis, a single-cell suspension was prepared using manual tissue dispersion rather than enzymatic digestion to preserve phosphorylated signal, and then prepared using the BD Biosciences Cytofix/Cytoperm™ kit according to manufacturer’s instructions. Phosphorylated protein analysis was conducted using the FACS Aria II system.

Antibodies used for FACS included: Anti-phospho-JUN (Cell Signaling, s73d47G9, lot: 5, used at 1:200), anti-phospho-STAT5-PECy7 (BD Biosciences, 560117, lot: 8266820, prediluted and used at manufacturer’s volume per test of 20 ul), anti-FSP-1 (Ray biotech, 188-11191, lot: 1804128, used at 10 ug/mL), IgG Pacific Blue (Thermo Fisher, p31582, lot: 1929717, used at 1 ug/mL), anti-PDGFRα (Abcam, ab90967, lot: gr321324-2, used at 10 ug/mL), IgG Alexa-Fluor 647 (Abcam, ab150159, lot: GR241187-2, used at 1:2000), IgG Alexa-Fluor 488 (Abcam, ab150077, lot: GR3224145-2, used at 1:2000), IgG Alexa-Fluor 647 (Abcam, ab: 150075, lot: GR269275-2, used at 1:2000), IgG Pacific Blue (Thermo Fisher, P10994, lot: 2045342, used at 1 ul/mL), anti-S100A4-PE (BioLegend, 370003; lot b286200, used at 1:20), anti-PDGFRa (Abcam, ab203491, lot: GR3226597-1, used at 1:50), anti-CD26 PECy7 (Biolegend, 302713, lot: B253866, used at 1:20), anti-CD45-FITC (Invitrogen, 11-9459-42, lot 4319940, used at 1:20), anti-Ter119-FITC (Invitrogen, 11-5921-85, lot :4322597, used at 2.5 ug/mL), anti-CD31-FITC (Thermo Fisher, 11-0311-82, lot: B224877, used at 1:100), anti-Tie2 (Thermo Fisher, 14-5987-82, lot: 2072830, used at 1:100), anti-CD324 (Biolegend, 147302, lot: B228369, used at 10 ug/mL), anti-CD326 (Biolegend, 118202, lot: B254013, used at 0.6 ug/mL), 488 secondary (Abcam, ab150157, used at 1:2000), anti-CD45-eFluor 450 (Invitrogen, 48-0451-82, lot: 1936503, used at 5 ug/mL), anti-Ter119-Pacific Blue (Invitrogen, 48-5921-82, lot: 1974934, used at 5 ug/mL), anti-CD31-eFluor 450 (Invitrogen, 48-0311-82, lot: 1982691, used at 2.5 ug/mL), anti-Tie2-biotin (Invitrogen, 13-5978-82, lot: 4304957, used at 5 ug/mL), anti-CD324-biotin (Invitrogen, 13-3249-82, lot:1916204, used at 2.5 ug/mL), eFluor 450-Streptavidin (Invitrogen, 48-4317-82, lot 1988686, used at 2.5 ug/mL), anti-CD326-eFluor 450 (Invitrogen, 48-5791-82, lot: 1984115, used at 10 ug/mL), anti-CD45-PECy7 (Thermo Fisher, MHCD4512, used at 1:100), anti-Ter119-PECy7 (Invitrogen, 25-5921-82, lot: 1994153, used at 5 ug/mL), anti-CD31-PECy7 (Invitrogen, 25-0311-81, lot 4318668, used at 5 ug/mL), anti-Tie2 (Invitrogen, 14-5987-82, lot: 2072830, used at 10 ug/mL), anti-CD326-PECy7, (BioLegend, 324221, lot: B266928, used at 1:20), and anti-CD324-PECy7 (Biolegend, 147310, lot: B255274, used at 2.4 ug/mL).

FACS gating and data analysis was performed using FlowJo. Gating schemes were established with fluorescence-minus-one controls. Single cells were first gated using FSC and SSC parameters. Dead and lineage-positive (non-fibroblast) cells were then excluded by gating against PI or DAPI, and lineage panel antibody staining, respectively. Gating schemes to quantitate and/or isolate fibroblasts and specific fibroblast subpopulations of interest were validated by plating a portion of the sorted cells for morphological visualization, immunocytochemistry, and/or qPCR assay.

### Bulk mRNA sequencing

For mouse and human specimens, RNA extraction was performed using Qiagen miRNeasy kit (cat. 1071023) with on column DNase treatment per the manufacturer’s recommendations. The Clontech Smarter Ultra Low Input RNA kit (Takara Bio, Cat. 634848) was used to generate cDNA from 150 pg total RNA following the manufacturer’s recommendations. Amplified cDNA was purified using SPRI Ampure Beads (Beckman Coulter, Cat. A63880) and the quality and quantity were measured using a High Sensitivity DNA chip on the Agilent 2100 Bioanalyzer (Agilent Technologies). cDNA was sheared to an average length of 300 basepairs using a Covaris S2 ultrasonicator (Covaris) and libraries were generated with the Clontech Low Input Library Prep kit (Takara Bio, Cat. 634947). The samples were uniquely barcoded, pooled, and sequenced on a single lane of the NextSeq 500 (Illumina). A total of 300 million paired-end, 151 base pair reads were obtained, resulting in 50 million reads per sample.

### Bulk mRNA sequencing data analysis

A total of nine human (six adhesions and three controls) and eight mouse (four adhesions and four sham-surgery controls) samples were profiled by bulk RNA sequencing as described above. Raw FASTQ reads were aligned to GENCODE v29 reference transcripts (GRCh38.p12) for human and GENCODE vM20 reference transcripts (GRCm38.p6) for mouse with Salmon^38^ v0.12.0 using the–seqBias,–gcBias,–posBias,–useVBOpt,–rangeFactorizationBins 4, and–validateMappings flags and otherwise default parameters for single-end mapping. Salmon results were merged into a single gene-level counts matrix using the R package, *tximport*^39^ v1.4.0.

Count normalization and differential gene expression analysis was performed using the DESeq2 v1.22.2 package in R^40^. Counts were size-factor normalized using the ‘DESeq’ function and log_2_-transformed. Pairwise differential gene expression analysis was performed using the lfcShrink function and indicating ‘type = apeglm’, which applies the adaptive t prior shrinkage estimator. As recommended^40^, a threshold of *P*-adjusted <0.1 was used to define significance for differentially expressed genes (Supplementary Data Sets 1 and 2).

Gene set enrichment analysis (GSEA) was performed on preranked gene lists of differentially expressed genes (*n* = 851 genes for mouse, *n* = 3003 genes for human) ordered by log_2_-fold change using the GSEA software provided by the Broad Institute^41^. The “HALLMARK_EPITHELIAL_MESENCHYMAL_TRANSITION” and “GO_REGULATION_OF_JUN_KINASE_ACTIVITY” gene sets were used to highlight genes involved in epithelial-to-mesenchymal (EMT) and *JUN* signaling pathway. Hypergeometric tests to analyze enrichment of gene ontology (GO) terms in the genes differentially expressed in the “Adhesion” versus “Sham” (mouse, *n* = 451 genes) or “Healthy” (human, *n* = 1443 genes) groups were performed using the clusterProfiler v3.10.0^42^ package in R.

### Single-cell barcoding, library preparation, and sequencing

Adhesion fibroblasts were FACS-isolated from mouse and human specimens using an unbiased, lineage-based strategy as previously described. Four mouse specimens derived from litter mates were used pooled for each timepoint (POD 2 and POD *7)*. Individual human specimens were tagged with hashtag oligos (HTOs) per the manufacturer’s protocol and then pooled. Cells were counted and filtered just prior to loading into the 10× machine.

Single cells were barcoded using the 10x Chromium Single Cell platform, and cDNA libraries were prepared according to the manufacturer’s protocol (Single Cell 3’ v3, 10x Genomics, USA). In brief, cell suspensions, reverse transcription master mix and partitioning oil were loaded on a single-cell chip, then run on the Chromium Controller. Reverse Transcription was performed within the droplets at 53 °C for 45 min. cDNA was amplified for a 12 cycles total on a BioRad C1000 Touch thermocycler. cDNA size selection was performed using SpriSelect beads (Beckman Coulter, USA) and a ratio of SpriSelect reagent volume to sample volume of 0.6. cDNA was analyzed on an Agilent Bioanalyzer High Sensitivity DNA chip for qualitative control purposes. cDNA was fragmented using the proprietary fragmentation enzyme blend for 5 min at 32 °C, followed by end repair and A-tailing at 65 °C for 30 min. cDNA were double-sided size selected using SpriSelect beats. Sequencing adaptors were ligated to the cDNA at 20 °C for 15 min. cDNA was amplified using a sample-specific index oligo as primer, followed by another round of double-sided size selection using SpriSelect beads. Final libraries were analyzed on an Agilent Bioanalyzer High Sensitivity DNA chip for qualitative control purposes. cDNA libraries were sequenced on a NextSeq 500 Illumina platform aiming for 50,000 reads per cell.

### Data processing, fastq generation, and read mapping

Base calls were converted to reads with the software Cell Ranger (10x Genomics; version 3.1)‘s implementation *mkfastq*. These were then aligned against either the GRCh38 v3.0.0 (for human) or mm10 v3.0.0 (for mouse) genomes using Cell Ranger’s *count* function (an implementation of STAR v2.7.0) with SC3Pv3 chemistry and 5000 expected cells per sample^43^. Cell barcodes representative of quality cells were delineated from barcodes of apoptotic cells or background RNA based on a threshold of having at least 1000 transcripts profiled and less than 5% of their transcriptome of mitochondrial origin. For human samples, this resulted in 1542 unique genes detected per cell, 6489 UMIs per cell, and 3.44% mitochondrial genes per cell. For mouse samples, we found 4183 unique genes detected per cell, 23,586 UMIs per cell, and 3.17% mitochondrial genes per cell.

### Data normalization, hashtag oligo demultiplexing, and cell subpopulation identification

UMIs from each cell barcode were retained for all downstream analysis. Raw UMI counts were normalized with a scale factor of 10,000 UMIs per cell and subsequently natural log transformed with a pseudocount of 1 using the R package Seurat (version 3.1.1)^44^. Hashtag oligos (HTOs) for human samples were demultiplexed using Seurat’s implementation *HTODemux*. Briefly, k-medoid clustering is performed on the normalized HTO values, after which a ‘negative’ HTO distribution is calculated. For each HTO, the cluster with the lowest average value is treated as the negative group and a negative binomial distribution is fit to this cluster. Using the 0.99 quantile of this distribution as a threshold, each cell is classified as positive or negative for each HTO. Cells that are positive for more than one HTOs are annotated as doublets and removed. Cells that are not positive for any HTO are also removed. Aggregated data were then evaluated using uniform manifold approximation and projection (UMAP) analysis over the first 15 principal components^45^. Cell annotations were ascribed using SingleR (version 3.11) against the Blueprint + ENCODE reference database for human cells, and against the Immunological Genome Project (ImmGen) and mouse RNA-seq reference sets for mouse cells.

### Generation of characteristic subpopulation markers and enrichment analysis

Cell-type marker lists were generated with two separate approaches. In the first approach, we employed Seurat’s native *FindMarkers* function with a log fold change threshold of 0.25 using the ROC test to assign predictive power to each gene. However, in order to better account for the mutual information contained within highly correlated predictive genes, we also employed a characteristic direction analysis^46^. The 50 most highly ranked genes from this analysis for each cluster were used to perform gene set enrichment analysis against the BROAD Institute databases (http://software.broadinstitute.org/gsea/index.jsp) in a programmatic fashion using EnrichR (version 2.1)^47^. Pseudotime analysis was performed using the Monocle2 package in R (version 2.4.0)^29^. Single-cell differentiation states were predicted in R using the CytoTRACE package publicly available at https://cytotrace.stanford.edu/^28^. The top 200 genes, or gene counts signature (GCS), from two independent datasets, mouse lung fibroblast and human mesoderm development, were extracted from precomputed files on the CytoTRACE website. The geometric mean of these top 200 genes was then calculated in our scRNA-seq datasets and visualized in a low-dimensional embedding.

### Real-time (RT) quantitative (q)PCR

RNA was extracted from mouse and human specimens using the Direct-Zol RNA extraction kit (Zymo Research) per the manufacturer’s guidelines. RNA concentrations were assessed using Nanodrop (ThermoFisher). cDNA libraries were created using the high capacity cDNA reverse transcription kit (Applied Biosystems) as per the manufacturer’s instructions. The reverse transcription reaction was performed using a 2720 Thermal Cycler (Applied Biosystems). Power SYBR Green Master Mix (Applied Biosystems) was used for amplification using a 20 µl reaction mixture per the manufacturer’s guidelines. The sequences of primers used are listed in Supplementary Table 4. Each experiment was carried out in triplicate for each datapoint, and the cycle threshold (Ct) value was used for analysis. Mean fold-changes in gene expression were normalized against GAPDH for mouse specimens and beta-actin for human samples.

### Human adhesion fibroblast cytokine analysis

Freshly isolated human adhesion fibroblasts were plated in antibiotic-free media and cell supernatant was collected at 24 and 48 h after plating, as well as cell-free media negative control. The 62-plex Luminex assay (custom-built by eBioscience) to assess cytokine content in the supernatant was conducted per the manufacturer’s protocol. Three biological replicates were analyzed per timepoint. Median fluorescence intensity was used for quantification.

### CRISPR-mediated genome engineering

Following the protocol from reported literature^48,49^, the sequences of the site-specific guide RNAs (sgRNAs) were selected using the online CRISPR Design Tool from Feng Zhang’s lab (https://zlab.bio/guide-design-resources). Oligonucleotides with these sequences were cloned into the lentiCRISPRv2 vector (AddGene), which are shown in Supplementary Table 5. The transfer plasmid (*JUN* CRISPR knockout plasmid) was then co-transfected with a pRRE Packing plasmid (GAG and Pol genes), a pRSV Packing plasmid (Rev gene), and a pMD2.G enveloping plasmid into HEK293T cells. The cell media was collected, ultracentrifuged, and frozen for use.

### In vitro T-5224 treatment

Freshly isolated mouse adhesion fibroblasts were grown to 90% confluence. Cultured cells were lifted using TrypLE (ThermoFisher Scientific) and replated the day of treatment in antibiotic-free media (DMEMF12 and 10% FBS). The cells were allowed to adhere, culture media was then removed and replaced with 0.1% bovine serum albumin (BSA) media for one hour. All cells were then stimulated with insulin for 1 h (5 mg/ml). Wells were randomly assigned to treatment versus control conditions; treatment wells received T-5224 treatment (10 µM), control wells received vehicle only. Cells were lifted 24 h later, and phospho-protein expression was analyzed using flow cytometry as previously described.

### CRISPR Cas9 *JUN*-knockdown treatment

Freshly isolated human adhesion fibroblasts were grown to 90% confluence. Cultured cells were lifted using TrypLE and replated the day of treatment in antibiotic-free media. The cells were allowed to adhere, culture media was then removed and replaced with 0.1% bovine serum albumin (BSA) media for 1 h. Cells were then stimulated with insulin for 1 h (5 mg/ml). Wells were randomly assigned to treatment, vehicle control (“induced control”), and vehicle-control-selection conditions; treatment wells received CRISPR Cas9 virus, vehicle control received vehicle only. CRISPR Cas9 and vehicle-control-selection wells received puromycin selection (1 µg/ml) dosed every 24 h until complete cell death was seen in the vehicle-control-selection wells. Selection was further confirmed via flow cytometry. Cells from the treatment and vehicle-control wells were then lifted and processed for analysis.

### Lentivirus preparation for virally mediated *JUN* overexpression

Ninety percent confluent 293 T cells were transfected with 4 μg Transfer plasmid (*JUN* tet-on overexpression plasmid, tetracycline controllable transactivator plasmid, *JUN* CRISPR knockout plasmid, TK control reporter plasmid, E7TK *CD47* enhancer reporter plasmid and Luciferase-GFP plasmid), 2 μg pRRE Packing plasmid (GAG and Pol genes), 1 μg pRSV Packing plasmid (Rev gene), 1 μg pMD2.G enveloping plasmid, and 24 μg PEI. The cell media was collected and centrifuged, then the supernatant was filtered through a 0.22 μm strainer, ultracentrifuged, and flash frozen for use.

### Virally mediated JUN overexpression treatment

NIH 3T3 and freshly isolated adhesion fibroblasts were grown to 90% confluence. Cultured cells were lifted using TrypLE and replated prior to treatment in antibiotic-free media (DMEMF12 and 10% FBS). The cells were allowed to adhere, culture media was then removed and replaced with 0.1% bovine serum albumin (BSA) media for one hour. Wells were randomly assigned to treatment versus vehicle-control conditions. Aliquots of JUN overexpression virus (prepared as above) were thawed just prior to use and applied to treatment wells with polybrene (Sigma–Aldrich, 1:1000). After 6-h incubation, the media was changed, and antibiotic-free media plus dox (2 ug/ml) was applied. Hygromycin selection was pursued at 48 h. Selection was confirmed using flow cytometry.

### Statistics and reproducibility

Statistical analyses were performed using the software GraphPad Prism v.6 (unless otherwise noted). Results are expressed as absolute numbers, percentages, fractions, or mean ± standard deviation (SD, unless otherwise noted). Unpaired *t*-test assuming two-tailed distribution or one-way analysis of variance (ANOVA) and post-hoc Tukey correction were used to compare groups where relevant. *P* < 0.05 was considered statistically significant. All experiments were replicated at least three times with similar results (unless otherwise noted).

### Reporting summary

Further information on research design is available in the Nature Research Reporting Summary linked to this article.

## Supplementary information

Supplementary Information

Description of Additional Supplementary Files

Supplementary Data 1

Supplementary Data 2

Reporting Summary

## Data Availability

Data to support the conclusions drawn in this manuscript can be found in the primary and supplemental figures. All RNA-seq data discussed in this publication have been deposited in NCBI’s Gene Expression Omnibus (GEO)^50^ and are accessible through GEO Series accession number GSE GSE153929. Source data are provided with this paper.

## Source Data

Source Data
