# Supplementary material for: Elucidating the fundamental fibrotic processes driving abdominal adhesion formation
Source: Nat Commun. 2020 Aug 13;11:4061. doi: 10.1038/s41467-020-17883-1 (PMC7426428; doi:10.1038/s41467-020-17883-1)
Supplement: Supplementary file 1 — Supplementary Information [file 41467_2020_17883_MOESM1_ESM.pdf]

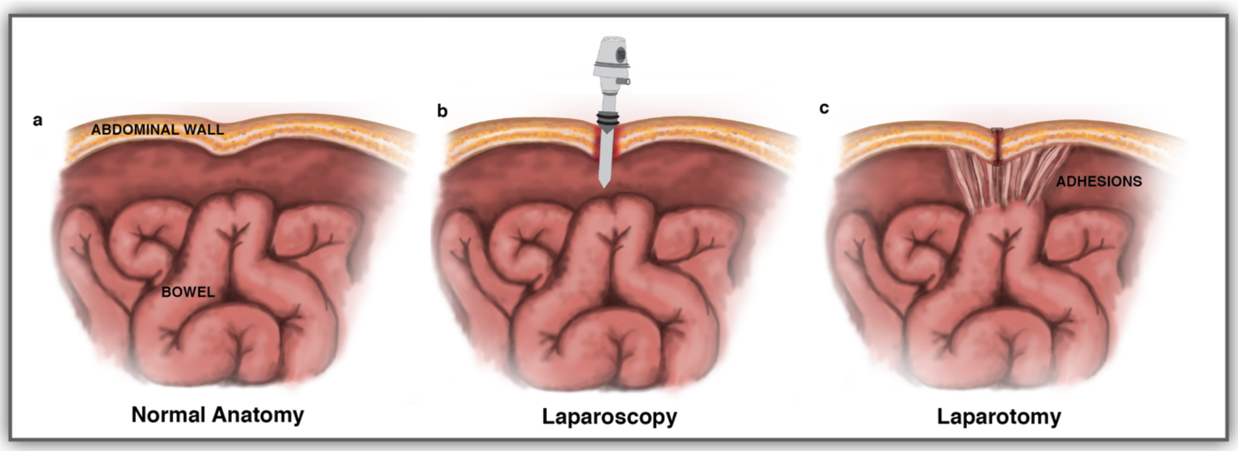

Supplementary Figure 1. Surgical schematic showing adhesions forming primarily after open laparotomy

a, Schematic of uninjured abdominal anatomy.

b, Schematic of laparoscopic abdominal surgical intervention; adhesions rarely form following isolated parietal peritoneal injury.

c, Schematic showing the abdomen following open laparotomy; abdominal adhesions have formed tethering a loop of bowel (visceral peritoneum) to the parietal peritoneum.

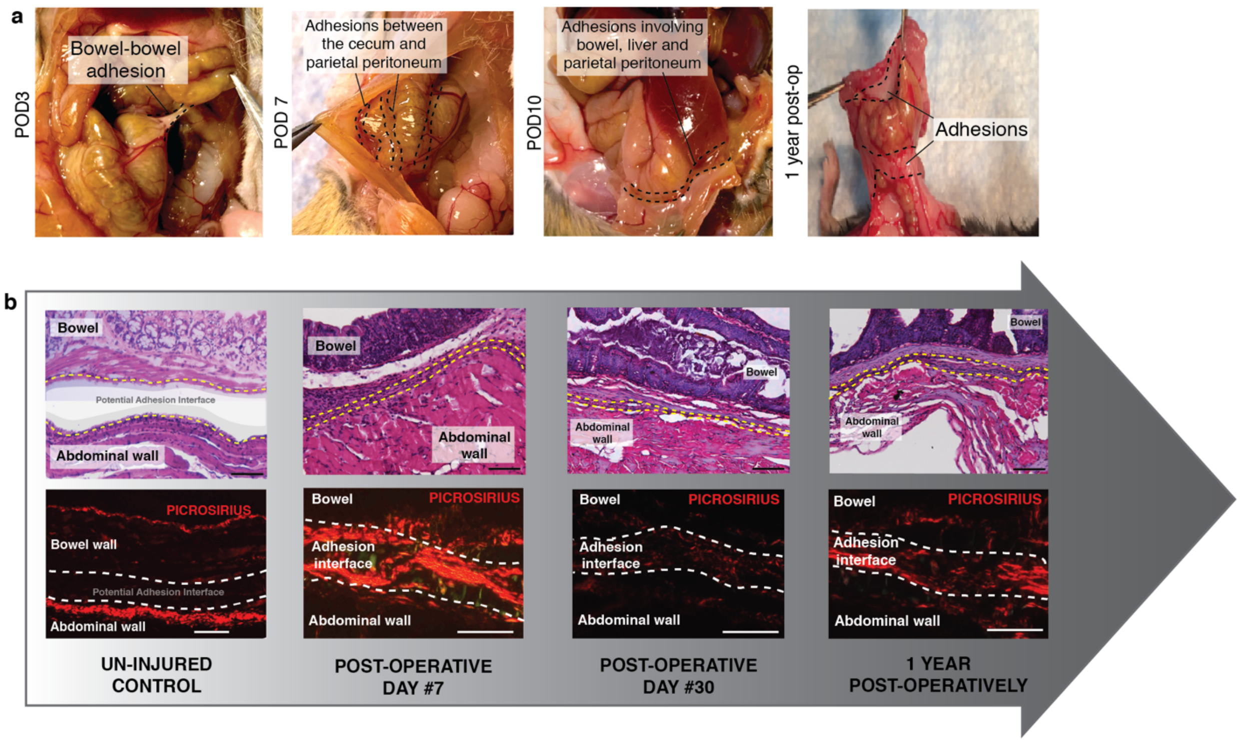

Supplementary Figure 2. Gross and histologic mouse abdominal adhesions
a, Photographs of representative samples illustrating adhesions in mice: bowel-bowel adhesion at POD 3 (**far left**), parietal to visceral peritoneal adhesion at POD 7 (**middle-left**), adhesions involving the liver (**middle-right**), and persistence of adhesions at one year post-operatively (**far right**). Adhesion interfaces outlined with black dotted lines, POD = post-operative day.
b, Time course of mouse adhesion formation using H&E (**top panels**) and picrosirius red (**bottom panels**) staining: Images of representative samples, uninjured control tissue from healthy litter mates (potential adhesion interface indicated, **far left**), POD 7 (**middle-left**), POD 30 (**middle-right**), 1 year post-operatively (**far right**). Stains as labelled in figure, adhesion interfaces outlined with dotted lines. Black scale bars, 100μm; white scale bars, 50μm. *n* > 5 biological replicates per timepoint.

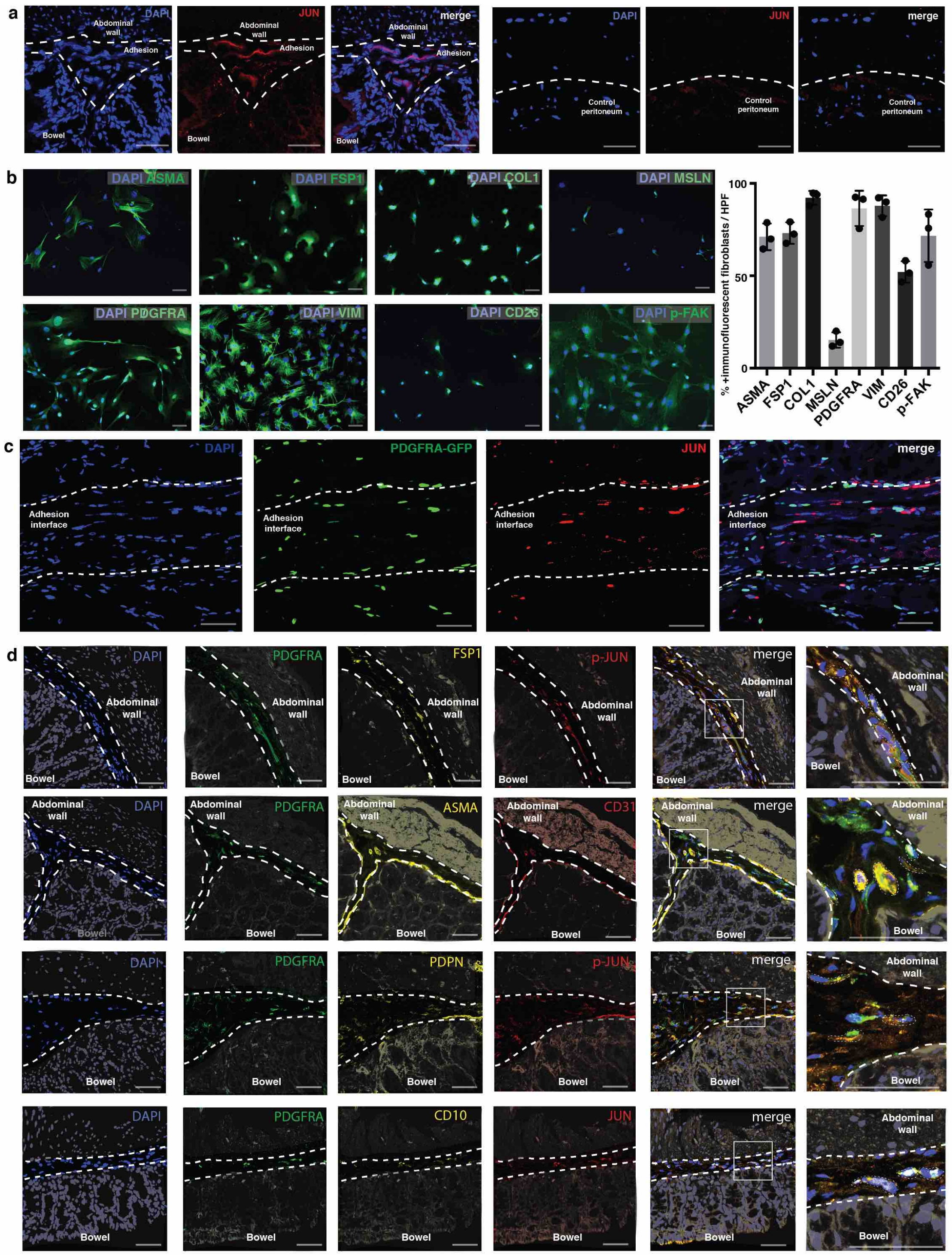

Supplementary Figure 3. Characterization of mouse abdominal adhesion fibroblast markers

a, Representative sample showing immunofluorescent staining for JUN in wild-type mouse adhesions (**left panels**) compared with control (uninjured) peritoneum from healthy litter mates (**right panels**). Individual channels, merge and structures as labelled in figure, white dotted lines highlight adhesion interface in left panel and edge of peritoneum in right panel. Scale bars, 50μm. *n* > 5 biological replicates per condition.

b, Representative images of immunocytochemistry screening of unbiased, FACS-isolated abdominal adhesion fibroblasts from wild-type mouse adhesions using ASMA, PDGFRA, FSP1, Vimentin (VIM), COL1, CD26, MSLN, and phospho-FAK. Quantitation at far right – shows % of +cells per marker per high power field (HPF). Scale bars, 100μm. *n* = 3 biological replicates.
c, Immunofluorescent staining for phospho (p)-JUN in a representative $PDGFRA^{GFP}$ adhesion tissue sample. Individual channels, merge and structures as labelled in figure, white dotted lines highlight adhesion interface. Scale bars, 50μm. *n* = 5 biological replicates.
d, Immunofluorescent staining of representative wild-type mouse abdominal adhesion tissue samples for fibrosis-relevant markers. Adhesion interfaces outlined with thick white dotted lines, antibody markers indicated at top right of images, individual channels with merge at right, zoom at far right, co-expressing cells indicated with thin white dotted lines where identified. Scale bars, 50μm. *n* = 3 biological replicates per condition.
Data and error bars represent means ± SD. Source data are provided as a Source Data file.

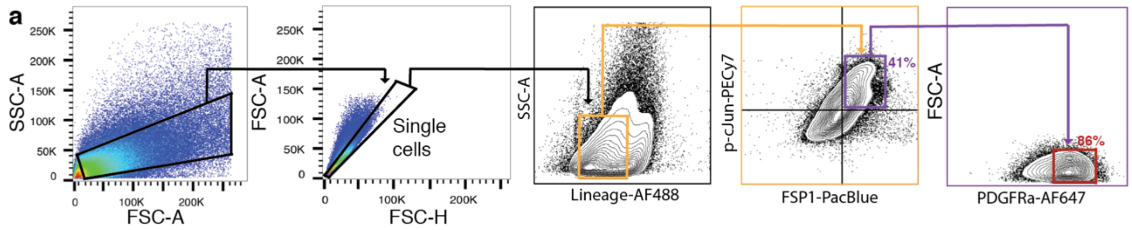

Supplementary Figure 4. Phospho-JUN, FSP1, and PDGFRA expression in mouse adhesion fibroblasts

a, Representative flow cytometry plots showing analysis of mouse adhesion fibroblasts for expression of phospho-JUN, FSP1 and PDGFRA. Percentages of cells noted in corresponding colors in figure.

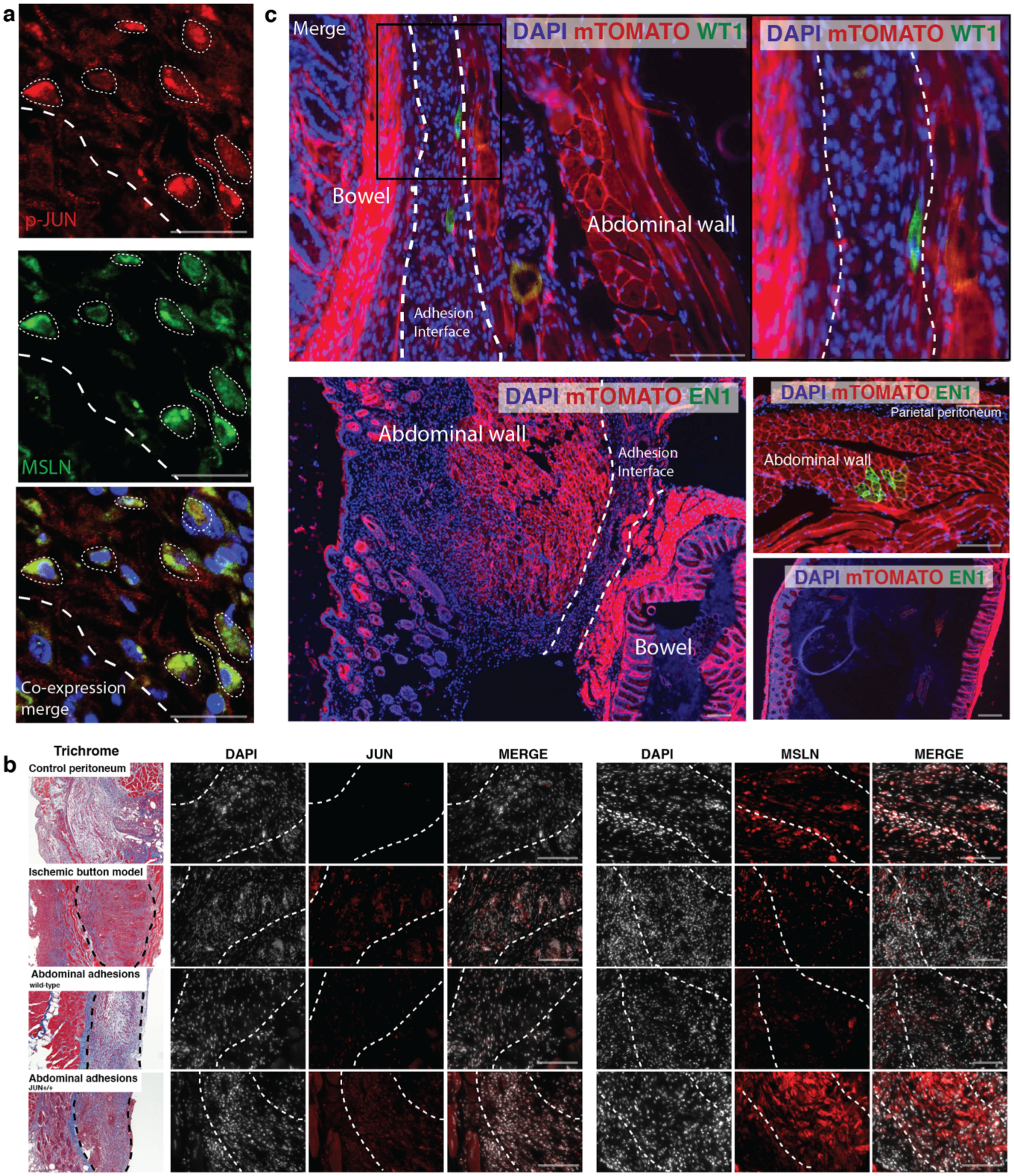

Supplementary Figure 5. MSLN, WT1 and EN1 expression in mouse adhesions

a, Images of representative mouse adhesion tissue with immunofluorescent staining for phospho-JUN and MSLN. Adhesion interfaces outlined with thick white dotted lines, antibody markers indicated at bottom left of images, co-expressing cells highlighted with thin white dotted lines. Scale bars, 25μm. *n* = 3 biological replicates.

b, Images of representative ischemic button peritoneal fibrosis mouse model and abdominal adhesion tissue with immunofluorescent staining for JUN (**left panels**) and MSLN (**right panels**) with corresponding trichrome staining (**far left panels**). Black dotted lines indicate adhesion interface in trichrome images, white dotted lines indicate adhesion interface in immunofluorescent

images. Conditions labelled at left and above images. Scale bars indicated on merge images, 100μm. *n* = 3 biological replicates.

c, Fluorescent imaging data from representative samples from WT1^{Cre}::ROSA26^{mTmG} mice shows that WT1-expressing fibroblasts (green) very rarely contribute to adhesion formation (**top panels,** zoom of box as indicated on adhesion interface at right. EN1-lineage fibroblasts appear minimally present in the parietal peritoneum (**middle right panel**), are not found in the bowel wall (**bottom right panel**), and do not contribute to adhesions (**bottom left panel**). White dotted lines indicate adhesion interface, structures as labeled on figure. Scale bars, 100μm. *n* = 3 biological replicates.

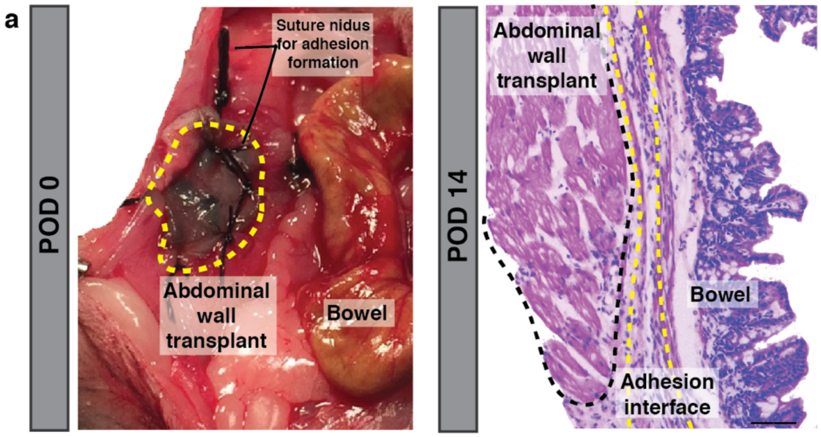

Supplementary Figure 6. Abdominal wall transplant model

a, Representative gross (**left panel,** yellow dotted lines outline abdominal wall transplant) and histologic (**right panel**, H&E, yellow dotted line outlines adhesion interface) images of abdominal wall transplant model. Structures as labelled in figure. *n* = 5 biological replicates. Scale bar, 50μm.

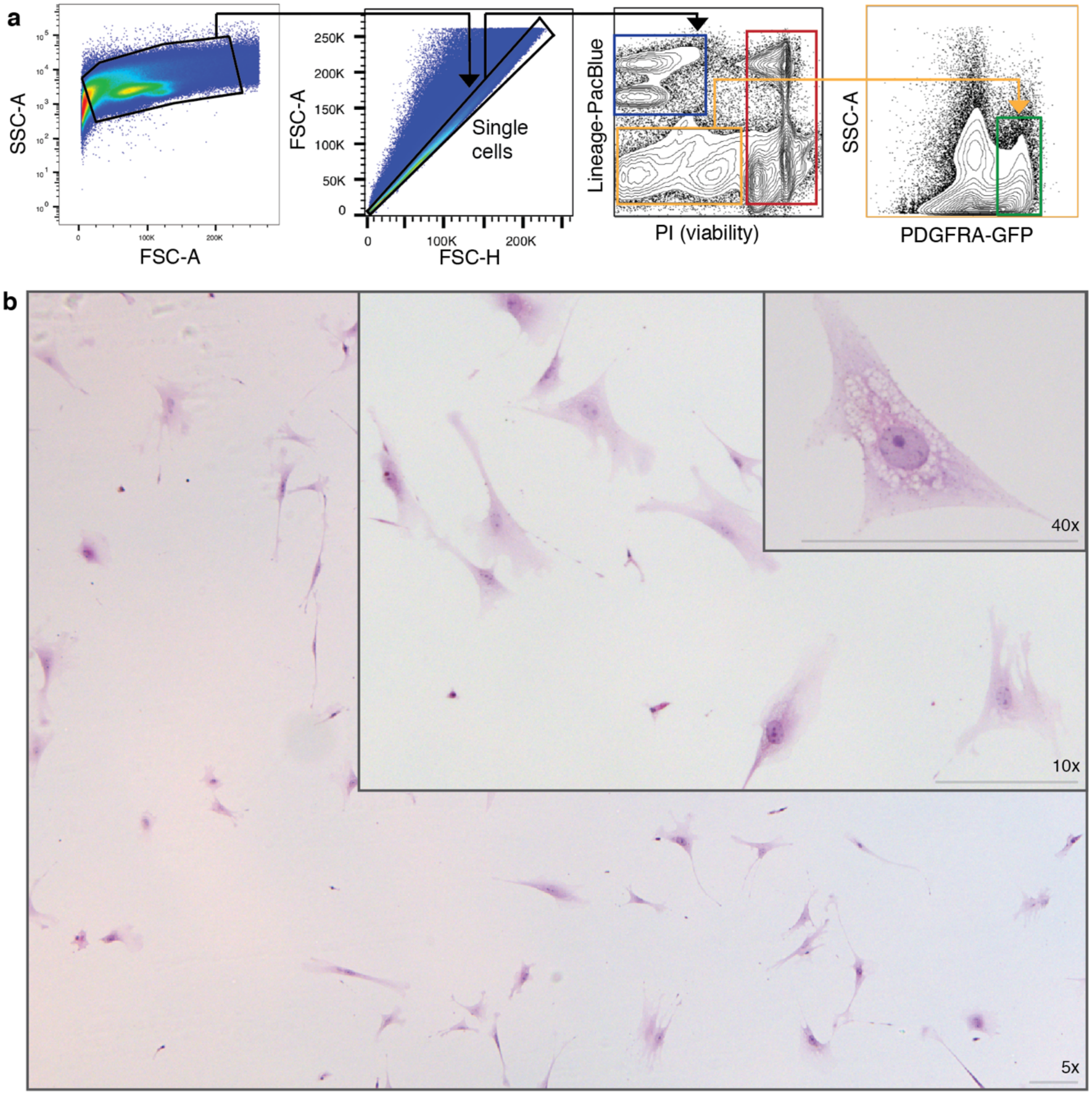

Supplementary Figure 7. Mouse adhesion fibroblast isolation

a, FACS isolation strategy for mouse abdominal adhesion fibroblasts, example of representative gating.

b, Eosin stained FACS-isolated mouse adhesion fibroblasts. Zoom noted at right bottom of images. Scale bars, 25μm.

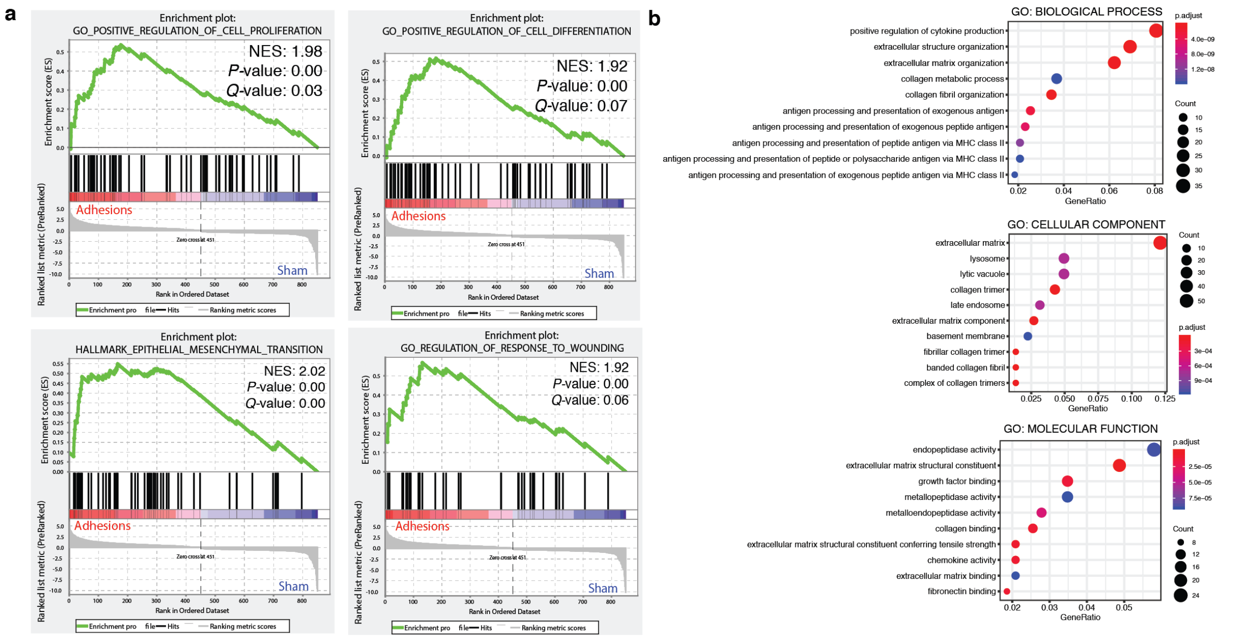

Supplementary Figure 8. Mouse adhesion fibroblast RNA-seq GSEA and GO analysis

a, Gene Set Enrichment Analysis (GSEA) of mouse adhesion fibroblast bulk RNA-seq data shows enrichment of ‘Cell Proliferation’, ‘Cell Differentiation’, ‘Epithelial Mesenchymal Transition (EMT)’ and ‘Regulation of Response to Wounding’ pathways. NES, *P* and Q values noted in figure panels. Normalized enrichment score is calculated using the Broad Institute GSEA software, which considers NES = actual ES (enrichment score) / *mean*(ES against all permutations of the dataset). Geneset considered significantly enriched if NES has an FDR q-value below 0.25. *P* value represents the nominal *P* value of the ES. *Q* value is *P* value adjusted for multiple hypothesis testing using the original GSEA approach based on comparing the tails of the observed and null distributions for the NES.[42]

b, Gene Ontology (GO) term analysis for mouse RNA-seq dataset. Terms and statistics as noted in figure panels.

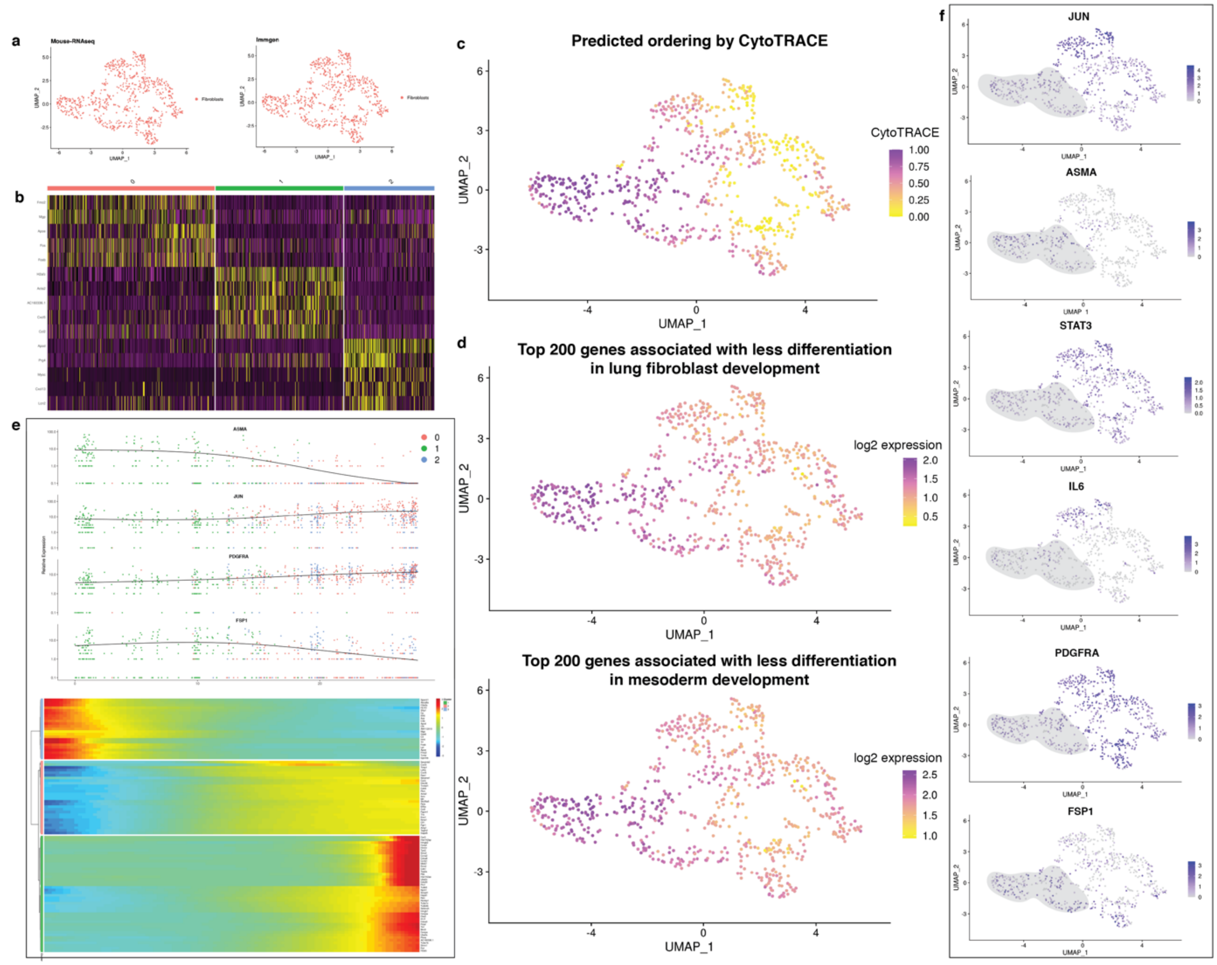

Supplementary Figure 9. Mouse adhesion fibroblast scRNA-seq

a, Mouse-RNAseq and Immgen objective analyses identify cell types (fibroblasts) represented in mouse adhesion fibroblast scRNA-seq data. Cell types as labelled by colors.

b, Heatmap showing differential gene expression patterns based on clusters (**Fig. 4d**). Colors and numbers represent clusters along top of panel, most differentially expressed genes labelled at left.

c, Predicted ordering of single cells by CytoTRACE in our mouse scRNA-seq data.

d, Visualization of the gene counts signature, or top 200 genes, from two independent datasets, lung fibroblast (**top panel**) and mesoderm development (**bottom panel**), in our mouse scRNA-seq data.

e, Pseudotime (Monocle 2) trajectories plot for mouse scRNA-seq data (**top panel,** for the following genes, from top to bottom: *ASMA*, *JUN*, *PDGFRA*, *FSP1*, colors indicate clusters from **Fig. 4d**) and heatmap (**bottom panel,** clusters from **Fig. 4d** noted on left, most significant genes displayed along right axis).

f, Mouse scRNA-seq data UMAP feature plots for the following genes, from top to bottom and right to left: *JUN*, *ASMA*, *STAT3*, *PDGFRA*, *IL6*, *FSP1*. Cluster 1 highlighted in grey.

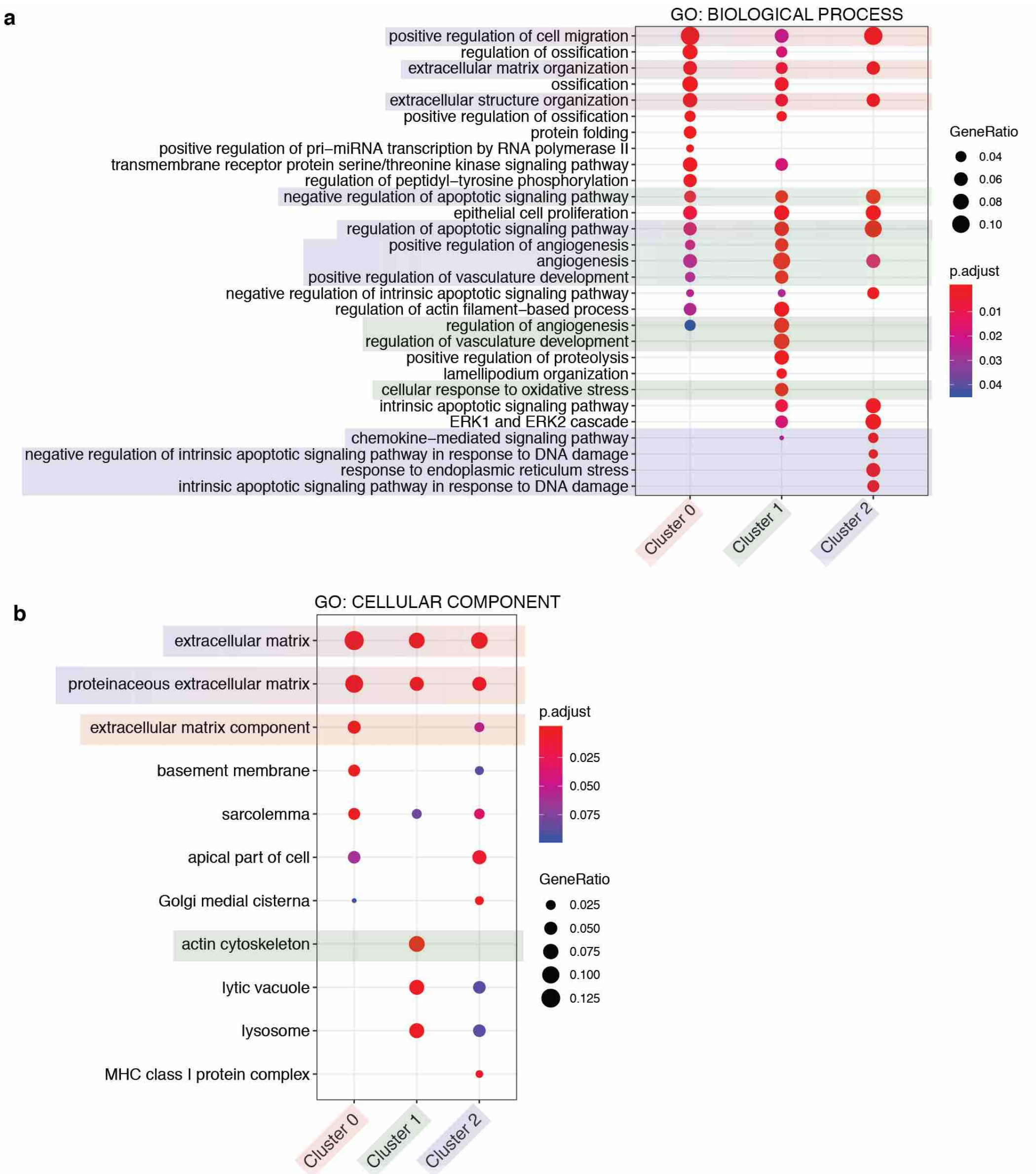

Supplementary Figure 10. Mouse adhesion fibroblast scRNA-seq GO analysis

a-b, GO term analysis for mouse scRNA-seq dataset. Terms and statistics as noted in figure panels. Colors used to highlight specific processes and components correlate with cluster colors used in **Fig. 4d.**

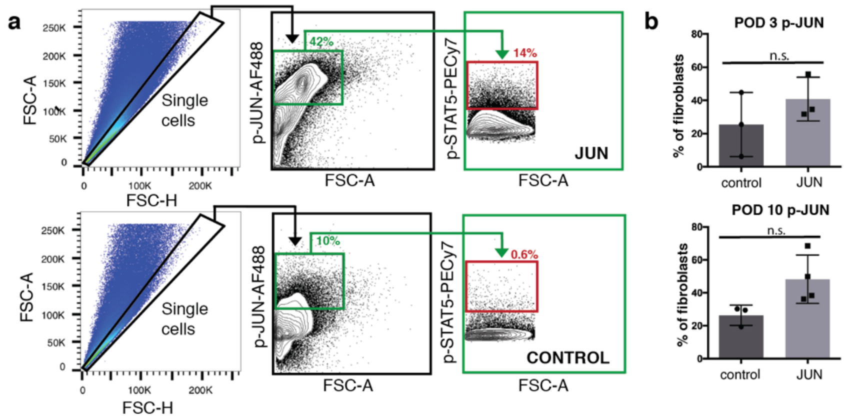

Supplementary Figure 11. JUN expression in mouse adhesions

a, Representative phospho-flow cytometry analysis for phospho(p)-JUN and p-STAT5 expression in abdominal adhesion fibroblasts isolated from JUN mice induced locally with doxycycline at the time of adhesion formation (**top panel**) compared with vehicle control (**bottom panel**) at 24 hours after surgery. Quantification in **Fig. 5**.

b, Quantification of phospho-JUN upregulation in abdominal adhesion tissue isolated from JUN mice induced locally with doxycycline at the time of adhesion formation compared with vehicle control, at POD 3 and POD 10. n.s. = not significant. n = 3 biological replicates per condition per timepoint.

Data and error bars represent means ± SD. Unpaired two-tailed t test. Source data are provided as a Source Data file.

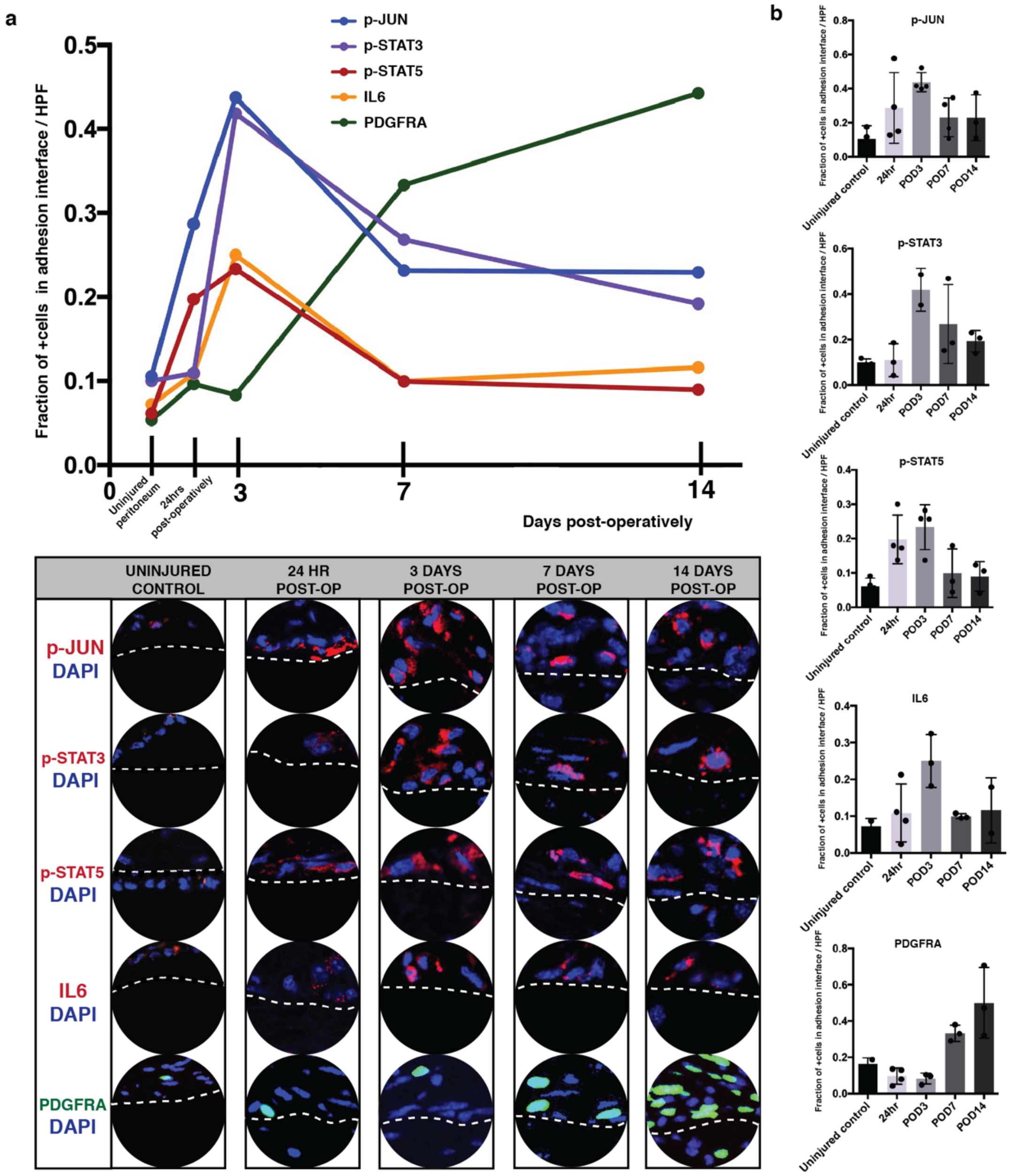

Supplementary Figure 12. Mouse abdominal adhesion tissues express JUN early and related pathways are activated

a, Quantitation (**top panel,** individual data points represent means) and representative immunofluorescent images (**bottom panel**) of tissue expression of p-JUN and related proteins (p-STAT3, p-STAT5, IL6, PDGFRA) in mouse abdominal adhesions at 24 hours, 3, 7, and 14 days post-operatively (post-op), compared with uninjured peritoneum control (left most panels). All protein expression in terms of immunofluorescent staining aside from PDGFRA, which is measured using the PDGFRA^{GFP} mouse model.

b, Individual datapoints for data summarized in **a**, top panel. $n \geq 3$ biological replicates assessed per condition per timepoint.

Data and error bars represent means ± SD. Source data are provided as a Source Data file.

a

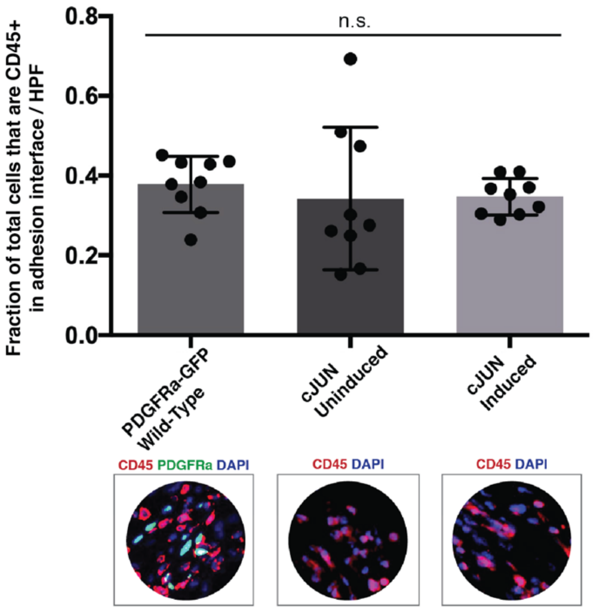

Supplementary Figure 13. JUN expression does not affect the number of CD45+ cells in the abdominal adhesion interface

a, Quantitation of CD45 expression in abdominal adhesion tissue samples from $PDGFRA^{GFP}$ (wild-type) mice (**left panels**), JUN mice – uninduced (**middle panels**), JUN mice – induced with doxycycline at the time of adhesion surgery (**right panels**). *n* = 3 separate adhesions examined from each of 3 mice per condition.

Data and error bars represent means ± SD. n.s. = not significant. One way Anova. Source data are provided as a Source Data file.

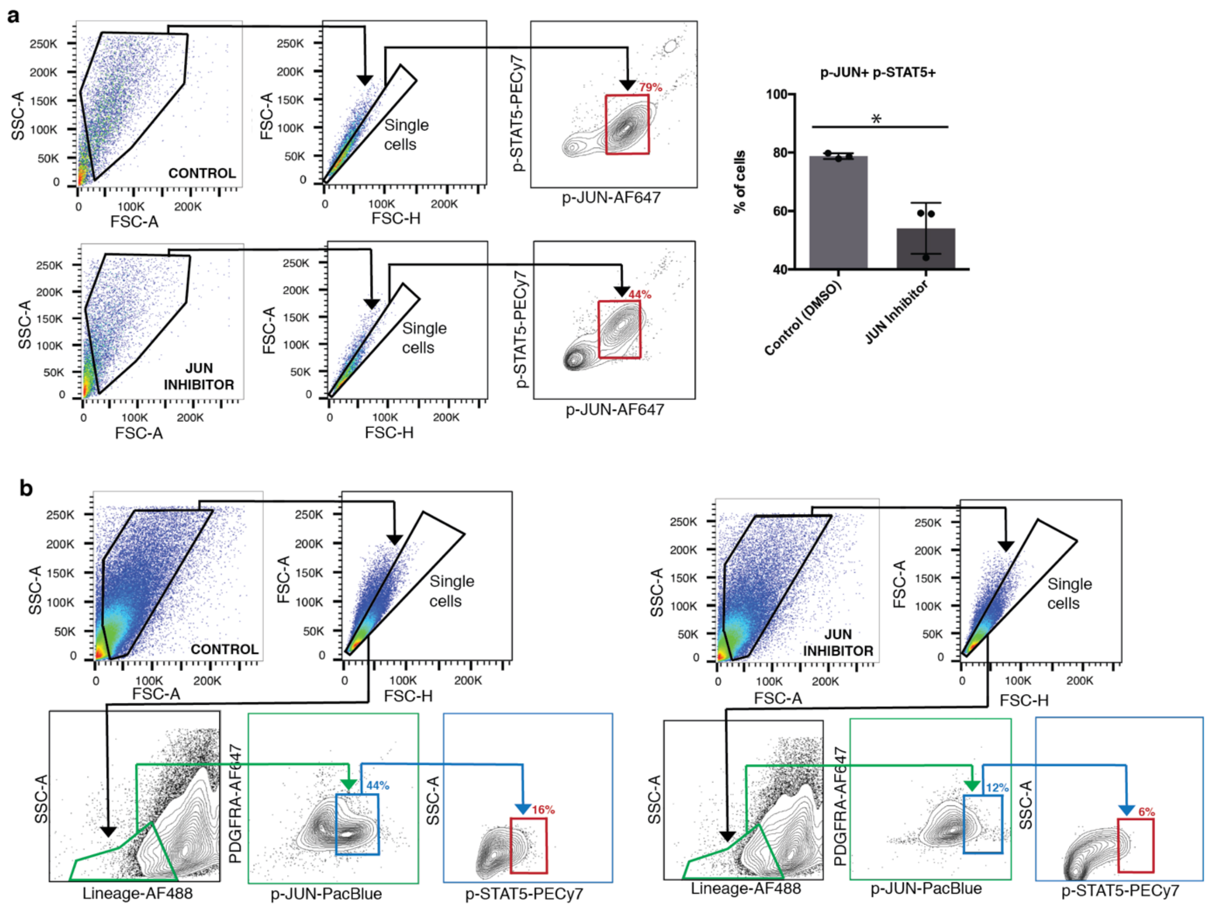

Supplementary Figure 14. T-5224 suppresses JUN and STAT5 expression in mouse fibroblasts *in vitro* and *in vivo*

a, Representative phospho-flow cytometry analysis for phospho-JUN and phospho-STAT5 expression in mouse adhesion fibroblasts treated with JUN inhibitor *in vitro* versus vehicle control, quantitation (**right panel**). n = 3 biological replicates

b, Representative phospho-flow cytometry analysis for phospho-JUN and phospho-STAT5 expression in JUN mouse adhesions treated locally with JUN inhibitor *in vivo* versus vehicle control. Quantitation in **Fig. 5**.

Data and error bars represent means ± SD. *P = 0.008, unpaired two-tailed t test. Source data are provided as a Source Data file.

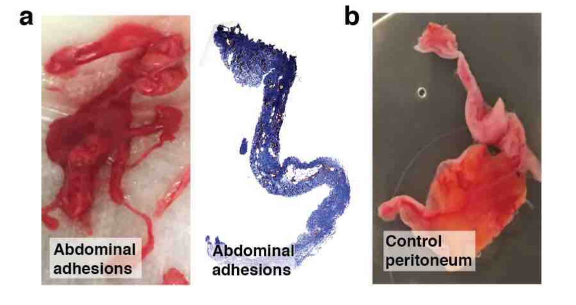

Supplementary Figure 15. Images of human abdominal adhesions specimens
a, Gross image of human abdominal adhesion tissue (**left panel**), stitched histologic image of human abdominal adhesion tissue stained with trichrome (**right panel**). *n* = 24.
b, Gross image of control human peritoneum tissue. *n* = 10.

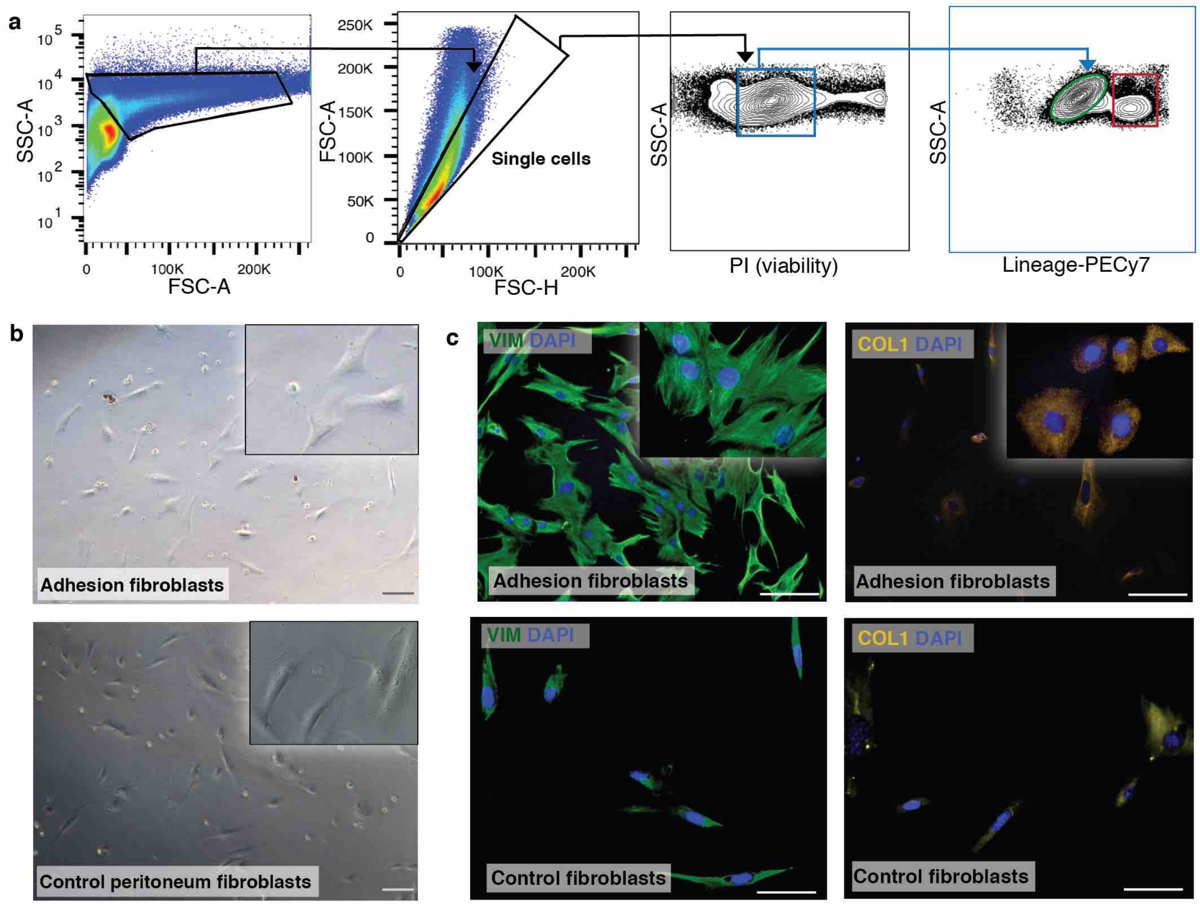

Supplementary Figure 16. Isolation of human abdominal adhesion fibroblasts

a, Representative plots illustrating unbiased FACS isolation strategy for sorting human abdominal adhesion fibroblasts.

b, Representative *in vitro* imaging of FACS-isolated human fibroblasts from abdominal adhesions (*n* = 24 unique patient specimens obtained, **left panel**) and control peritoneum (*n* = 10 unique patient specimens obtained, **right panel**). Insets, zooms at right top; scale bars, 25μm.

c, Representative FACS-isolated human abdominal adhesion fibroblasts (**top panels**) and human control peritoneal fibroblasts (**bottom panels**) staining for vimentin (VIM) and Collagen 1 (COL1) on immunocytochemistry. *n* = 3 biological replicates per condition. Insets, zooms at right top; scale bars, 25μm.

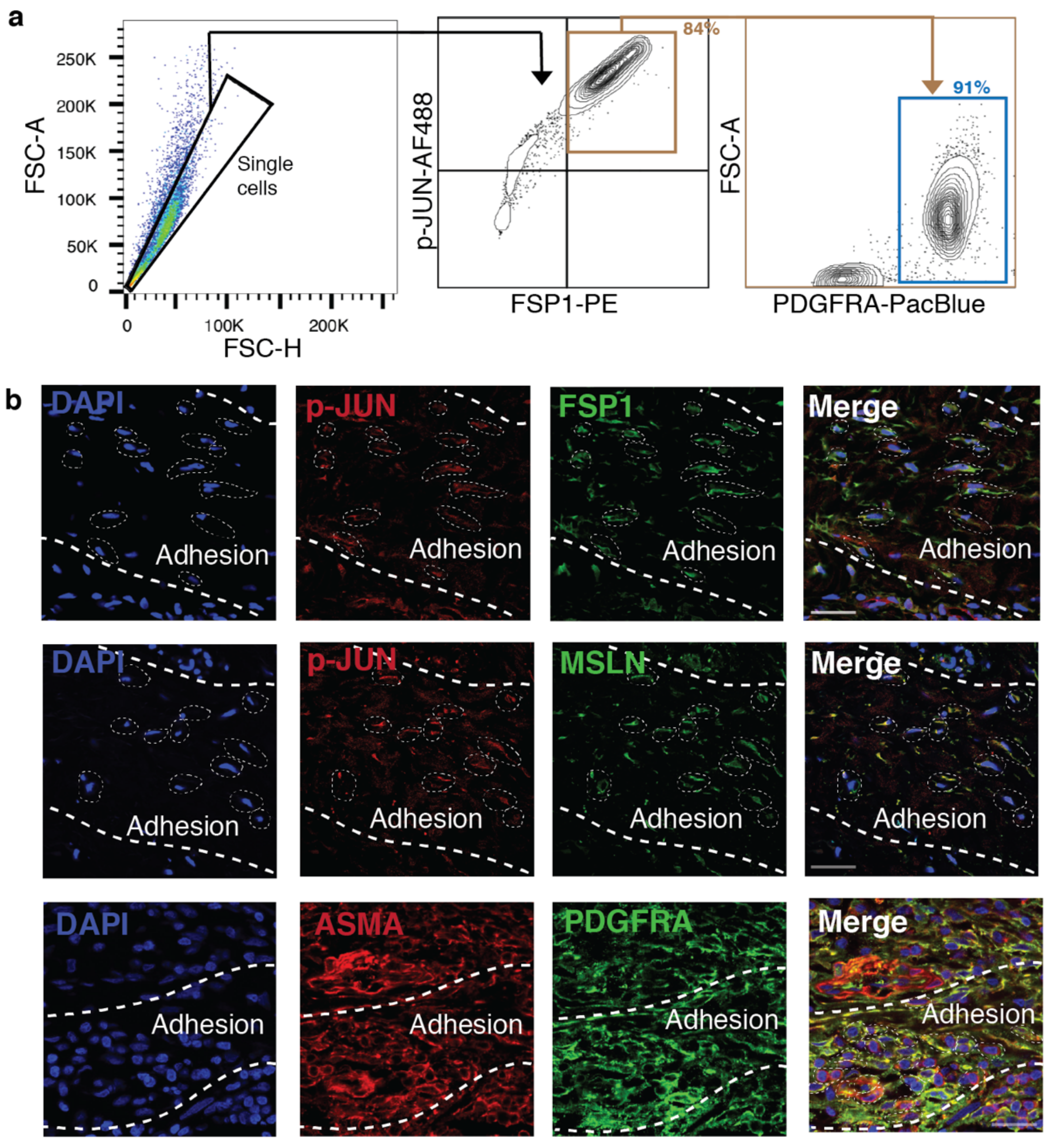

Supplementary Figure 17. Characterization of human abdominal adhesion fibroblast markers

a, Representative plots showing unbiased phospho-flow cytometry analysis of human adhesion fibroblasts for expression of phospho(p)-JUN, FSP1, and PDGFRA (percentages of cells noted in corresponding color next to gates).

b, Immunofluorescent staining of human abdominal adhesion tissue for phospho-JUN and FSP1 (**top panels**), phospho-JUN and MSLN (**middle panels**), ASMA and PDGFRA (**bottom panels**). Adhesion tissue outlined with thick white lines, co-expressing cells highlighted with thin white lines, antibodies noted in top left corner of each panel, merge panels at far right. *n* = 3 biological replicates per condition. Scale bars, 25μm.

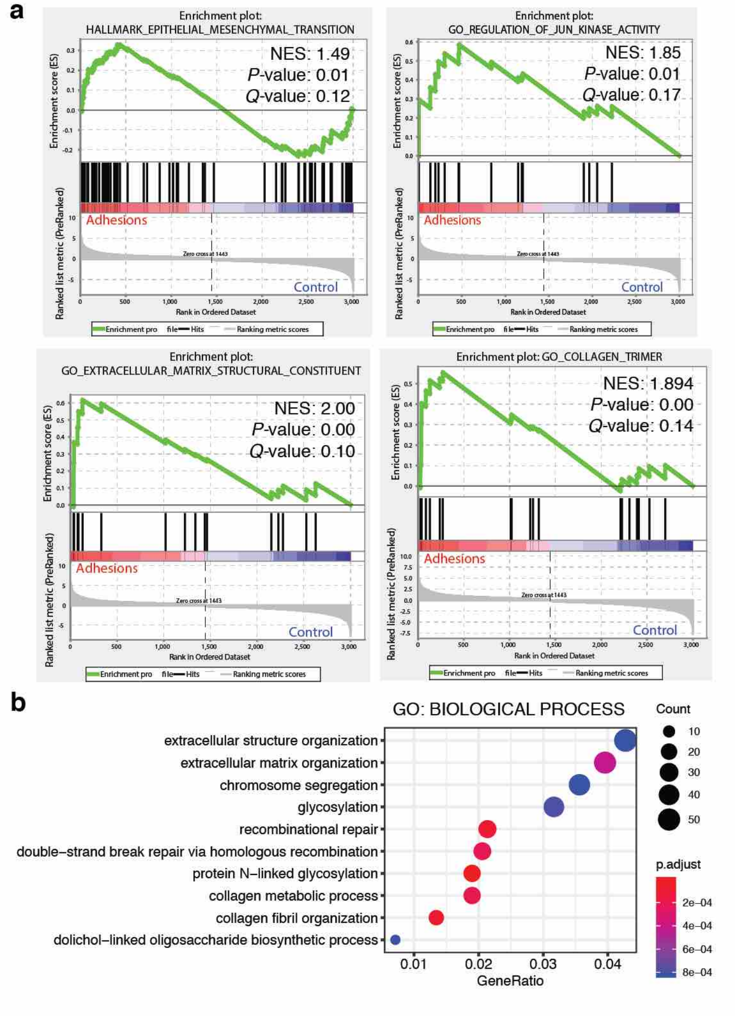

Supplementary Figure 18. Human adhesion fibroblast RNA-seq GSEA and GO analysis
a, GSEA of human adhesion fibroblast bulk RNA-seq data shows enrichment of 'EMT', '*JUN* Kinase Activity', 'Extracellular Matrix Structural Constituent' and 'Collagen Trimer' pathways. NES, P and Q values noted in figure panels (See **Supplementary Figure 8** for additional details).
b, Gene Ontology (GO) term analysis for human bulk RNA-seq dataset. Terms and statistics as noted in figure panels.

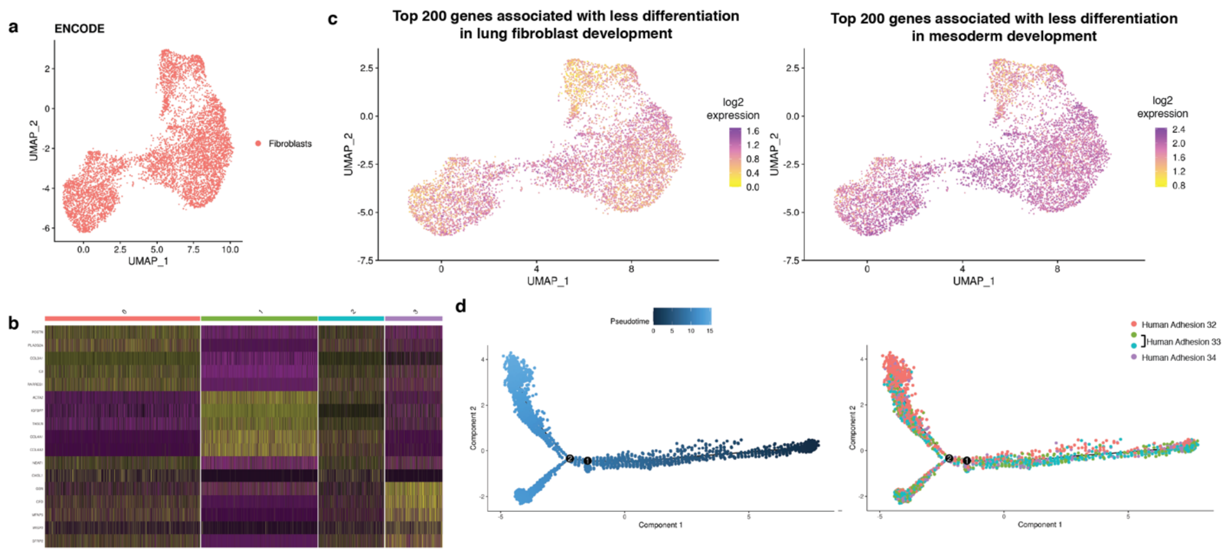

Supplementary Figure 19. Human adhesion fibroblast scRNA-seq

a, ENCODE objective analysis to identify cell types (fibroblasts) represented in human adhesion fibroblast scRNA-seq data (**Fig. 8a**). Cell types as labelled by colors.

b, Heatmap showing differential gene expression patterns based on clusters (**Fig. 8a**). Colors and numbers represent clusters along top of panel, most differentially expressed genes labelled at left.

c, Visualization of the gene counts signature, or top 200 genes, from two independent datasets, lung fibroblast (**left panel**) and mesoderm development (**right panel**), in human scRNA-seq data.

d, Pseudotime plots for human scRNA-seq data – pseudotime (**left panel)** and clusters (from **Fig. 8a**, **right panel**).

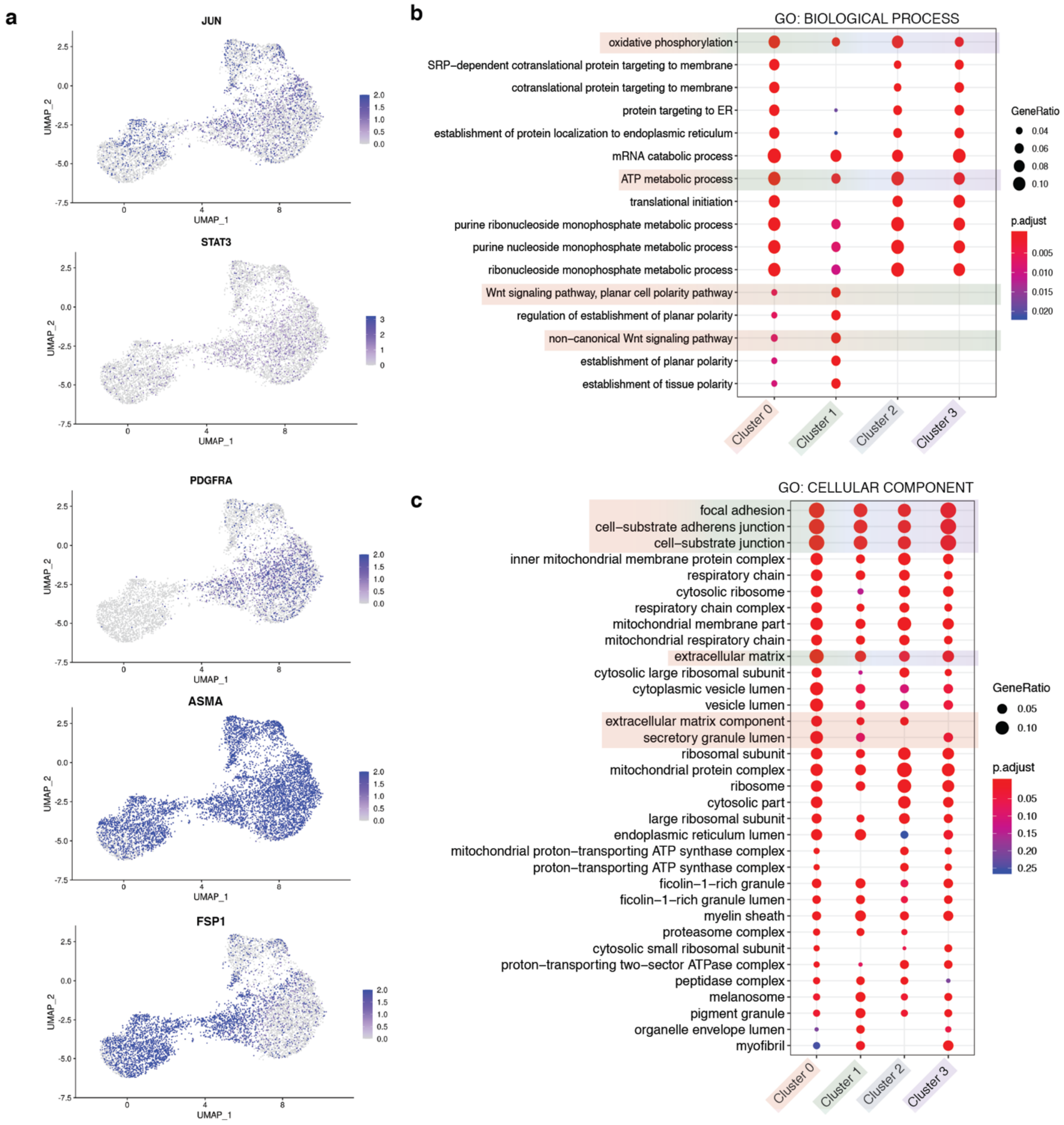

Supplementary Figure 20. Human adhesion fibroblast scRNA-seq feature plots and GO analysis

a, Human scRNA-seq data UMAP feature plots for the following genes, from top to bottom: *JUN*, *ASMA*, *STAT3*, *PDGFRA*, and *FSP1*.

b-c, GO term analysis for our human scRNA-seq dataset. Terms and statistics as noted in figure panels. Colors used to highlight specific processes and components correlate with cluster colors used in **Fig. 8a.**

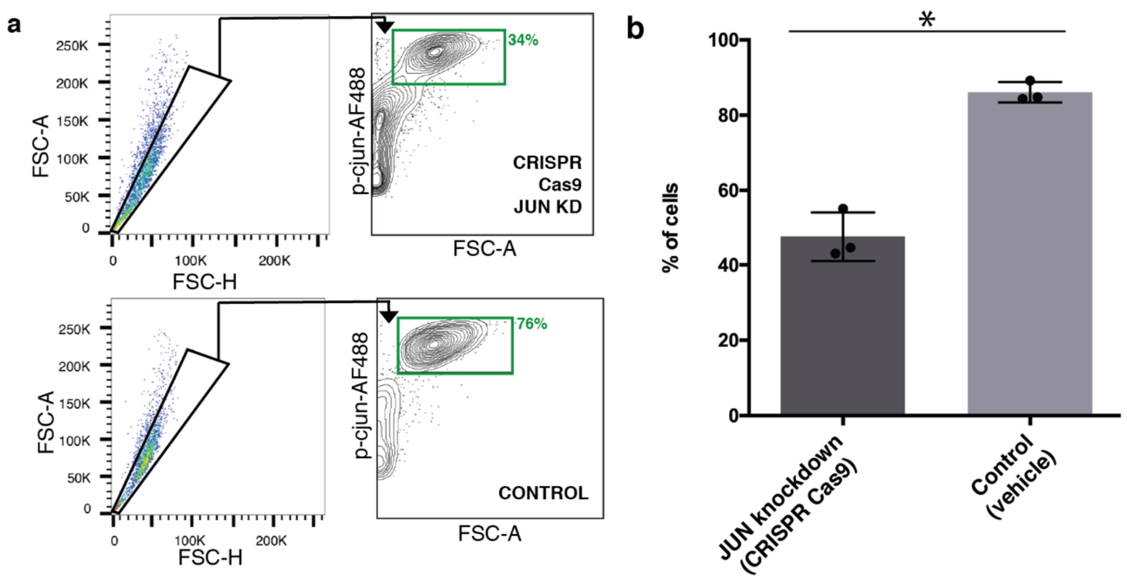

Supplementary Figure 21. *JUN* knockdown in human adhesion fibroblasts
a, Representative phospho-flow cytometry analysis of human abdominal adhesion cells shows knock down of phospho-JUN expression with CRISPR Cas9 (**top panels**) compared with vehicle-control abdominal adhesion cells (**bottom panels**). Conditions as noted in figure, percentages of cells noted in corresponding color next to gates.
b, Quantitation of phospho-flow cytometry analysis of phospho-JUN expression in human adhesion fibroblast samples treated with CRISPR Cas9 to knock-down *JUN* or vehicle control. n = 3 biological replicates, data points represent average of technical replicates.
Data and error bars represent means ± SD. *P = 0.0001, unpaired two-tailed t test. Source data are provided as a Source Data file.

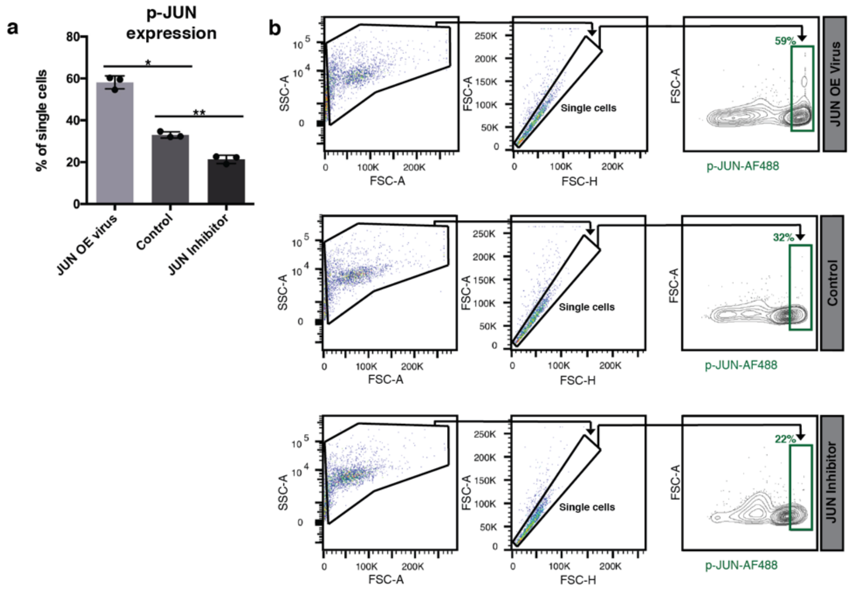

Supplementary Figure 22. Fibroblast phospho-flow cytometry comparison *JUN* overexpression, control, and JUN inhibitor

a, Quantitation of phospho-flow cytometry analysis showing % p-JUN+ of total single cells of NIH 3T3 cells treated with the following conditions: virally-mediated *JUN* overexpression, vehicle control, and JUN inhibitor. n = 3 replicates.

b, Representative phospho-flow cytometry plots for data quantitated in **a.** Conditions as labelled in figure panel.

Data and error bars represent means ± SD. *P = 0.0002, **P = 0.001, unpaired two-tailed t test. Source data are provided as a Source Data file.

Supplementary Table 1. Human adhesions and control specimens – all

Specimen type	Number female	Number male	Months after prior surgery
Adhesion	9	15	Mean 28 (Range 2-344) months
Control	5	5	N/A

Supplementary Table 2. Human adhesions and control specimens used for bulk RNA-seq

Specimen type	Number female	Number male	Months after prior surgery
Adhesion	2	4	Mean 19 (Range 3-65) months
Control	2	1	N/A

Supplementary Table 3. Human adhesions specimens used for scRNA-seq

Specimen type	Number female	Number male	Months after prior surgery
Adhesion	2	1	Mean 13 (Range 9-19) months

Supplementary Table 4. Primer sequence for qRT-PCR

Transcript	Forward Primer Sequence (5'-3')	Reverse Primer Sequence (5'-3')
hACTB	ACAGAGCCTCGCCTTTG	CCTTGCACATGCCGGAG
hJUN	GACGGACTGTTCTATGACTGC	TGCTGTGTTTCAGGATCTTGG
hSTAT3	GAGGCATTCGGAAAGTATTGTC	CATCGGCAGGTCAATGGTAT
hSTAT5a	AACAGAGGCTGGTCCGA	CTGGTTGATCTGAAGGTGCT
hSPP1	CCCCACAGTAGACACATATGATG	TTCAACTCCTCGCTTTCCAT
mActB	GATTACTGCTCTGGCTCCTAG	GACTCATCGTACTCCTGCTTG
mVim	CGTCCACACGCACCTACAG	GGGGGATGAGGAATAGAGGCT
mCOL1a	GCCAAGAAGACATCCCTGAAG	TGTGGCAGATACAGATCAAGC

h = human, m = mouse

Supplementary Table 5. sgRNA sequences for CRISPR-mediated genome engineering

**Transcript**	**Primer Sequence (5'-3')**
CJUN sgRNA_1 F	CACCGTGAACCTGGCCGACCCAGTG
CJUN sgRNA_1 R	AAACCACTGGGTCGGCCAGGTTCAC
CJUN sgRNA_2 F	CACCGCCGTCCGAGAGCGGACCTTA
CJUN sgRNA_2 R	AAACTAAGGTCCGCTCTCGGACGGC
