# Supplementary material for: Elucidating the fundamental fibrotic processes driving abdominal adhesion formation
Source: Nat Commun. 2020 Aug 13;11:4061. doi: 10.1038/s41467-020-17883-1 (PMC7426428; doi:10.1038/s41467-020-17883-1)
Supplement: Supplementary file 2 — Description of Additional Supplementary Files [file 41467_2020_17883_MOESM2_ESM.docx]

Description of Additional Supplementary Files

Title: Supplementary Dataset 1.

Description: Genes differentially expressed between mouse fibroblasts isolated from abdominal adhesions ('Adhesions') versus sham-surgery controls ('Sham') by DESeq2 Table of differentially expressed genes between mouse fibroblasts isolated from abdominal adhesions (n = 4 samples) and sham controls (n = 4 samples) after performing lfcShrink using Approximate Posterior Estimation for GLM (apeglm) in DESeq2. A significance threshold of Padjusted < 0.1 was used to filter genes. Positive log2FoldChange values correspond to genes significantly enriched in abdominal adhesions compared to sham controls.

Title: Supplementary Dataset 2:

Description: Genes differentially expressed between human fibroblasts isolated from abdominal adhesions ('Adhesions') versus control peritoneum tissue ('Healthy') by DESeq2 Table of differentially expressed genes between human fibroblasts isolated from abdominal adhesions (n = 6 samples) and control peritoneal tissue (n = 3 samples) after performing lfcShrink using Approximate Posterior Estimation for GLM (apeglm) in DESeq2. A significance threshold of P-adjusted < 0.1 was used to filter genes. Positive log2FoldChange values correspond to genes significantly enriched in abdominal adhesions compared to healthy controls.
