# Supplementary material for: Elucidating the fundamental fibrotic processes driving abdominal adhesion formation
Source: Nat Commun. 2020 Aug 13;11:4061. doi: 10.1038/s41467-020-17883-1 (PMC7426428; doi:10.1038/s41467-020-17883-1)
Supplement: Supplementary file 3 — Supplementary Data 1 [file 41467_2020_17883_MOESM3_ESM.pdf]

Supplementary Dataset 1: Genes differentially expressed between mouse fibroblasts isolated from abdominal adhesions ('Adhesions') versus sham-surgery controls ('Sham') by DESeq2

This workbook contains one sheet:
Sheet 1: Table of differentially expressed genes between mouse fibroblasts isolated from abdominal adhesions (*n* = 4 samples) and sham controls (*n* = 4 samples) after performing lfcShrink using Approximate Posterior Estimation for GLM (apeglm) in DESeq2. A significance threshold of *P-adjusted* < 0.1 was used to filter genes. Positive log2FoldChange values correspond to genes significantly enriched in abdominal adhesions compared to sham controls.

GeneID	baseMean	log2FoldChange	lfcSE	pvalue	padj
Spp1	1096.48	5.83	0.48	6.35E-27	2.26E-23
Ms4a6d	180.65	4.97	0.79	4.59E-11	2.11E-08
Col24a1	220.05	4.65	0.46	3.08E-21	6.28E-18
Trem2	63.84	4.12	0.79	1.21E-07	2.50E-05
Cnn1	144.00	3.85	0.56	2.20E-11	1.14E-08
Scx	232.95	3.83	0.76	3.93E-07	6.37E-05
Cxcl5	674.03	3.76	0.57	2.33E-10	9.24E-08
Ptn	7790.27	3.59	0.27	2.03E-36	2.89E-32
Ctla2a	730.83	3.47	0.56	1.49E-09	4.72E-07
Ccl3	114.65	3.38	0.80	2.83E-06	3.11E-04
Hp	2166.06	3.20	0.35	5.06E-18	7.21E-15
Gpr176	367.92	3.16	0.35	6.21E-19	9.83E-16
Saa3	4844.89	2.97	0.80	3.15E-05	2.24E-03
Dlk1	2617.64	2.97	0.38	6.59E-16	7.22E-13
Zfp185	751.75	2.92	0.37	2.12E-14	1.89E-11
Slc7a2	288.05	2.91	0.74	1.56E-05	1.34E-03
Glipr1	221.19	2.81	0.42	2.25E-11	1.14E-08
Tagln	830.36	2.76	0.43	6.27E-11	2.79E-08
Ctxn1	166.28	2.75	0.48	3.15E-09	9.76E-07
Cthrc1	9438.80	2.74	0.28	3.71E-23	1.06E-19
Wisp1	1477.93	2.69	0.48	8.89E-09	2.35E-06
Adam12	2962.80	2.61	0.45	8.66E-10	2.94E-07
C1qc	1013.80	2.56	0.46	6.55E-09	1.82E-06
Csf1r	1007.45	2.47	0.38	3.45E-11	1.64E-08
Ms4a7	227.98	2.43	0.53	8.62E-07	1.18E-04
Marcksl1	2625.82	2.36	0.20	2.25E-32	1.60E-28
Pirb	243.05	2.33	0.70	7.00E-05	4.25E-03
C1qb	1360.11	2.30	0.49	3.37E-07	5.52E-05
Acta2	2575.47	2.28	0.44	2.19E-08	5.11E-06
Asic2	175.67	2.25	0.68	6.37E-05	3.94E-03
Fcgr3	425.60	2.22	0.54	2.66E-06	2.94E-04
Fbn2	1239.21	2.20	0.60	1.80E-05	1.49E-03
Tnc	7481.43	2.17	0.19	1.72E-29	8.18E-26
Adamts7	341.49	2.15	0.52	1.73E-06	2.07E-04
Cybb	379.65	2.14	0.54	3.25E-06	3.46E-04
Cdh11	4922.04	2.10	0.29	7.74E-14	6.48E-11
P4ha3	1068.74	2.09	0.52	3.66E-06	3.81E-04
Olfm2	242.47	2.09	0.55	1.07E-05	9.86E-04
Mdk	3235.70	2.08	0.27	5.02E-15	5.11E-12
Eln	102601.50	2.06	0.25	2.92E-17	3.78E-14
Bhlhe22	174.06	2.02	0.67	1.68E-04	8.08E-03
Tyrobp	257.29	2.00	0.56	2.48E-05	1.86E-03
Rgs4	572.45	1.98	0.59	5.81E-05	3.69E-03
Serpinb6b	147.96	1.98	0.52	1.27E-05	1.14E-03
Comp	185.85	1.97	0.71	2.24E-04	9.90E-03
Tnmd	1449.73	1.95	0.33	7.10E-10	2.47E-07
C1qa	662.14	1.93	0.56	2.77E-05	2.03E-03
Sult1e1	1383.88	1.91	0.56	2.99E-05	2.15E-03
Runx1	883.38	1.85	0.55	6.03E-05	3.81E-03
Uck2	571.16	1.84	0.36	4.22E-08	9.55E-06
Capn6	3255.31	1.80	0.20	5.09E-20	9.06E-17
Cyp7b1	1062.92	1.78	0.46	6.84E-06	6.81E-04
Slc16a3	520.27	1.77	0.48	1.40E-05	1.23E-03
Pdlim3	729.63	1.76	0.41	1.82E-06	2.12E-04
Lilrb4a	298.37	1.74	0.56	1.22E-04	6.37E-03
Nt5dc2	1609.76	1.73	0.26	4.97E-12	3.22E-09
Ctss	1502.33	1.72	0.29	6.78E-10	2.47E-07
Laptm5	730.58	1.72	0.59	1.60E-04	7.82E-03
Cd83	546.46	1.71	0.42	2.47E-06	2.80E-04
Ly6h	255.62	1.69	0.48	2.14E-05	1.69E-03
Apoe	31964.11	1.66	0.27	9.02E-11	3.78E-08
Pf4	153.52	1.64	0.69	6.47E-04	2.24E-02
Anln	409.19	1.61	0.53	1.45E-04	7.25E-03
H2-Aa	1966.60	1.59	0.42	1.07E-05	9.86E-04
Fcer1g	412.94	1.58	0.55	1.61E-04	7.82E-03
Myo7a	929.24	1.58	0.35	4.20E-07	6.50E-05
Timp1	2929.46	1.55	0.27	1.19E-09	3.95E-07
Kcnma1	514.21	1.55	0.39	4.45E-06	4.56E-04
Arxes2	317.18	1.54	0.41	1.01E-05	9.36E-04
Syndig1	677.49	1.53	0.43	2.27E-05	1.72E-03
H2-Ab1	3200.32	1.51	0.36	1.78E-06	2.10E-04
Rgs16	933.09	1.50	0.50	1.09E-04	5.88E-03
C1s2	202.34	1.47	0.74	1.36E-03	3.77E-02
Gata6	1343.98	1.46	0.35	2.48E-06	2.80E-04
C1qtnf3	22984.66	1.45	0.25	3.40E-10	1.31E-07
Cd68	313.41	1.45	0.79	1.85E-03	4.59E-02
Sdc1	3911.65	1.43	0.23	2.68E-11	1.32E-08
Arl4c	148.00	1.42	0.87	2.52E-03	5.67E-02
Sphk1	888.46	1.41	0.22	8.07E-12	4.93E-09
Lbh	542.08	1.41	0.44	7.76E-05	4.61E-03
9-Sep	1536.05	1.39	0.26	6.61E-09	1.82E-06
Csdc2	585.06	1.39	0.30	2.75E-07	4.84E-05
Pld4	264.97	1.37	0.63	9.97E-04	3.08E-02
Cdk1	240.66	1.35	0.60	9.24E-04	2.94E-02
Cdkn2b	385.27	1.35	0.41	5.44E-05	3.51E-03
Clu	9093.48	1.35	0.28	9.74E-08	2.04E-05
Pdgfa	622.98	1.35	0.29	3.31E-07	5.49E-05
Fndc5	518.41	1.34	0.28	8.28E-08	1.79E-05
Gpr39	244.03	1.34	0.69	1.61E-03	4.19E-02
Plxna4	259.78	1.34	0.64	1.30E-03	3.67E-02
Wnt4	699.90	1.34	0.59	9.02E-04	2.90E-02
Nectin2	1333.91	1.33	0.31	1.10E-06	1.44E-04
Arhgdib	893.82	1.30	0.30	6.55E-07	9.42E-05
Fcgr2b	374.28	1.30	0.64	1.28E-03	3.64E-02
H2-Eb1	1784.21	1.30	0.30	8.19E-07	1.13E-04

Cd74	9314.10	1.30	0.36	1.91E-05	1.55E-03
Garnl3	752.13	1.30	0.27	1.62E-07	3.16E-05
Rhod	456.58	1.28	0.29	7.58E-07	1.08E-04
Fscn1	3625.07	1.26	0.28	3.30E-07	5.49E-05
Egfl6	522.18	1.26	0.30	1.67E-06	2.04E-04
Mest	5380.45	1.25	0.40	7.60E-05	4.55E-03
Car3	1255.92	1.25	0.55	9.39E-04	2.95E-02
H2-DMb1	400.20	1.25	0.57	9.95E-04	3.08E-02
Lrmda	217.07	1.23	0.71	2.13E-03	5.03E-02
Npl	247.96	1.22	0.85	3.71E-03	7.39E-02
Schip1	339.27	1.22	0.35	2.50E-05	1.86E-03
Akap2	516.78	1.21	0.38	6.41E-05	3.96E-03
Nkd1	1033.74	1.21	0.29	1.43E-06	1.81E-04
Ccnb2	404.89	1.21	0.43	2.27E-04	9.97E-03
Ccl19	284.86	1.21	0.43	2.18E-04	9.76E-03
Lhfpl2	3193.67	1.20	0.26	1.99E-07	3.72E-05
Fabp5	510.58	1.19	0.27	4.41E-07	6.76E-05
Tspan11	1293.66	1.18	0.23	1.41E-08	3.51E-06
Mfap2	23491.13	1.18	0.13	8.15E-22	1.94E-18
Cxcl2	1147.46	1.18	0.87	3.53E-03	7.15E-02
Plekho1	317.86	1.16	0.52	9.69E-04	3.02E-02
Tpm2	2933.15	1.16	0.17	8.29E-13	5.91E-10
Pla2g7	587.20	1.15	0.41	2.32E-04	1.01E-02
Tmem98	2341.71	1.14	0.31	1.66E-05	1.40E-03
Gucy1a1	1861.35	1.14	0.24	1.38E-07	2.76E-05
Sox4	3960.08	1.13	0.20	1.23E-09	3.97E-07
Ednra	1081.65	1.13	0.37	1.17E-04	6.19E-03
F2r	5440.61	1.13	0.24	2.09E-07	3.87E-05
Shisa4	794.77	1.12	0.31	1.63E-05	1.38E-03
Adamts3	376.12	1.12	0.54	1.36E-03	3.77E-02
Nrep	9139.60	1.11	0.19	7.12E-10	2.47E-07
Gpc1	1388.40	1.11	0.35	8.03E-05	4.71E-03
Hvcn1	2141.02	1.11	0.17	1.68E-11	9.59E-09
Fhl2	1514.98	1.10	0.30	1.19E-05	1.08E-03
Mrvi1	949.52	1.08	0.63	2.37E-03	5.44E-02
Actn1	515.22	1.07	0.49	1.12E-03	3.29E-02
Acp5	1299.21	1.07	0.33	6.48E-05	3.98E-03
Piezo2	816.86	1.07	0.38	2.24E-04	9.90E-03
Rexo5	261.21	1.06	0.52	1.42E-03	3.87E-02
Slc27a3	871.91	1.06	0.33	8.37E-05	4.83E-03
Rbp1	6923.43	1.05	0.20	6.66E-09	1.82E-06
Cdh2	170.34	1.04	0.67	3.25E-03	6.77E-02
Stmn1	1412.11	1.03	0.29	2.11E-05	1.68E-03
Kcng4	308.36	1.03	0.34	1.20E-04	6.31E-03
Trf	11655.15	1.03	0.33	9.26E-05	5.24E-03
Sfrp1	18709.76	1.03	0.20	5.06E-08	1.13E-05
Palld	3978.60	1.02	0.15	1.58E-13	1.25E-10
Cxcl9	441.32	1.02	0.34	1.27E-04	6.62E-03
Depdc7	179.46	1.02	0.96	5.21E-03	9.18E-02
Unc119	409.29	1.01	0.44	9.26E-04	2.94E-02
Nxn	418.20	1.01	0.37	2.76E-04	1.16E-02
Art4	317.43	1.01	0.68	3.73E-03	7.41E-02
Cpz	4518.17	1.00	0.32	1.07E-04	5.81E-03
Cdc20	369.64	1.00	0.61	3.02E-03	6.41E-02
Wt1	888.64	0.99	0.47	1.28E-03	3.64E-02
Sfrp2	25180.41	0.98	0.19	1.27E-08	3.22E-06
Ctsc	6845.39	0.98	0.25	3.31E-06	3.49E-04
S1pr3	2298.17	0.98	0.54	2.36E-03	5.43E-02
Dkk3	8029.00	0.97	0.16	1.72E-10	7.02E-08
Id4	2503.65	0.97	0.32	1.15E-04	6.14E-03
Mgp	22617.91	0.95	0.20	2.14E-07	3.90E-05
Gm2564	483.96	0.95	0.42	9.62E-04	3.00E-02
Steap1	312.78	0.95	0.45	1.33E-03	3.71E-02
Cd14	1221.20	0.94	0.36	3.81E-04	1.52E-02
Atoh8	1285.82	0.94	0.30	9.16E-05	5.20E-03
Id2	7136.48	0.92	0.21	7.93E-07	1.12E-04
Mical2	624.20	0.92	0.43	1.23E-03	3.51E-02
Fblim1	766.31	0.92	0.48	2.07E-03	4.96E-02
Prlr	1339.62	0.92	0.32	1.94E-04	8.95E-03
Bgn	43868.75	0.92	0.22	1.78E-06	2.10E-04
Mgmt	289.30	0.91	0.37	5.82E-04	2.09E-02
Matn2	12253.34	0.90	0.14	4.63E-12	3.14E-09
Speg	248.66	0.90	0.57	3.33E-03	6.87E-02
Rasl11a	639.20	0.90	0.38	7.68E-04	2.54E-02
Adgra2	1981.25	0.90	0.22	2.05E-06	2.35E-04
BC028528	284.52	0.89	0.47	1.90E-03	4.67E-02
Igf2	177.73	0.89	0.54	3.11E-03	6.58E-02
Adamts15	2442.05	0.89	0.45	1.71E-03	4.37E-02
Sdc3	2231.45	0.88	0.29	1.41E-04	7.08E-03
Serf1	1239.45	0.88	0.25	2.80E-05	2.05E-03
Plagl1	3806.53	0.87	0.31	2.14E-04	9.69E-03
Tmem176b	12468.60	0.87	0.18	1.61E-07	3.16E-05
Agt	1880.82	0.87	0.50	2.79E-03	6.09E-02
Trabd2b	772.32	0.87	0.39	1.12E-03	3.29E-02
Mpzl1	3532.29	0.86	0.11	7.16E-17	8.50E-14
Dusp5	467.71	0.86	0.63	4.23E-03	8.01E-02
Maged2	24181.48	0.86	0.19	3.25E-07	5.49E-05
Tgfb3	9404.30	0.86	0.19	4.00E-07	6.37E-05
Csrp1	7241.56	0.85	0.11	6.60E-15	6.27E-12
Ak1	3407.41	0.84	0.12	6.41E-13	4.81E-10
Plxnd1	668.53	0.84	0.43	1.96E-03	4.77E-02
Tead2	599.42	0.84	0.30	3.06E-04	1.25E-02
Gucy1b1	809.90	0.83	0.31	3.83E-04	1.52E-02
Tmem132a	670.13	0.83	0.28	1.72E-04	8.19E-03
Limd2	1315.02	0.83	0.22	1.10E-05	1.00E-03
Gm10762	226.22	0.82	0.55	3.98E-03	7.73E-02
Adamts4	3545.73	0.82	0.35	8.52E-04	2.75E-02
Irak2	441.36	0.82	0.36	9.35E-04	2.95E-02
Lgmn	7447.83	0.81	0.18	2.84E-07	4.87E-05
Steap2	1073.46	0.81	0.35	9.84E-04	3.06E-02
Ror2	1377.11	0.81	0.28	2.35E-04	1.01E-02
Kif20a	548.08	0.81	0.67	5.43E-03	9.39E-02

Plk2	1668.96	0.80	0.17	2.62E-07	4.66E-05
Tcea3	1255.23	0.80	0.23	2.83E-05	2.06E-03
Cd1d1	618.20	0.80	0.40	1.93E-03	4.73E-02
Cdkn2c	563.45	0.79	0.31	5.02E-04	1.86E-02
St3gal4	2843.86	0.79	0.24	6.32E-05	3.94E-03
Igf2bp2	1166.31	0.78	0.26	1.32E-04	6.75E-03
Lpcat2	300.97	0.77	0.51	3.99E-03	7.73E-02
Cdkn1c	12728.74	0.77	0.21	1.81E-05	1.49E-03
Cd276	3035.82	0.77	0.19	2.57E-06	2.86E-04
Ddah1	558.03	0.77	0.29	4.44E-04	1.68E-02
Aoc3	3097.87	0.76	0.32	9.09E-04	2.91E-02
C1qtnf6	6007.84	0.75	0.18	3.56E-06	3.73E-04
Mpp6	2186.99	0.75	0.18	2.02E-06	2.34E-04
Sorcs2	1247.13	0.75	0.33	1.07E-03	3.20E-02
Azin2	992.47	0.74	0.27	3.43E-04	1.39E-02
Ypel1	353.00	0.74	0.60	5.71E-03	9.67E-02
Col16a1	13561.55	0.74	0.14	4.77E-09	1.42E-06
Col3a1	1879394.22	0.74	0.17	1.49E-06	1.88E-04
Il1r1	7737.35	0.73	0.21	3.30E-05	2.33E-03
Mex3b	808.38	0.73	0.26	2.42E-04	1.03E-02
Ephb3	2231.32	0.73	0.27	4.16E-04	1.62E-02
Bex3	1687.77	0.73	0.20	2.43E-05	1.83E-03
Nkain4	545.98	0.73	0.57	5.62E-03	9.54E-02
Tmem42	524.36	0.72	0.25	1.81E-04	8.50E-03
Nek6	2850.95	0.72	0.14	1.47E-08	3.62E-06
Tspan6	2952.19	0.72	0.27	4.41E-04	1.67E-02
Lrrn2	1875.99	0.72	0.32	1.09E-03	3.24E-02
Chst15	838.60	0.72	0.48	4.38E-03	8.23E-02
Bmper	10112.01	0.71	0.20	2.18E-05	1.71E-03
Col1a1	705890.24	0.71	0.25	2.81E-04	1.17E-02
Adgrl2	1432.45	0.70	0.31	1.16E-03	3.37E-02
Smim3	2193.24	0.70	0.31	1.32E-03	3.71E-02
Cyp1b1	6410.17	0.69	0.44	4.09E-03	7.86E-02
Dact3	2074.16	0.69	0.30	1.05E-03	3.17E-02
Psat1	1040.45	0.69	0.35	2.05E-03	4.95E-02
Cenpa	385.40	0.69	0.48	4.92E-03	8.78E-02
Ppic	23493.70	0.68	0.13	1.95E-08	4.72E-06
Sned1	4530.53	0.68	0.34	2.02E-03	4.90E-02
Col8a2	1920.19	0.68	0.44	4.66E-03	8.46E-02
Pdgfrl	9481.02	0.68	0.21	8.69E-05	4.97E-03
Tmem176a	5308.95	0.68	0.20	4.70E-05	3.16E-03
Tmem80	516.36	0.68	0.30	1.32E-03	3.70E-02
Trp53inp1	2045.52	0.68	0.22	1.34E-04	6.83E-03
Pafah1b3	664.23	0.67	0.26	4.98E-04	1.85E-02
Fdxr	944.94	0.67	0.29	1.11E-03	3.28E-02
Ldhb	1126.55	0.67	0.35	2.39E-03	5.47E-02
Slc24a3	1036.79	0.67	0.44	4.39E-03	8.23E-02
Tnfrsf23	697.12	0.67	0.31	1.61E-03	4.19E-02
Snx8	1100.96	0.67	0.26	6.33E-04	2.22E-02
Aass	309.88	0.67	0.52	5.84E-03	9.82E-02
Avpr1a	1623.06	0.65	0.22	1.61E-04	7.82E-03
Fbxw4	319.87	0.64	0.46	5.35E-03	9.31E-02
Rnf19b	1554.83	0.64	0.21	1.63E-04	7.88E-03
Adam19	3382.97	0.64	0.18	2.27E-05	1.72E-03
Pkdcc	1757.85	0.63	0.33	2.58E-03	5.78E-02
Myo1d	600.07	0.63	0.32	2.45E-03	5.55E-02
Pdzrn3	1827.07	0.62	0.24	6.20E-04	2.17E-02
Prr5	1250.40	0.62	0.35	3.40E-03	6.95E-02
Tmem86a	1198.22	0.62	0.28	1.49E-03	3.98E-02
Stom	3060.88	0.62	0.20	1.54E-04	7.58E-03
Tns3	521.93	0.62	0.31	2.19E-03	5.13E-02
Tpm1	11834.30	0.62	0.15	1.70E-06	2.05E-04
Phldb2	3849.44	0.62	0.20	1.25E-04	6.51E-03
Casp6	1634.77	0.61	0.23	4.30E-04	1.66E-02
Isyna1	1117.88	0.61	0.25	8.44E-04	2.73E-02
Cacnb3	1763.39	0.60	0.25	9.89E-04	3.07E-02
Fuca2	1823.76	0.60	0.26	1.14E-03	3.33E-02
Hacd1	2051.03	0.59	0.27	1.61E-03	4.19E-02
Tpbg	1725.55	0.59	0.19	1.16E-04	6.19E-03
Spon2	18455.75	0.59	0.30	2.70E-03	5.95E-02
Cfb	30072.91	0.59	0.15	4.32E-06	4.46E-04
Mmp14	46583.49	0.59	0.14	1.52E-06	1.88E-04
Cxcl16	2761.43	0.58	0.21	3.79E-04	1.51E-02
Gnpda1	1789.42	0.58	0.24	1.03E-03	3.15E-02
Dhrs3	7983.48	0.57	0.34	4.27E-03	8.07E-02
Itm2a	41934.44	0.57	0.14	4.72E-06	4.80E-04
Plod2	7075.94	0.57	0.17	5.10E-05	3.36E-03
Pycr1	2653.75	0.57	0.20	3.12E-04	1.27E-02
Stk17b	2263.98	0.56	0.26	1.77E-03	4.44E-02
Fkbp11	2224.26	0.56	0.20	3.09E-04	1.26E-02
Col5a2	46669.39	0.56	0.16	2.55E-05	1.89E-03
Ptprs	6361.59	0.56	0.16	5.41E-05	3.50E-03
Zfyve1	1215.35	0.56	0.23	1.00E-03	3.08E-02
Rgs19	1182.61	0.56	0.21	6.06E-04	2.14E-02
Ifi30	1495.09	0.56	0.22	8.17E-04	2.65E-02
Lum	225687.60	0.56	0.14	8.98E-06	8.59E-04
Fxyd5	4302.85	0.56	0.15	1.47E-05	1.29E-03
Wdr6	3108.06	0.55	0.14	5.51E-06	5.56E-04
Cpxm1	23753.60	0.54	0.16	5.32E-05	3.46E-03
Enpp1	2271.68	0.54	0.24	1.66E-03	4.27E-02
Arfgef2	1573.90	0.54	0.20	5.01E-04	1.86E-02
Lhpp	495.94	0.53	0.33	5.32E-03	9.30E-02
Col12a1	6466.14	0.53	0.31	4.43E-03	8.26E-02
Col1a2	655537.72	0.53	0.16	6.54E-05	3.99E-03
Igfbp7	73601.17	0.53	0.09	3.69E-10	1.38E-07
Rgs3	1468.75	0.52	0.32	5.26E-03	9.22E-02
Src	813.00	0.52	0.28	3.85E-03	7.57E-02
Crlf2	504.46	0.51	0.28	4.00E-03	7.74E-02
Igfbp4	54166.09	0.51	0.09	3.73E-09	1.13E-06
Vmp1	7540.48	0.51	0.11	1.68E-07	3.18E-05
Slc39a14	2810.75	0.51	0.29	4.57E-03	8.36E-02
Tceal9	10808.99	0.51	0.13	9.60E-06	8.99E-04

Chsy1	1734.00	0.51	0.23	2.15E-03	5.06E-02
Rgs10	838.24	0.50	0.22	1.61E-03	4.19E-02
Prrx2	2402.38	0.50	0.26	3.51E-03	7.14E-02
Lox	25916.77	0.50	0.19	7.54E-04	2.51E-02
Tmem53	941.53	0.49	0.29	5.22E-03	9.19E-02
Raly	3268.74	0.49	0.22	1.77E-03	4.44E-02
Spats2	1804.01	0.49	0.21	1.69E-03	4.35E-02
Olfml3	15665.51	0.49	0.19	7.16E-04	2.42E-02
Btg1	12529.70	0.48	0.24	3.14E-03	6.62E-02
Pld3	6666.00	0.48	0.14	6.56E-05	3.99E-03
Grb10	28105.85	0.48	0.16	2.86E-04	1.18E-02
Elk3	2648.21	0.48	0.13	2.94E-05	2.13E-03
Slc22a17	1934.45	0.47	0.18	1.00E-03	3.08E-02
Mafb	3117.18	0.47	0.28	4.98E-03	8.88E-02
B4galt5	1528.34	0.46	0.22	2.75E-03	6.03E-02
Mmp2	115941.83	0.46	0.09	9.14E-08	1.94E-05
L3hypdh	2218.83	0.45	0.16	5.40E-04	1.97E-02
Ctsk	20437.72	0.45	0.15	2.78E-04	1.16E-02
Ap1ar	2349.90	0.45	0.17	7.19E-04	2.43E-02
Aprt	2264.95	0.45	0.19	1.54E-03	4.10E-02
Msl2	1920.28	0.45	0.22	3.52E-03	7.15E-02
Nln	1865.78	0.45	0.22	3.30E-03	6.82E-02
Rgs12	1323.07	0.45	0.17	7.21E-04	2.43E-02
Sparc	616721.07	0.45	0.17	7.30E-04	2.45E-02
Plpp1	9796.49	0.44	0.21	2.86E-03	6.20E-02
Pfkm	1319.67	0.44	0.19	1.57E-03	4.13E-02
Fam49b	947.62	0.44	0.25	5.52E-03	9.45E-02
Rnf149	1301.19	0.44	0.21	3.50E-03	7.12E-02
Cnn2	4711.02	0.43	0.14	1.86E-04	8.67E-03
Fkbp10	17479.44	0.43	0.13	1.05E-04	5.72E-03
Ckap4	11254.93	0.43	0.12	5.48E-05	3.52E-03
Rab31	6708.08	0.43	0.16	6.63E-04	2.27E-02
Chpf	15296.77	0.42	0.14	2.82E-04	1.17E-02
Tubb2a	11454.38	0.42	0.13	1.03E-04	5.67E-03
Hif1a	9069.97	0.42	0.15	5.40E-04	1.97E-02
Serpinh1	201034.21	0.41	0.12	1.40E-04	7.08E-03
Cdkn1a	8022.77	0.41	0.18	2.20E-03	5.16E-02
Dera	1309.35	0.41	0.22	5.81E-03	9.80E-02
Csrp2	4849.13	0.41	0.21	5.24E-03	9.21E-02
Mmp23	14640.45	0.40	0.13	5.26E-04	1.93E-02
Slc35b2	5896.57	0.40	0.13	3.42E-04	1.39E-02
Gnb5	2015.57	0.39	0.17	2.36E-03	5.43E-02
Gpx7	6084.51	0.39	0.14	7.81E-04	2.56E-02
Rab5c	1582.66	0.39	0.18	3.22E-03	6.73E-02
Ebpl	1309.34	0.39	0.16	1.81E-03	4.49E-02
Pck2	2170.59	0.38	0.18	3.01E-03	6.40E-02
Cdk4	9107.73	0.38	0.10	1.97E-05	1.59E-03
Galnt10	4343.58	0.37	0.20	5.96E-03	9.98E-02
Plin2	7713.75	0.37	0.16	2.67E-03	5.91E-02
Ormdl3	4560.35	0.37	0.15	2.64E-03	5.86E-02
Lars2	73256.65	0.36	0.14	1.42E-03	3.87E-02
Idh2	5637.98	0.36	0.14	1.33E-03	3.71E-02
Sec61b	6075.86	0.36	0.17	4.12E-03	7.89E-02
Sccpdh	2337.42	0.36	0.17	4.52E-03	8.34E-02
Smyd2	1934.15	0.36	0.16	3.70E-03	7.38E-02
Litaf	10265.76	0.36	0.13	7.78E-04	2.56E-02
Maged1	26892.38	0.36	0.11	1.86E-04	8.67E-03
Sh3bp5	3052.59	0.35	0.16	3.92E-03	7.67E-02
Sh3bgrl3	5856.32	0.35	0.18	5.96E-03	9.98E-02
Arf5	10719.56	0.35	0.14	1.58E-03	4.15E-02
Hmgn2	10563.76	0.35	0.11	2.35E-04	1.01E-02
Pofut2	12283.70	0.35	0.15	2.98E-03	6.38E-02
B4galt2	2976.09	0.34	0.16	4.54E-03	8.35E-02
Atp6v0e	8464.90	0.34	0.17	5.78E-03	9.76E-02
Rad23b	3065.18	0.34	0.13	1.55E-03	4.11E-02
Ssr2	13868.75	0.34	0.14	2.13E-03	5.03E-02
Slc25a20	3086.97	0.34	0.16	4.57E-03	8.36E-02
Cnpy2	7871.10	0.33	0.15	3.21E-03	6.72E-02
Flcn	3942.15	0.33	0.16	5.34E-03	9.30E-02
Mapkapk2	6038.98	0.33	0.13	1.94E-03	4.74E-02
Dapk3	2641.74	0.33	0.13	2.07E-03	4.96E-02
Xpnpep1	5205.55	0.32	0.10	2.18E-04	9.76E-03
Lhfp	19576.41	0.32	0.10	2.19E-04	9.76E-03
Rcn1	21308.67	0.32	0.10	1.72E-04	8.19E-03
Col5a1	80857.84	0.31	0.12	9.54E-04	2.99E-02
Prdx5	8484.11	0.31	0.13	2.42E-03	5.51E-02
Ap2s1	3880.29	0.30	0.14	5.34E-03	9.30E-02
Cope	12108.77	0.30	0.11	1.05E-03	3.17E-02
Cd63	62220.11	0.30	0.14	5.36E-03	9.31E-02
Mcrip1	8103.41	0.30	0.12	2.73E-03	6.01E-02
Cmtm3	17081.57	0.30	0.10	4.96E-04	1.85E-02
Dbi	6010.57	0.30	0.11	1.18E-03	3.42E-02
Eno1	24361.90	0.30	0.11	1.19E-03	3.43E-02
Cdk5rap3	4806.62	0.30	0.14	5.92E-03	9.93E-02
Tmem9	4069.82	0.29	0.13	3.54E-03	7.16E-02
Htra1	50779.65	0.29	0.09	1.76E-04	8.31E-03
Itm2c	20552.39	0.29	0.11	2.07E-03	4.97E-02
Gosr2	8457.73	0.28	0.12	4.17E-03	7.94E-02
Dad1	9196.66	0.28	0.12	4.50E-03	8.32E-02
Colgalt1	11571.08	0.28	0.12	3.72E-03	7.40E-02
Ykt6	4789.89	0.27	0.11	3.28E-03	6.80E-02
H1f0	19798.81	0.27	0.12	4.36E-03	8.21E-02
Sparcl1	28547.89	0.26	0.09	1.32E-03	3.70E-02
Scube2	167.67	0.20	0.42	2.29E-03	5.28E-02
Wfdc17	239.42	0.16	0.32	1.07E-03	3.20E-02
Ms4a6c	86.39	0.16	0.31	1.11E-03	3.28E-02
Fgd6	74.69	0.16	0.32	4.11E-04	1.61E-02
Zfp57	83.34	0.15	0.29	4.92E-03	8.78E-02
Adamts17	104.01	0.15	0.29	6.15E-05	3.86E-03
Plekhg4	91.53	0.14	0.29	8.30E-12	4.93E-09
Clec4a3	145.25	0.14	0.27	9.46E-05	5.28E-03
Myrf	265.82	0.14	0.26	1.74E-03	4.43E-02

Kif26b	77.87	0.14	0.27	2.00E-11	1.09E-08
Agtr2	715.94	0.13	0.26	1.57E-04	7.71E-03
Lyz2	7638.76	0.13	0.26	2.09E-03	4.98E-02
Scn3b	176.57	0.13	0.25	4.37E-03	8.22E-02
Brinp3	135.20	0.12	0.24	1.93E-03	4.73E-02
Ripor3	115.69	0.12	0.24	4.50E-03	8.32E-02
C1ql3	61.92	0.12	0.24	2.92E-03	6.28E-02
Actg2	198.10	0.12	0.23	2.76E-03	6.04E-02
Ccl4	167.94	0.11	0.23	5.06E-05	3.36E-03
Pik3ap1	102.28	0.11	0.22	2.37E-04	1.02E-02
Cd93	85.60	0.11	0.22	6.05E-05	3.81E-03
Cd300a	106.29	0.10	0.22	2.87E-03	6.20E-02
Crabp2	100.92	0.10	0.22	8.55E-06	8.23E-04
Il1a	55.28	0.10	0.22	6.26E-09	1.82E-06
Tmem44	125.77	0.09	0.21	3.87E-03	7.60E-02
Calcb	82.63	0.09	0.20	3.38E-05	2.37E-03
Mmp12	95.87	0.08	0.20	4.11E-03	7.88E-02
Col9a3	51.49	0.08	0.20	1.96E-03	4.77E-02
Creg2	90.63	0.08	0.20	1.40E-03	3.84E-02
Adgre1	280.54	0.08	0.19	3.41E-03	6.95E-02
Mmp9	320.69	0.08	0.19	4.83E-03	8.66E-02
Ocstamp	34.70	0.07	0.19	3.56E-03	7.18E-02
Prss35	186.59	0.07	0.19	1.56E-03	4.12E-02
Cyp26a1	61.94	0.07	0.19	1.62E-05	1.38E-03
Itgb2	307.42	0.07	0.19	5.14E-03	9.08E-02
Cd244a	38.02	0.07	0.19	4.17E-07	6.50E-05
Lrrc15	829.89	0.06	0.19	4.38E-05	2.99E-03
Sirpb1c	47.21	0.06	0.19	9.45E-07	1.28E-04
Arhgap9	70.53	0.06	0.19	4.05E-03	7.79E-02
4930432K21Rik	24.41	0.06	0.18	1.50E-05	1.30E-03
Ctla2b	25.09	0.05	0.18	4.15E-04	1.62E-02
Uox	32.83	0.05	0.18	1.93E-04	8.92E-03
Cyp2e1	91.61	0.05	0.18	5.55E-03	9.47E-02
Fcgr4	59.78	0.05	0.18	5.42E-04	1.97E-02
Chl1	175.78	0.05	0.18	4.21E-03	7.98E-02
Calca	93.16	0.05	0.18	4.17E-03	7.94E-02
Prl2c2	65.57	0.05	0.18	6.87E-04	2.34E-02
Nxpe5	22.34	0.05	0.18	2.06E-03	4.95E-02
Fcgr1	40.39	0.05	0.18	1.56E-03	4.13E-02
Gm14851	52.34	0.05	0.18	6.39E-04	2.23E-02
C130050O18Rik	22.28	0.04	0.18	9.68E-05	5.37E-03
Gpr62	24.80	0.04	0.17	5.47E-03	9.41E-02
F2rl2	23.86	0.04	0.17	9.68E-05	5.37E-03
Sirpb1a	18.59	0.04	0.17	6.55E-04	2.26E-02
Defa29	28.49	0.03	0.17	5.29E-03	9.27E-02
Gpr34	34.13	0.02	0.17	1.75E-03	4.43E-02
St6galnac3	30.40	0.02	0.17	2.45E-03	5.55E-02
Matn3	28.66	0.02	0.17	2.89E-03	6.22E-02
Clec5a	22.44	0.02	0.17	3.98E-03	7.73E-02
Lct	44.31	0.02	0.17	2.63E-03	5.85E-02
Pkhd1	27.26	-0.02	0.17	1.86E-03	4.60E-02
Jph1	34.59	-0.02	0.17	8.18E-04	2.65E-02
Sstr1	20.12	-0.04	0.17	4.27E-04	1.65E-02
Baiap2l2	61.77	-0.04	0.17	4.81E-03	8.66E-02
Capn2	17086.81	-0.22	0.08	2.23E-03	5.19E-02
Lpar1	22572.01	-0.23	0.09	3.91E-03	7.65E-02
Esyt2	11386.85	-0.24	0.09	2.83E-03	6.16E-02
Aplp2	23609.45	-0.24	0.10	5.07E-03	9.00E-02
Rbpj	6951.42	-0.24	0.10	4.83E-03	8.66E-02
Tmem65	7102.70	-0.25	0.10	4.58E-03	8.36E-02
Timp2	243173.16	-0.25	0.10	2.66E-03	5.90E-02
Efr3a	9700.65	-0.25	0.09	1.29E-03	3.64E-02
Cul1	9097.31	-0.26	0.10	2.60E-03	5.80E-02
Fgfr1	20594.38	-0.26	0.12	5.44E-03	9.39E-02
Nptn	13677.96	-0.27	0.11	4.50E-03	8.32E-02
Clic4	28974.74	-0.27	0.10	2.03E-03	4.91E-02
Plxdc2	27005.76	-0.28	0.12	4.17E-03	7.94E-02
Kras	7419.29	-0.28	0.12	5.05E-03	8.98E-02
Mapre2	7637.63	-0.28	0.12	3.66E-03	7.35E-02
Pdgfra	26082.51	-0.28	0.12	4.47E-03	8.29E-02
Nckap1	17116.00	-0.29	0.10	1.05E-03	3.17E-02
Il6st	34068.31	-0.29	0.13	5.40E-03	9.35E-02
Anxa7	8545.30	-0.30	0.12	2.52E-03	5.67E-02
Pdlim5	5910.46	-0.30	0.13	3.19E-03	6.71E-02
Eif5	13888.35	-0.30	0.13	2.91E-03	6.27E-02
Ebf1	14358.18	-0.31	0.12	2.54E-03	5.70E-02
Fcho2	4590.36	-0.31	0.14	4.57E-03	8.36E-02
Pacsin2	8127.25	-0.31	0.14	5.46E-03	9.40E-02
Tns1	7987.55	-0.31	0.12	2.46E-03	5.56E-02
Slco3a1	7659.68	-0.31	0.12	1.55E-03	4.11E-02
Fam102b	13796.61	-0.31	0.13	2.41E-03	5.48E-02
Pkd2	14087.80	-0.31	0.11	6.48E-04	2.24E-02
Cd34	128982.41	-0.31	0.14	4.72E-03	8.55E-02
Stag2	4534.00	-0.32	0.15	5.33E-03	9.30E-02
Tmeff2	10700.43	-0.32	0.14	4.14E-03	7.91E-02
Nr3c1	11953.75	-0.33	0.12	1.18E-03	3.42E-02
Reep3	6845.61	-0.33	0.15	4.55E-03	8.35E-02
Rhoq	12834.99	-0.33	0.12	1.24E-03	3.55E-02
Pcif1	3656.44	-0.33	0.14	2.60E-03	5.80E-02
Prpf8	3481.77	-0.33	0.16	4.52E-03	8.34E-02
Dnm1	9623.44	-0.33	0.13	1.48E-03	3.97E-02
Zyx	24732.76	-0.33	0.13	1.49E-03	3.98E-02
Penk	47052.74	-0.33	0.15	2.95E-03	6.32E-02
Fam111a	3217.80	-0.34	0.16	4.64E-03	8.43E-02
Irgm2	6977.44	-0.34	0.17	4.39E-03	8.23E-02
Akap13	7536.50	-0.34	0.16	4.77E-03	8.60E-02
Tom1l2	3974.83	-0.34	0.16	4.12E-03	7.89E-02
Pcdh18	6190.13	-0.35	0.14	1.78E-03	4.45E-02
Arhgef10	4877.35	-0.35	0.17	5.52E-03	9.45E-02
Dpep1	48618.03	-0.35	0.13	6.71E-04	2.30E-02
Nfatc3	2458.17	-0.36	0.16	3.77E-03	7.47E-02
Esco1	1747.36	-0.36	0.18	5.92E-03	9.93E-02

Efemp1	94412.62	-0.36	0.18	5.25E-03	9.22E-02
Tax1bp1	14828.65	-0.36	0.13	1.12E-03	3.29E-02
Tmem106b	8024.85	-0.36	0.14	1.65E-03	4.27E-02
Ndrg1	6364.66	-0.36	0.13	1.08E-03	3.23E-02
Prkd3	4277.98	-0.36	0.17	4.43E-03	8.26E-02
Cmtm6	9362.57	-0.36	0.14	1.13E-03	3.30E-02
Klf3	13505.81	-0.36	0.18	5.58E-03	9.51E-02
Mill2	1929.27	-0.36	0.18	5.12E-03	9.08E-02
Zdhhc13	1444.70	-0.36	0.18	4.82E-03	8.66E-02
Wdfy2	2936.72	-0.36	0.17	4.45E-03	8.28E-02
Cd47	28015.31	-0.36	0.12	5.78E-04	2.08E-02
Arhgap12	4139.50	-0.36	0.15	2.05E-03	4.95E-02
Ncor1	5820.53	-0.36	0.16	2.95E-03	6.33E-02
Smarcc2	7111.80	-0.37	0.14	1.12E-03	3.29E-02
Stk3	3657.05	-0.37	0.15	1.62E-03	4.19E-02
Larp6	3168.05	-0.37	0.17	3.82E-03	7.53E-02
Irak3	6301.22	-0.37	0.14	1.43E-03	3.89E-02
Golim4	11286.75	-0.37	0.16	2.86E-03	6.20E-02
Cfh	23747.38	-0.37	0.14	1.31E-03	3.69E-02
Lats2	5802.11	-0.37	0.15	1.33E-03	3.71E-02
Mturn	7268.79	-0.37	0.18	3.57E-03	7.21E-02
Phf20	2061.48	-0.37	0.14	1.20E-03	3.45E-02
Smim10l1	4991.66	-0.37	0.12	4.36E-04	1.67E-02
Ppp1r15b	5828.14	-0.38	0.14	7.86E-04	2.57E-02
C1qtnf1	16632.26	-0.38	0.09	3.15E-06	3.38E-04
Slc29a1	23982.70	-0.38	0.15	1.75E-03	4.43E-02
Stk38	9411.73	-0.38	0.13	5.69E-04	2.05E-02
Arid4b	1913.27	-0.39	0.14	7.84E-04	2.57E-02
Zdhhc8	2267.36	-0.39	0.15	1.45E-03	3.91E-02
Klf9	11298.20	-0.39	0.11	9.30E-05	5.24E-03
Magt1	6488.73	-0.39	0.18	3.35E-03	6.89E-02
Mtf2	1190.24	-0.39	0.18	3.25E-03	6.77E-02
Hspa12a	2142.79	-0.39	0.19	3.69E-03	7.37E-02
Ufl1	3104.70	-0.39	0.17	2.16E-03	5.07E-02
Top2b	6581.57	-0.39	0.19	3.67E-03	7.36E-02
Nid1	146994.87	-0.40	0.09	2.90E-06	3.15E-04
Tubb4a	5521.05	-0.40	0.10	9.57E-06	8.99E-04
Papss2	6404.68	-0.40	0.15	9.18E-04	2.93E-02
Manea	3687.03	-0.40	0.20	4.55E-03	8.35E-02
Mfn2	4274.68	-0.40	0.20	4.57E-03	8.36E-02
Zbtb20	18534.73	-0.40	0.19	4.02E-03	7.76E-02
Sipa1	5433.49	-0.40	0.14	6.17E-04	2.17E-02
Cyfip1	11543.04	-0.40	0.15	9.36E-04	2.95E-02
Sdc2	41085.74	-0.40	0.13	2.23E-04	9.90E-03
Adgrd1	21087.47	-0.40	0.14	6.84E-04	2.33E-02
Mier3	2526.02	-0.40	0.21	5.83E-03	9.82E-02
Lamc1	65477.38	-0.40	0.12	7.66E-05	4.57E-03
Gapvd1	3898.37	-0.40	0.12	1.35E-04	6.85E-03
Heg1	23232.29	-0.40	0.16	1.37E-03	3.78E-02
Adamtsl4	11980.11	-0.41	0.16	1.41E-03	3.85E-02
Csf1	27067.22	-0.41	0.15	6.81E-04	2.33E-02
Lrch3	5318.31	-0.41	0.13	2.72E-04	1.15E-02
Axl	111258.22	-0.41	0.15	5.26E-04	1.93E-02
Ltbp4	89060.44	-0.41	0.21	4.62E-03	8.41E-02
Edem2	5210.99	-0.41	0.13	2.31E-04	1.01E-02
Wdfy3	3790.73	-0.41	0.20	3.34E-03	6.89E-02
Cd55	32533.95	-0.42	0.17	1.59E-03	4.17E-02
Atp2b1	3881.98	-0.42	0.23	5.81E-03	9.80E-02
Agap1	3561.29	-0.42	0.23	5.62E-03	9.54E-02
Atl2	3636.41	-0.42	0.12	7.16E-05	4.32E-03
Smc1a	5628.70	-0.42	0.17	1.36E-03	3.76E-02
1700047I17Rik2	4050.90	-0.42	0.20	2.84E-03	6.17E-02
Cpd	6356.28	-0.42	0.14	3.74E-04	1.50E-02
Qpct	23535.72	-0.42	0.15	7.71E-04	2.54E-02
Ifngr1	24387.27	-0.43	0.12	3.27E-05	2.32E-03
Cpne3	3908.19	-0.43	0.18	1.92E-03	4.71E-02
Scaf8	1902.53	-0.43	0.21	3.38E-03	6.92E-02
Mbnl1	20370.93	-0.43	0.15	3.75E-04	1.50E-02
Elmsan1	1074.93	-0.43	0.23	5.13E-03	9.08E-02
Bcor	1041.61	-0.43	0.22	4.18E-03	7.94E-02
Tgfbi	33155.36	-0.44	0.19	2.21E-03	5.16E-02
Fn1	209665.48	-0.44	0.19	1.77E-03	4.44E-02
Otud1	1394.08	-0.45	0.19	1.90E-03	4.67E-02
Pard3	2123.09	-0.45	0.23	4.18E-03	7.94E-02
Glud1	11317.61	-0.45	0.12	1.87E-05	1.53E-03
Ifih1	2283.88	-0.45	0.16	4.27E-04	1.65E-02
Trip10	14668.44	-0.46	0.12	1.47E-05	1.29E-03
Mapk8ip3	2000.73	-0.46	0.15	2.17E-04	9.76E-03
Xdh	31622.59	-0.46	0.20	2.11E-03	5.01E-02
Hmcn2	14495.41	-0.46	0.26	5.53E-03	9.45E-02
Ces1d	13923.01	-0.46	0.17	4.97E-04	1.85E-02
Prelp	38933.31	-0.46	0.17	6.49E-04	2.24E-02
Pold3	1496.47	-0.46	0.27	5.47E-03	9.41E-02
Slc43a3	50166.90	-0.46	0.23	3.69E-03	7.37E-02
Atp6v0e2	2157.72	-0.47	0.17	5.49E-04	1.99E-02
Atp7a	1726.08	-0.47	0.19	1.39E-03	3.81E-02
Tctn3	957.48	-0.47	0.23	3.30E-03	6.82E-02
Cry2	3516.04	-0.47	0.15	2.32E-04	1.01E-02
Hs3st1	1897.40	-0.47	0.22	2.67E-03	5.90E-02
Lamb2	37232.27	-0.47	0.15	2.14E-04	9.69E-03
Naa15	1985.85	-0.47	0.21	2.12E-03	5.03E-02
Bmp4	6244.76	-0.47	0.24	3.38E-03	6.92E-02
Traf5	1700.61	-0.48	0.16	2.28E-04	9.99E-03
Bclaf1	5094.02	-0.48	0.21	1.74E-03	4.43E-02
Slc37a4	1021.86	-0.48	0.26	4.29E-03	8.09E-02
Csgalnact2	6317.11	-0.48	0.14	5.07E-05	3.36E-03
Pgf	910.97	-0.48	0.25	3.70E-03	7.37E-02
Ncoa2	1730.27	-0.48	0.23	2.75E-03	6.03E-02
Slc1a4	1200.83	-0.48	0.25	3.62E-03	7.29E-02
Cast	13511.35	-0.48	0.16	2.10E-04	9.58E-03
Cask	3704.32	-0.48	0.18	4.58E-04	1.73E-02
Adamts5	9890.96	-0.49	0.15	8.25E-05	4.80E-03

Kcnk5	2477.14	-0.49	0.23	2.48E-03	5.61E-02
Gsn	2378472.30	-0.49	0.14	3.47E-05	2.42E-03
Rb1cc1	3355.99	-0.49	0.21	1.51E-03	4.02E-02
Sirpa	10419.89	-0.49	0.15	1.03E-04	5.67E-03
Slmap	4033.28	-0.49	0.19	7.89E-04	2.57E-02
Sptbn1	25818.55	-0.49	0.11	6.02E-07	8.92E-05
Zhx2	1179.43	-0.50	0.26	3.81E-03	7.51E-02
Sacm1l	6565.34	-0.50	0.14	2.25E-05	1.72E-03
Mindy2	3473.91	-0.50	0.16	1.98E-04	9.09E-03
Ncoa6	1529.63	-0.50	0.19	7.01E-04	2.38E-02
Clock	2631.49	-0.50	0.21	1.35E-03	3.75E-02
Ece1	9225.15	-0.50	0.12	3.01E-06	3.25E-04
Btaf1	1520.44	-0.50	0.20	1.06E-03	3.19E-02
Uap1	27739.75	-0.50	0.11	2.81E-07	4.87E-05
Pkia	3271.08	-0.50	0.23	1.86E-03	4.60E-02
Smoc2	100289.06	-0.51	0.25	2.88E-03	6.21E-02
Ackr3	55531.47	-0.51	0.19	7.37E-04	2.47E-02
Prrg1	1612.77	-0.51	0.27	3.62E-03	7.29E-02
Gab1	2449.83	-0.51	0.22	1.78E-03	4.45E-02
Scg3	1603.36	-0.51	0.29	4.70E-03	8.52E-02
Syde2	2345.70	-0.51	0.24	2.40E-03	5.47E-02
Orc2	1197.96	-0.51	0.22	1.43E-03	3.89E-02
Pcolce2	37456.65	-0.51	0.16	1.52E-04	7.53E-03
Ccl11	50336.05	-0.51	0.22	1.44E-03	3.91E-02
Slc30a1	432.36	-0.51	0.32	5.58E-03	9.51E-02
Gnpnat1	10194.97	-0.52	0.20	7.55E-04	2.51E-02
Ahnak	24923.04	-0.52	0.12	1.51E-06	1.88E-04
Wnt11	6033.80	-0.52	0.16	1.32E-04	6.75E-03
Gm14440	684.26	-0.52	0.32	5.50E-03	9.44E-02
Pdzd2	1689.79	-0.52	0.28	3.95E-03	7.70E-02
2610002M06Rik	1531.68	-0.52	0.21	9.29E-04	2.94E-02
Lims2	8687.05	-0.52	0.13	7.69E-06	7.50E-04
Gstm1	64030.93	-0.52	0.15	5.32E-05	3.46E-03
Pla2r1	3369.80	-0.52	0.21	9.46E-04	2.97E-02
Kif13a	1555.97	-0.53	0.25	2.22E-03	5.17E-02
Scn7a	11455.60	-0.53	0.23	1.69E-03	4.34E-02
Ahctf1	2612.31	-0.53	0.22	1.11E-03	3.28E-02
Clip1	6490.13	-0.53	0.19	3.55E-04	1.43E-02
Itih5	33545.05	-0.53	0.11	4.03E-07	6.37E-05
Wdr3	1832.08	-0.53	0.27	3.00E-03	6.40E-02
Elmo1	1805.76	-0.53	0.33	5.40E-03	9.35E-02
Fndc1	90357.52	-0.53	0.12	8.04E-07	1.12E-04
Spry4	1026.41	-0.53	0.34	5.76E-03	9.75E-02
Esf1	1403.28	-0.53	0.26	2.27E-03	5.26E-02
Slco2b1	5076.27	-0.54	0.28	3.09E-03	6.55E-02
Kbtbd7	955.28	-0.55	0.30	3.89E-03	7.63E-02
Bmp6	2600.76	-0.55	0.28	3.19E-03	6.71E-02
Dennd4c	2264.41	-0.55	0.30	3.77E-03	7.47E-02
Acsl3	1492.08	-0.55	0.24	1.73E-03	4.43E-02
Uaca	2321.91	-0.56	0.29	3.14E-03	6.62E-02
Phka1	1373.21	-0.56	0.27	2.45E-03	5.55E-02
Lrrk2	1512.51	-0.56	0.29	2.74E-03	6.01E-02
Gm4631	3063.72	-0.56	0.23	1.05E-03	3.17E-02
Traf3	1121.01	-0.56	0.35	5.02E-03	8.94E-02
Taf7	1190.35	-0.56	0.30	3.38E-03	6.92E-02
Pdxk	5731.46	-0.56	0.29	3.10E-03	6.55E-02
Camk1d	1289.81	-0.56	0.30	3.20E-03	6.72E-02
Lrch1	1876.46	-0.57	0.26	1.78E-03	4.45E-02
Zeb1	4826.01	-0.57	0.13	1.06E-06	1.40E-04
Tbc1d32	641.27	-0.57	0.37	5.38E-03	9.33E-02
Frmd4a	1071.70	-0.57	0.32	3.81E-03	7.51E-02
Fyn	21200.95	-0.57	0.12	1.34E-07	2.72E-05
Zfp868	765.71	-0.57	0.25	1.35E-03	3.75E-02
Polr3e	1400.46	-0.57	0.26	1.63E-03	4.21E-02
Ankhd1	2277.74	-0.57	0.18	1.17E-04	6.19E-03
Krt18	1041.53	-0.58	0.21	4.41E-04	1.67E-02
Ppp1r12b	3259.10	-0.58	0.20	2.51E-04	1.06E-02
Flrt3	5211.26	-0.59	0.20	2.25E-04	9.90E-03
Tsc22d3	18982.36	-0.59	0.19	1.22E-04	6.37E-03
Abcb6	1165.49	-0.59	0.25	1.09E-03	3.25E-02
Abca2	2617.61	-0.59	0.21	3.01E-04	1.24E-02
1810010H24Rik	1044.67	-0.59	0.18	7.81E-05	4.62E-03
Golga4	2178.03	-0.59	0.16	2.22E-05	1.72E-03
Cdh13	4996.80	-0.59	0.25	1.02E-03	3.13E-02
Ephx4	863.01	-0.60	0.25	1.03E-03	3.15E-02
Zfp955a	976.57	-0.60	0.29	2.09E-03	4.99E-02
Zfp385a	6907.01	-0.60	0.13	1.65E-07	3.18E-05
Phf3	1702.62	-0.60	0.23	5.94E-04	2.11E-02
Synj1	1218.12	-0.60	0.27	1.38E-03	3.81E-02
Rif1	889.30	-0.60	0.37	4.50E-03	8.32E-02
Ablim1	20042.96	-0.60	0.18	4.08E-05	2.81E-03
Nr1d1	12222.90	-0.60	0.21	3.06E-04	1.25E-02
Abca5	1545.92	-0.61	0.26	1.19E-03	3.43E-02
Nipbl	1531.79	-0.61	0.20	1.71E-04	8.19E-03
Cadm3	1363.62	-0.61	0.24	7.48E-04	2.50E-02
Dpp4	26082.45	-0.61	0.37	4.41E-03	8.24E-02
Enpp2	22285.76	-0.61	0.19	9.75E-05	5.38E-03
Prdm8	1776.28	-0.61	0.26	1.03E-03	3.16E-02
Loxl4	1695.19	-0.61	0.28	1.48E-03	3.97E-02
Dact1	16457.39	-0.61	0.16	6.46E-06	6.48E-04
Colgalt2	1811.50	-0.62	0.30	2.03E-03	4.92E-02
Slc25a23	907.24	-0.62	0.34	3.27E-03	6.80E-02
Ercc6l2	1221.07	-0.62	0.30	2.12E-03	5.03E-02
Lama2	18588.46	-0.62	0.12	2.42E-08	5.56E-06
Podn	10508.55	-0.62	0.24	6.01E-04	2.13E-02
Ifit2	3491.29	-0.63	0.19	7.94E-05	4.67E-03
Dennd1a	1521.17	-0.63	0.20	1.41E-04	7.08E-03
Tmod2	3417.99	-0.63	0.14	5.40E-07	8.10E-05
Aif1l	5671.49	-0.63	0.39	4.64E-03	8.43E-02
Islr2	872.54	-0.63	0.25	7.52E-04	2.51E-02
Zfp53	870.41	-0.64	0.31	1.98E-03	4.81E-02
Fgf2	4025.38	-0.64	0.28	1.16E-03	3.37E-02

Trim16	2871.42	-0.64	0.19	3.75E-05	2.61E-03
Lamc2	2650.22	-0.64	0.25	6.45E-04	2.24E-02
Ktn1	3008.93	-0.65	0.20	8.09E-05	4.72E-03
Osr2	4863.96	-0.65	0.32	2.25E-03	5.21E-02
Man1a	31081.11	-0.65	0.14	6.07E-07	8.92E-05
Nr3c2	465.18	-0.65	0.43	4.81E-03	8.66E-02
Timp3	59678.93	-0.65	0.13	2.07E-08	4.91E-06
Zfp292	1262.74	-0.65	0.41	4.40E-03	8.24E-02
Tgfbr3	28989.66	-0.65	0.12	9.46E-09	2.45E-06
Wdr11	2717.73	-0.66	0.23	2.39E-04	1.02E-02
Smc3	4212.33	-0.66	0.24	4.27E-04	1.65E-02
Nqo1	659.83	-0.66	0.33	2.24E-03	5.21E-02
Smad5	4082.14	-0.66	0.19	3.77E-05	2.61E-03
Ptprj	1472.45	-0.66	0.28	1.05E-03	3.17E-02
Nol8	476.96	-0.66	0.44	4.83E-03	8.66E-02
Entpd1	2728.79	-0.67	0.17	7.38E-06	7.25E-04
Ppip5k1	928.84	-0.67	0.25	3.99E-04	1.57E-02
Abca6	7641.78	-0.68	0.22	1.72E-04	8.19E-03
Samhd1	3214.88	-0.69	0.18	7.33E-06	7.25E-04
Itga11	14200.04	-0.69	0.23	1.97E-04	9.07E-03
Ankrd12	3229.42	-0.69	0.36	2.52E-03	5.67E-02
Cwf19l2	1403.88	-0.69	0.26	4.38E-04	1.67E-02
Dmd	2305.87	-0.69	0.22	8.44E-05	4.85E-03
Rock2	7769.65	-0.70	0.19	1.72E-05	1.43E-03
Ebf2	8943.34	-0.70	0.19	1.65E-05	1.39E-03
Pcsk6	32154.66	-0.70	0.21	4.72E-05	3.16E-03
Gcnt3	490.37	-0.70	0.50	5.14E-03	9.08E-02
Heatr5b	1226.80	-0.70	0.26	4.33E-04	1.66E-02
Emx2	784.06	-0.70	0.41	3.41E-03	6.95E-02
Abca8b	4709.18	-0.70	0.46	4.44E-03	8.27E-02
Adam33	2974.53	-0.70	0.16	6.44E-07	9.36E-05
Rabep1	3135.64	-0.71	0.24	1.92E-04	8.89E-03
Icosl	1930.18	-0.71	0.37	2.32E-03	5.36E-02
Fgl2	32630.28	-0.71	0.32	1.58E-03	4.15E-02
Bdh1	436.52	-0.71	0.53	5.33E-03	9.30E-02
Rorb	791.26	-0.72	0.27	4.57E-04	1.73E-02
Etv1	533.06	-0.72	0.46	3.99E-03	7.73E-02
Galnt15	5323.68	-0.72	0.24	1.49E-04	7.43E-03
Car2	2904.87	-0.73	0.56	5.61E-03	9.54E-02
Bmp2k	743.00	-0.73	0.27	3.67E-04	1.48E-02
Farp2	859.40	-0.73	0.38	2.28E-03	5.26E-02
Tpr	4645.72	-0.74	0.22	4.45E-05	3.02E-03
Gpr165	424.96	-0.74	0.34	1.45E-03	3.91E-02
Hao2	852.85	-0.74	0.58	5.66E-03	9.60E-02
Adam22	828.42	-0.74	0.36	1.84E-03	4.56E-02
Zfp229	581.46	-0.74	0.55	5.16E-03	9.10E-02
Tmtc3	2660.19	-0.75	0.26	2.02E-04	9.23E-03
Creb5	4133.88	-0.77	0.20	9.56E-06	8.99E-04
Zfp638	3204.02	-0.77	0.24	8.84E-05	5.04E-03
Sclt1	693.19	-0.78	0.30	4.32E-04	1.66E-02
Tspan15	551.04	-0.78	0.57	4.90E-03	8.77E-02
Pcgf5	1061.96	-0.79	0.31	5.92E-04	2.11E-02
Abca8a	29566.54	-0.79	0.21	1.36E-05	1.21E-03
Cldn3	1010.29	-0.79	0.32	6.62E-04	2.27E-02
Zfp518a	649.22	-0.80	0.42	2.19E-03	5.13E-02
Dkk2	1719.19	-0.80	0.30	3.92E-04	1.55E-02
Stxbp6	5216.40	-0.80	0.26	1.09E-04	5.88E-03
2610528A11Rik	459.96	-0.80	0.53	4.04E-03	7.79E-02
Fam208b	1492.67	-0.81	0.59	4.76E-03	8.60E-02
Gm3055	540.28	-0.81	0.39	1.51E-03	4.02E-02
Klf12	444.43	-0.82	0.37	1.26E-03	3.58E-02
Epcam	3562.61	-0.82	0.51	3.64E-03	7.31E-02
Gk5	823.91	-0.82	0.20	2.51E-06	2.81E-04
Musk	917.16	-0.82	0.43	2.11E-03	5.01E-02
Rgmb	11295.26	-0.82	0.18	4.64E-07	7.03E-05
Crispld1	3286.90	-0.83	0.27	1.29E-04	6.67E-03
Sdr42e1	447.57	-0.83	0.33	5.95E-04	2.11E-02
Ptprd	1124.61	-0.84	0.42	1.77E-03	4.44E-02
Sult1d1	1197.91	-0.84	0.42	1.80E-03	4.49E-02
Gnao1	260.59	-0.85	0.50	3.06E-03	6.49E-02
Sos1	1421.84	-0.85	0.29	1.83E-04	8.56E-03
Krt80	1244.70	-0.86	0.36	7.57E-04	2.51E-02
Msh2	1338.99	-0.87	0.24	2.11E-05	1.68E-03
Lepr	6633.70	-0.89	0.29	1.29E-04	6.67E-03
Hip1r	500.06	-0.93	0.47	1.74E-03	4.43E-02
Fsd1l	676.71	-0.93	0.55	2.88E-03	6.21E-02
Msh3	454.02	-0.94	0.49	2.08E-03	4.98E-02
Prokr1	2423.11	-0.97	0.22	1.02E-06	1.36E-04
Cnmd	1004.93	-0.97	0.39	5.33E-04	1.95E-02
Omd	2194.27	-0.98	0.40	5.87E-04	2.10E-02
Ptgfr	2624.62	-0.99	0.35	2.48E-04	1.06E-02
Nr1d2	11042.81	-1.00	0.31	7.44E-05	4.47E-03
Akr1c12	562.37	-1.03	0.66	3.33E-03	6.87E-02
Eea1	2372.99	-1.03	0.22	2.45E-07	4.42E-05
Negr1	709.92	-1.03	0.42	6.20E-04	2.17E-02
Zc3h6	454.03	-1.04	0.75	4.05E-03	7.79E-02
Sarm1	269.91	-1.04	0.47	1.07E-03	3.21E-02
Chml	294.41	-1.06	0.49	1.19E-03	3.43E-02
Prkaa2	588.22	-1.07	0.30	1.59E-05	1.37E-03
Sntb1	2387.69	-1.08	0.35	1.12E-04	6.03E-03
Pcdh9	223.85	-1.10	0.56	1.77E-03	4.44E-02
Moxd1	551.30	-1.11	0.71	3.26E-03	6.77E-02
Arhgef28	909.26	-1.11	0.57	1.76E-03	4.44E-02
Ppp1r1b	514.01	-1.13	0.33	2.69E-05	1.99E-03
Gm33869	133.91	-1.14	0.81	3.78E-03	7.48E-02
Hspa4l	1270.59	-1.15	0.36	8.36E-05	4.83E-03
Ppl	3961.16	-1.15	0.39	1.74E-04	8.25E-03
Duox1	2001.45	-1.16	0.39	1.50E-04	7.43E-03
Oit1	757.26	-1.17	0.39	1.32E-04	6.75E-03
Bicd1	438.89	-1.18	0.71	2.73E-03	6.00E-02
Adh7	426.52	-1.18	0.42	2.51E-04	1.06E-02
Sgpp2	227.32	-1.20	1.23	5.45E-03	9.40E-02

Ackr2	2852.51	-1.23	0.20	6.47E-11	2.79E-08
Pigr	3591.99	-1.24	0.29	1.17E-06	1.51E-04
Lrba	198.34	-1.24	0.49	4.88E-04	1.83E-02
Akr1c13	848.97	-1.26	0.34	1.27E-05	1.14E-03
Sema3e	953.29	-1.28	0.29	9.65E-07	1.30E-04
Tmprss2	667.27	-1.39	0.56	5.50E-04	1.99E-02
Ap5b1	231.60	-1.40	0.68	1.47E-03	3.95E-02
Hlf	2948.16	-1.43	0.40	2.16E-05	1.70E-03
Igsf9b	215.18	-1.44	0.63	9.10E-04	2.91E-02
Kcne3	337.10	-1.57	0.48	5.21E-05	3.42E-03
Sucnr1	164.28	-1.59	0.63	5.23E-04	1.93E-02
2610021A01Rik	607.76	-1.60	0.48	4.39E-05	2.99E-03
Akr1c19	1112.23	-1.67	0.46	1.89E-05	1.54E-03
Tll1	214.92	-1.93	0.45	1.65E-06	2.02E-04
Gm28035	34.53	-2.27	1.25	2.62E-03	5.84E-02
Zfp976	418.09	-2.40	0.79	1.57E-04	7.71E-03
Prdm9	112.64	-2.41	0.94	4.82E-04	1.81E-02
Aldh1a7	49.89	-2.46	1.54	3.21E-03	6.72E-02
Kl	23.95	-2.65	1.43	2.64E-03	5.87E-02
Exd1	130.02	-2.69	0.82	6.34E-05	3.94E-03
Slfn4	68.66	-2.75	2.12	3.99E-03	7.73E-02
Mlip	118.90	-2.88	1.42	2.39E-03	5.47E-02
Atp2a1	151.27	-2.88	1.04	2.95E-04	1.22E-02
Mrgprg	4094.30	-2.88	1.02	2.76E-04	1.16E-02
9330159F19Rik	720.64	-3.13	1.36	9.28E-04	2.94E-02
Cyfip2	112.16	-3.49	4.87	4.19E-03	7.95E-02
Trim66	66.69	-3.52	0.94	2.28E-05	1.72E-03
Ttc39a	149.16	-3.60	2.01	1.65E-03	4.27E-02
Gabra2	32.12	-3.81	1.29	3.86E-04	1.53E-02
Fyb2	90.07	-4.37	5.11	3.00E-03	6.40E-02
Ang4	707.31	-4.38	1.10	8.33E-06	8.07E-04
Kcnq1	188.77	-4.39	1.37	4.71E-05	3.16E-03
Noxa1	102.07	-4.68	2.82	1.19E-03	3.43E-02
Ccdc89	50.65	-4.80	2.52	8.42E-04	2.72E-02
Myh4	93.71	-4.84	1.18	3.13E-05	2.24E-03
Nyap1	25.42	-4.85	1.54	2.48E-04	1.06E-02
Cacna1h	28.77	-6.07	1.73	9.38E-05	5.26E-03
BC016579	37.56	-6.45	1.80	2.26E-05	1.72E-03
Ifi206	77.21	-7.57	1.60	7.68E-08	1.68E-05
Gprin3	23.99	-8.74	3.23	5.76E-05	3.68E-03
Pkd1l2	39.48	-9.43	3.13	1.39E-06	1.79E-04
Rundc3b	44.12	-10.09	3.07	7.76E-09	2.09E-06
