# Supplementary material for: Elucidating the fundamental fibrotic processes driving abdominal adhesion formation
Source: Nat Commun. 2020 Aug 13;11:4061. doi: 10.1038/s41467-020-17883-1 (PMC7426428; doi:10.1038/s41467-020-17883-1)
Supplement: Supplementary file 4 — Supplementary Data 2 [file 41467_2020_17883_MOESM4_ESM.pdf]

Supplementary Dataset 2: Genes differentially expressed between human fibroblasts isolated from abdominal adhesions ('Adhesions') versus control peritoneum tissue ('Healthy') by DESeq2

This workbook contains one sheet:
Sheet 1: Table of differentially expressed genes between human fibroblasts isolated from abdominal adhesions (*n* = 6 samples) and control peritoneal tissue (*n* = 3 samples) after performing lfcShrink using Approximate Posterior Estimation for GLM (apeglm) in DESeq2. A significance threshold of *P-adjusted* < 0.1 was used to filter genes. Positive log2FoldChange values correspond to genes significantly enriched in abdominal adhesions compared to healthy controls.

GeneID	baseMean	log2FoldChange	lfcSE	pvalue	padj
TBC1D3D	66.57	10.21	3.06	1.56E-11	7.02E-09
ALPK2	321.19	6.11	1.04	4.59E-11	1.85E-08
KLF17	23.39	6.07	1.71	9.93E-06	4.55E-04
DLL3	6.62	5.85	2.78	9.85E-05	2.49E-03
GUCA1A	36.02	5.48	1.37	3.23E-06	1.97E-04
MAB21L2	1003.01	5.36	1.04	2.26E-09	5.42E-07
PTPN22	2468.02	5.21	1.04	6.28E-09	1.32E-06
LRRN1	7966.96	5.13	1.11	3.22E-08	5.04E-06
EPN3	56.70	4.87	1.27	2.23E-06	1.47E-04
GJB2	2655.17	4.85	0.80	1.45E-11	6.80E-09
IGDCC3	54.14	4.84	0.86	6.00E-10	1.61E-07
PROC	9.41	4.76	1.44	4.15E-05	1.33E-03
EVI2A	1131.46	4.68	0.79	3.91E-11	1.67E-08
TNFRSF9	906.51	4.59	0.96	2.05E-08	3.50E-06
IL21R	275.54	4.57	1.16	8.58E-07	7.11E-05
SLC12A8	4130.34	4.46	1.02	1.32E-07	1.63E-05
IQGAP2	845.26	4.17	1.12	2.15E-06	1.43E-04
C19orf81	10.69	3.94	1.47	4.23E-04	7.21E-03
ADAM12	13955.97	3.91	1.10	4.31E-06	2.46E-04
ADD2	1882.55	3.77	1.08	5.52E-06	2.90E-04
F2RL2	506.88	3.75	1.10	8.22E-06	3.98E-04
ACSBG2	12.97	3.54	1.42	3.64E-04	6.51E-03
CD22	102.78	3.53	1.11	2.17E-05	8.05E-04
SDC1	1898.33	3.51	0.84	4.93E-07	4.56E-05
SAMD5	149.14	3.47	0.81	3.48E-07	3.56E-05
SPTSSB	92.79	3.46	1.25	6.52E-05	1.87E-03
C1QTNF6	11609.83	3.45	0.70	1.46E-08	2.61E-06
ATP1B1	5355.55	3.44	0.81	3.16E-07	3.41E-05
STRA6	2322.68	3.42	1.01	9.00E-06	4.25E-04
SAMD3	113.80	3.31	1.07	2.96E-05	1.03E-03
NLRP2	25.59	3.30	1.10	6.18E-05	1.79E-03
KLK13	16.53	3.29	1.03	5.16E-05	1.56E-03
C3orf80	1970.33	3.24	0.98	1.31E-05	5.54E-04
PLEKHN1	95.54	3.23	0.77	6.22E-07	5.49E-05
SMTNL2	2806.46	3.21	1.11	4.56E-05	1.42E-03
COL24A1	1734.84	3.18	0.82	1.71E-06	1.23E-04
COL1A1	6550287.27	3.17	0.81	1.60E-06	1.17E-04
PRSS35	1230.38	3.17	0.95	1.28E-05	5.47E-04
SHANK1	25.61	3.11	1.36	3.51E-04	6.37E-03
COL3A1	4484053.61	3.02	0.98	2.79E-05	9.87E-04
GCNT1	986.16	3.02	0.38	5.06E-17	9.70E-14
KIF26B	637.99	2.99	0.70	3.46E-07	3.56E-05
SLC38A5	1342.90	2.98	0.95	2.53E-05	9.19E-04
DEF6	563.53	2.98	0.93	2.15E-05	8.04E-04
ADAMTS14	4752.23	2.93	0.96	3.44E-05	1.15E-03
CILP2	9323.87	2.91	1.01	5.52E-05	1.64E-03
RUNX2	1359.05	2.90	0.45	2.87E-12	1.84E-09
NEDD4L	1137.38	2.87	1.15	1.58E-04	3.53E-03
CDH2	1169.11	2.86	1.03	7.67E-05	2.09E-03
SIT1	52.65	2.85	1.30	3.60E-04	6.46E-03
EVA1A	501.97	2.79	0.60	6.95E-08	9.33E-06
ZNF114	65.74	2.77	0.46	7.78E-11	2.71E-08
KERA	593.34	2.73	1.27	3.44E-04	6.28E-03
DUSP4	1113.19	2.72	0.81	1.41E-05	5.84E-04
GSDMA	136.43	2.72	1.13	2.20E-04	4.51E-03
CDC45	97.73	2.70	1.28	4.16E-04	7.12E-03
CSTA	379.21	2.69	1.00	1.10E-04	2.69E-03
TEX35	21.18	2.67	1.31	7.02E-04	1.02E-02
OCIAD2	598.14	2.66	0.87	3.71E-05	1.21E-03
PODNL1	2008.76	2.66	0.62	4.79E-07	4.45E-05
SELL	535.46	2.64	1.30	4.54E-04	7.59E-03
ST6GAL2	2365.09	2.63	1.09	2.16E-04	4.45E-03
MCTP2	953.46	2.63	0.73	7.09E-06	3.54E-04
CXCL5	107.77	2.61	1.12	2.76E-04	5.36E-03
RAC3	43.33	2.61	1.22	4.56E-04	7.60E-03
TNFRSF18	105.54	2.61	1.28	4.86E-04	7.92E-03
COL26A1	283.75	2.60	1.30	5.05E-04	8.10E-03
ENC1	2490.33	2.59	0.91	7.07E-05	1.99E-03
NAT8L	9.15	2.59	1.49	2.01E-03	2.14E-02
IQCA1	688.06	2.59	0.84	3.60E-05	1.19E-03
CNIH2	38.22	2.57	1.26	5.96E-04	9.06E-03
B3GALT2	70.74	2.55	0.97	1.60E-04	3.57E-03
PCDH7	7153.62	2.54	0.76	1.62E-05	6.48E-04
CRABP2	31273.44	2.51	0.65	2.83E-06	1.76E-04
DGKI	538.88	2.50	0.70	8.67E-06	4.12E-04
IL4I1	571.39	2.49	1.28	5.78E-04	8.94E-03
CLNK	46.00	2.48	1.34	8.13E-04	1.13E-02
COMP	45407.23	2.45	0.95	1.66E-04	3.68E-03
PNPLA3	39.50	2.43	0.71	1.82E-05	7.06E-04
GALNT5	2486.19	2.41	1.24	6.00E-04	9.08E-03
P4HA3	6227.88	2.40	1.06	3.25E-04	6.00E-03
NEFH	205.10	2.39	1.07	3.81E-04	6.71E-03
SPARC	1797901.02	2.38	0.58	9.56E-07	7.63E-05
LCTL	139.68	2.38	0.86	1.11E-04	2.69E-03
SLN	151.92	2.38	1.05	3.59E-04	6.44E-03
MEX3A	113.75	2.38	0.66	8.68E-06	4.12E-04
COL5A1	112579.09	2.37	0.73	2.54E-05	9.20E-04
FSCN1	9092.52	2.35	0.55	4.65E-07	4.40E-05
F2R	11380.17	2.33	0.88	1.51E-04	3.42E-03
CLEC11A	12166.07	2.30	0.43	3.71E-09	8.36E-07
FAM151B	222.71	2.28	0.44	6.60E-09	1.35E-06
PYCR1	7232.42	2.27	0.78	7.61E-05	2.09E-03
KIAA2012	112.80	2.26	0.29	9.12E-17	1.55E-13
CCDC102B	4013.39	2.26	0.79	8.54E-05	2.22E-03
PHEX	166.87	2.25	0.83	1.37E-04	3.15E-03

VSNL1	926.75	2.24	1.09	5.55E-04	8.68E-03
SFT2D3	331.90	2.24	0.81	1.18E-04	2.81E-03
KCNAB3	84.31	2.24	1.01	4.76E-04	7.81E-03
CDC25C	43.30	2.21	1.21	9.58E-04	1.27E-02
RRM2	852.94	2.20	1.26	9.37E-04	1.25E-02
KIF4A	199.80	2.19	0.98	4.23E-04	7.21E-03
CDCA3	242.50	2.19	0.55	1.86E-06	1.29E-04
PKP2	2108.08	2.19	0.92	3.00E-04	5.66E-03
ZNF469	1961.94	2.19	0.52	8.03E-07	6.77E-05
ARL9	70.98	2.18	0.71	5.41E-05	1.61E-03
LIMD2	1381.64	2.17	0.85	2.00E-04	4.26E-03
CEMIP	475.12	2.16	1.07	6.41E-04	9.54E-03
PRAG1	142.53	2.15	1.18	9.21E-04	1.24E-02
GYG2	282.45	2.14	0.55	2.71E-06	1.72E-04
PSD	1025.14	2.14	0.71	6.12E-05	1.78E-03
GRIA3	1211.17	2.12	1.07	6.80E-04	1.00E-02
RMI2	462.80	2.11	0.33	9.57E-12	5.24E-09
DNAH14	203.64	2.10	0.74	1.08E-04	2.66E-03
SERINC5	2915.57	2.10	0.59	1.09E-05	4.89E-04
DNAJC12	572.21	2.10	0.49	5.02E-07	4.59E-05
FGD6	1046.63	2.09	0.52	1.75E-06	1.25E-04
KIF2C	228.16	2.08	0.89	3.78E-04	6.69E-03
OSBPL3	1513.19	2.07	0.50	1.11E-06	8.67E-05
QPRT	1386.28	2.06	0.68	5.81E-05	1.70E-03
TRIM59	423.24	2.06	0.60	1.68E-05	6.68E-04
SPON1	41874.28	2.05	0.97	5.78E-04	8.94E-03
LXN	9088.58	2.05	0.91	4.72E-04	7.77E-03
B4GALNT3	253.77	2.04	1.15	1.06E-03	1.37E-02
ANXA13	47.26	2.04	1.11	1.09E-03	1.40E-02
CBX8	384.30	2.03	0.65	4.87E-05	1.49E-03
KCNK6	1237.14	2.02	0.62	2.88E-05	1.01E-03
PCLAF	322.89	2.00	1.03	8.38E-04	1.15E-02
ENTPD7	847.10	2.00	0.57	1.21E-05	5.23E-04
CSMD2	843.48	1.99	0.51	2.90E-06	1.80E-04
SMCO2	12.42	1.99	1.35	2.53E-03	2.53E-02
COL5A2	74425.44	1.99	0.74	1.66E-04	3.68E-03
SLC17A9	1249.57	1.96	0.55	1.14E-05	5.00E-04
KIAA1211	157.85	1.94	1.06	1.06E-03	1.37E-02
PRR7	316.22	1.92	0.55	1.47E-05	6.03E-04
PTGER1	187.99	1.91	0.93	7.58E-04	1.07E-02
PKIA	2153.68	1.91	0.44	4.17E-07	4.13E-05
CDKN2A	1045.67	1.90	1.04	1.04E-03	1.36E-02
NRK	1191.07	1.90	0.69	1.53E-04	3.45E-03
CCNE1	207.37	1.88	0.55	2.06E-05	7.78E-04
DEPDC7	447.07	1.88	0.42	2.53E-07	2.92E-05
BMF	633.34	1.88	0.32	2.51E-10	7.71E-08
SSX2IP	1299.68	1.87	0.37	1.31E-08	2.38E-06
UNC5B	4372.79	1.86	0.50	5.85E-06	3.03E-04
PLK4	202.52	1.85	0.50	6.81E-06	3.41E-04
HMGB3	569.31	1.84	0.81	5.03E-04	8.08E-03
RHBDL2	163.95	1.84	0.58	4.79E-05	1.47E-03
NRCAM	168.84	1.84	1.01	1.19E-03	1.48E-02
NFE2	70.98	1.83	1.54	2.38E-03	2.42E-02
FAP	14186.15	1.83	0.70	2.26E-04	4.60E-03
TTYH3	5373.07	1.83	0.63	1.05E-04	2.59E-03
MICB	172.98	1.82	0.78	4.75E-04	7.81E-03
C1QTNF12	139.85	1.81	1.29	1.99E-03	2.13E-02
KDELR3	13867.14	1.80	0.77	4.25E-04	7.22E-03
LRRC8E	560.49	1.80	0.95	1.08E-03	1.39E-02
ZNF474	46.56	1.79	0.80	6.71E-04	9.95E-03
TRIB2	3829.80	1.79	0.56	4.59E-05	1.43E-03
ST6GALNAC5	344.90	1.79	0.97	1.21E-03	1.50E-02
PLOD2	16341.55	1.78	0.89	8.99E-04	1.22E-02
SEMA5A	4900.60	1.77	0.31	5.45E-10	1.49E-07
NUSAP1	645.71	1.77	1.02	1.40E-03	1.66E-02
GNG2	6122.31	1.76	0.65	1.97E-04	4.22E-03
NREP	14634.97	1.76	0.52	2.12E-05	7.98E-04
MMP15	352.93	1.76	0.66	2.08E-04	4.38E-03
SH3BP1	241.10	1.76	0.66	2.20E-04	4.51E-03
RNF175	236.95	1.74	0.95	1.28E-03	1.56E-02
BTBD11	235.30	1.74	0.56	6.63E-05	1.90E-03
KIF11	408.19	1.74	0.55	4.86E-05	1.49E-03
KCNG1	157.55	1.72	0.99	1.50E-03	1.75E-02
SLC16A3	534.54	1.71	0.37	1.92E-07	2.27E-05
ACSM3	173.60	1.71	0.35	3.94E-08	5.93E-06
SEZ6	38.07	1.71	0.47	1.28E-05	5.47E-04
DEPDC1	215.16	1.69	1.27	2.38E-03	2.42E-02
GPR137C	175.23	1.69	0.56	8.95E-05	2.30E-03
PARPBP	482.31	1.68	0.36	1.15E-07	1.45E-05
ARMC9	2667.59	1.68	0.56	9.41E-05	2.39E-03
MXRA5	35898.30	1.67	0.77	5.89E-04	9.03E-03
MMP16	4071.84	1.66	1.00	1.69E-03	1.89E-02
SYNDIG1	652.42	1.66	0.78	8.10E-04	1.13E-02
SLC1A1	734.10	1.66	0.58	1.37E-04	3.15E-03
PLEKHG4B	70.97	1.66	1.32	2.77E-03	2.66E-02
PLK1	295.88	1.65	1.04	1.94E-03	2.09E-02
ALCAM	6972.62	1.64	0.65	3.47E-04	6.31E-03
C9orf84	22.17	1.64	0.59	2.52E-04	5.02E-03
TMTC2	367.50	1.62	1.06	2.11E-03	2.21E-02
RAB40A	86.33	1.62	0.97	1.88E-03	2.05E-02
SMC1B	6.75	1.62	1.24	5.68E-03	4.36E-02
PRPH2	676.87	1.62	0.67	4.57E-04	7.61E-03
TMEM154	589.27	1.61	0.53	8.28E-05	2.19E-03
FNDC10	1304.43	1.61	0.76	8.63E-04	1.18E-02
GPR162	233.15	1.60	0.60	2.50E-04	4.99E-03
SMC4	3917.28	1.59	0.29	1.17E-09	2.93E-07
GRK3	1319.29	1.58	0.93	1.75E-03	1.95E-02
PLPPR4	2029.95	1.57	0.74	8.99E-04	1.22E-02
ABCC4	977.90	1.56	0.74	9.21E-04	1.24E-02
BMP8A	61.74	1.55	0.80	1.42E-03	1.67E-02
TLR9	20.19	1.55	1.00	2.81E-03	2.68E-02
BRCA2	486.17	1.55	0.52	1.10E-04	2.69E-03

DCBLD1	1587.78	1.55	0.29	7.95E-09	1.56E-06
OTUB2	144.94	1.54	0.54	1.57E-04	3.52E-03
RAD54L	86.89	1.52	1.59	3.57E-03	3.17E-02
HTRA4	68.17	1.51	0.53	1.71E-04	3.75E-03
DIRAS3	692.89	1.51	1.17	2.95E-03	2.77E-02
FAM171A2	726.46	1.51	0.79	1.42E-03	1.67E-02
SHCBP1	326.72	1.51	1.29	3.23E-03	2.95E-02
BCL11A	62.86	1.50	1.22	3.38E-03	3.04E-02
SULF2	22441.74	1.50	0.62	4.83E-04	7.90E-03
TMEM200A	2807.82	1.50	0.62	5.09E-04	8.13E-03
CDH11	24057.44	1.50	0.69	7.50E-04	1.07E-02
CHRNE	143.54	1.50	0.73	1.19E-03	1.49E-02
TNFRSF6B	1045.82	1.49	1.47	3.44E-03	3.07E-02
ARHGAP28	4829.74	1.49	1.00	2.54E-03	2.54E-02
HAGHL	353.04	1.49	0.40	8.24E-06	3.98E-04
VDR	1561.58	1.49	0.66	7.50E-04	1.07E-02
ZNF608	538.82	1.48	0.79	1.56E-03	1.79E-02
STK17B	5869.09	1.47	0.79	1.53E-03	1.77E-02
CASP1	1034.93	1.46	0.58	4.11E-04	7.06E-03
GXYLT2	13456.10	1.46	0.99	2.67E-03	2.62E-02
FKBP11	4415.41	1.46	0.38	6.53E-06	3.32E-04
CFAP206	18.53	1.46	1.51	4.65E-03	3.82E-02
PANO1	37.95	1.45	0.73	1.51E-03	1.75E-02
RAD51	235.78	1.45	1.00	2.85E-03	2.71E-02
XPR1	2360.09	1.43	0.97	2.78E-03	2.67E-02
OSTC	15442.18	1.43	0.38	9.59E-06	4.48E-04
SMCO4	1435.74	1.42	0.28	2.49E-08	4.11E-06
TLCD1	203.51	1.42	1.06	3.30E-03	3.00E-02
CENPK	313.23	1.42	1.00	3.05E-03	2.84E-02
CACNA1D	180.91	1.41	0.54	3.67E-04	6.54E-03
P2RY1	909.16	1.40	0.45	7.80E-05	2.11E-03
LAYN	2181.04	1.40	0.50	2.07E-04	4.37E-03
C4orf48	897.12	1.39	0.55	4.64E-04	7.70E-03
C1orf74	253.29	1.39	0.68	1.31E-03	1.58E-02
TLR6	140.25	1.39	1.07	3.58E-03	3.17E-02
ACTR3C	144.30	1.39	0.56	5.02E-04	8.08E-03
ZNF730	58.66	1.39	0.63	1.05E-03	1.37E-02
TANC2	2097.31	1.38	0.24	3.07E-10	9.05E-08
ADAMTS12	1897.36	1.38	0.59	6.73E-04	9.96E-03
MAGEL2	466.23	1.38	1.02	3.40E-03	3.05E-02
COL8A1	17566.18	1.38	0.60	7.14E-04	1.04E-02
ABHD17C	469.67	1.38	0.48	1.88E-04	4.07E-03
ZNF93	495.20	1.38	0.36	6.23E-06	3.20E-04
DIAPH3	288.05	1.38	1.35	4.09E-03	3.50E-02
GBP3	2484.56	1.37	0.33	2.09E-06	1.41E-04
GRAMD1B	201.30	1.37	1.14	3.88E-03	3.36E-02
GRIK2	155.41	1.37	0.77	2.19E-03	2.27E-02
HENMT1	768.90	1.37	0.31	5.41E-07	4.79E-05
SERPINH1	55253.57	1.37	0.49	2.17E-04	4.45E-03
SRGAP3	970.55	1.37	0.48	2.08E-04	4.38E-03
ENPP2	9052.02	1.36	1.35	4.09E-03	3.49E-02
CENPE	275.17	1.35	0.78	2.34E-03	2.39E-02
BANK1	125.87	1.34	1.19	4.22E-03	3.56E-02
ARHGAP22	1300.81	1.34	0.91	3.17E-03	2.91E-02
TUSC3	3762.48	1.34	0.55	5.91E-04	9.05E-03
ZKSCAN4	485.01	1.33	0.43	1.02E-04	2.55E-03
EPHB2	3742.10	1.33	0.56	6.54E-04	9.72E-03
SERPINE2	13321.12	1.32	0.92	3.31E-03	3.00E-02
PTGER3	3173.17	1.32	0.83	2.77E-03	2.67E-02
TIGD2	358.78	1.32	0.38	3.20E-05	1.11E-03
FZD2	504.43	1.31	0.64	1.43E-03	1.68E-02
ORAI2	3937.47	1.31	0.39	3.81E-05	1.24E-03
BICDL2	49.30	1.30	0.53	6.78E-04	1.00E-02
ADGRL1	870.62	1.30	0.52	5.38E-04	8.47E-03
FKBP10	3333.20	1.28	0.83	3.19E-03	2.92E-02
COBL	67.70	1.27	0.59	1.27E-03	1.56E-02
RIPK3	601.82	1.27	0.71	2.40E-03	2.44E-02
STIL	203.91	1.27	0.70	2.34E-03	2.39E-02
ZNF697	359.65	1.27	0.21	5.11E-11	1.97E-08
P3H4	7350.05	1.26	0.66	1.89E-03	2.05E-02
C4orf47	154.93	1.26	1.08	4.69E-03	3.85E-02
POMC	202.41	1.26	0.80	3.33E-03	3.02E-02
FASTKD1	1073.13	1.25	0.41	1.04E-04	2.58E-03
CCDC184	175.83	1.25	0.94	4.25E-03	3.58E-02
CTHRC1	42439.03	1.25	1.07	4.55E-03	3.77E-02
MAD2L1	573.57	1.25	0.62	1.62E-03	1.84E-02
LAMB4	38.49	1.25	0.63	2.07E-03	2.19E-02
MAPK12	1386.45	1.25	1.07	4.64E-03	3.82E-02
LRRC61	777.38	1.25	0.66	2.08E-03	2.20E-02
SULF1	25454.90	1.25	0.63	1.74E-03	1.94E-02
OXSM	626.76	1.24	0.43	2.09E-04	4.38E-03
CARD14	61.26	1.24	0.55	1.11E-03	1.42E-02
C1orf53	81.09	1.24	0.72	2.93E-03	2.77E-02
ZFP69B	156.75	1.23	0.71	2.81E-03	2.68E-02
FANCF	735.78	1.23	0.19	1.21E-11	6.39E-09
RTP4	288.57	1.22	0.60	1.69E-03	1.89E-02
RIMBP2	692.06	1.22	1.22	5.28E-03	4.18E-02
IGF1	23223.49	1.21	0.97	4.66E-03	3.83E-02
RHOBTB1	2538.76	1.20	0.30	5.17E-06	2.79E-04
MAGED1	31746.77	1.20	0.58	1.55E-03	1.78E-02
CKAP2	1779.46	1.20	0.46	4.98E-04	8.06E-03
FXYD5	4527.18	1.20	0.68	2.71E-03	2.63E-02
PLOD1	18377.83	1.20	0.26	2.34E-07	2.74E-05
GREM1	3604.42	1.19	1.21	5.73E-03	4.38E-02
ANKRD2	109.77	1.19	0.57	1.68E-03	1.88E-02
P3H1	16501.19	1.19	0.59	1.80E-03	1.98E-02
LMO7	1253.40	1.18	0.45	4.19E-04	7.17E-03
SOX12	1054.18	1.18	0.43	3.44E-04	6.28E-03
FBXO4	1186.17	1.18	0.24	6.03E-08	8.49E-06
CPPED1	3277.26	1.18	0.50	9.08E-04	1.22E-02
UBE2T	344.48	1.18	1.07	5.47E-03	4.26E-02
RCC1	763.99	1.17	0.42	2.86E-04	5.46E-03

GRM6	28.28	1.17	0.64	3.06E-03	2.84E-02
DNAJC15	4046.76	1.17	0.37	9.16E-05	2.34E-03
MIS18BP1	3246.00	1.16	0.59	2.13E-03	2.23E-02
VANGL2	455.43	1.15	0.63	2.67E-03	2.62E-02
EPHB3	4209.14	1.15	1.00	5.45E-03	4.25E-02
N4BP2	1891.00	1.15	0.37	1.10E-04	2.69E-03
HSD11B2	1637.41	1.15	1.57	5.92E-03	4.49E-02
IDH2	8016.33	1.15	0.52	1.22E-03	1.51E-02
PLPP2	166.48	1.15	1.12	6.02E-03	4.55E-02
SCD	835.04	1.15	0.43	4.68E-04	7.73E-03
CAPG	7423.12	1.15	0.43	4.38E-04	7.36E-03
TRIM16	1617.51	1.14	0.47	8.27E-04	1.14E-02
CCDC28B	1403.80	1.14	0.35	6.83E-05	1.94E-03
HSBP1L1	672.94	1.14	0.56	2.00E-03	2.14E-02
IL1RAP	1759.76	1.14	0.52	1.45E-03	1.70E-02
SGO2	717.70	1.14	0.74	4.16E-03	3.52E-02
AIPL1	18.82	1.13	0.81	6.14E-03	4.61E-02
RAP2B	3741.18	1.13	0.40	2.85E-04	5.46E-03
FERMT1	214.16	1.13	0.95	5.86E-03	4.46E-02
SPATA17	78.80	1.13	0.76	4.78E-03	3.90E-02
B4GALNT4	77.25	1.13	1.53	6.32E-03	4.71E-02
RBM43	728.47	1.13	0.42	4.35E-04	7.35E-03
MCEE	2187.16	1.12	0.13	3.19E-18	9.80E-15
ZNF888	246.58	1.12	0.38	2.31E-04	4.68E-03
KCTD15	850.69	1.12	0.37	1.47E-04	3.34E-03
CASC10	412.98	1.12	0.79	4.89E-03	3.96E-02
PTK7	9053.38	1.12	0.98	5.90E-03	4.49E-02
RMI1	623.54	1.12	0.33	5.35E-05	1.61E-03
NHLRC1	264.05	1.12	0.42	4.93E-04	7.99E-03
ANLN	775.28	1.12	1.62	6.20E-03	4.64E-02
DAPK1	3413.51	1.11	0.67	3.70E-03	3.26E-02
CENPF	588.09	1.11	0.87	5.54E-03	4.29E-02
FBN2	236.17	1.11	1.37	6.42E-03	4.75E-02
MFAP2	20659.07	1.11	0.64	3.41E-03	3.06E-02
MORN3	85.85	1.11	0.50	1.51E-03	1.75E-02
CHST2	1418.60	1.10	0.91	5.93E-03	4.50E-02
CTSB	78372.91	1.10	0.30	1.49E-05	6.09E-04
SNX8	1099.60	1.10	0.47	1.03E-03	1.34E-02
PRUNE2	2488.18	1.10	0.66	3.80E-03	3.32E-02
CRMP1	1201.08	1.10	1.04	6.34E-03	4.71E-02
FKBP14	7910.41	1.10	0.80	5.31E-03	4.19E-02
RTN4RL2	1177.41	1.10	0.41	4.64E-04	7.70E-03
SCD5	1737.91	1.10	0.46	9.48E-04	1.27E-02
SLC2A9	293.22	1.09	0.72	4.80E-03	3.91E-02
PHTF2	4137.25	1.09	0.31	3.48E-05	1.16E-03
TRPM8	11.98	1.08	1.09	8.99E-03	5.95E-02
SAMD9	1791.89	1.08	0.28	9.82E-06	4.54E-04
AP1S1	546.97	1.08	0.68	4.29E-03	3.60E-02
MAGED4B	2310.29	1.08	0.36	1.81E-04	3.95E-03
TICAM2	2174.20	1.08	0.45	1.01E-03	1.33E-02
TPMT	2008.27	1.08	0.29	1.78E-05	6.98E-04
ZNF85	497.90	1.08	0.28	1.15E-05	5.00E-04
SLC9A5	405.86	1.08	0.63	3.83E-03	3.33E-02
VWA7	42.18	1.07	0.95	7.27E-03	5.18E-02
ZKSCAN7	596.59	1.07	0.37	2.91E-04	5.56E-03
PAFAH1B3	571.28	1.06	0.52	2.19E-03	2.27E-02
SLC29A4	589.77	1.06	0.73	5.38E-03	4.22E-02
SPDL1	790.45	1.06	0.23	3.23E-07	3.44E-05
RAB31	14448.41	1.05	0.30	3.61E-05	1.19E-03
LSAMP	2350.33	1.05	0.71	5.34E-03	4.20E-02
LHFPL2	11315.79	1.05	0.62	3.99E-03	3.44E-02
SPAG4	199.94	1.05	1.07	7.28E-03	5.18E-02
SSC4D	80.87	1.05	0.43	1.01E-03	1.33E-02
DNAJB7	9.22	1.05	1.23	1.04E-02	6.53E-02
KCNE3	506.89	1.04	0.41	6.99E-04	1.02E-02
SLC7A7	1077.86	1.04	2.01	6.29E-03	4.69E-02
CCDC96	135.65	1.04	0.42	9.65E-04	1.28E-02
PABPC4L	447.59	1.04	0.44	1.15E-03	1.44E-02
SLC38A6	2175.97	1.04	0.32	8.41E-05	2.22E-03
NAT14	746.43	1.04	0.32	8.45E-05	2.22E-03
EFNA4	526.30	1.03	0.45	1.39E-03	1.65E-02
HELLS	439.34	1.03	0.50	2.30E-03	2.36E-02
LRRC19	19.95	1.03	0.81	8.01E-03	5.53E-02
PPIB	52039.63	1.03	0.33	1.25E-04	2.91E-03
SLC16A1	10493.86	1.03	0.77	6.29E-03	4.69E-02
DHFR	515.76	1.03	0.76	6.29E-03	4.69E-02
AKR7L	28.92	1.02	0.49	2.59E-03	2.57E-02
RAD51AP1	231.62	1.02	1.03	7.77E-03	5.42E-02
SLFN12	828.45	1.01	0.55	3.32E-03	3.01E-02
AGRN	1301.23	1.01	0.35	2.80E-04	5.39E-03
ZNF587B	304.65	1.01	0.34	2.15E-04	4.45E-03
AKAP5	56.05	1.01	0.37	5.08E-04	8.12E-03
ZMYND12	122.20	1.01	0.79	7.17E-03	5.13E-02
FSBP	152.78	1.01	0.51	2.88E-03	2.72E-02
GPX7	3941.30	1.01	0.71	6.03E-03	4.55E-02
OSBPL7	902.54	1.01	0.40	7.48E-04	1.07E-02
GLIPR1	7304.40	1.01	0.46	1.62E-03	1.84E-02
GINS4	112.56	1.01	0.50	2.57E-03	2.55E-02
PDIA5	5512.79	1.01	0.59	4.23E-03	3.57E-02
NMI	2207.90	1.01	0.28	2.70E-05	9.65E-04
TP53INP1	5488.53	1.01	0.45	1.62E-03	1.84E-02
SLC7A11	46.67	1.01	0.98	8.43E-03	5.70E-02
BRIP1	110.70	1.00	0.65	5.73E-03	4.38E-02
SLC39A7	4931.36	1.00	0.76	6.96E-03	5.02E-02
FAAP24	253.94	1.00	0.34	2.98E-04	5.64E-03
KDELC1	2664.37	1.00	0.42	1.29E-03	1.57E-02
TRIM6	834.28	0.99	0.78	7.24E-03	5.17E-02
TMEM132E	157.13	0.99	0.55	4.12E-03	3.51E-02
HMGN1	12524.41	0.99	0.15	2.18E-12	1.45E-09
PGM2L1	1537.28	0.99	0.34	2.84E-04	5.45E-03
RGS14	652.64	0.99	0.81	7.61E-03	5.36E-02
CCT6B	204.28	0.99	0.35	3.75E-04	6.65E-03

ARHGEF19	924.97	0.99	0.58	4.59E-03	3.79E-02
HS2ST1	2811.32	0.98	0.36	5.06E-04	8.10E-03
TGFB1	3572.94	0.98	0.42	1.42E-03	1.67E-02
C17orf107	516.26	0.98	0.53	3.76E-03	3.30E-02
IFT20	3167.41	0.98	0.24	4.70E-06	2.59E-04
IL15	426.23	0.98	1.04	8.43E-03	5.70E-02
MMP14	4532.85	0.98	0.74	7.28E-03	5.18E-02
TTC30B	468.44	0.98	0.37	6.74E-04	9.96E-03
XKR8	1087.71	0.97	0.27	3.05E-05	1.06E-03
SPATS2	2129.45	0.97	0.19	3.11E-08	4.96E-06
TBC1D8B	3527.27	0.97	0.41	1.38E-03	1.65E-02
TMEM177	433.05	0.97	0.34	4.20E-04	7.18E-03
CASP8	1365.81	0.97	0.41	1.38E-03	1.65E-02
BCAS1	54.13	0.97	0.62	6.20E-03	4.64E-02
ZNF860	75.73	0.96	0.58	5.46E-03	4.25E-02
ICAM3	767.47	0.96	0.36	6.27E-04	9.37E-03
GOLM1	12882.41	0.96	0.60	5.53E-03	4.29E-02
UAP1L1	3410.52	0.96	0.29	8.58E-05	2.23E-03
STK32C	956.11	0.95	0.39	1.18E-03	1.48E-02
CYP2R1	748.16	0.95	0.24	8.26E-06	3.98E-04
ZDHHC20	4603.30	0.95	0.57	5.44E-03	4.25E-02
CMTM4	2084.40	0.95	0.63	6.65E-03	4.86E-02
CASR	43.24	0.94	0.86	9.58E-03	6.19E-02
SAMD13	129.80	0.94	0.45	2.77E-03	2.67E-02
WNK3	148.98	0.94	0.59	6.10E-03	4.59E-02
OR2L2	63.38	0.94	0.51	4.25E-03	3.58E-02
TGFB3	11255.01	0.94	0.46	2.95E-03	2.78E-02
IL11	50.87	0.94	0.68	8.22E-03	5.63E-02
OSTF1	3531.28	0.94	0.43	2.03E-03	2.16E-02
B4GALT6	220.71	0.94	0.45	2.75E-03	2.66E-02
ACTR2	16092.55	0.94	0.46	2.63E-03	2.59E-02
CERCAM	42367.41	0.94	0.84	8.57E-03	5.77E-02
SYT7	247.37	0.94	0.79	8.93E-03	5.92E-02
CGNL1	806.51	0.94	0.47	3.21E-03	2.93E-02
THY1	71863.18	0.93	0.79	8.77E-03	5.85E-02
NUDT14	616.38	0.93	0.77	8.79E-03	5.86E-02
RASD2	334.00	0.93	0.58	6.03E-03	4.55E-02
SYT5	28.84	0.93	0.57	6.71E-03	4.89E-02
RCN1	26724.66	0.93	0.52	4.50E-03	3.74E-02
MDK	13087.63	0.93	0.74	8.65E-03	5.81E-02
PYCARD	1446.12	0.93	0.65	7.63E-03	5.36E-02
MLKL	1180.91	0.93	0.68	8.02E-03	5.53E-02
PHTF1	2397.94	0.93	0.42	2.21E-03	2.29E-02
ZNF20	74.59	0.92	0.67	8.31E-03	5.64E-02
CYB5R2	1171.41	0.92	0.73	8.73E-03	5.83E-02
RAB7B	4482.33	0.92	0.62	7.16E-03	5.12E-02
ORMDL3	3287.00	0.92	0.49	4.04E-03	3.47E-02
RYR1	241.40	0.92	0.57	6.34E-03	4.71E-02
PRPF19	569.31	0.91	0.29	1.53E-04	3.45E-03
SLC16A14	296.50	0.91	0.35	7.60E-04	1.07E-02
SLX4IP	601.86	0.91	0.26	5.40E-05	1.61E-03
TBC1D3B	48.11	0.91	0.69	9.20E-03	6.04E-02
FNBP1L	3696.37	0.91	0.22	4.02E-06	2.34E-04
RACGAP1	1078.69	0.91	0.70	8.88E-03	5.91E-02
PAQR6	36.52	0.91	0.62	8.27E-03	5.64E-02
TRPS1	3063.60	0.90	0.44	3.14E-03	2.89E-02
GALM	2427.89	0.90	0.24	1.57E-05	6.33E-04
CDH24	389.31	0.90	0.77	9.97E-03	6.36E-02
BET1	3828.28	0.90	0.24	1.84E-05	7.14E-04
ZNF555	621.61	0.89	0.42	2.80E-03	2.68E-02
CLN6	400.58	0.89	0.35	9.84E-04	1.30E-02
GPX8	13109.72	0.89	0.74	9.48E-03	6.15E-02
TNIK	683.67	0.89	0.49	4.96E-03	3.98E-02
TMED3	8119.15	0.89	0.36	1.13E-03	1.43E-02
CDC42EP5	2947.33	0.89	0.24	2.77E-05	9.83E-04
SLC35B3	3300.35	0.89	0.37	1.46E-03	1.70E-02
BORA	432.00	0.89	0.44	3.44E-03	3.07E-02
ZNF675	776.39	0.88	0.28	1.75E-04	3.84E-03
CYBA	12977.62	0.88	0.77	1.03E-02	6.48E-02
SRD5A3	2319.52	0.88	0.30	3.20E-04	5.93E-03
SOCS5	6208.10	0.88	0.48	4.90E-03	3.96E-02
DISC1	459.76	0.88	1.04	1.07E-02	6.62E-02
OIP5	97.37	0.88	0.62	9.13E-03	6.00E-02
NUDT19	527.90	0.88	0.50	5.66E-03	4.35E-02
CCDC170	235.05	0.88	0.74	1.05E-02	6.56E-02
DNASE1L1	1233.35	0.87	0.42	3.03E-03	2.83E-02
ZNF792	275.55	0.87	0.60	8.60E-03	5.79E-02
FAM214B	2306.27	0.87	0.26	7.96E-05	2.14E-03
ZNF155	459.61	0.87	0.24	3.30E-05	1.12E-03
RPGRIP1L	1226.39	0.87	0.55	7.30E-03	5.19E-02
ARID3A	373.55	0.87	0.38	2.15E-03	2.24E-02
SLC25A15	396.41	0.87	0.33	8.23E-04	1.14E-02
PAPSS1	7829.06	0.87	0.24	2.95E-05	1.03E-03
CREB3L1	12122.77	0.87	0.97	1.10E-02	6.76E-02
CYB561D2	1690.68	0.86	0.23	1.97E-05	7.56E-04
RASSF2	4468.19	0.86	0.69	1.02E-02	6.45E-02
DNAJC28	158.04	0.86	0.48	5.44E-03	4.25E-02
FTL	327933.32	0.86	0.32	1.01E-03	1.33E-02
GEN1	485.05	0.86	0.48	5.38E-03	4.22E-02
TMEM60	1935.66	0.86	0.25	7.85E-05	2.12E-03
PIF1	254.03	0.86	0.52	6.86E-03	4.98E-02
RCN3	31857.08	0.86	0.86	1.13E-02	6.89E-02
SRPRB	5192.61	0.86	0.46	5.01E-03	4.01E-02
CERS6	3726.14	0.86	0.32	7.11E-04	1.03E-02
STIM2	2520.74	0.86	0.34	1.13E-03	1.43E-02
PPP2R3A	2585.62	0.86	0.21	6.56E-06	3.32E-04
RAB42	713.11	0.86	0.85	1.16E-02	6.98E-02
PGM3	7045.22	0.86	0.36	1.73E-03	1.93E-02
DCBLD2	11307.81	0.85	0.76	1.10E-02	6.74E-02
FANCI	587.39	0.85	0.61	9.34E-03	6.09E-02
EVL	1559.47	0.85	0.26	1.23E-04	2.88E-03
PPP1R36	59.94	0.85	0.96	1.22E-02	7.22E-02

MAP10	249.81	0.85	0.28	2.60E-04	5.12E-03
SSR1	21450.18	0.84	0.31	6.51E-04	9.68E-03
ZNF530	209.28	0.84	0.20	5.04E-06	2.75E-04
MGAT3	121.34	0.84	1.30	1.05E-02	6.56E-02
DSN1	1449.73	0.84	0.21	6.76E-06	3.40E-04
C9orf116	190.94	0.84	0.28	3.27E-04	6.02E-03
ZNF737	650.17	0.84	0.43	4.36E-03	3.66E-02
CCNB1	742.20	0.84	0.69	1.14E-02	6.92E-02
ARV1	1363.46	0.84	0.19	2.13E-06	1.43E-04
GORAB	1676.20	0.84	0.18	2.78E-07	3.18E-05
IL18R1	438.48	0.84	1.10	1.14E-02	6.92E-02
IKBIP	4799.05	0.84	0.58	9.46E-03	6.15E-02
GTF2E1	1128.34	0.83	0.31	7.99E-04	1.11E-02
PEX11G	417.10	0.83	0.27	2.78E-04	5.38E-03
ZNF43	1519.53	0.83	0.34	1.41E-03	1.66E-02
TCEAL8	7286.76	0.83	0.27	2.63E-04	5.17E-03
SPATS2L	13263.84	0.83	0.33	1.26E-03	1.54E-02
BMP1	15662.46	0.83	0.66	1.10E-02	6.76E-02
SLC39A10	3970.83	0.83	0.34	1.61E-03	1.83E-02
SUV39H2	466.03	0.83	0.35	1.95E-03	2.10E-02
RIDA	1017.48	0.83	0.18	3.96E-07	3.94E-05
PTRHD1	1107.85	0.83	0.31	9.11E-04	1.22E-02
SHB	1246.78	0.83	0.47	6.35E-03	4.71E-02
SESN3	2834.75	0.83	0.28	3.93E-04	6.86E-03
WDR5B	720.34	0.83	0.44	5.27E-03	4.17E-02
KIF15	229.89	0.82	0.85	1.29E-02	7.50E-02
ATM	2807.99	0.82	0.18	7.46E-07	6.39E-05
PRR11	188.92	0.82	0.63	1.17E-02	7.02E-02
BBS12	500.90	0.82	0.54	9.35E-03	6.10E-02
H2AFY2	1318.09	0.82	0.25	1.22E-04	2.87E-03
AGA	1694.62	0.82	0.30	7.45E-04	1.07E-02
C3orf14	1022.64	0.82	0.32	1.22E-03	1.51E-02
RIN1	1011.17	0.82	0.21	1.36E-05	5.74E-04
MFSD3	690.71	0.81	0.26	2.70E-04	5.27E-03
TCN2	4914.17	0.81	0.44	5.49E-03	4.27E-02
LPAR2	225.43	0.81	0.65	1.22E-02	7.22E-02
LZTS1	1114.87	0.81	0.64	1.20E-02	7.16E-02
ARHGAP11A	252.70	0.81	0.94	1.30E-02	7.53E-02
ZNF701	805.00	0.81	0.25	1.34E-04	3.10E-03
UBE2E1	708.29	0.81	0.49	8.22E-03	5.63E-02
TST	795.71	0.81	0.57	1.07E-02	6.62E-02
WDHD1	525.37	0.81	0.24	1.28E-04	2.96E-03
TBC1D7	1422.51	0.80	0.34	2.11E-03	2.21E-02
SYCE1L	158.64	0.80	0.79	1.37E-02	7.87E-02
SEC11A	7061.35	0.80	0.19	5.45E-06	2.89E-04
SSR3	13440.72	0.80	0.43	5.28E-03	4.18E-02
PTPA	2634.41	0.80	0.40	4.54E-03	3.76E-02
PRDX4	14637.55	0.80	0.55	1.05E-02	6.56E-02
MYL5	479.33	0.80	0.44	6.37E-03	4.72E-02
ABCC9	5531.98	0.80	0.94	1.32E-02	7.60E-02
RNASEH2B	3915.10	0.80	0.15	7.53E-09	1.52E-06
KLHL35	271.72	0.80	0.64	1.30E-02	7.52E-02
PDXK	7036.40	0.79	0.20	1.26E-05	5.40E-04
DNAJC13	4080.83	0.79	0.32	1.69E-03	1.89E-02
PPP1R8	1412.76	0.79	0.46	7.31E-03	5.19E-02
ACY1	1337.11	0.79	0.24	1.47E-04	3.34E-03
LRP2BP	234.87	0.79	0.80	1.43E-02	8.08E-02
GALNT1	11013.35	0.79	0.29	8.94E-04	1.22E-02
SLC18B1	1716.34	0.79	0.46	7.90E-03	5.48E-02
ABRACL	1569.49	0.79	1.70	8.90E-03	5.91E-02
CHSY3	1112.87	0.79	0.46	7.77E-03	5.42E-02
MRPL17	2150.25	0.79	0.32	1.79E-03	1.97E-02
YIPF5	10062.51	0.79	0.40	4.64E-03	3.82E-02
TMEM45A	7536.69	0.78	0.52	1.01E-02	6.41E-02
AURKA	269.32	0.78	0.68	1.41E-02	8.00E-02
YIPF2	3494.17	0.78	0.44	6.98E-03	5.03E-02
SLC50A1	2605.55	0.78	0.26	4.25E-04	7.22E-03
ZWILCH	1208.73	0.78	0.36	3.76E-03	3.30E-02
RASL11B	881.64	0.78	1.11	1.25E-02	7.35E-02
CCNA2	428.40	0.78	1.33	1.10E-02	6.74E-02
RUNX1	5162.93	0.78	0.83	1.45E-02	8.15E-02
CHPF2	7883.43	0.78	0.40	5.23E-03	4.15E-02
ARSB	2513.23	0.77	0.57	1.25E-02	7.35E-02
SNX25	2215.47	0.77	0.57	1.28E-02	7.44E-02
GPR107	2243.08	0.77	0.26	4.71E-04	7.77E-03
AP2S1	4041.13	0.77	0.30	1.49E-03	1.74E-02
TMEM167B	4919.74	0.76	0.24	2.00E-04	4.26E-03
IKBKE	831.27	0.76	0.32	2.18E-03	2.27E-02
CCDC138	375.76	0.76	0.25	3.25E-04	6.00E-03
LIPT2	122.12	0.76	0.39	5.57E-03	4.31E-02
USP51	167.89	0.76	0.38	5.29E-03	4.18E-02
ZNF561	1346.63	0.76	0.20	1.60E-05	6.43E-04
CRISPLD1	5549.98	0.76	1.19	1.14E-02	6.92E-02
NBPF1	6936.19	0.76	0.30	1.45E-03	1.70E-02
BLOC1S3	864.65	0.76	0.36	4.47E-03	3.72E-02
C1orf122	4007.08	0.76	0.29	1.15E-03	1.45E-02
ETAA1	1877.09	0.76	0.37	4.96E-03	3.99E-02
C2orf81	704.37	0.75	0.38	5.65E-03	4.35E-02
MMP11	2429.48	0.75	0.41	6.95E-03	5.02E-02
P4HA1	12217.39	0.75	0.45	9.22E-03	6.04E-02
ZNF572	138.97	0.75	0.39	6.33E-03	4.71E-02
OR14J1	250.43	0.75	0.25	4.64E-04	7.70E-03
BEND6	1502.01	0.75	1.07	1.36E-02	7.80E-02
UBXN6	1967.32	0.75	0.38	5.44E-03	4.25E-02
SPTSSA	6159.21	0.75	0.25	4.52E-04	7.56E-03
VHL	1847.38	0.75	0.20	2.42E-05	8.88E-04
ATF5	242.53	0.75	0.40	6.92E-03	5.00E-02
PCBP3	508.07	0.74	0.50	1.24E-02	7.31E-02
PPIL3	1458.32	0.74	0.18	5.65E-06	2.96E-04
MFSD1	9613.92	0.74	0.26	7.11E-04	1.03E-02
ZNF613	436.26	0.74	0.27	8.26E-04	1.14E-02
CISD1	2094.62	0.74	0.17	2.55E-06	1.64E-04

KNSTRN	448.61	0.74	0.52	1.32E-02	7.60E-02
MPHOSPH9	1163.07	0.74	0.30	2.04E-03	2.17E-02
SH3BP2	1886.88	0.74	0.62	1.60E-02	8.72E-02
GMPPA	4691.38	0.74	0.28	1.16E-03	1.46E-02
SMYD2	2192.15	0.74	0.36	4.84E-03	3.94E-02
ATF7IP2	1065.56	0.74	1.01	1.43E-02	8.08E-02
ZNF286A	1270.21	0.74	0.35	4.64E-03	3.82E-02
CHRNB1	1070.81	0.73	0.31	2.56E-03	2.55E-02
SLX1A	2017.54	0.73	0.52	1.36E-02	7.79E-02
C21orf58	207.68	0.73	0.31	2.67E-03	2.62E-02
SRD5A1	993.09	0.73	0.57	1.54E-02	8.50E-02
EMC10	1372.61	0.73	0.23	1.92E-04	4.13E-03
PARVB	716.22	0.73	0.45	1.06E-02	6.59E-02
LBHD1	415.38	0.73	0.38	6.46E-03	4.77E-02
ATP8B3	317.17	0.73	0.60	1.64E-02	8.86E-02
BRSK1	32.59	0.73	0.66	1.86E-02	9.68E-02
CAPZB	18471.49	0.73	0.20	5.40E-05	1.61E-03
TTC3	26416.87	0.73	0.41	8.17E-03	5.61E-02
MAGOHB	1799.94	0.73	0.21	8.28E-05	2.19E-03
HSF2BP	71.28	0.73	0.39	8.06E-03	5.56E-02
PPP3R1	492.84	0.73	0.42	9.09E-03	5.99E-02
SLC31A1	3068.65	0.72	0.33	4.12E-03	3.51E-02
IFIT2	2187.49	0.72	0.47	1.21E-02	7.20E-02
PDIK1L	927.80	0.72	0.24	4.27E-04	7.24E-03
FASTKD3	802.81	0.72	0.28	1.61E-03	1.83E-02
ZNF596	369.50	0.72	0.47	1.28E-02	7.45E-02
RBM3	14283.42	0.72	0.28	1.44E-03	1.69E-02
RWDD2B	1251.05	0.72	0.23	3.36E-04	6.16E-03
MSANTD3	2644.63	0.72	0.37	7.11E-03	5.10E-02
TP53I3	4979.41	0.72	0.37	6.49E-03	4.79E-02
CHEK1	545.55	0.71	0.38	7.79E-03	5.43E-02
ZNF273	547.27	0.71	0.29	2.30E-03	2.36E-02
BBOF1	794.78	0.71	0.49	1.41E-02	8.00E-02
SSR2	14410.26	0.71	0.21	1.41E-04	3.22E-03
CCDC167	784.47	0.71	0.28	1.91E-03	2.07E-02
WDR41	4948.75	0.71	0.27	1.41E-03	1.66E-02
SLC19A1	1097.21	0.71	0.39	7.93E-03	5.49E-02
ZNF253	701.81	0.71	0.43	1.10E-02	6.76E-02
SNX30	1850.48	0.71	0.37	7.20E-03	5.15E-02
MC1R	630.39	0.71	0.58	1.75E-02	9.30E-02
CCDC122	541.98	0.71	0.48	1.38E-02	7.90E-02
NUDT12	1030.15	0.70	0.26	1.07E-03	1.38E-02
BIVM	2307.53	0.70	0.41	9.68E-03	6.25E-02
NUCB2	11440.99	0.70	0.57	1.65E-02	8.90E-02
COG6	4600.16	0.70	0.42	1.06E-02	6.59E-02
TONSL	436.03	0.70	0.36	6.58E-03	4.83E-02
COPZ2	12184.77	0.70	0.57	1.65E-02	8.89E-02
HYLS1	706.88	0.70	0.40	9.61E-03	6.21E-02
ENAH	7190.16	0.70	0.45	1.24E-02	7.29E-02
PRELID3A	41.71	0.70	0.37	8.41E-03	5.69E-02
PLIN3	11422.97	0.70	0.24	7.85E-04	1.10E-02
CNIH1	7797.34	0.70	0.24	5.88E-04	9.02E-03
NAA40	1026.83	0.70	0.26	1.13E-03	1.43E-02
CCZ1B	3887.13	0.69	0.37	8.24E-03	5.64E-02
CTBS	3937.42	0.69	0.20	8.47E-05	2.22E-03
ZNF587	913.81	0.69	0.19	5.03E-05	1.53E-03
PEX12	752.04	0.69	0.26	1.40E-03	1.66E-02
RARS	3937.07	0.69	0.19	5.65E-05	1.67E-03
NINL	1157.15	0.69	0.54	1.78E-02	9.40E-02
KDM2B	927.61	0.69	0.23	5.28E-04	8.36E-03
PUS7L	1721.02	0.69	0.19	4.34E-05	1.36E-03
SLC29A3	1814.41	0.69	0.29	2.79E-03	2.67E-02
ODF2L	3970.94	0.69	0.37	8.14E-03	5.59E-02
FADD	1069.01	0.69	0.39	9.21E-03	6.04E-02
TMEM119	15776.71	0.69	0.65	1.90E-02	9.79E-02
CA11	548.25	0.69	0.36	8.13E-03	5.59E-02
ZNF260	2049.24	0.69	0.22	2.85E-04	5.46E-03
HSD11B1L	954.33	0.69	0.43	1.22E-02	7.22E-02
RCC2	2838.54	0.69	0.44	1.31E-02	7.59E-02
TMEM121	456.82	0.69	0.40	1.09E-02	6.72E-02
FAM227A	130.99	0.69	0.40	1.13E-02	6.89E-02
ATXN1	3219.09	0.68	0.16	5.47E-06	2.89E-04
SQLE	1776.96	0.68	0.52	1.80E-02	9.47E-02
PEX11B	1979.96	0.68	0.38	9.25E-03	6.05E-02
GGACT	431.60	0.68	0.46	1.51E-02	8.38E-02
NBPF15	2339.14	0.68	0.30	3.60E-03	3.18E-02
TMEM223	1504.89	0.68	0.23	5.82E-04	8.94E-03
ZBED6	1043.21	0.68	0.25	1.13E-03	1.43E-02
FHOD1	1154.17	0.68	0.41	1.21E-02	7.18E-02
FBXL8	899.21	0.68	0.42	1.29E-02	7.50E-02
STXBP5	2740.29	0.68	0.38	1.06E-02	6.57E-02
EPHB4	3391.51	0.68	0.16	5.20E-06	2.79E-04
PLGRKT	1333.94	0.68	0.25	1.21E-03	1.50E-02
ATP6V1D	4706.93	0.67	0.33	6.58E-03	4.83E-02
LAPTM4B	5957.11	0.67	0.22	4.37E-04	7.36E-03
ERCC2	498.63	0.67	0.14	7.31E-07	6.30E-05
FEN1	958.40	0.67	0.46	1.64E-02	8.85E-02
AP5S1	1100.97	0.67	0.16	6.56E-06	3.32E-04
POLN	159.94	0.67	0.32	5.72E-03	4.38E-02
RPL26L1	1537.24	0.67	0.20	2.11E-04	4.40E-03
RAI14	4277.44	0.67	0.41	1.28E-02	7.44E-02
TIMP1	74677.24	0.67	0.54	1.50E-02	8.34E-02
EFNB1	4375.76	0.67	0.35	9.05E-03	5.97E-02
ACP2	2913.25	0.67	0.37	1.04E-02	6.54E-02
SLC35A2	2417.49	0.67	0.29	3.87E-03	3.36E-02
WHRN	779.94	0.67	0.26	1.82E-03	1.99E-02
CCDC34	436.40	0.67	0.33	6.79E-03	4.94E-02
PCNA	3589.19	0.67	0.26	1.74E-03	1.94E-02
ETV2	70.65	0.67	0.46	1.79E-02	9.44E-02
HMBS	1328.39	0.66	0.36	9.62E-03	6.21E-02
ZNF485	219.62	0.66	0.20	1.80E-04	3.94E-03
PPP1R9B	490.08	0.66	0.32	5.90E-03	4.49E-02

ITPR2	1744.94	0.66	0.50	1.91E-02	9.84E-02
IBTK	5087.89	0.66	0.15	1.32E-06	1.01E-04
MGAT4B	3622.72	0.66	0.45	1.55E-02	8.55E-02
ARF4	44829.90	0.66	0.41	1.22E-02	7.23E-02
MED8	3960.12	0.66	0.22	6.06E-04	9.14E-03
TMEM167A	10368.64	0.66	0.45	1.61E-02	8.77E-02
AKAP7	674.17	0.66	0.31	5.83E-03	4.44E-02
TMEM65	1149.23	0.66	0.36	9.90E-03	6.35E-02
C19orf48	1577.55	0.66	0.24	1.37E-03	1.64E-02
MOSPD1	1338.98	0.66	0.25	1.60E-03	1.82E-02
RHNO1	1422.08	0.66	0.20	1.92E-04	4.13E-03
UBXN8	767.47	0.65	0.29	3.86E-03	3.36E-02
HNMT	6644.30	0.65	0.34	8.58E-03	5.78E-02
IFT80	2454.51	0.65	0.46	1.82E-02	9.55E-02
MFAP3	1763.97	0.65	0.23	7.42E-04	1.06E-02
CCDC18	462.93	0.65	0.32	7.07E-03	5.07E-02
TMEM87B	3178.83	0.65	0.23	8.04E-04	1.12E-02
C20orf96	320.04	0.65	0.28	4.17E-03	3.54E-02
MAP4K4	15542.85	0.65	0.87	1.87E-02	9.68E-02
PPP1R37	420.66	0.65	0.40	1.49E-02	8.32E-02
ZNF467	720.59	0.65	1.39	1.00E-02	6.37E-02
AGPAT3	1740.77	0.64	0.29	4.72E-03	3.86E-02
ISCA2	1662.32	0.64	0.34	1.01E-02	6.38E-02
ZNF784	174.62	0.64	0.43	1.71E-02	9.15E-02
NHSL2	2816.25	0.64	0.20	2.60E-04	5.12E-03
RNASE10	102.65	0.64	0.33	8.73E-03	5.83E-02
NUS1	2958.34	0.64	0.35	1.07E-02	6.63E-02
TPM4	67910.60	0.64	0.26	2.38E-03	2.42E-02
MCTS1	2876.14	0.64	0.20	3.90E-04	6.82E-03
VIPAS39	1523.26	0.64	0.10	6.92E-12	3.93E-09
STK16	776.68	0.64	0.31	6.59E-03	4.83E-02
FBXO41	123.26	0.64	1.05	1.57E-02	8.62E-02
ZNF117	893.49	0.64	0.41	1.66E-02	8.93E-02
DNAJC1	3207.29	0.63	0.23	1.39E-03	1.65E-02
SOX4	10408.63	0.63	0.30	6.40E-03	4.74E-02
UROS	3796.84	0.63	0.29	5.08E-03	4.06E-02
RAB27A	2028.78	0.63	0.40	1.67E-02	8.99E-02
UBTD1	2156.64	0.63	0.33	1.03E-02	6.50E-02
NMNAT1	942.33	0.63	0.25	2.75E-03	2.65E-02
GOLGA5	6576.83	0.63	0.35	1.22E-02	7.22E-02
TSPAN5	1337.33	0.62	0.95	1.75E-02	9.29E-02
MPDU1	3701.06	0.62	0.35	1.31E-02	7.59E-02
KCTD18	1702.20	0.62	0.19	2.19E-04	4.50E-03
KLK10	154.79	0.62	1.17	1.29E-02	7.49E-02
ALG5	3441.66	0.62	0.17	6.28E-05	1.82E-03
YDJC	905.38	0.62	0.34	1.11E-02	6.81E-02
MGAT2	3755.62	0.62	0.39	1.67E-02	8.98E-02
C16orf87	1178.53	0.62	0.24	2.05E-03	2.17E-02
ZNF578	94.65	0.62	0.35	1.39E-02	7.92E-02
COA6	1158.45	0.62	0.30	6.90E-03	4.99E-02
ALKBH4	1076.47	0.62	0.27	4.20E-03	3.55E-02
EML4	3003.82	0.62	0.40	1.79E-02	9.43E-02
ZNF69	332.23	0.62	0.27	4.88E-03	3.95E-02
ANKIB1	2804.67	0.62	0.31	8.87E-03	5.90E-02
ZNF254	1768.68	0.61	0.18	2.06E-04	4.35E-03
MORF4L2	24859.26	0.61	0.25	3.08E-03	2.86E-02
P4HB	65037.84	0.61	0.40	1.76E-02	9.32E-02
ASTE1	1124.46	0.61	0.24	2.63E-03	2.59E-02
TMED2	46161.56	0.61	0.27	5.28E-03	4.18E-02
ZNF223	381.59	0.61	0.27	4.63E-03	3.82E-02
TMEM208	2709.22	0.61	0.14	1.84E-06	1.28E-04
ZNF30	305.85	0.61	0.34	1.39E-02	7.92E-02
ZNF616	1077.20	0.60	0.37	1.70E-02	9.10E-02
MPHOSPH10	3025.20	0.60	0.26	4.22E-03	3.56E-02
MYDGF	12234.34	0.60	0.32	1.11E-02	6.81E-02
PIGW	620.35	0.60	0.23	2.19E-03	2.27E-02
TMEM243	3894.62	0.60	0.12	1.66E-07	2.01E-05
SEC22B	8662.40	0.60	0.34	1.32E-02	7.60E-02
HTR2A	979.24	0.60	1.08	1.35E-02	7.77E-02
MNS1	463.82	0.60	0.35	1.58E-02	8.65E-02
CNPY2	4702.85	0.60	0.13	8.91E-07	7.31E-05
ZNF816	469.78	0.60	0.27	5.47E-03	4.26E-02
TRIQK	3145.73	0.59	0.32	1.20E-02	7.16E-02
TEN1	816.09	0.59	0.23	2.47E-03	2.48E-02
KDM4D	123.28	0.59	0.29	9.13E-03	6.00E-02
MED18	437.20	0.59	0.22	1.63E-03	1.85E-02
GGCT	1449.32	0.59	0.23	2.10E-03	2.21E-02
EFCAB11	547.72	0.59	0.20	8.10E-04	1.13E-02
G6PC3	3998.91	0.59	0.25	4.37E-03	3.66E-02
EFNB2	1663.53	0.59	1.00	1.59E-02	8.70E-02
GAR1	1028.38	0.59	0.23	2.57E-03	2.55E-02
TBC1D32	581.24	0.59	0.31	1.08E-02	6.67E-02
PEA15	6280.69	0.59	0.32	1.30E-02	7.53E-02
SMIM15	4820.27	0.59	0.22	1.88E-03	2.05E-02
TMEM141	1713.00	0.58	0.25	4.87E-03	3.95E-02
CMPK2	522.11	0.58	0.35	1.82E-02	9.53E-02
MIA2	2753.46	0.58	0.17	1.18E-04	2.80E-03
FHL3	4106.04	0.58	0.21	1.31E-03	1.58E-02
DBR1	1563.79	0.58	0.26	5.63E-03	4.33E-02
THUMPD3	1671.44	0.58	0.15	2.05E-05	7.78E-04
RAB9A	2929.13	0.58	0.34	1.63E-02	8.82E-02
ADAMTS2	33467.83	0.58	0.89	1.60E-02	8.72E-02
PKM	53444.12	0.58	0.31	1.13E-02	6.89E-02
GDI2	19399.66	0.58	0.26	6.10E-03	4.58E-02
DAP	16125.63	0.58	0.25	4.62E-03	3.80E-02
ZNF514	558.78	0.58	0.29	9.24E-03	6.05E-02
AMZ2	4874.44	0.58	0.10	2.35E-09	5.54E-07
ALG1	2623.42	0.58	0.30	1.07E-02	6.63E-02
SPC24	117.88	0.58	0.35	1.87E-02	9.70E-02
TARBP2	1259.43	0.58	0.26	5.78E-03	4.41E-02
THNSL1	687.83	0.58	0.34	1.75E-02	9.29E-02
WNT3	202.30	0.58	0.35	1.80E-02	9.45E-02

MTERF1	733.27	0.58	0.22	2.43E-03	2.46E-02
LRRC1	754.53	0.58	0.35	1.85E-02	9.62E-02
SHMT2	3103.48	0.58	0.19	7.72E-04	1.09E-02
ZNF720	1446.10	0.58	0.17	1.41E-04	3.22E-03
PPCS	4793.23	0.58	0.08	2.09E-12	1.45E-09
HOXB7	1015.25	0.58	0.27	7.63E-03	5.36E-02
ZNF827	1276.08	0.58	0.29	1.05E-02	6.56E-02
ZNF814	649.21	0.58	0.30	1.21E-02	7.19E-02
COQ2	1113.04	0.58	0.21	1.54E-03	1.77E-02
BNIP1	731.15	0.57	0.21	1.76E-03	1.96E-02
PROX2	9.54	0.57	1.33	8.57E-03	5.77E-02
AKR1A1	9239.92	0.57	0.22	2.42E-03	2.45E-02
COMMD9	3219.99	0.57	0.27	8.14E-03	5.59E-02
BROX	4107.54	0.57	0.22	2.29E-03	2.35E-02
ZNHIT1	5479.38	0.57	0.21	1.53E-03	1.77E-02
MLST8	3150.95	0.57	0.11	3.77E-08	5.78E-06
NME2	10708.11	0.57	0.34	1.79E-02	9.45E-02
SLFN13	509.92	0.57	0.31	1.30E-02	7.53E-02
JTB	13518.19	0.57	0.25	5.36E-03	4.22E-02
GSR	2972.95	0.57	0.22	2.56E-03	2.55E-02
NABP2	462.26	0.57	0.33	1.75E-02	9.31E-02
SLC39A4	495.07	0.57	0.29	1.09E-02	6.73E-02
SCRN3	1360.86	0.57	0.16	1.02E-04	2.55E-03
SPINK9	97.35	0.57	0.25	5.59E-03	4.32E-02
E2F5	269.92	0.57	0.28	1.01E-02	6.39E-02
C19orf24	1576.44	0.57	0.18	5.15E-04	8.19E-03
TDRD3	1437.55	0.57	0.30	1.21E-02	7.21E-02
PIGBOS1	934.34	0.57	0.18	5.62E-04	8.75E-03
IDH1	8054.56	0.56	0.29	1.05E-02	6.56E-02
ZNF419	699.84	0.56	0.32	1.63E-02	8.82E-02
AIDA	6541.61	0.56	0.33	1.86E-02	9.68E-02
SPC25	172.01	0.56	0.99	1.55E-02	8.56E-02
NLK	235.82	0.56	0.17	3.70E-04	6.59E-03
RARS2	5799.62	0.56	0.32	1.58E-02	8.67E-02
ZNF569	729.21	0.56	0.21	2.31E-03	2.36E-02
SLC38A7	2193.90	0.56	0.27	8.21E-03	5.62E-02
CNEP1R1	2155.50	0.56	0.16	1.20E-04	2.85E-03
OR5A1	53.72	0.56	0.28	1.19E-02	7.13E-02
L3HYPDH	2282.26	0.56	0.23	3.38E-03	3.04E-02
COMMD8	2339.97	0.56	0.29	1.29E-02	7.50E-02
LGALS3	32432.22	0.56	0.20	1.25E-03	1.53E-02
ZNF550	1010.03	0.56	0.23	4.23E-03	3.57E-02
LTC4S	613.25	0.56	0.91	1.85E-02	9.62E-02
SYT3	37.75	0.56	0.93	1.85E-02	9.63E-02
TXNDC17	2449.64	0.56	0.28	1.13E-02	6.89E-02
ZNF776	943.13	0.56	0.27	9.10E-03	5.99E-02
RFESD	202.77	0.55	0.30	1.51E-02	8.38E-02
SULT1B1	758.88	0.55	1.06	1.16E-02	6.98E-02
CCDC106	1015.97	0.55	0.18	7.27E-04	1.05E-02
ZIK1	458.95	0.55	0.31	1.73E-02	9.23E-02
TMEM106A	782.98	0.55	0.28	1.19E-02	7.11E-02
ERCC8	940.89	0.55	0.17	4.47E-04	7.49E-03
JRKL	1570.40	0.55	0.20	1.86E-03	2.03E-02
C8orf76	1187.82	0.55	0.28	1.25E-02	7.36E-02
RHBDD3	1751.40	0.55	0.16	1.81E-04	3.95E-03
METTL18	557.97	0.55	0.30	1.61E-02	8.77E-02
ZNF562	1110.02	0.55	0.30	1.61E-02	8.77E-02
AP5M1	3545.37	0.55	0.19	1.18E-03	1.47E-02
EIF2AK1	9207.34	0.54	0.21	2.85E-03	2.71E-02
IFI44	1782.78	0.54	0.30	1.63E-02	8.82E-02
ADAM32	362.33	0.54	0.26	9.87E-03	6.33E-02
CUTA	9299.63	0.54	0.22	3.35E-03	3.03E-02
UCHL3	1726.28	0.54	0.31	1.89E-02	9.75E-02
TRPM7	3874.80	0.54	0.26	8.92E-03	5.92E-02
LGALS1	67604.99	0.54	0.30	1.90E-02	9.81E-02
EEF1A2	38.10	0.54	1.26	5.36E-03	4.22E-02
IFI27L2	1938.31	0.54	0.26	1.00E-02	6.38E-02
GLMN	761.77	0.54	0.24	6.03E-03	4.55E-02
TCEAL9	12829.92	0.54	0.29	1.45E-02	8.17E-02
RBAK	1433.51	0.54	0.22	4.18E-03	3.54E-02
VPS25	3085.83	0.54	0.15	1.04E-04	2.58E-03
ZBED3	2151.56	0.54	0.30	1.73E-02	9.21E-02
ZNF107	704.32	0.54	0.17	4.27E-04	7.23E-03
DPAGT1	3571.06	0.54	0.17	6.11E-04	9.19E-03
PRKCSH	6437.04	0.53	0.26	1.09E-02	6.73E-02
TFB2M	1344.31	0.53	0.21	2.66E-03	2.62E-02
ZNF461	445.44	0.53	0.27	1.26E-02	7.40E-02
MRS2	2168.49	0.53	0.17	7.27E-04	1.05E-02
MIER2	1360.41	0.53	0.27	1.26E-02	7.39E-02
ZNF189	653.41	0.53	0.24	7.28E-03	5.18E-02
ZSCAN2	447.50	0.53	0.19	1.78E-03	1.96E-02
LZTFL1	1934.05	0.53	0.29	1.61E-02	8.76E-02
CD2AP	1783.21	0.53	0.20	2.56E-03	2.55E-02
C12orf76	732.52	0.53	0.21	3.56E-03	3.17E-02
SPAST	1871.38	0.53	0.26	1.04E-02	6.52E-02
ANAPC13	6336.66	0.53	0.11	4.46E-07	4.28E-05
EDARADD	122.00	0.53	1.75	2.27E-04	4.61E-03
B9D2	328.01	0.53	0.24	8.11E-03	5.58E-02
CHD3	4022.92	0.52	0.11	1.67E-06	1.21E-04
TRIM27	4650.71	0.52	0.17	5.40E-04	8.48E-03
RCBTB2	3083.38	0.52	0.10	3.36E-08	5.21E-06
CDK7	1461.97	0.52	0.21	3.25E-03	2.95E-02
B3GALT6	2970.30	0.52	0.18	1.19E-03	1.48E-02
RPP38	946.26	0.52	0.23	6.95E-03	5.02E-02
CHCHD1	1523.10	0.52	0.23	5.92E-03	4.50E-02
PIGC	3467.75	0.52	0.21	4.48E-03	3.72E-02
SFXN5	1041.71	0.52	0.27	1.39E-02	7.92E-02
TMEM39A	5444.47	0.52	0.29	1.81E-02	9.51E-02
C14orf119	5163.51	0.52	0.24	8.89E-03	5.91E-02
B3GNTL1	407.13	0.52	0.23	7.83E-03	5.45E-02
FAM200A	793.18	0.52	0.27	1.53E-02	8.48E-02
C18orf21	534.18	0.52	0.26	1.23E-02	7.26E-02

PEAK1	5182.24	0.52	0.22	5.81E-03	4.44E-02
PRIM2	798.19	0.52	0.27	1.52E-02	8.41E-02
TGDS	1342.12	0.52	0.19	1.77E-03	1.96E-02
TMEM260	2114.33	0.51	0.14	1.23E-04	2.89E-03
FAM114A2	2456.55	0.51	0.22	6.08E-03	4.57E-02
TACO1	1098.17	0.51	0.16	3.18E-04	5.92E-03
AP3B1	5657.08	0.51	0.22	5.95E-03	4.51E-02
APH1A	4528.44	0.51	0.25	1.13E-02	6.89E-02
TOR1AIP2	3929.80	0.51	0.25	1.10E-02	6.76E-02
CGREF1	2351.62	0.51	1.00	9.58E-03	6.19E-02
SLC25A16	783.96	0.51	0.15	2.81E-04	5.40E-03
DDX42	2691.25	0.51	0.26	1.40E-02	7.98E-02
ZFP30	582.42	0.51	0.16	5.94E-04	9.06E-03
ATP2C1	4856.20	0.51	0.26	1.46E-02	8.19E-02
POLA2	587.85	0.51	0.26	1.41E-02	8.01E-02
DECR1	5352.22	0.51	0.23	7.13E-03	5.11E-02
FOXP1	12943.84	0.51	0.17	1.07E-03	1.38E-02
NXT2	1245.13	0.51	0.19	2.35E-03	2.39E-02
EBP	1441.51	0.51	0.19	2.45E-03	2.47E-02
CBX3	7302.65	0.51	0.19	2.87E-03	2.72E-02
ZCCHC7	2236.12	0.51	0.12	5.18E-06	2.79E-04
FAH	2690.41	0.51	1.09	5.08E-03	4.06E-02
GLB1	7386.17	0.51	0.25	1.23E-02	7.25E-02
TRNT1	1761.97	0.50	0.25	1.17E-02	7.02E-02
ZNF180	839.84	0.50	0.26	1.45E-02	8.17E-02
RGS17	132.96	0.50	0.85	1.88E-02	9.73E-02
MARCKSL1	11813.60	0.50	0.81	1.94E-02	9.94E-02
AGGF1	3205.55	0.50	0.14	8.45E-05	2.22E-03
ZNF490	266.61	0.50	0.26	1.40E-02	7.97E-02
OVCA2	1236.42	0.50	0.19	2.68E-03	2.62E-02
SEC61G	5335.01	0.50	0.19	2.66E-03	2.61E-02
ZSCAN20	241.88	0.50	0.23	8.42E-03	5.69E-02
TAOK2	2844.37	0.50	0.26	1.59E-02	8.68E-02
CCDC90B	4964.44	0.50	0.16	6.15E-04	9.24E-03
NPTX2	464.73	0.50	1.11	3.79E-03	3.32E-02
PDIA3	54467.48	0.50	0.23	9.29E-03	6.07E-02
EME1	119.16	0.50	0.88	1.57E-02	8.62E-02
B4GALT7	2297.07	0.50	0.19	2.35E-03	2.39E-02
MYO1B	14724.95	0.50	0.21	5.55E-03	4.29E-02
SHANK2	42.61	0.50	0.94	1.16E-02	7.00E-02
TRA2A	3345.82	0.50	0.21	6.49E-03	4.79E-02
GPATCH2	1236.44	0.50	0.17	1.24E-03	1.52E-02
SAC3D1	882.84	0.50	0.23	8.67E-03	5.81E-02
LAGE3	954.34	0.50	0.21	5.15E-03	4.10E-02
C3orf38	2264.77	0.49	0.23	9.09E-03	5.99E-02
SMIM20	1923.49	0.49	0.20	4.47E-03	3.72E-02
CASP6	1336.90	0.49	0.24	1.28E-02	7.46E-02
TATDN2	1389.23	0.49	0.26	1.68E-02	9.01E-02
RAP2A	3625.67	0.49	0.24	1.18E-02	7.06E-02
PPIC	23173.86	0.49	0.75	1.93E-02	9.90E-02
MRPS33	1234.81	0.49	0.20	4.57E-03	3.78E-02
DNAJC4	2125.03	0.49	0.22	7.41E-03	5.24E-02
PGLS	2946.59	0.49	0.24	1.18E-02	7.06E-02
SRP9	18588.62	0.49	0.13	8.71E-05	2.25E-03
CSGALNACT2	5414.97	0.49	0.25	1.53E-02	8.47E-02
WDFY1	9030.44	0.49	0.17	1.30E-03	1.58E-02
SLFN12L	157.58	0.49	0.92	1.10E-02	6.76E-02
NCK1	3502.07	0.49	0.19	3.42E-03	3.06E-02
IMPACT	3731.85	0.48	0.14	2.96E-04	5.61E-03
DERL2	4883.65	0.48	0.19	3.41E-03	3.06E-02
MRI1	1701.87	0.48	0.25	1.59E-02	8.70E-02
TBC1D23	6371.45	0.48	0.22	9.93E-03	6.36E-02
TEX11	16.70	0.48	1.07	2.86E-03	2.71E-02
RWDD2A	954.88	0.48	0.19	3.43E-03	3.07E-02
SUPT20H	4018.32	0.48	0.15	4.12E-04	7.08E-03
PDZD11	2869.80	0.48	0.25	1.78E-02	9.40E-02
SCN11A	95.22	0.48	0.96	6.57E-03	4.83E-02
CCDC110	537.77	0.48	0.85	1.44E-02	8.13E-02
RGS4	4649.99	0.48	1.00	3.84E-03	3.34E-02
S100PBP	2753.24	0.48	0.15	4.83E-04	7.90E-03
COX18	495.16	0.48	0.19	3.82E-03	3.33E-02
OXLD1	1271.58	0.48	0.14	2.79E-04	5.38E-03
ALG3	3179.62	0.48	0.18	2.71E-03	2.63E-02
MYL6B	1918.01	0.48	0.23	1.22E-02	7.24E-02
COX16	2671.49	0.47	0.13	1.09E-04	2.67E-03
TMEM42	1313.10	0.47	0.21	7.31E-03	5.19E-02
LINS1	1327.15	0.47	0.21	7.15E-03	5.12E-02
VTA1	6045.60	0.47	0.21	8.39E-03	5.68E-02
CDK19	676.08	0.47	0.21	8.15E-03	5.60E-02
LGALS2	461.56	0.47	1.21	8.16E-05	2.18E-03
KDELR1	11981.39	0.47	0.24	1.53E-02	8.49E-02
EVA1B	3240.48	0.47	0.18	3.29E-03	2.99E-02
COMMD3	3727.63	0.47	0.09	1.56E-08	2.74E-06
ASCC3	4324.46	0.47	0.16	1.04E-03	1.36E-02
SCFD1	7625.92	0.47	0.17	2.45E-03	2.48E-02
KDM8	438.49	0.47	0.19	4.27E-03	3.59E-02
RNF113A	886.50	0.47	0.15	9.61E-04	1.27E-02
TRAPPC1	3919.67	0.47	0.18	4.10E-03	3.50E-02
SAYSD1	1503.95	0.47	0.21	8.86E-03	5.90E-02
TMEFF1	108.65	0.47	0.95	4.78E-03	3.90E-02
ADAT1	1241.55	0.47	0.15	7.32E-04	1.05E-02
COPZ1	9781.28	0.46	0.18	3.20E-03	2.93E-02
NFXL1	1163.08	0.46	0.16	1.54E-03	1.77E-02
ALG14	1336.99	0.46	0.18	4.01E-03	3.45E-02
PPHLN1	4239.11	0.46	0.12	2.40E-05	8.82E-04
GEMIN6	583.29	0.46	0.20	8.01E-03	5.53E-02
NEURL4	507.17	0.46	0.14	4.03E-04	6.96E-03
RBM4B	2157.92	0.46	0.23	1.43E-02	8.08E-02
SUPT4H1	3693.63	0.46	0.17	3.01E-03	2.82E-02
GTPBP8	858.36	0.46	0.18	4.73E-03	3.87E-02
C1QL1	248.70	0.46	0.98	2.00E-03	2.14E-02
TMEM219	8374.08	0.46	0.12	3.32E-05	1.12E-03

MMS22L	588.57	0.46	0.22	1.24E-02	7.32E-02
IL27RA	927.83	0.45	0.92	4.05E-03	3.47E-02
GSK3B	2487.05	0.45	0.15	8.59E-04	1.18E-02
FAHD1	1622.74	0.45	0.18	4.42E-03	3.69E-02
DAD1	16794.70	0.45	0.21	1.16E-02	7.01E-02
LOX	39004.88	0.45	0.78	1.30E-02	7.53E-02
ADM2	65.80	0.45	0.83	1.19E-02	7.11E-02
COL1A2	3678459.70	0.45	0.76	2.36E-04	4.76E-03
NAGK	5564.84	0.45	0.21	1.06E-02	6.59E-02
SUSD3	391.13	0.45	0.90	4.81E-03	3.92E-02
FIBP	4670.99	0.45	0.20	1.00E-02	6.38E-02
CHRNA9	8.06	0.45	1.07	5.78E-05	1.70E-03
HAUS3	1987.12	0.45	0.10	3.29E-06	2.00E-04
ZNF611	1206.22	0.45	0.18	5.20E-03	4.13E-02
MTRF1	714.27	0.45	0.23	1.70E-02	9.09E-02
ESCO2	184.44	0.45	0.85	8.52E-03	5.75E-02
RBM45	766.16	0.45	0.20	9.82E-03	6.32E-02
ANKRD49	1369.94	0.45	0.23	1.65E-02	8.90E-02
ARL14EP	2620.89	0.45	0.16	1.57E-03	1.80E-02
TMEM216	1695.73	0.45	0.21	1.28E-02	7.45E-02
PMCH	7.46	0.45	1.04	8.08E-05	2.16E-03
YARS2	1376.81	0.45	0.19	6.93E-03	5.01E-02
FUNDC1	997.12	0.45	0.16	1.89E-03	2.06E-02
FKBP3	4310.09	0.45	0.19	7.36E-03	5.21E-02
KRCC1	2638.68	0.44	0.20	1.16E-02	7.00E-02
PPIP5K2	5534.66	0.44	0.14	5.51E-04	8.63E-03
SH3GLB1	12787.65	0.44	0.18	5.39E-03	4.22E-02
NUP37	1607.34	0.44	0.21	1.24E-02	7.29E-02
C1orf131	1025.82	0.44	0.16	2.43E-03	2.46E-02
INIP	1969.00	0.44	0.17	4.46E-03	3.72E-02
DPM2	2775.61	0.44	0.14	9.10E-04	1.22E-02
LAMP5	1737.71	0.44	0.85	5.39E-03	4.22E-02
GTF2H5	2809.82	0.44	0.08	6.72E-08	9.12E-06
TIAL1	1754.81	0.44	0.21	1.27E-02	7.42E-02
ACIN1	2743.63	0.44	0.08	1.97E-08	3.44E-06
TTC9C	1179.18	0.44	0.14	5.56E-04	8.68E-03
DYRK1B	1294.96	0.44	0.17	4.96E-03	3.98E-02
ZNF480	1520.95	0.44	0.18	6.23E-03	4.65E-02
RING1	1244.25	0.44	0.22	1.71E-02	9.14E-02
ZCCHC9	1400.32	0.43	0.13	4.08E-04	7.03E-03
SMIM18	11.25	0.43	0.80	1.17E-02	7.04E-02
C18orf32	6307.23	0.43	0.15	1.39E-03	1.65E-02
C10orf82	102.17	0.43	0.84	5.40E-03	4.23E-02
ELP6	1437.80	0.43	0.20	1.19E-02	7.11E-02
ZDHHC16	2559.30	0.43	0.19	9.68E-03	6.25E-02
APLP1	1426.56	0.43	0.73	1.91E-02	9.84E-02
MAPK8	1461.60	0.43	0.17	4.94E-03	3.98E-02
CCDC43	1761.96	0.43	0.20	1.27E-02	7.43E-02
NDST3	24.58	0.43	0.81	7.27E-03	5.18E-02
KLK11	67.15	0.43	0.77	1.29E-02	7.48E-02
ZNF138	680.79	0.43	0.22	1.93E-02	9.90E-02
SPOCD1	76.18	0.43	0.89	9.94E-04	1.31E-02
TMEM126B	3131.16	0.43	0.15	1.94E-03	2.09E-02
DSG2	2251.22	0.43	0.94	8.47E-05	2.22E-03
MRPL19	3286.13	0.43	0.13	3.52E-04	6.37E-03
DENR	6466.10	0.43	0.14	9.87E-04	1.30E-02
CENPW	1224.84	0.43	0.73	1.83E-02	9.58E-02
NXPH4	158.53	0.43	0.89	9.91E-04	1.31E-02
CASP2	1371.79	0.42	0.17	6.10E-03	4.58E-02
ACTR8	1893.47	0.42	0.15	1.62E-03	1.84E-02
TMEM258	4029.32	0.42	0.15	1.91E-03	2.07E-02
WDPCP	1454.56	0.42	0.19	1.06E-02	6.58E-02
SCG2	3064.12	0.42	0.94	1.70E-05	6.75E-04
CARNMT1	1528.12	0.42	0.19	1.23E-02	7.27E-02
RAB33A	145.95	0.42	0.73	1.62E-02	8.80E-02
DEUP1	17.88	0.42	0.93	4.33E-05	1.36E-03
ARHGAP8	82.25	0.42	0.77	9.99E-03	6.37E-02
CHST6	396.47	0.42	0.85	1.59E-03	1.82E-02
R3HDM1	1392.85	0.42	0.18	9.05E-03	5.97E-02
SMYD3	1424.49	0.42	0.17	4.94E-03	3.98E-02
POLQ	98.28	0.42	0.82	2.68E-03	2.62E-02
ARF5	10209.80	0.41	0.14	1.02E-03	1.33E-02
GSS	2762.72	0.41	0.15	3.23E-03	2.95E-02
TIRAP	492.77	0.41	0.16	4.44E-03	3.70E-02
ZNF766	1734.63	0.41	0.15	2.82E-03	2.69E-02
RAD50	5210.32	0.41	0.14	1.64E-03	1.86E-02
GAS2L3	336.53	0.41	0.72	1.41E-02	8.00E-02
ZMYM2	3948.05	0.41	0.20	1.79E-02	9.43E-02
NUPL2	1606.04	0.41	0.12	3.86E-04	6.77E-03
HLTF	4399.98	0.41	0.20	1.75E-02	9.29E-02
RWDD3	1354.04	0.41	0.19	1.40E-02	7.97E-02
LEO1	2163.94	0.41	0.16	3.92E-03	3.40E-02
C1orf174	2371.15	0.41	0.13	6.89E-04	1.01E-02
MED19	1404.55	0.41	0.20	1.87E-02	9.71E-02
PTPRN	70.33	0.41	0.81	1.37E-03	1.64E-02
ADCK5	748.78	0.41	0.17	8.16E-03	5.60E-02
MORF4L1	33867.10	0.41	0.15	3.58E-03	3.17E-02
ELAC1	909.13	0.40	0.17	6.71E-03	4.89E-02
ORC5	1317.52	0.40	0.09	4.08E-06	2.35E-04
C9orf85	992.72	0.40	0.13	1.11E-03	1.42E-02
ZNF146	5703.56	0.40	0.17	9.21E-03	6.04E-02
CPSF2	3084.32	0.40	0.18	1.26E-02	7.36E-02
ALG2	3859.20	0.40	0.19	1.41E-02	8.01E-02
TMCO1	8813.33	0.40	0.14	2.85E-03	2.71E-02
MRPL27	3350.33	0.40	0.19	1.62E-02	8.79E-02
LAG3	691.71	0.40	0.75	4.94E-03	3.98E-02
PUSL1	983.69	0.40	0.17	9.49E-03	6.15E-02
YIPF3	10400.29	0.40	0.18	1.20E-02	7.15E-02
SLC25A46	4005.78	0.39	0.10	7.34E-05	2.04E-03
C12orf73	520.91	0.39	0.19	1.68E-02	9.01E-02
SLC35E3	1839.36	0.39	0.17	1.01E-02	6.40E-02
NSA2	1482.29	0.39	0.17	1.12E-02	6.82E-02

OST4	11688.54	0.39	0.16	6.62E-03	4.84E-02
TADA1	1318.79	0.39	0.19	1.79E-02	9.42E-02
STX18	2365.57	0.39	0.09	1.40E-05	5.82E-04
EED	1572.45	0.39	0.18	1.50E-02	8.36E-02
GTF2H3	1524.88	0.39	0.13	1.30E-03	1.58E-02
DHX29	3415.04	0.39	0.17	1.00E-02	6.37E-02
YEATS2	1551.82	0.39	0.18	1.46E-02	8.18E-02
SMC6	2951.05	0.39	0.14	3.02E-03	2.82E-02
CIR1	2795.53	0.38	0.15	6.00E-03	4.54E-02
ANGPTL6	298.75	0.38	0.72	5.84E-03	4.45E-02
SEZ6L2	1210.01	0.38	0.77	3.49E-04	6.36E-03
ZCCHC12	154.40	0.38	0.71	6.90E-03	5.00E-02
ZNF281	2407.22	0.38	0.16	8.97E-03	5.94E-02
BICD1	1762.81	0.38	0.18	1.59E-02	8.68E-02
HTR2B	799.54	0.38	0.71	6.61E-03	4.84E-02
MPV17	4772.26	0.38	0.15	5.70E-03	4.37E-02
ARMCX6	1180.18	0.38	0.15	4.54E-03	3.76E-02
CNTN6	137.84	0.38	0.70	7.17E-03	5.13E-02
DLG1	3881.33	0.38	0.18	1.84E-02	9.60E-02
TIMM21	2515.57	0.38	0.11	1.60E-04	3.58E-03
ASPN	65896.35	0.38	0.67	9.75E-03	6.28E-02
PGGT1B	1774.42	0.38	0.13	1.59E-03	1.82E-02
AASDHPPT	4125.98	0.38	0.18	1.54E-02	8.50E-02
GLS	8583.99	0.38	0.16	9.87E-03	6.33E-02
PGAM1	21174.33	0.38	0.17	1.25E-02	7.35E-02
APOPT1	2217.89	0.38	0.15	5.63E-03	4.33E-02
RBM47	1285.46	0.38	0.74	6.11E-04	9.19E-03
CLK2	1765.68	0.37	0.17	1.15E-02	6.94E-02
COQ4	2462.56	0.37	0.12	9.57E-04	1.27E-02
MED31	1338.44	0.37	0.18	1.83E-02	9.57E-02
GOLGA7	9727.84	0.37	0.14	4.15E-03	3.52E-02
COLGALT1	7694.60	0.37	0.15	7.58E-03	5.33E-02
SPAG9	7502.61	0.37	0.16	1.22E-02	7.23E-02
CLN5	5369.80	0.37	0.16	9.98E-03	6.37E-02
RPAP2	3071.16	0.37	0.16	9.70E-03	6.25E-02
CLSPN	154.64	0.37	0.64	1.70E-02	9.12E-02
CHST1	392.84	0.37	0.65	1.44E-02	8.13E-02
PSMA2	8262.01	0.37	0.16	9.85E-03	6.32E-02
LAMTOR3	4345.93	0.37	0.13	2.50E-03	2.51E-02
ANKRD26	1218.86	0.37	0.17	1.39E-02	7.92E-02
LRFN1	144.56	0.37	0.67	6.90E-03	5.00E-02
SLC30A9	7000.01	0.37	0.13	2.35E-03	2.39E-02
CPXM1	61889.57	0.37	0.63	1.62E-02	8.82E-02
ALDH7A1	4312.54	0.36	0.15	9.85E-03	6.32E-02
UFC1	3598.45	0.36	0.16	1.19E-02	7.13E-02
PPIH	920.78	0.36	0.15	9.40E-03	6.12E-02
TIMM9	1518.84	0.36	0.16	1.07E-02	6.63E-02
HIF1A	15094.08	0.36	0.17	1.74E-02	9.28E-02
PIAS2	3120.96	0.36	0.15	1.00E-02	6.37E-02
PANX2	33.43	0.36	0.72	1.55E-05	6.29E-04
LYPD1	189.59	0.36	0.68	2.48E-03	2.50E-02
HMCN1	14982.38	0.36	0.66	6.43E-03	4.75E-02
TAF12	2494.26	0.35	0.13	3.04E-03	2.83E-02
WISP3	124.71	0.35	0.63	1.45E-02	8.16E-02
ERLIN2	960.31	0.35	0.12	1.39E-03	1.65E-02
DNAAF2	1666.79	0.35	0.15	1.01E-02	6.41E-02
POLR2G	5001.96	0.35	0.15	1.04E-02	6.53E-02
AGPAT2	2948.52	0.35	0.13	3.03E-03	2.83E-02
SUCLG1	5244.80	0.35	0.15	1.16E-02	7.01E-02
ZNF354A	1104.97	0.35	0.12	2.14E-03	2.24E-02
AIFM3	42.31	0.35	0.64	8.81E-03	5.87E-02
PRR36	101.09	0.35	0.65	3.04E-03	2.83E-02
KIAA1024	414.83	0.35	0.63	8.74E-03	5.84E-02
PTPMT1	2837.30	0.35	0.15	1.08E-02	6.65E-02
NFE2L3	852.13	0.34	0.65	2.09E-03	2.20E-02
APOC1	1124.10	0.34	0.67	7.63E-04	1.08E-02
ALG8	2753.25	0.34	0.15	1.18E-02	7.10E-02
SEMA6B	430.06	0.34	0.61	1.70E-02	9.10E-02
CWC15	3936.29	0.34	0.14	7.19E-03	5.15E-02
SMARCAD1	2536.46	0.34	0.15	1.14E-02	6.92E-02
EPYC	4137.16	0.34	0.69	5.26E-11	1.97E-08
ZNF775	812.46	0.34	0.16	1.68E-02	9.04E-02
MYO19	1575.79	0.34	0.15	1.48E-02	8.27E-02
FAM173B	808.12	0.34	0.14	7.97E-03	5.51E-02
MDFI	671.65	0.34	0.62	8.45E-03	5.71E-02
EFNB3	669.79	0.34	0.66	1.90E-04	4.09E-03
GFER	555.34	0.34	0.16	1.76E-02	9.35E-02
EXOSC1	1435.39	0.34	0.16	1.81E-02	9.51E-02
G2E3	1811.98	0.34	0.16	1.77E-02	9.35E-02
SFRP4	132492.23	0.34	0.60	1.31E-02	7.58E-02
CENPM	143.25	0.33	0.62	8.32E-03	5.65E-02
NLGN4X	767.62	0.33	0.65	2.77E-04	5.37E-03
FCHO1	224.31	0.33	0.64	1.04E-03	1.36E-02
DNAJC22	354.29	0.33	0.65	8.77E-05	2.26E-03
LSM8	1932.99	0.33	0.16	1.89E-02	9.75E-02
DDOST	21348.98	0.33	0.15	1.64E-02	8.86E-02
TNFSF11	226.48	0.33	0.64	7.11E-04	1.03E-02
CLIC3	924.67	0.33	0.59	1.83E-02	9.55E-02
SNUPN	2124.61	0.33	0.16	1.88E-02	9.73E-02
SLC35F5	5718.54	0.33	0.14	1.25E-02	7.35E-02
NOL8	2842.20	0.33	0.16	1.94E-02	9.93E-02
AKR7A2	6344.17	0.33	0.14	1.11E-02	6.80E-02
TTK	156.84	0.33	0.61	3.69E-03	3.25E-02
DPH5	736.70	0.32	0.14	1.15E-02	6.94E-02
APOE	53974.53	0.32	0.59	1.27E-02	7.42E-02
WDR92	1072.03	0.32	0.14	1.27E-02	7.43E-02
BFAR	2900.51	0.32	0.12	5.35E-03	4.21E-02
SUMO1	9869.83	0.32	0.09	1.57E-04	3.53E-03
TRMT61B	1319.99	0.32	0.13	1.02E-02	6.46E-02
KRT80	51.42	0.32	0.62	1.28E-04	2.96E-03
ARSE	318.53	0.32	0.59	1.01E-02	6.38E-02
TACC3	439.66	0.32	0.58	1.15E-02	6.94E-02

SCT	4.96	0.32	0.61	4.65E-04	7.70E-03
RNF128	106.17	0.32	0.61	3.02E-04	5.68E-03
BIRC2	6909.52	0.32	0.13	1.20E-02	7.16E-02
RUBCNL	204.75	0.31	0.60	2.46E-03	2.48E-02
EEF1AKMT2	1784.24	0.31	0.14	1.50E-02	8.34E-02
NKPD1	8.32	0.31	0.59	6.26E-03	4.67E-02
HIGD1B	740.45	0.31	0.59	6.80E-03	4.94E-02
TDO2	2953.48	0.31	0.62	1.15E-05	5.00E-04
ADGRF5	5610.68	0.31	0.56	1.91E-02	9.83E-02
COL9A2	1610.75	0.31	0.57	1.28E-02	7.46E-02
MOV10L1	102.58	0.31	0.58	1.05E-02	6.56E-02
LRRC66	19.47	0.31	0.57	1.55E-02	8.56E-02
SPAG17	42.79	0.31	0.58	6.35E-03	4.71E-02
TFCP2	1376.77	0.31	0.11	2.09E-03	2.20E-02
USP34	3414.95	0.31	0.11	2.24E-03	2.31E-02
FAM136A	2725.86	0.31	0.13	9.29E-03	6.07E-02
GALNT14	708.18	0.31	0.59	1.32E-03	1.59E-02
CPA4	472.50	0.31	0.59	1.08E-03	1.39E-02
SPCS1	7968.06	0.31	0.13	1.25E-02	7.33E-02
CPNE7	76.37	0.31	0.60	2.45E-04	4.92E-03
TRAPPC5	3312.22	0.30	0.14	1.79E-02	9.44E-02
BEAN1	339.23	0.30	0.57	9.96E-03	6.36E-02
UFL1	4740.99	0.30	0.13	1.40E-02	7.95E-02
TSPAN13	2564.38	0.30	0.57	8.27E-03	5.64E-02
TMPRSS3	10.59	0.30	0.56	1.65E-02	8.91E-02
TAF10	4043.67	0.30	0.11	5.46E-03	4.25E-02
HOXA1	80.55	0.30	0.55	1.44E-02	8.12E-02
GPR158	79.93	0.30	0.57	1.79E-03	1.97E-02
TMEM147	6016.12	0.29	0.13	1.75E-02	9.29E-02
SOX30	38.53	0.29	0.54	1.93E-02	9.92E-02
FLRT3	450.56	0.29	0.54	1.59E-02	8.70E-02
CDCP1	159.00	0.29	0.56	2.07E-03	2.19E-02
NEBL	102.58	0.29	0.54	1.77E-02	9.38E-02
TRIM14	603.69	0.29	0.54	1.19E-02	7.11E-02
PTPN4	1357.09	0.29	0.12	1.35E-02	7.77E-02
AGAP2	46.57	0.28	0.54	1.35E-02	7.76E-02
CEP55	253.75	0.28	0.54	1.43E-02	8.08E-02
GTSE1	175.68	0.28	0.54	1.15E-02	6.94E-02
COL10A1	581.23	0.28	0.55	1.32E-03	1.59E-02
SKA3	117.19	0.28	0.54	4.90E-03	3.96E-02
TMEM163	74.17	0.28	0.53	9.81E-03	6.31E-02
APLN	1004.81	0.28	0.53	1.23E-02	7.27E-02
CLNS1A	3048.66	0.28	0.13	1.94E-02	9.94E-02
ASPHD1	152.86	0.28	0.55	7.39E-04	1.06E-02
RFLNA	798.90	0.27	0.53	9.11E-03	6.00E-02
TESMIN	24.54	0.27	0.52	1.90E-02	9.81E-02
UHRF1	501.21	0.27	0.52	1.69E-02	9.06E-02
KRT18	3828.70	0.27	0.54	1.10E-03	1.41E-02
KIAA1324L	17670.34	0.27	0.53	3.71E-03	3.26E-02
SNX10	4617.01	0.27	0.55	5.49E-06	2.89E-04
DIRC1	35.76	0.27	0.53	1.60E-03	1.82E-02
TSNAX	4278.23	0.27	0.12	1.51E-02	8.38E-02
PLPPR3	18.18	0.26	0.52	8.83E-03	5.88E-02
SIDT1	103.31	0.26	0.53	3.86E-04	6.77E-03
PLAC1	39.33	0.26	0.53	1.03E-03	1.35E-02
KIAA1211L	371.86	0.26	0.51	1.32E-02	7.60E-02
USP43	55.36	0.26	0.51	8.92E-03	5.92E-02
MAGED4	787.97	0.26	0.51	1.65E-02	8.90E-02
TMEM26	548.34	0.25	0.52	5.33E-04	8.41E-03
PARN	3837.24	0.25	0.10	6.58E-03	4.83E-02
RAB27B	327.03	0.25	0.51	2.10E-03	2.20E-02
C10orf90	20.72	0.25	0.51	4.43E-03	3.70E-02
P2RY6	178.10	0.25	0.52	1.36E-06	1.03E-04
CD7	179.86	0.25	0.52	4.29E-05	1.36E-03
IL19	27.64	0.25	0.52	3.99E-04	6.94E-03
DSC2	1147.12	0.25	0.51	3.72E-04	6.62E-03
PDRG1	1125.58	0.25	0.09	2.85E-03	2.71E-02
EPHX3	109.06	0.25	0.50	1.65E-02	8.91E-02
TMEM92	175.01	0.25	0.51	4.12E-03	3.51E-02
GIP	42.74	0.25	0.52	3.35E-07	3.52E-05
POU2F3	35.56	0.24	0.50	7.83E-03	5.45E-02
FAM19A3	99.62	0.24	0.51	7.27E-04	1.05E-02
VTN	1163.41	0.24	0.50	8.61E-03	5.79E-02
MCHR1	99.97	0.24	0.51	1.80E-06	1.27E-04
NRG1	358.67	0.24	0.51	4.80E-05	1.47E-03
LRRC43	27.74	0.24	0.49	1.11E-02	6.77E-02
HMMR	226.43	0.24	0.49	1.19E-02	7.11E-02
M1AP	19.74	0.24	0.49	7.84E-03	5.45E-02
RAC2	2348.63	0.24	0.50	4.53E-06	2.52E-04
CLDN16	63.07	0.24	0.50	7.96E-04	1.11E-02
TOX	189.98	0.23	0.50	1.35E-03	1.62E-02
CALB2	424.49	0.23	0.50	9.27E-04	1.24E-02
F2RL1	169.36	0.23	0.48	1.58E-02	8.66E-02
TNIP3	15.01	0.23	0.49	3.39E-03	3.05E-02
CAMK2N2	11.99	0.23	0.49	2.40E-03	2.44E-02
INHA	20.06	0.23	0.48	1.21E-02	7.20E-02
HRASLS	26.23	0.23	0.49	7.48E-03	5.28E-02
HSD17B6	236.95	0.23	0.49	2.16E-03	2.25E-02
AQP5	130.04	0.23	0.48	9.87E-03	6.33E-02
CLDN1	9051.93	0.23	0.49	7.80E-04	1.10E-02
GALNT13	3407.91	0.23	0.48	7.21E-03	5.15E-02
C7orf57	12.27	0.23	0.48	4.61E-03	3.80E-02
FXYD7	76.44	0.23	0.48	1.90E-02	9.81E-02
NKX2-3	648.78	0.22	0.49	1.35E-05	5.69E-04
EPCAM	9.70	0.22	0.48	9.12E-03	6.00E-02
ELFN1	451.61	0.22	0.48	4.37E-04	7.36E-03
PPP2R5C	6776.65	0.22	0.09	1.45E-02	8.15E-02
SPATA8	8.31	0.22	0.48	4.59E-03	3.79E-02
AIM2	29.34	0.22	0.48	3.14E-03	2.89E-02
PRSS21	20.29	0.22	0.48	1.18E-04	2.80E-03
CPLX1	125.04	0.22	0.47	7.11E-03	5.10E-02
BDH1	247.98	0.22	0.47	9.21E-03	6.04E-02

MARCO	283.56	0.21	0.47	3.17E-03	2.91E-02
LIX1	54.46	0.21	0.48	3.94E-06	2.31E-04
OPN4	61.26	0.21	0.46	1.40E-02	7.98E-02
REC8	1171.51	0.21	0.47	1.92E-03	2.08E-02
IL1R2	233.32	0.20	0.47	1.35E-03	1.62E-02
GRM3	42.61	0.20	0.47	1.19E-03	1.48E-02
KLK14	78.98	0.20	0.46	1.23E-02	7.27E-02
IGFL2	71.46	0.20	0.47	4.22E-05	1.35E-03
GSTM1	1845.23	0.20	0.46	9.06E-04	1.22E-02
WNK4	249.85	0.20	0.46	7.54E-03	5.32E-02
HHIPL2	186.20	0.20	0.45	1.82E-02	9.54E-02
HIST1H3C	3.42	0.20	0.45	1.50E-02	8.34E-02
RAB4A	4440.65	0.20	0.08	9.04E-03	5.97E-02
DSC3	728.72	0.20	0.46	3.72E-03	3.27E-02
LRRN4	630.05	0.19	0.46	9.96E-05	2.51E-03
NKX3-2	723.27	0.19	0.46	2.61E-03	2.58E-02
SLC22A18AS	46.17	0.19	0.45	1.61E-02	8.77E-02
KRT39	16.62	0.19	0.45	7.29E-03	5.19E-02
SPINT1	786.47	0.19	0.45	8.38E-04	1.15E-02
MUC3A	81.65	0.19	0.45	3.21E-03	2.93E-02
CALB1	279.69	0.19	0.45	6.58E-03	4.83E-02
OTOF	38.70	0.18	0.45	2.69E-04	5.25E-03
TAS2R10	6.23	0.18	0.44	5.19E-03	4.13E-02
ALK	9.33	0.18	0.44	1.45E-02	8.17E-02
ISL2	113.42	0.17	0.44	5.08E-04	8.12E-03
DMKN	2351.18	0.17	0.44	8.66E-03	5.81E-02
IL10	1028.03	0.17	0.44	2.14E-05	8.03E-04
LMNTD2	71.77	0.17	0.44	4.10E-03	3.50E-02
POSTN	163371.21	0.17	0.43	1.43E-02	8.09E-02
CXADR	776.42	0.17	0.43	1.76E-02	9.33E-02
LGALS12	142.07	0.16	0.43	2.48E-03	2.50E-02
GJA9	10.30	0.16	0.44	3.16E-04	5.89E-03
WFIKKN1	165.20	0.16	0.43	1.33E-02	7.64E-02
ALOX15B	104.22	0.16	0.43	4.05E-04	6.99E-03
CHRM2	347.62	0.16	0.43	1.01E-02	6.41E-02
CXCL13	28.11	0.16	0.43	1.50E-05	6.09E-04
TNNT1	65.22	0.16	0.43	5.94E-04	9.06E-03
TCERG1L	53.34	0.16	0.43	2.47E-04	4.95E-03
STXBP2	584.51	0.16	0.43	3.39E-04	6.20E-03
FAM83H	42.77	0.16	0.43	1.43E-03	1.68E-02
CA9	495.01	0.16	0.43	7.06E-05	1.99E-03
KRT8	6689.99	0.16	0.43	7.00E-04	1.02E-02
BRINP2	92.03	0.16	0.43	2.76E-06	1.74E-04
KISS1R	3.69	0.15	0.43	4.91E-03	3.97E-02
KLHL31	23.16	0.15	0.43	8.03E-03	5.54E-02
F5	2504.15	0.15	0.43	1.36E-02	7.83E-02
MYRF	1077.22	0.15	0.43	1.09E-03	1.40E-02
ADAMTS19	253.38	0.15	0.42	1.86E-02	9.68E-02
CCL22	288.80	0.15	0.43	1.15E-02	6.98E-02
P2RY10	57.45	0.15	0.42	1.06E-02	6.57E-02
ILDR2	581.77	0.15	0.43	6.69E-03	4.88E-02
IGFBP2	49074.57	0.15	0.42	7.37E-03	5.22E-02
NOTUM	202.51	0.15	0.43	5.80E-04	8.94E-03
PALM3	16.06	0.15	0.42	2.70E-03	2.63E-02
EGFL6	8086.16	0.15	0.42	5.42E-03	4.24E-02
CBLN2	90.00	0.14	0.42	8.81E-04	1.20E-02
PRKCQ	100.08	0.14	0.42	7.15E-05	2.00E-03
DSC1	17.64	0.14	0.42	2.06E-03	2.18E-02
NEFL	590.08	0.14	0.42	9.50E-03	6.15E-02
CCDC68	205.98	0.14	0.42	1.78E-02	9.40E-02
NXPH2	13.93	0.14	0.42	2.41E-04	4.85E-03
TNN	1483.64	0.14	0.42	2.78E-03	2.67E-02
USH2A	7.04	0.14	0.42	1.88E-02	9.73E-02
TREM1	226.78	0.14	0.42	1.13E-03	1.43E-02
WNT4	11124.99	0.13	0.42	1.54E-03	1.77E-02
SLC46A2	77.18	0.13	0.42	2.13E-04	4.42E-03
KLK7	161.96	0.13	0.41	1.64E-02	8.87E-02
PCSK9	92.83	0.13	0.41	1.35E-02	7.76E-02
BCL11B	48.55	0.13	0.41	1.91E-02	9.82E-02
DRC1	16.85	0.13	0.41	1.07E-02	6.64E-02
IL13RA2	409.20	0.12	0.41	2.24E-03	2.31E-02
NR1I2	7.00	0.12	0.41	2.29E-03	2.35E-02
KLK4	87.99	0.12	0.41	4.58E-03	3.79E-02
RGPD4	11.01	0.12	0.41	1.94E-02	9.93E-02
FASLG	46.91	0.12	0.41	1.43E-05	5.89E-04
SP7	250.83	0.12	0.41	2.80E-03	2.68E-02
NR1H4	45.13	0.12	0.41	8.65E-04	1.18E-02
ATG9B	145.25	0.12	0.41	7.01E-03	5.04E-02
ABCA12	8.72	0.11	0.41	7.08E-03	5.08E-02
ARMC3	4.21	0.11	0.41	8.33E-03	5.65E-02
FOLR3	77.23	0.11	0.41	8.12E-03	5.58E-02
TFAP2C	188.50	0.11	0.41	5.09E-03	4.06E-02
IL5	4.26	0.11	0.40	8.28E-03	5.64E-02
CRB2	174.74	0.11	0.40	1.81E-02	9.51E-02
TRIM71	109.98	0.11	0.40	2.69E-03	2.62E-02
ASTL	8.55	0.11	0.40	2.07E-03	2.19E-02
SOWAHD	13.49	0.10	0.40	3.82E-03	3.33E-02
HIST1H2AJ	15.33	0.10	0.40	6.92E-04	1.02E-02
GBX2	7.66	0.10	0.40	3.38E-03	3.04E-02
CYP2J2	14.64	0.10	0.40	1.02E-02	6.45E-02
CXCR3	92.82	0.10	0.40	3.17E-03	2.91E-02
SLC9C2	11.11	0.10	0.40	1.78E-03	1.97E-02
CFAP46	19.06	0.10	0.40	6.77E-03	4.93E-02
SLC30A10	146.88	0.10	0.40	1.83E-03	2.01E-02
HIST1H2AB	5.83	0.10	0.40	7.00E-03	5.04E-02
GZMM	24.49	0.09	0.40	3.01E-04	5.67E-03
CARD11	58.29	0.09	0.40	5.86E-04	9.00E-03
SBK2	10.56	0.09	0.40	2.14E-03	2.23E-02
RFPL1	10.13	0.09	0.40	2.52E-03	2.53E-02
VNN1	693.50	0.09	0.40	4.38E-04	7.36E-03
ST8SIA6	68.14	0.09	0.40	1.29E-02	7.50E-02
INA	11.12	0.09	0.40	9.40E-03	6.12E-02

KIRREL2	158.08	0.09	0.40	7.48E-05	2.06E-03
ADGRE1	31.95	0.09	0.40	3.03E-03	2.83E-02
GRP	8.45	0.08	0.39	5.02E-03	4.02E-02
EPGN	68.89	0.08	0.39	1.53E-02	8.48E-02
RORC	68.25	0.08	0.39	2.23E-03	2.31E-02
CD244	63.94	0.08	0.39	2.79E-03	2.68E-02
CCR2	86.20	0.08	0.39	9.94E-03	6.36E-02
ADGRF4	15.60	0.07	0.39	9.07E-03	5.99E-02
KCNK4	22.52	0.07	0.39	1.71E-03	1.91E-02
FRMPD4	7.66	0.07	0.39	1.23E-02	7.25E-02
FAM216B	221.62	0.07	0.39	8.29E-03	5.64E-02
OTOS	5.88	0.06	0.39	1.93E-02	9.90E-02
SCEL	16.79	0.06	0.39	3.48E-03	3.10E-02
CFC1	11.73	0.06	0.39	6.89E-03	4.99E-02
PRR9	8.25	0.06	0.39	1.69E-02	9.06E-02
ASGR2	27.10	0.05	0.39	8.96E-03	5.94E-02
XCR1	15.98	0.05	0.39	8.53E-03	5.76E-02
HAPLN1	21.32	0.05	0.39	1.61E-02	8.77E-02
TIMD4	25.31	0.05	0.39	1.47E-02	8.21E-02
SPHAR	46.33	0.04	0.38	5.31E-03	4.19E-02
TBC1D3C	45.51	0.03	0.38	6.39E-03	4.73E-02
USH1G	33.12	0.03	0.38	1.18E-02	7.09E-02
TOX3	8.26	-0.10	0.40	9.36E-03	6.10E-02
OPRK1	6.51	-0.13	0.41	1.74E-02	9.28E-02
SOST	21.35	-0.14	0.42	1.89E-02	9.75E-02
OCA2	89.09	-0.16	0.43	1.38E-02	7.89E-02
OSTN	86.89	-0.16	0.43	1.32E-02	7.62E-02
ALLC	3.88	-0.17	0.44	9.73E-03	6.27E-02
NUP85	2810.14	-0.19	0.08	1.80E-02	9.45E-02
NUPR2	16.49	-0.19	0.45	1.75E-02	9.29E-02
GRIK5	40.07	-0.19	0.45	1.67E-02	8.99E-02
SPRR2A	65.01	-0.19	0.45	1.39E-02	7.94E-02
SNTG2	17.99	-0.19	0.46	5.09E-03	4.06E-02
TP53TG3	8.76	-0.20	0.46	8.07E-03	5.56E-02
MRPS26	2889.57	-0.21	0.09	1.44E-02	8.13E-02
PDCD5	3821.12	-0.21	0.08	6.10E-03	4.58E-02
SLC8A2	16.90	-0.21	0.47	1.38E-02	7.89E-02
CIDEA	7.52	-0.21	0.47	2.65E-03	2.61E-02
CPNE6	49.09	-0.22	0.48	1.32E-02	7.61E-02
TRIM44	7792.27	-0.22	0.10	1.61E-02	8.77E-02
PTPN5	28.34	-0.23	0.49	1.26E-02	7.39E-02
FBN3	37.95	-0.23	0.49	1.93E-02	9.90E-02
ARFRP1	4613.46	-0.24	0.10	9.04E-03	5.97E-02
ARIH2	5572.97	-0.24	0.11	1.55E-02	8.56E-02
TRIM37	1992.03	-0.25	0.08	1.53E-03	1.77E-02
RRM2B	3173.43	-0.25	0.11	1.62E-02	8.79E-02
VIM	182513.46	-0.26	0.09	2.68E-03	2.62E-02
PRPH	1001.70	-0.26	0.52	1.59E-02	8.70E-02
ATG13	7065.31	-0.26	0.11	1.21E-02	7.21E-02
TARDBP	7968.88	-0.26	0.11	1.29E-02	7.50E-02
TCF15	258.53	-0.26	0.52	1.85E-02	9.63E-02
CDK9	3869.67	-0.27	0.12	1.69E-02	9.06E-02
TPCN2	1665.40	-0.27	0.11	8.62E-03	5.79E-02
SMU1	4806.36	-0.28	0.08	5.04E-04	8.10E-03
PSMD11	6915.11	-0.28	0.08	5.92E-04	9.05E-03
RRP1	2058.90	-0.28	0.12	1.75E-02	9.29E-02
EWSR1	18918.05	-0.28	0.13	1.88E-02	9.74E-02
CTNNA1	17566.62	-0.29	0.07	6.01E-05	1.75E-03
SLC35A1	3354.52	-0.29	0.13	1.66E-02	8.92E-02
CC2D1A	2514.50	-0.29	0.11	8.58E-03	5.78E-02
CDH4	81.33	-0.29	0.55	1.47E-02	8.22E-02
GPS1	6217.63	-0.29	0.11	7.89E-03	5.47E-02
SERINC3	22438.63	-0.29	0.13	1.60E-02	8.74E-02
TTC21B	1760.73	-0.30	0.13	1.83E-02	9.56E-02
ZFAND4	631.40	-0.30	0.11	4.37E-03	3.66E-02
DYNC1LI2	9621.39	-0.30	0.12	1.03E-02	6.50E-02
CDK5RAP2	2736.63	-0.30	0.10	2.54E-03	2.54E-02
PRKAR2A	3429.04	-0.30	0.13	1.17E-02	7.02E-02
ZXDA	617.60	-0.30	0.13	1.08E-02	6.65E-02
WRNIP1	3441.93	-0.30	0.14	1.80E-02	9.47E-02
RBM4	7486.71	-0.31	0.11	2.84E-03	2.71E-02
CHM	2281.86	-0.31	0.14	1.94E-02	9.93E-02
GTF3C5	2983.67	-0.31	0.12	6.51E-03	4.80E-02
SF3B4	6129.19	-0.31	0.14	1.58E-02	8.66E-02
DCTN2	10199.55	-0.31	0.11	1.92E-03	2.08E-02
UIMC1	1384.69	-0.32	0.15	1.91E-02	9.84E-02
SEC22C	4969.84	-0.32	0.14	1.82E-02	9.53E-02
SEC24C	6239.98	-0.32	0.14	1.52E-02	8.43E-02
HUWE1	4469.42	-0.32	0.12	5.30E-03	4.18E-02
CYB5R1	3369.30	-0.32	0.15	1.77E-02	9.35E-02
TBX1	220.91	-0.32	0.60	1.08E-02	6.67E-02
SART3	2699.71	-0.32	0.15	1.93E-02	9.90E-02
NMT1	4229.67	-0.32	0.14	1.07E-02	6.63E-02
DNAJB12	5094.68	-0.32	0.14	1.44E-02	8.14E-02
TRIM28	7711.64	-0.32	0.12	5.59E-03	4.32E-02
PIGO	2167.72	-0.33	0.13	7.64E-03	5.37E-02
BRD7	3271.65	-0.33	0.12	3.49E-03	3.11E-02
DDC	4.39	-0.33	0.61	1.57E-02	8.62E-02
GIGYF2	3986.93	-0.33	0.12	3.57E-03	3.17E-02
ALKBH1	894.74	-0.33	0.15	1.62E-02	8.82E-02
YLPM1	4392.43	-0.33	0.15	1.81E-02	9.51E-02
TAF1C	2972.77	-0.34	0.12	3.58E-03	3.17E-02
CGGBP1	9314.36	-0.34	0.14	1.06E-02	6.57E-02
PSKH1	3367.14	-0.34	0.16	1.86E-02	9.66E-02
UBR3	3101.22	-0.34	0.12	2.97E-03	2.79E-02
KCNK5	195.18	-0.34	0.63	1.39E-02	7.92E-02
AQR	3222.03	-0.34	0.13	5.43E-03	4.25E-02
EIF4ENIF1	1825.45	-0.34	0.11	9.67E-04	1.28E-02
VPS72	3078.10	-0.34	0.14	9.08E-03	5.99E-02
PRKRA	5216.47	-0.34	0.15	1.19E-02	7.11E-02
AIP	3142.54	-0.34	0.14	9.85E-03	6.32E-02
TMUB2	4505.89	-0.35	0.16	1.88E-02	9.72E-02

MTRR	3114.18	-0.35	0.15	1.43E-02	8.09E-02
LEP	13.66	-0.35	0.65	1.17E-02	7.04E-02
NFRKB	2244.78	-0.35	0.15	1.15E-02	6.94E-02
HCRTR1	15.33	-0.35	0.65	1.07E-02	6.64E-02
RRP8	1470.02	-0.35	0.13	3.88E-03	3.36E-02
ARAF	5349.50	-0.35	0.13	4.90E-03	3.96E-02
NELFE	4943.02	-0.35	0.10	1.80E-04	3.95E-03
PCID2	3275.34	-0.35	0.13	4.66E-03	3.83E-02
PPP2R1A	19272.22	-0.36	0.13	3.37E-03	3.04E-02
TMEM259	12896.24	-0.36	0.11	1.11E-03	1.42E-02
SART1	4482.90	-0.36	0.14	6.60E-03	4.84E-02
TAF9B	3255.77	-0.36	0.15	7.55E-03	5.32E-02
CNST	3349.35	-0.36	0.15	7.93E-03	5.49E-02
MINK1	3545.58	-0.36	0.16	1.54E-02	8.50E-02
TOX4	4168.69	-0.36	0.16	1.22E-02	7.23E-02
ARMC2	469.28	-0.36	0.16	1.15E-02	6.94E-02
DOK7	27.74	-0.36	0.67	1.49E-02	8.30E-02
ATG4B	4217.38	-0.37	0.11	7.04E-04	1.03E-02
SHPK	1570.46	-0.37	0.17	1.44E-02	8.13E-02
GPD2	1592.37	-0.37	0.17	1.38E-02	7.90E-02
DDX54	3198.30	-0.37	0.13	2.85E-03	2.71E-02
PEF1	5802.60	-0.37	0.17	1.67E-02	8.97E-02
PIGU	1819.06	-0.38	0.16	1.13E-02	6.89E-02
CLIP1	8792.12	-0.38	0.17	1.50E-02	8.35E-02
XPO7	2724.87	-0.38	0.15	7.74E-03	5.41E-02
TANGO6	1082.45	-0.38	0.14	4.15E-03	3.52E-02
KANK3	1773.21	-0.38	0.68	1.85E-02	9.62E-02
SAP30BP	4657.59	-0.38	0.13	1.46E-03	1.71E-02
MAPK1IP1L	11131.87	-0.38	0.14	3.11E-03	2.87E-02
ANKRD11	7705.73	-0.38	0.12	8.47E-04	1.16E-02
PPP4R3A	6246.38	-0.38	0.16	9.97E-03	6.36E-02
DDX19A	3656.27	-0.38	0.09	5.24E-06	2.80E-04
ANKRD12	7007.13	-0.38	0.16	8.73E-03	5.83E-02
LVRN	632.50	-0.38	0.70	1.62E-02	8.82E-02
ARFGAP2	5292.73	-0.38	0.12	7.53E-04	1.07E-02
CBLN1	37.25	-0.38	0.70	1.58E-02	8.67E-02
MTR	3632.81	-0.38	0.16	7.31E-03	5.19E-02
PFAS	1593.24	-0.39	0.19	1.93E-02	9.92E-02
NFYB	3865.69	-0.39	0.15	6.14E-03	4.61E-02
USP10	5636.34	-0.39	0.15	5.21E-03	4.14E-02
NSFL1C	6022.20	-0.39	0.11	2.06E-04	4.35E-03
AAMP	7691.83	-0.39	0.15	5.94E-03	4.50E-02
ACADVL	34530.72	-0.39	0.18	1.84E-02	9.62E-02
MAU2	4059.61	-0.39	0.14	2.94E-03	2.77E-02
RALGAPB	3031.28	-0.39	0.12	5.61E-04	8.73E-03
PPP6C	4952.51	-0.39	0.14	3.07E-03	2.86E-02
PUM1	10822.72	-0.39	0.15	4.83E-03	3.93E-02
MFSD10	6053.66	-0.39	0.17	1.09E-02	6.73E-02
POLR2E	11242.44	-0.39	0.13	1.93E-03	2.08E-02
TRMT12	588.39	-0.39	0.15	5.32E-03	4.19E-02
RAD54L2	2386.34	-0.39	0.13	1.84E-03	2.01E-02
TM7SF3	5103.75	-0.39	0.16	7.24E-03	5.17E-02
PPP5C	3451.41	-0.39	0.13	1.62E-03	1.84E-02
BORCS5	828.18	-0.39	0.17	1.23E-02	7.25E-02
HRAS	1457.67	-0.39	0.19	1.71E-02	9.14E-02
RNF41	2513.93	-0.39	0.15	5.46E-03	4.25E-02
MARK2	3189.36	-0.40	0.18	1.38E-02	7.90E-02
RRP15	1085.81	-0.40	0.18	1.43E-02	8.08E-02
USP19	4483.23	-0.40	0.18	1.45E-02	8.14E-02
CCT7	11204.65	-0.40	0.15	3.82E-03	3.33E-02
SNRPA	2170.61	-0.40	0.19	1.67E-02	8.97E-02
XRCC6	21447.58	-0.40	0.08	8.63E-07	7.12E-05
GEMIN5	1652.89	-0.40	0.19	1.87E-02	9.68E-02
KMT2D	1382.86	-0.40	0.16	5.69E-03	4.37E-02
KIF1BP	4641.35	-0.40	0.17	9.81E-03	6.31E-02
MED24	3561.24	-0.40	0.15	3.87E-03	3.36E-02
ELOC	4999.13	-0.40	0.17	7.69E-03	5.39E-02
NHLRC2	2817.87	-0.40	0.18	1.32E-02	7.60E-02
REPS1	2558.02	-0.40	0.15	2.99E-03	2.80E-02
LARP1B	1614.90	-0.40	0.20	1.95E-02	9.97E-02
WASHC2C	4858.37	-0.41	0.15	4.18E-03	3.54E-02
RBM7	5706.93	-0.41	0.13	1.22E-03	1.51E-02
ZNF777	1583.10	-0.41	0.18	1.28E-02	7.47E-02
ABCF1	3232.27	-0.41	0.15	2.89E-03	2.73E-02
SEH1L	3119.38	-0.41	0.16	4.50E-03	3.74E-02
STRN4	6691.04	-0.41	0.11	1.17E-04	2.79E-03
CDC73	5310.78	-0.41	0.16	6.02E-03	4.55E-02
GET4	2486.98	-0.41	0.20	1.91E-02	9.81E-02
ZNF593	811.58	-0.41	0.19	1.54E-02	8.50E-02
CYP51A1	4430.83	-0.41	0.19	1.63E-02	8.83E-02
GTPBP6	3186.16	-0.41	0.20	1.83E-02	9.56E-02
CELF1	7017.86	-0.41	0.13	4.87E-04	7.92E-03
POC1B	1423.35	-0.41	0.20	1.96E-02	9.99E-02
ZBTB7A	6181.44	-0.41	0.18	1.00E-02	6.38E-02
UBE3B	3416.12	-0.42	0.12	2.16E-04	4.45E-03
MED30	1813.70	-0.42	0.18	8.24E-03	5.64E-02
ELMO2	5418.05	-0.42	0.17	7.36E-03	5.21E-02
RRP9	1024.91	-0.42	0.19	1.23E-02	7.27E-02
LRWD1	1230.50	-0.42	0.16	3.57E-03	3.17E-02
POU6F1	652.82	-0.42	0.17	7.33E-03	5.20E-02
ACAT1	7179.25	-0.42	0.20	1.78E-02	9.42E-02
TINF2	5386.53	-0.42	0.12	1.81E-04	3.95E-03
ASB6	2171.36	-0.42	0.16	3.57E-03	3.17E-02
ETFDH	2223.90	-0.42	0.16	3.93E-03	3.40E-02
EFCAB14	13611.98	-0.42	0.15	1.98E-03	2.12E-02
CUL1	5996.15	-0.42	0.10	2.24E-05	8.25E-04
PCIF1	5210.54	-0.42	0.21	1.79E-02	9.43E-02
PPP3CC	3037.85	-0.43	0.21	1.94E-02	9.92E-02
NELFCD	5256.22	-0.43	0.19	1.09E-02	6.73E-02
DIMT1	3300.33	-0.43	0.18	9.44E-03	6.14E-02
BAG1	4643.30	-0.43	0.18	7.72E-03	5.40E-02
TBRG1	3356.01	-0.43	0.13	3.52E-04	6.37E-03

DDX24	11739.94	-0.43	0.15	2.56E-03	2.55E-02
MRPL38	3865.26	-0.43	0.13	5.08E-04	8.12E-03
MED29	5845.41	-0.43	0.14	1.05E-03	1.37E-02
NR2C2AP	665.09	-0.43	0.18	7.98E-03	5.51E-02
ZNF76	2324.35	-0.43	0.18	7.91E-03	5.48E-02
ELOVL5	7036.39	-0.43	0.15	1.50E-03	1.75E-02
FAM120AOS	5562.44	-0.43	0.17	5.70E-03	4.37E-02
CDIPT	9141.54	-0.43	0.11	3.68E-05	1.21E-03
DUSP22	3691.05	-0.43	0.21	1.86E-02	9.67E-02
RBBP7	10902.47	-0.43	0.14	1.38E-03	1.65E-02
POR	6563.42	-0.43	0.17	5.53E-03	4.29E-02
GPR108	5937.21	-0.43	0.20	1.26E-02	7.39E-02
SRRT	4628.06	-0.43	0.16	2.96E-03	2.79E-02
ANKHD1	8021.34	-0.44	0.17	5.73E-03	4.38E-02
AK9	450.42	-0.44	0.16	2.62E-03	2.59E-02
NAPA	8387.73	-0.44	0.20	1.17E-02	7.05E-02
PTEN	12355.39	-0.44	0.19	8.10E-03	5.58E-02
AKAP8	2735.19	-0.44	0.16	2.94E-03	2.77E-02
ZNF333	2124.46	-0.44	0.19	9.99E-03	6.37E-02
DNAJA3	3268.17	-0.44	0.17	4.40E-03	3.68E-02
SARS	9953.39	-0.44	0.15	1.97E-03	2.11E-02
B4GALT3	2635.21	-0.44	0.21	1.42E-02	8.02E-02
CTBP2	5416.24	-0.44	0.22	1.84E-02	9.60E-02
RRN3	3399.20	-0.44	0.16	3.08E-03	2.86E-02
FBXO33	2065.38	-0.44	0.16	3.11E-03	2.87E-02
MUS81	3101.17	-0.44	0.22	1.90E-02	9.78E-02
CPSF4	1356.70	-0.44	0.21	1.63E-02	8.82E-02
NAT10	2827.13	-0.44	0.16	2.64E-03	2.60E-02
SELENOO	1843.80	-0.44	0.21	1.57E-02	8.63E-02
CCDC97	3187.18	-0.45	0.20	1.24E-02	7.32E-02
HMBOX1	3326.82	-0.45	0.20	1.12E-02	6.81E-02
TJAP1	1660.06	-0.45	0.21	1.39E-02	7.92E-02
SF3A1	6208.58	-0.45	0.22	1.57E-02	8.63E-02
LARP4	3090.83	-0.45	0.22	1.61E-02	8.77E-02
GPKOW	1746.81	-0.45	0.17	3.05E-03	2.84E-02
DHX8	2319.25	-0.45	0.21	1.26E-02	7.40E-02
TTBK2	1599.97	-0.45	0.22	1.76E-02	9.33E-02
TOPORS	4429.58	-0.45	0.17	3.88E-03	3.36E-02
PRPF3	2740.94	-0.45	0.22	1.54E-02	8.51E-02
APH1B	2486.55	-0.45	0.17	4.14E-03	3.52E-02
ETS2	9336.33	-0.45	0.85	1.80E-02	9.48E-02
ZNF335	1969.80	-0.45	0.18	4.52E-03	3.75E-02
UTP4	2878.05	-0.45	0.16	2.09E-03	2.20E-02
NELFA	2518.38	-0.46	0.17	2.67E-03	2.62E-02
MTMR3	3993.66	-0.46	0.21	1.29E-02	7.48E-02
ZBTB40	1621.95	-0.46	0.18	5.51E-03	4.28E-02
SDHA	10230.71	-0.46	0.15	1.05E-03	1.37E-02
GAK	4816.59	-0.46	0.15	8.42E-04	1.16E-02
ATF4	14677.23	-0.46	0.20	7.91E-03	5.49E-02
ACTR5	704.72	-0.46	0.16	2.30E-03	2.36E-02
CRAMP1	1434.37	-0.46	0.23	1.75E-02	9.29E-02
DHX15	8052.91	-0.46	0.15	8.96E-04	1.22E-02
PSMF1	9828.59	-0.46	0.17	3.13E-03	2.89E-02
COX20	3142.41	-0.46	0.22	1.44E-02	8.13E-02
BBX	6539.53	-0.46	0.20	9.18E-03	6.03E-02
VEZF1	5011.21	-0.46	0.20	9.65E-03	6.23E-02
SENP2	2113.87	-0.46	0.11	1.69E-05	6.72E-04
RBBP6	5479.12	-0.46	0.22	1.36E-02	7.82E-02
RAB11B	11143.21	-0.46	0.19	6.04E-03	4.55E-02
ECSIT	1799.94	-0.46	0.16	1.47E-03	1.71E-02
EXOC7	9075.19	-0.46	0.21	1.03E-02	6.50E-02
AATF	2709.70	-0.46	0.16	2.10E-03	2.20E-02
PIN1	4763.45	-0.46	0.17	2.80E-03	2.68E-02
RNF123	2332.45	-0.46	0.10	2.79E-06	1.75E-04
FBXO31	3299.79	-0.46	0.22	1.33E-02	7.63E-02
CYB5D2	2157.02	-0.46	0.19	6.75E-03	4.91E-02
HNRNPU	31294.23	-0.46	0.18	4.93E-03	3.97E-02
POLR3E	1433.77	-0.46	0.17	3.17E-03	2.91E-02
RBM42	7440.12	-0.46	0.12	4.76E-05	1.47E-03
IARS2	6573.44	-0.46	0.17	3.16E-03	2.90E-02
ZNF143	1595.51	-0.47	0.16	1.68E-03	1.88E-02
UBE3A	8138.23	-0.47	0.15	1.18E-03	1.48E-02
ATF7	5535.14	-0.47	0.23	1.49E-02	8.33E-02
PPA1	5150.44	-0.47	0.22	1.40E-02	7.98E-02
DHRS7	7295.12	-0.47	0.20	7.41E-03	5.24E-02
ULK2	3530.57	-0.47	0.22	1.20E-02	7.16E-02
SLC25A28	2009.85	-0.47	0.20	7.87E-03	5.47E-02
BRPF1	1626.64	-0.47	0.17	2.74E-03	2.65E-02
TRIM39	1167.46	-0.47	0.20	7.64E-03	5.37E-02
ERCC3	4245.97	-0.47	0.11	1.78E-05	6.98E-04
RNPS1	12341.14	-0.47	0.15	1.05E-03	1.36E-02
PRPF6	6788.78	-0.47	0.15	9.62E-04	1.27E-02
UCKL1	2658.30	-0.47	0.16	1.35E-03	1.62E-02
LIN7C	5557.57	-0.47	0.16	1.72E-03	1.91E-02
EDC4	4481.67	-0.47	0.14	3.84E-04	6.75E-03
RRP1B	2811.86	-0.47	0.16	1.30E-03	1.58E-02
LRSAM1	1902.20	-0.47	0.20	7.01E-03	5.04E-02
ZNF324	1505.16	-0.48	0.22	1.19E-02	7.13E-02
ZNFX1	7525.04	-0.48	0.13	8.60E-05	2.23E-03
CLASP1	3757.20	-0.48	0.23	1.38E-02	7.89E-02
RANBP10	1937.32	-0.48	0.14	2.78E-04	5.38E-03
PA2G4	7321.07	-0.48	0.21	8.85E-03	5.89E-02
POLG	3867.24	-0.48	0.20	6.13E-03	4.60E-02
TGFBRAP1	2626.25	-0.48	0.15	7.14E-04	1.04E-02
NCOA6	3030.93	-0.48	0.08	5.71E-09	1.23E-06
HINT3	1875.24	-0.48	0.17	1.54E-03	1.77E-02
GORASP1	3938.76	-0.48	0.07	1.29E-11	6.40E-09
SIVA1	4963.05	-0.48	0.19	3.50E-03	3.12E-02
GPHN	1257.24	-0.48	0.16	1.10E-03	1.41E-02
BCAS3	2529.88	-0.48	0.20	5.39E-03	4.22E-02
ST3GAL3	1784.15	-0.48	0.21	7.47E-03	5.27E-02
TCTA	3537.71	-0.48	0.25	1.83E-02	9.58E-02

PCGF5	4675.52	-0.48	0.21	7.71E-03	5.39E-02
MECP2	2582.73	-0.49	0.22	9.47E-03	6.15E-02
SF1	9709.96	-0.49	0.20	6.21E-03	4.64E-02
POLD2	4912.80	-0.49	0.14	1.85E-04	4.02E-03
NOC4L	1271.17	-0.49	0.14	2.02E-04	4.29E-03
TBC1D22B	755.14	-0.49	0.17	1.11E-03	1.42E-02
STAT6	12664.38	-0.49	0.24	1.27E-02	7.42E-02
CASKIN2	1125.01	-0.50	0.21	6.85E-03	4.97E-02
ZNF131	2526.74	-0.50	0.20	4.43E-03	3.70E-02
HNRNPM	12896.11	-0.50	0.13	7.43E-05	2.05E-03
STARD7	11927.90	-0.50	0.23	1.20E-02	7.14E-02
RPAP1	1671.17	-0.50	0.16	7.19E-04	1.04E-02
RELA	6015.79	-0.50	0.16	7.33E-04	1.05E-02
LRRC57	1411.40	-0.50	0.23	1.04E-02	6.53E-02
CALCOCO2	10958.28	-0.50	0.14	1.22E-04	2.88E-03
PSIP1	7216.09	-0.50	0.15	2.36E-04	4.76E-03
ABHD14B	4273.98	-0.50	0.15	3.33E-04	6.10E-03
THTPA	1324.19	-0.50	0.21	6.55E-03	4.82E-02
SMG7	3999.68	-0.50	0.23	1.06E-02	6.57E-02
EIF1AX	6560.81	-0.50	0.22	8.26E-03	5.64E-02
SOGA1	2013.52	-0.50	0.16	7.80E-04	1.10E-02
NUP214	3388.95	-0.50	0.15	2.56E-04	5.08E-03
CCDC61	858.71	-0.50	0.15	2.09E-04	4.38E-03
ADD1	24907.05	-0.50	0.26	1.72E-02	9.20E-02
MAFG	3194.34	-0.50	0.12	1.96E-05	7.56E-04
BTG3	4172.80	-0.50	0.23	9.12E-03	6.00E-02
UBE2H	13834.34	-0.51	0.19	3.08E-03	2.86E-02
UBAP1	5799.37	-0.51	0.20	3.95E-03	3.41E-02
TCP1	11270.55	-0.51	0.20	4.04E-03	3.47E-02
MLYCD	1484.26	-0.51	0.19	2.94E-03	2.77E-02
INPP5J	174.78	-0.51	1.00	1.81E-02	9.48E-02
INPP5E	1776.34	-0.51	0.14	1.32E-04	3.04E-03
SDE2	2086.24	-0.51	0.21	5.14E-03	4.09E-02
NRF1	1069.92	-0.51	0.13	3.19E-05	1.11E-03
SRSF4	8274.28	-0.51	0.15	2.51E-04	5.01E-03
MFSD14A	6208.39	-0.51	0.18	1.67E-03	1.88E-02
RAB21	5889.46	-0.52	0.21	5.41E-03	4.24E-02
ZNF410	4756.46	-0.52	0.16	5.81E-04	8.94E-03
SLC22A4	425.53	-0.52	0.26	1.54E-02	8.50E-02
ZXDB	1446.82	-0.52	0.25	1.24E-02	7.32E-02
WDFY3	2663.97	-0.52	0.16	4.01E-04	6.96E-03
GABPB2	991.26	-0.52	0.19	2.15E-03	2.24E-02
PUF60	10311.68	-0.52	0.13	3.34E-05	1.12E-03
ILKAP	2286.93	-0.52	0.14	7.72E-05	2.10E-03
HOMER1	541.51	-0.52	0.21	4.73E-03	3.87E-02
RXRA	3863.77	-0.52	0.25	1.28E-02	7.45E-02
SCAF4	3252.69	-0.52	0.20	3.35E-03	3.03E-02
FLVCR1	352.41	-0.52	0.23	8.18E-03	5.61E-02
PPP2CB	2318.53	-0.52	0.28	1.85E-02	9.64E-02
GRB10	3059.28	-0.52	0.21	4.84E-03	3.93E-02
TTC7B	2129.61	-0.52	0.28	1.86E-02	9.68E-02
CNPPD1	4865.80	-0.52	0.14	8.43E-05	2.22E-03
ROCK2	12055.01	-0.52	0.19	1.70E-03	1.90E-02
UBIAD1	1874.39	-0.52	0.18	1.11E-03	1.42E-02
CNBP	34476.24	-0.52	0.16	2.72E-04	5.32E-03
CSAD	942.36	-0.52	0.23	8.29E-03	5.64E-02
CES2	5288.01	-0.52	0.19	1.93E-03	2.08E-02
GNA12	9260.26	-0.52	0.22	6.22E-03	4.65E-02
HIF1AN	3211.96	-0.53	0.20	2.73E-03	2.65E-02
LDLRAD3	1912.65	-0.53	0.28	1.88E-02	9.73E-02
AHCTF1	3622.36	-0.53	0.22	5.29E-03	4.18E-02
TSSC4	3165.82	-0.53	0.16	3.95E-04	6.88E-03
USP42	1838.36	-0.53	0.22	6.18E-03	4.63E-02
SLC25A12	2320.55	-0.53	0.10	1.08E-07	1.38E-05
USP18	948.70	-0.53	0.20	2.98E-03	2.79E-02
CXXC1	3889.96	-0.53	0.16	3.44E-04	6.28E-03
BBS7	1992.05	-0.53	0.19	1.92E-03	2.07E-02
CYB5R3	50265.27	-0.53	0.25	1.01E-02	6.41E-02
TFEB	2126.82	-0.53	0.18	1.23E-03	1.52E-02
RHOQ	6422.14	-0.53	0.25	1.09E-02	6.73E-02
VPS4A	4105.08	-0.53	0.10	8.44E-08	1.11E-05
TAPT1	2490.90	-0.54	0.18	9.51E-04	1.27E-02
INTS1	5725.56	-0.54	0.27	1.49E-02	8.32E-02
SUPT6H	4357.88	-0.54	0.15	1.50E-04	3.40E-03
LYSMD4	752.88	-0.54	0.28	1.72E-02	9.17E-02
BCL2L1	2824.43	-0.54	0.22	5.30E-03	4.18E-02
FAM53C	5799.22	-0.54	0.18	8.67E-04	1.18E-02
TRMT2B	924.82	-0.54	0.24	8.23E-03	5.63E-02
NOLC1	6607.44	-0.54	0.25	9.45E-03	6.14E-02
STX16	5323.56	-0.54	0.24	8.32E-03	5.65E-02
DHX30	5825.12	-0.54	0.11	3.19E-07	3.43E-05
PITPNB	9872.05	-0.54	0.16	3.04E-04	5.72E-03
WDR45B	7648.04	-0.55	0.13	1.10E-05	4.91E-04
PAF1	4677.67	-0.55	0.19	1.14E-03	1.43E-02
UBE2B	4522.04	-0.55	0.16	1.69E-04	3.72E-03
DIXDC1	4925.38	-0.55	0.30	1.87E-02	9.68E-02
NATD1	1461.14	-0.55	0.28	1.52E-02	8.41E-02
C18orf25	2018.58	-0.55	0.20	2.42E-03	2.45E-02
TOLLIP	5441.61	-0.55	0.17	3.86E-04	6.77E-03
ING1	2352.99	-0.55	0.30	1.94E-02	9.92E-02
USP20	2302.82	-0.55	0.25	9.04E-03	5.97E-02
RPTOR	868.11	-0.55	0.23	5.08E-03	4.06E-02
IBA57	687.15	-0.55	0.17	2.94E-04	5.60E-03
KAT6A	3149.50	-0.55	0.21	2.70E-03	2.63E-02
SOD1	11246.08	-0.55	0.15	7.59E-05	2.09E-03
DHX38	2791.58	-0.55	0.14	1.98E-05	7.58E-04
SLC27A5	317.03	-0.56	0.25	8.30E-03	5.64E-02
PLP2	5849.52	-0.56	0.25	8.30E-03	5.64E-02
ATL2	2750.49	-0.56	0.21	2.86E-03	2.71E-02
SLC38A2	40804.00	-0.56	0.22	3.70E-03	3.25E-02
PHC2	10808.91	-0.56	0.30	1.58E-02	8.66E-02
PCF11	2894.26	-0.56	0.21	2.07E-03	2.19E-02

ZNF653	270.08	-0.56	0.30	1.63E-02	8.84E-02
LRRC47	4095.43	-0.56	0.19	8.58E-04	1.18E-02
SLC41A3	2617.80	-0.56	0.20	1.96E-03	2.10E-02
OSBPL1A	8399.93	-0.56	0.23	4.87E-03	3.95E-02
DDX51	1830.07	-0.56	0.25	7.55E-03	5.32E-02
LZTS2	9755.50	-0.56	0.28	1.12E-02	6.85E-02
BCR	2662.70	-0.56	0.23	3.99E-03	3.44E-02
KLC1	11872.27	-0.56	0.13	4.02E-06	2.34E-04
JOSD1	1291.92	-0.56	0.22	3.54E-03	3.15E-02
PHRF1	3119.55	-0.56	0.19	1.10E-03	1.41E-02
NUDC	8739.43	-0.56	0.19	7.35E-04	1.05E-02
LRRC75B	2189.12	-0.56	0.31	1.80E-02	9.48E-02
NFKBIB	888.76	-0.57	0.23	4.48E-03	3.72E-02
YY1AP1	4750.93	-0.57	0.17	3.51E-04	6.37E-03
PIK3C2A	3354.87	-0.57	0.20	1.63E-03	1.85E-02
BAZ2A	4763.72	-0.57	0.20	1.32E-03	1.59E-02
SSBP2	6409.44	-0.57	0.31	1.77E-02	9.35E-02
NFAT5	3765.22	-0.57	0.20	1.19E-03	1.48E-02
COQ8B	1763.43	-0.57	0.24	5.01E-03	4.01E-02
PRKAA2	253.93	-0.57	1.21	1.85E-02	9.64E-02
APLP2	40263.77	-0.57	0.26	7.97E-03	5.51E-02
AKAP8L	4986.54	-0.57	0.26	8.28E-03	5.64E-02
SNRNP200	8747.76	-0.57	0.16	1.07E-04	2.65E-03
PTPRK	4208.56	-0.58	0.27	9.18E-03	6.03E-02
NGRN	11789.97	-0.58	0.21	1.75E-03	1.95E-02
ELMSAN1	4761.43	-0.58	0.29	1.21E-02	7.18E-02
TICAM1	1460.42	-0.58	0.30	1.42E-02	8.04E-02
RBMXL1	1995.66	-0.58	0.23	3.65E-03	3.22E-02
COX17	1888.54	-0.58	0.23	3.43E-03	3.07E-02
ARL2	6479.00	-0.58	0.14	1.07E-05	4.83E-04
NOD1	1454.73	-0.58	0.22	2.61E-03	2.58E-02
FBXO42	3814.28	-0.58	0.23	3.34E-03	3.02E-02
FAM219B	4299.33	-0.58	0.28	1.02E-02	6.44E-02
VAV2	1959.81	-0.58	0.15	4.16E-05	1.33E-03
PANK4	2042.70	-0.58	0.14	8.21E-06	3.98E-04
MAP3K3	5988.61	-0.58	0.14	1.41E-05	5.84E-04
DHPS	2570.43	-0.58	0.22	2.23E-03	2.31E-02
BTN2A1	2510.64	-0.58	0.27	7.47E-03	5.27E-02
TSR2	3107.80	-0.58	0.21	1.58E-03	1.81E-02
SP2	1185.21	-0.59	0.23	2.87E-03	2.72E-02
TMEM129	5460.09	-0.59	0.23	3.24E-03	2.95E-02
VSIR	16020.97	-0.59	0.32	1.54E-02	8.50E-02
AEN	1719.19	-0.59	0.29	1.03E-02	6.48E-02
SLC25A44	2358.08	-0.59	0.16	5.01E-05	1.52E-03
POLRMT	2861.87	-0.59	0.13	1.93E-06	1.33E-04
BARD1	705.02	-0.59	0.26	6.53E-03	4.81E-02
SMC1A	5630.24	-0.59	0.20	1.11E-03	1.42E-02
KAT7	4336.23	-0.59	0.27	6.96E-03	5.03E-02
DHX16	2835.04	-0.59	0.19	4.61E-04	7.68E-03
CDK2	1895.15	-0.59	0.32	1.54E-02	8.52E-02
TSNARE1	644.54	-0.60	0.24	3.78E-03	3.31E-02
RIMKLB	3092.26	-0.60	0.33	1.71E-02	9.14E-02
ADCY9	2818.54	-0.60	0.27	7.69E-03	5.39E-02
CEP68	4642.29	-0.60	0.24	3.99E-03	3.44E-02
CCNDBP1	6070.96	-0.60	0.19	3.74E-04	6.63E-03
MYLIP	5601.26	-0.60	0.26	6.04E-03	4.55E-02
ATG4D	971.64	-0.60	0.28	8.72E-03	5.83E-02
FAM199X	1771.08	-0.60	0.26	5.59E-03	4.32E-02
CLK3	3911.93	-0.60	0.19	4.24E-04	7.22E-03
TUSC1	4640.19	-0.60	0.30	1.00E-02	6.38E-02
TMEM120A	3114.73	-0.60	0.18	3.09E-04	5.80E-03
CALCOCO1	11594.05	-0.60	0.34	1.79E-02	9.42E-02
OCRL	1689.03	-0.60	0.29	9.03E-03	5.97E-02
PPM1B	5586.11	-0.60	0.20	5.99E-04	9.08E-03
ANAPC16	7942.11	-0.60	0.29	1.02E-02	6.43E-02
SLC29A2	116.60	-0.60	1.30	1.89E-02	9.75E-02
CLCN6	1844.98	-0.60	0.28	7.52E-03	5.31E-02
FDFT1	5706.58	-0.60	0.27	7.06E-03	5.07E-02
EHD1	7447.44	-0.61	0.26	5.57E-03	4.31E-02
NCOA1	6792.27	-0.61	0.22	1.39E-03	1.65E-02
BCL2L13	4267.86	-0.61	0.31	1.21E-02	7.20E-02
INPP4A	2375.40	-0.61	0.24	2.73E-03	2.64E-02
TBL3	3368.02	-0.61	0.17	1.15E-04	2.75E-03
DNAJA1	17942.06	-0.61	0.34	1.63E-02	8.82E-02
SMAD7	2953.40	-0.61	0.30	9.94E-03	6.36E-02
RERE	3815.35	-0.61	0.26	4.87E-03	3.95E-02
SYPL1	12828.72	-0.61	0.18	1.88E-04	4.07E-03
YPEL2	4182.46	-0.61	0.29	8.64E-03	5.80E-02
SAFB	7708.00	-0.61	0.18	1.58E-04	3.53E-03
VPS13D	6245.45	-0.61	0.26	5.02E-03	4.02E-02
NRBP2	4148.70	-0.61	0.26	4.48E-03	3.72E-02
H3F3B	95184.70	-0.61	0.15	2.06E-05	7.78E-04
PLAGL2	1020.98	-0.61	0.29	8.98E-03	5.95E-02
NFE2L2	19157.30	-0.61	0.24	2.99E-03	2.80E-02
ZYX	27661.24	-0.61	0.17	7.13E-05	2.00E-03
INPP5K	3201.49	-0.61	0.22	1.23E-03	1.52E-02
UNC45A	7205.88	-0.62	0.20	7.27E-04	1.05E-02
TRMT6	848.97	-0.62	0.22	1.59E-03	1.82E-02
SELENOK	6244.30	-0.62	0.33	1.40E-02	7.98E-02
TAF3	2163.18	-0.62	0.28	6.62E-03	4.84E-02
PEX19	5589.73	-0.62	0.26	4.28E-03	3.59E-02
GAS8	1042.89	-0.62	0.34	1.47E-02	8.25E-02
HSP90AB1	42868.21	-0.62	0.30	9.45E-03	6.14E-02
KLHL42	2923.58	-0.62	0.22	1.01E-03	1.32E-02
ATG101	1679.66	-0.63	0.28	5.75E-03	4.40E-02
KIAA0232	2716.16	-0.63	0.24	2.25E-03	2.32E-02
LIN37	547.77	-0.63	0.24	2.12E-03	2.22E-02
EIF4H	23468.40	-0.63	0.12	1.20E-07	1.49E-05
ENGASE	2245.61	-0.63	0.32	1.05E-02	6.56E-02
TECR	6733.95	-0.63	0.22	1.29E-03	1.57E-02
IDI1	4291.78	-0.63	0.34	1.33E-02	7.64E-02
GDI1	9401.54	-0.63	0.21	5.95E-04	9.06E-03

NACC2	2239.10	-0.63	0.27	4.88E-03	3.95E-02
ZNF263	3221.34	-0.63	0.25	3.12E-03	2.88E-02
FAM47E	96.63	-0.63	0.32	1.03E-02	6.50E-02
FAHD2A	1348.04	-0.63	0.23	1.45E-03	1.70E-02
SHMT1	941.85	-0.64	0.33	1.10E-02	6.76E-02
TMEM135	2572.72	-0.64	0.35	1.51E-02	8.37E-02
ZFYVE21	8221.13	-0.64	0.30	7.75E-03	5.41E-02
KLF8	1338.39	-0.64	0.37	1.74E-02	9.28E-02
ZNF891	565.28	-0.64	0.30	7.68E-03	5.38E-02
SLCO3A1	5598.56	-0.64	0.28	5.25E-03	4.16E-02
WBP11	6266.19	-0.64	0.16	2.15E-05	8.04E-04
RALY	12931.47	-0.64	0.21	5.42E-04	8.50E-03
KIF13A	2542.30	-0.64	0.37	1.64E-02	8.88E-02
TNFSF10	3339.13	-0.64	0.30	7.87E-03	5.47E-02
IFFO2	261.27	-0.64	0.36	1.48E-02	8.29E-02
KLF13	2571.93	-0.64	0.30	7.55E-03	5.32E-02
PINK1	9556.06	-0.64	0.23	1.17E-03	1.47E-02
RANBP2	4722.33	-0.64	0.21	4.93E-04	7.99E-03
GMEB2	1463.68	-0.64	0.33	1.14E-02	6.92E-02
ANXA11	22371.25	-0.64	0.15	3.87E-06	2.29E-04
PRPF8	14120.97	-0.65	0.19	1.64E-04	3.65E-03
TMEM106C	4526.09	-0.65	0.31	7.78E-03	5.42E-02
BRPF3	1919.79	-0.65	0.32	9.29E-03	6.07E-02
URGCP	2932.43	-0.65	0.22	8.53E-04	1.17E-02
DLC1	13455.28	-0.65	0.33	1.05E-02	6.56E-02
ANKRD46	1870.35	-0.65	0.28	4.15E-03	3.52E-02
DYRK3	1508.57	-0.65	0.23	1.06E-03	1.37E-02
NUDT7	631.87	-0.65	0.32	9.36E-03	6.10E-02
TUBB4B	13640.37	-0.66	0.36	1.29E-02	7.50E-02
RAVER2	940.52	-0.66	0.39	1.74E-02	9.25E-02
DNAJC18	1689.11	-0.66	0.33	9.05E-03	5.97E-02
PPT2	1697.75	-0.66	0.30	6.04E-03	4.55E-02
GPATCH4	1208.63	-0.66	0.21	4.68E-04	7.73E-03
GAMT	1896.46	-0.66	0.32	7.92E-03	5.49E-02
DAB2IP	833.45	-0.66	0.33	9.70E-03	6.25E-02
SGF29	1132.06	-0.66	0.33	8.57E-03	5.77E-02
SUDS3	3383.11	-0.66	0.23	8.36E-04	1.15E-02
MANSC1	1468.32	-0.66	0.25	1.87E-03	2.04E-02
CTTNBP2	704.36	-0.66	0.36	1.29E-02	7.50E-02
TRIM47	7749.91	-0.66	0.24	1.39E-03	1.65E-02
PIR	599.47	-0.67	0.32	7.37E-03	5.22E-02
CLDND1	9613.81	-0.67	0.18	3.71E-05	1.21E-03
CAMTA1	3432.57	-0.67	0.33	9.49E-03	6.15E-02
CABIN1	1425.93	-0.67	0.28	4.12E-03	3.51E-02
ENSA	6611.23	-0.67	0.25	2.15E-03	2.24E-02
PNMA1	8769.98	-0.67	0.33	8.72E-03	5.83E-02
CEP131	1382.48	-0.67	0.38	1.46E-02	8.20E-02
SRRM2	38102.79	-0.67	0.25	1.24E-03	1.53E-02
TAF7	10832.26	-0.67	0.19	1.07E-04	2.64E-03
HIPK1	2999.93	-0.67	0.26	2.40E-03	2.43E-02
TMBIM1	20155.60	-0.67	0.26	1.87E-03	2.04E-02
LCLAT1	2494.65	-0.67	0.42	1.89E-02	9.76E-02
MTMR12	1833.57	-0.68	0.15	2.15E-06	1.43E-04
ZBTB10	3185.17	-0.68	0.31	5.78E-03	4.41E-02
DDX27	3759.48	-0.68	0.21	2.74E-04	5.33E-03
HYI	1596.72	-0.68	0.30	4.53E-03	3.76E-02
GNB5	3150.32	-0.68	0.30	4.25E-03	3.58E-02
WBP4	1832.06	-0.68	0.20	1.17E-04	2.80E-03
EXTL3	2541.46	-0.69	0.21	2.11E-04	4.40E-03
GSTM2	3006.91	-0.69	0.32	6.62E-03	4.84E-02
FKBP4	4850.31	-0.69	0.38	1.27E-02	7.42E-02
RNF38	3280.53	-0.69	0.27	2.43E-03	2.46E-02
SMARCD3	4491.56	-0.69	0.37	1.13E-02	6.89E-02
NOC2L	4668.05	-0.69	0.19	7.23E-05	2.02E-03
DDX5	85900.34	-0.69	0.18	3.21E-05	1.11E-03
MAPKBP1	1154.45	-0.69	0.33	7.02E-03	5.05E-02
LRP10	20321.49	-0.70	0.33	6.83E-03	4.96E-02
EHD2	65644.28	-0.70	0.26	1.84E-03	2.01E-02
ACBD4	1394.97	-0.70	0.31	4.81E-03	3.92E-02
SRF	1461.86	-0.70	0.18	2.24E-05	8.26E-04
ECH1	13995.63	-0.70	0.27	2.11E-03	2.21E-02
MED14OS	136.21	-0.70	0.31	4.89E-03	3.96E-02
FKRP	1307.66	-0.70	0.27	1.78E-03	1.97E-02
EEF2K	3799.20	-0.70	0.27	2.04E-03	2.16E-02
GTPBP1	3164.66	-0.70	0.16	3.50E-06	2.11E-04
MAF1	11333.07	-0.70	0.18	2.51E-05	9.14E-04
SYNE1	9324.02	-0.70	0.30	3.67E-03	3.23E-02
BCL7B	3840.82	-0.70	0.22	3.16E-04	5.89E-03
LRRC14	2708.51	-0.70	0.16	4.20E-06	2.41E-04
KLC2	1906.27	-0.70	0.34	7.33E-03	5.20E-02
NHEJ1	1033.20	-0.71	0.23	3.83E-04	6.74E-03
C2CD2	2494.74	-0.71	0.26	1.16E-03	1.45E-02
SIRT1	3417.18	-0.71	0.28	2.06E-03	2.19E-02
GPRC5B	5482.81	-0.71	0.46	1.82E-02	9.53E-02
MAGI2	1790.85	-0.71	0.47	1.93E-02	9.90E-02
MAP1B	32707.54	-0.71	0.39	1.10E-02	6.76E-02
LRCH1	1824.45	-0.71	0.30	3.23E-03	2.95E-02
KYAT1	400.53	-0.71	0.26	1.40E-03	1.66E-02
PLCB1	2428.70	-0.71	0.24	5.80E-04	8.94E-03
TLCD2	434.74	-0.71	0.32	4.61E-03	3.80E-02
SLC25A23	4264.97	-0.71	0.45	1.65E-02	8.90E-02
DZANK1	191.04	-0.72	0.30	3.23E-03	2.95E-02
KLHL36	2623.45	-0.72	0.27	1.65E-03	1.86E-02
HNRNPH3	8465.15	-0.72	0.15	3.65E-07	3.68E-05
ALDH16A1	1827.73	-0.72	0.33	5.18E-03	4.12E-02
SMIM10	867.62	-0.72	0.43	1.41E-02	7.98E-02
PVR	1829.30	-0.72	0.49	1.86E-02	9.66E-02
MTRF1L	1494.78	-0.73	0.23	2.94E-04	5.60E-03
LYRM1	1977.99	-0.73	0.18	1.12E-05	4.96E-04
ILK	23913.66	-0.73	0.25	6.74E-04	9.96E-03
STK38L	4133.87	-0.73	0.25	7.50E-04	1.07E-02
RAF1	7856.79	-0.73	0.13	6.24E-09	1.32E-06

FUK	813.78	-0.73	0.31	3.40E-03	3.06E-02
FAM207A	532.01	-0.73	0.37	8.48E-03	5.72E-02
HABP4	3473.91	-0.73	0.34	5.60E-03	4.32E-02
FCHSD2	4653.73	-0.73	0.45	1.50E-02	8.36E-02
IGSF8	4095.77	-0.73	0.31	3.17E-03	2.91E-02
PPP1CB	20500.89	-0.73	0.22	1.96E-04	4.20E-03
UPF1	6006.68	-0.73	0.19	3.24E-05	1.11E-03
PRRT2	1743.99	-0.73	0.45	1.55E-02	8.56E-02
EHBP1	3891.21	-0.73	0.42	1.22E-02	7.23E-02
SAFB2	3846.86	-0.74	0.27	1.30E-03	1.58E-02
FAM131A	977.26	-0.74	0.32	4.00E-03	3.44E-02
BRD2	11831.26	-0.74	0.35	5.53E-03	4.29E-02
TMEM245	5846.24	-0.74	0.29	2.00E-03	2.14E-02
DCP1A	4272.93	-0.74	0.19	2.53E-05	9.19E-04
SCPEP1	12712.07	-0.74	0.39	9.53E-03	6.17E-02
KCTD10	8480.65	-0.74	0.21	7.31E-05	2.03E-03
RNF122	288.64	-0.74	0.40	9.51E-03	6.16E-02
PKIG	3557.60	-0.74	0.42	1.13E-02	6.87E-02
BCL7A	614.13	-0.75	0.47	1.53E-02	8.47E-02
DGKE	402.04	-0.75	0.40	9.39E-03	6.11E-02
LMTK2	1020.30	-0.75	0.33	4.02E-03	3.45E-02
STAT5B	6294.99	-0.75	0.23	1.96E-04	4.21E-03
CFL2	7548.36	-0.75	0.20	3.28E-05	1.11E-03
HIPK3	9764.72	-0.75	0.26	8.59E-04	1.18E-02
MFN2	4943.81	-0.75	0.21	5.94E-05	1.73E-03
RBM14	5461.34	-0.75	0.09	1.25E-17	2.74E-14
IGHMBP2	1509.99	-0.76	0.33	3.44E-03	3.08E-02
KLHDC4	652.45	-0.76	0.19	1.99E-05	7.60E-04
HERC3	2517.30	-0.76	0.33	3.41E-03	3.06E-02
CCDC9	1255.44	-0.76	0.28	1.30E-03	1.58E-02
MRPS6	4795.47	-0.76	0.37	6.22E-03	4.65E-02
CSNK1E	7677.01	-0.77	0.23	1.37E-04	3.15E-03
DUSP1	140575.22	-0.77	0.53	1.50E-02	8.34E-02
HDX	772.67	-0.77	0.27	6.94E-04	1.02E-02
PPARG	1361.10	-0.77	0.52	1.72E-02	9.16E-02
SORBS3	21676.48	-0.77	0.37	6.05E-03	4.56E-02
CDKN2D	820.99	-0.77	0.42	9.55E-03	6.18E-02
SLC44A2	12326.35	-0.77	0.23	1.92E-04	4.13E-03
IRF2BP2	8514.67	-0.77	0.28	9.04E-04	1.22E-02
SELENOW	18532.02	-0.78	0.36	4.76E-03	3.89E-02
ANKDD1A	811.51	-0.78	0.26	5.75E-04	8.91E-03
ST6GALNAC6	13688.15	-0.78	0.33	3.24E-03	2.95E-02
TCF7L1	2813.58	-0.78	0.41	8.66E-03	5.81E-02
SLC20A2	2495.85	-0.78	0.26	4.78E-04	7.84E-03
AKIRIN1	4838.91	-0.78	0.19	9.65E-06	4.49E-04
RMND5B	2882.43	-0.78	0.24	2.52E-04	5.01E-03
DHODH	644.64	-0.78	0.35	4.04E-03	3.47E-02
RAD23A	9132.57	-0.78	0.19	4.64E-06	2.57E-04
SLC5A3	2128.82	-0.78	0.57	1.88E-02	9.73E-02
SERGEF	1150.58	-0.78	0.27	6.08E-04	9.16E-03
TLE3	3172.43	-0.78	0.49	1.38E-02	7.90E-02
TMEM173	6723.23	-0.79	0.20	1.13E-05	4.97E-04
BEND3	253.86	-0.79	0.39	6.67E-03	4.87E-02
CAMK2N1	2594.38	-0.79	0.42	8.87E-03	5.90E-02
MACROD1	1362.55	-0.79	0.22	8.30E-05	2.19E-03
FYCO1	3764.72	-0.80	0.46	1.13E-02	6.89E-02
KIAA1958	328.26	-0.80	0.47	1.15E-02	6.96E-02
WDR74	1694.85	-0.80	0.31	1.52E-03	1.76E-02
TBC1D8	1169.83	-0.80	0.39	5.58E-03	4.31E-02
MAST4	3582.41	-0.80	0.27	5.00E-04	8.07E-03
GBA2	3900.63	-0.80	0.26	3.54E-04	6.38E-03
ANG	1714.81	-0.80	0.48	1.22E-02	7.23E-02
ZFP36L2	82189.00	-0.80	0.38	5.20E-03	4.13E-02
HOXA6	404.93	-0.80	0.36	3.75E-03	3.29E-02
PHF1	3806.79	-0.80	0.37	4.68E-03	3.84E-02
FAM107A	1017.78	-0.80	2.37	1.65E-02	8.90E-02
MAMLD1	687.55	-0.81	0.46	1.07E-02	6.61E-02
UBC	200791.70	-0.81	0.17	9.03E-07	7.37E-05
SPIDR	4964.44	-0.81	0.22	4.77E-05	1.47E-03
RUBCN	2547.46	-0.81	0.30	1.21E-03	1.50E-02
TNRC6C	1971.82	-0.81	0.23	6.90E-05	1.96E-03
HADH	3980.77	-0.81	0.29	7.89E-04	1.10E-02
EPM2A	718.07	-0.82	0.43	7.70E-03	5.39E-02
CLTCL1	630.19	-0.82	0.36	3.73E-03	3.27E-02
ACTA1	20.70	-0.82	0.60	1.64E-02	8.85E-02
POLE	1075.59	-0.82	0.28	6.37E-04	9.51E-03
ROCK1	11938.97	-0.82	0.36	3.31E-03	3.00E-02
KCNJ14	62.15	-0.82	0.45	8.71E-03	5.83E-02
SRSF3	24725.16	-0.82	0.28	5.42E-04	8.50E-03
TMEM47	12942.99	-0.82	0.41	6.59E-03	4.83E-02
JUP	4157.05	-0.82	0.58	1.72E-02	9.16E-02
DNAJB5	2205.64	-0.82	0.29	7.54E-04	1.07E-02
CYSTM1	6139.56	-0.82	0.36	3.40E-03	3.06E-02
C9orf3	4162.48	-0.82	0.45	9.15E-03	6.01E-02
PPP2R1B	2902.57	-0.83	0.30	8.73E-04	1.19E-02
TYSND1	1245.94	-0.83	0.27	4.03E-04	6.96E-03
NDE1	3445.13	-0.83	0.26	2.54E-04	5.04E-03
RBM12	4499.01	-0.83	0.22	2.67E-05	9.60E-04
TNFRSF10D	1908.41	-0.83	0.52	1.27E-02	7.40E-02
DNAJB4	9785.26	-0.84	0.26	2.65E-04	5.20E-03
CASC3	8110.16	-0.84	0.23	4.56E-05	1.42E-03
SLC4A5	110.42	-0.84	0.43	6.35E-03	4.71E-02
STARD9	1625.18	-0.84	0.24	1.04E-04	2.58E-03
MTUS1	6070.42	-0.84	0.48	9.95E-03	6.36E-02
ASB13	2685.46	-0.84	0.24	7.65E-05	2.09E-03
TPCN1	8622.99	-0.84	0.31	1.14E-03	1.43E-02
SREBF2	4396.00	-0.84	0.24	9.50E-05	2.41E-03
BTBD3	2035.26	-0.84	0.34	2.03E-03	2.16E-02
PEAR1	4119.55	-0.85	0.69	1.89E-02	9.78E-02
KLF7	6072.63	-0.85	0.24	5.83E-05	1.71E-03
CTDSPL	3730.53	-0.85	0.32	1.23E-03	1.52E-02
SPEN	5944.09	-0.85	0.31	9.58E-04	1.27E-02

MYO1C	23671.11	-0.85	0.21	1.21E-05	5.23E-04
PLEKHG6	58.35	-0.85	0.67	1.77E-02	9.36E-02
LDOC1	5836.28	-0.85	0.62	1.66E-02	8.94E-02
FAHD2B	974.39	-0.85	0.51	1.06E-02	6.59E-02
BCL2L2	766.63	-0.85	0.25	9.97E-05	2.51E-03
VCL	29718.83	-0.86	0.25	1.27E-04	2.95E-03
DEPTOR	3624.18	-0.86	0.36	2.28E-03	2.34E-02
DUSP3	7001.45	-0.86	0.30	7.32E-04	1.05E-02
CCDC71L	2331.19	-0.86	0.63	1.58E-02	8.66E-02
SLC45A4	356.17	-0.86	0.33	1.35E-03	1.62E-02
BCOR	1412.00	-0.86	0.30	6.04E-04	9.12E-03
ZNF10	964.02	-0.86	0.37	2.69E-03	2.62E-02
TRIP10	7984.98	-0.87	0.42	5.00E-03	4.01E-02
NDRG2	11807.79	-0.87	0.62	1.55E-02	8.54E-02
KCNIP3	1593.50	-0.87	0.61	1.47E-02	8.24E-02
CRTC3	6196.81	-0.87	0.34	1.40E-03	1.65E-02
PANX1	2812.41	-0.88	0.37	2.54E-03	2.54E-02
MGP	172523.94	-0.88	0.49	7.26E-03	5.18E-02
IFRD1	7124.14	-0.88	0.34	1.52E-03	1.76E-02
PHF10	2997.27	-0.88	0.31	5.99E-04	9.08E-03
PPP2R5A	3035.19	-0.88	0.29	3.54E-04	6.38E-03
NDRG1	12156.76	-0.88	0.38	2.55E-03	2.54E-02
PACSIN2	5734.11	-0.88	0.21	4.45E-06	2.51E-04
ARHGEF17	7489.30	-0.88	0.41	3.82E-03	3.33E-02
PIP5KL1	132.18	-0.88	0.51	8.77E-03	5.85E-02
RELL1	1010.13	-0.88	0.36	1.80E-03	1.98E-02
FAM13A	4214.47	-0.89	0.31	7.02E-04	1.02E-02
TLN2	2204.15	-0.89	0.31	5.79E-04	8.94E-03
MINDY2	4163.52	-0.89	0.20	2.44E-06	1.59E-04
TNFSF12	4542.01	-0.89	0.26	9.55E-05	2.42E-03
MFHAS1	967.47	-0.89	0.22	8.55E-06	4.09E-04
MCL1	35066.62	-0.89	0.26	9.02E-05	2.31E-03
ATP6V0A1	4484.18	-0.89	0.38	2.34E-03	2.39E-02
ARPC1A	12823.56	-0.90	0.35	1.37E-03	1.64E-02
FST	1954.03	-0.90	0.88	1.96E-02	9.99E-02
UNC13B	1797.67	-0.90	0.23	1.11E-05	4.93E-04
PPRC1	3574.12	-0.90	0.42	3.94E-03	3.41E-02
CDADC1	1565.85	-0.90	0.28	2.16E-04	4.45E-03
C22orf39	2680.77	-0.90	0.29	2.86E-04	5.46E-03
NAP1L5	1518.64	-0.90	0.52	8.69E-03	5.82E-02
PDK2	5513.35	-0.90	0.45	5.13E-03	4.09E-02
DDX3X	33593.67	-0.90	0.31	6.01E-04	9.08E-03
PTCHD1	101.92	-0.90	0.86	1.86E-02	9.66E-02
TESK2	586.91	-0.90	0.54	9.85E-03	6.32E-02
GPER1	1581.81	-0.90	0.39	2.56E-03	2.55E-02
NR1H2	8700.87	-0.91	0.20	6.70E-07	5.87E-05
SERP2	400.08	-0.91	0.43	4.13E-03	3.51E-02
TNS2	16419.49	-0.91	0.34	9.49E-04	1.27E-02
TXLNG	1666.80	-0.91	0.44	4.68E-03	3.84E-02
ENDOD1	4593.08	-0.91	0.33	7.80E-04	1.10E-02
OVGP1	71.40	-0.91	0.40	2.65E-03	2.61E-02
C8orf86	29.35	-0.92	0.82	1.71E-02	9.15E-02
MTHFR	3191.46	-0.92	0.29	2.02E-04	4.29E-03
ISYNA1	8360.97	-0.92	0.80	1.82E-02	9.53E-02
GARNL3	1606.69	-0.92	0.41	3.16E-03	2.90E-02
COX7A1	2607.10	-0.92	0.36	1.45E-03	1.70E-02
KLHL21	6416.15	-0.92	0.44	4.39E-03	3.67E-02
SIX2	1700.65	-0.92	0.55	9.20E-03	6.04E-02
FAM122A	4158.39	-0.92	0.30	3.25E-04	6.00E-03
ALS2CL	1490.04	-0.92	0.67	1.39E-02	7.92E-02
MAML3	677.11	-0.93	0.73	1.54E-02	8.51E-02
ZSCAN18	6514.22	-0.93	0.33	7.00E-04	1.02E-02
TLN1	63434.59	-0.93	0.26	5.74E-05	1.69E-03
SH3RF3	1996.59	-0.93	0.43	3.82E-03	3.33E-02
ARHGAP17	7689.35	-0.93	0.22	5.98E-06	3.08E-04
LINGO1	2986.91	-0.93	0.71	1.47E-02	8.23E-02
MYH7B	128.03	-0.93	0.53	7.80E-03	5.43E-02
CAMK1	3381.75	-0.94	0.26	4.99E-05	1.52E-03
CCDC86	1948.06	-0.94	0.24	1.41E-05	5.84E-04
CYTH3	6826.01	-0.94	0.32	5.59E-04	8.72E-03
RPS6KA5	1367.03	-0.94	0.24	1.58E-05	6.37E-04
SPSB1	7466.79	-0.94	0.69	1.38E-02	7.90E-02
VAMP2	5217.83	-0.94	0.41	2.77E-03	2.67E-02
ELL	2551.36	-0.94	0.32	4.82E-04	7.88E-03
SLC41A1	2490.06	-0.94	0.29	1.67E-04	3.70E-03
FOXO3	17788.49	-0.94	0.42	2.83E-03	2.70E-02
EDA	371.27	-0.94	0.60	1.05E-02	6.56E-02
PPP1R12A	15885.26	-0.94	0.36	1.26E-03	1.54E-02
SYNE3	1216.25	-0.94	0.35	9.90E-04	1.31E-02
MAP7D3	3714.23	-0.94	0.42	2.76E-03	2.66E-02
TTLL7	2364.52	-0.94	0.59	9.97E-03	6.36E-02
TPM1	34066.27	-0.94	0.37	1.59E-03	1.82E-02
ACACB	1659.66	-0.95	0.38	1.70E-03	1.90E-02
ACTG2	9848.81	-0.95	1.19	1.93E-02	9.92E-02
MOB3B	2130.28	-0.95	0.77	1.57E-02	8.63E-02
PTK2	12348.98	-0.95	0.34	7.52E-04	1.07E-02
GEM	12385.49	-0.95	0.39	1.77E-03	1.96E-02
OLFM1	1945.01	-0.95	0.64	1.15E-02	6.94E-02
MAPRE2	17894.73	-0.96	0.53	7.43E-03	5.25E-02
H2AFJ	4046.24	-0.96	0.25	2.02E-05	7.72E-04
MAP3K6	4252.16	-0.96	0.29	1.24E-04	2.90E-03
COL4A6	1134.84	-0.96	2.80	1.53E-02	8.49E-02
MPRIP	11825.63	-0.96	0.38	1.47E-03	1.71E-02
LGI3	59.28	-0.96	0.77	1.45E-02	8.15E-02
SLC7A2	2598.63	-0.96	0.99	1.81E-02	9.48E-02
ARHGEF6	3692.22	-0.96	0.34	6.66E-04	9.89E-03
LRRC24	397.55	-0.96	0.49	5.26E-03	4.17E-02
DAAM2	4326.86	-0.96	0.48	5.00E-03	4.01E-02
FEM1C	3630.19	-0.97	0.44	3.11E-03	2.87E-02
MANEAL	160.25	-0.97	0.86	1.61E-02	8.77E-02
WDR1	20087.84	-0.97	0.29	9.03E-05	2.31E-03
IDS	14060.90	-0.97	0.29	1.14E-04	2.74E-03

ZNRD1	2241.69	-0.97	0.50	5.52E-03	4.29E-02
ICAM1	17526.07	-0.97	0.96	1.73E-02	9.23E-02
NRARP	2228.35	-0.97	1.05	1.77E-02	9.35E-02
C1orf198	15141.89	-0.97	0.47	4.20E-03	3.55E-02
FILIP1	4175.68	-0.98	0.21	4.76E-07	4.45E-05
SLC8B1	5943.68	-0.98	0.32	3.19E-04	5.92E-03
RUSC2	6703.79	-0.98	0.26	1.80E-05	7.04E-04
PPP1R10	7841.31	-0.98	0.27	4.68E-05	1.45E-03
SLC48A1	2600.54	-0.98	0.26	2.70E-05	9.65E-04
CAMK2G	893.49	-0.98	0.40	1.73E-03	1.93E-02
ACTN4	5316.25	-0.98	0.30	1.66E-04	3.68E-03
JMJD6	2424.77	-0.99	0.42	2.28E-03	2.34E-02
DGKD	1864.44	-0.99	0.33	3.23E-04	5.98E-03
SMURF1	2209.96	-0.99	0.19	2.49E-08	4.11E-06
CDH13	5076.97	-0.99	0.44	2.59E-03	2.57E-02
ERF	983.86	-0.99	0.41	1.67E-03	1.88E-02
C12orf75	9906.53	-0.99	0.60	8.20E-03	5.62E-02
LGALS4	41.00	-1.00	0.68	1.10E-02	6.75E-02
FSTL3	6909.22	-1.00	0.41	1.74E-03	1.93E-02
SLC17A7	1076.00	-1.00	0.89	1.56E-02	8.58E-02
EPN2	4421.88	-1.00	0.34	4.45E-04	7.46E-03
SUN1	16532.16	-1.00	0.29	7.65E-05	2.09E-03
PER2	2434.70	-1.00	0.36	6.19E-04	9.28E-03
RRAS	11905.56	-1.00	0.27	3.63E-05	1.19E-03
MBOAT1	319.76	-1.00	0.34	3.57E-04	6.42E-03
SMTN	18594.57	-1.00	0.44	2.49E-03	2.50E-02
EPAS1	18893.34	-1.00	0.70	1.20E-02	7.14E-02
GABARAPL1	6435.89	-1.00	0.22	4.68E-07	4.40E-05
SIPA1L1	2278.84	-1.00	0.48	3.92E-03	3.40E-02
CCDC181	21.65	-1.01	2.28	1.48E-02	8.26E-02
DCAF4L1	11.27	-1.01	1.64	1.61E-02	8.77E-02
RAB6B	539.27	-1.01	0.42	1.96E-03	2.11E-02
CDS2	4473.30	-1.01	0.49	4.20E-03	3.55E-02
TSC22D2	9742.65	-1.01	0.34	4.01E-04	6.96E-03
LONRF1	4941.34	-1.01	0.25	9.46E-06	4.44E-04
LPP	17846.74	-1.01	0.29	6.98E-05	1.97E-03
SMYD4	2215.28	-1.02	0.39	1.05E-03	1.36E-02
TMEM109	17785.63	-1.02	0.20	3.90E-08	5.93E-06
PID1	4330.45	-1.02	0.55	5.92E-03	4.49E-02
CTC1	1071.22	-1.02	0.30	8.20E-05	2.19E-03
HAPLN3	2167.63	-1.02	0.57	6.51E-03	4.80E-02
CNNM2	1747.82	-1.03	0.46	2.71E-03	2.63E-02
EIF1B	5637.54	-1.03	0.23	9.13E-07	7.41E-05
LRP5	3679.87	-1.03	0.51	4.41E-03	3.69E-02
SBDS	13855.92	-1.03	0.24	1.87E-06	1.29E-04
TGFBR2	36003.97	-1.03	0.44	2.17E-03	2.25E-02
SLC7A9	13.64	-1.04	0.61	6.89E-03	4.99E-02
OSGIN1	634.55	-1.04	0.39	8.99E-04	1.22E-02
ZNF274	1491.71	-1.04	0.38	7.39E-04	1.06E-02
RNF150	1430.13	-1.04	0.88	1.40E-02	7.97E-02
ATP13A4	16.19	-1.04	1.66	1.55E-02	8.55E-02
SMIM1	37.94	-1.04	0.72	1.04E-02	6.54E-02
FOXO1	10337.54	-1.05	0.42	1.53E-03	1.77E-02
ATP6V0E2	1184.79	-1.05	0.71	1.02E-02	6.44E-02
FAAH	330.51	-1.05	0.47	2.54E-03	2.54E-02
PDE3A	4530.57	-1.05	0.52	4.08E-03	3.49E-02
PYGB	10097.29	-1.05	0.37	5.25E-04	8.33E-03
PLK3	3809.81	-1.06	0.18	7.69E-10	2.00E-07
LSMEM1	66.62	-1.06	0.70	9.48E-03	6.15E-02
TEAD3	2533.90	-1.06	0.31	7.71E-05	2.10E-03
COQ10B	6600.26	-1.06	0.32	1.14E-04	2.73E-03
TESK1	2270.35	-1.06	0.23	3.25E-07	3.44E-05
HSPB2	666.13	-1.06	0.29	2.77E-05	9.83E-04
UTRN	15224.52	-1.07	0.44	1.76E-03	1.95E-02
HSPA1A	152624.93	-1.07	0.76	1.05E-02	6.56E-02
FBXO32	7861.81	-1.07	0.61	6.62E-03	4.84E-02
MKNK2	5811.95	-1.07	0.32	1.15E-04	2.76E-03
ABR	5933.60	-1.07	0.36	3.23E-04	5.98E-03
SOCS2	2692.82	-1.07	0.59	5.89E-03	4.48E-02
SLC6A9	2245.00	-1.07	0.63	7.30E-03	5.19E-02
BCL3	5525.69	-1.07	0.37	4.75E-04	7.81E-03
CCBE1	837.58	-1.07	1.39	1.58E-02	8.66E-02
ADCY6	4396.37	-1.08	0.37	4.06E-04	7.01E-03
ACSS1	2878.41	-1.08	0.58	5.49E-03	4.27E-02
STK24	8534.25	-1.08	0.36	3.73E-04	6.63E-03
ECHDC2	3848.98	-1.08	0.52	3.49E-03	3.11E-02
IRF2BPL	3361.66	-1.08	0.34	2.05E-04	4.34E-03
ARHGEF7	6397.62	-1.08	0.45	1.85E-03	2.02E-02
EZH1	3776.89	-1.08	0.41	9.07E-04	1.22E-02
SAMD4A	1451.16	-1.08	0.47	2.27E-03	2.34E-02
TACC2	3336.06	-1.08	0.36	3.07E-04	5.76E-03
WWP2	4980.80	-1.08	0.40	7.96E-04	1.11E-02
SRPK3	176.69	-1.09	1.86	1.50E-02	8.34E-02
RCL1	1514.76	-1.09	0.28	1.25E-05	5.38E-04
GZF1	1720.45	-1.09	0.40	7.69E-04	1.08E-02
ESRRG	51.99	-1.09	1.24	1.50E-02	8.36E-02
FLOT2	5259.51	-1.09	0.26	4.23E-06	2.42E-04
ZNF460	912.68	-1.09	0.23	4.22E-07	4.15E-05
MTURN	2704.41	-1.10	0.53	3.68E-03	3.25E-02
HSPB8	8253.74	-1.10	0.57	4.78E-03	3.90E-02
SPATA2L	953.41	-1.10	0.27	5.73E-06	2.99E-04
ALDH4A1	1343.92	-1.10	0.48	2.21E-03	2.29E-02
RGS9	505.62	-1.10	0.40	5.96E-04	9.06E-03
C8orf88	864.54	-1.10	0.40	6.01E-04	9.08E-03
CFAP221	67.76	-1.11	0.77	9.93E-03	6.36E-02
STK40	5675.59	-1.11	0.18	3.71E-11	1.62E-08
SLC27A3	5879.51	-1.11	0.28	9.63E-06	4.49E-04
SV2C	26.37	-1.11	2.09	1.43E-02	8.09E-02
PPP1R15B	7841.00	-1.11	0.31	4.41E-05	1.38E-03
NFKBIA	54985.90	-1.11	0.76	9.42E-03	6.13E-02
SLC25A33	948.03	-1.11	0.33	7.99E-05	2.15E-03
EMP2	7755.74	-1.12	0.20	1.19E-09	2.95E-07

EHBP1L1	3317.99	-1.12	0.40	5.10E-04	8.13E-03
RAI2	3109.08	-1.12	0.83	1.07E-02	6.63E-02
PHF13	2246.76	-1.13	0.47	1.65E-03	1.86E-02
LRRC8A	10134.12	-1.13	0.16	4.65E-14	5.09E-11
SOX15	272.48	-1.13	1.53	1.46E-02	8.20E-02
HOXA13	139.21	-1.13	2.06	1.42E-02	8.04E-02
STOM	31841.22	-1.14	0.27	3.65E-06	2.18E-04
VPS37B	2951.46	-1.14	0.44	1.06E-03	1.37E-02
PELI2	1828.29	-1.14	0.49	1.91E-03	2.07E-02
ARHGEF37	511.33	-1.14	0.40	5.11E-04	8.13E-03
PLXNB1	1922.14	-1.14	0.46	1.29E-03	1.57E-02
PRODH	56.01	-1.14	0.75	8.29E-03	5.64E-02
HS6ST1	4610.57	-1.15	0.56	3.56E-03	3.17E-02
NECAB1	1439.61	-1.15	0.31	2.55E-05	9.20E-04
PCDH1	4791.80	-1.15	1.26	1.49E-02	8.31E-02
UBA2	8865.65	-1.15	0.33	6.72E-05	1.92E-03
MTMR7	76.95	-1.15	0.46	1.19E-03	1.48E-02
PRDM11	728.59	-1.15	0.23	1.14E-07	1.45E-05
CPT1A	1896.73	-1.15	0.33	5.64E-05	1.67E-03
PNPLA2	5260.66	-1.16	0.25	3.83E-07	3.84E-05
PRKAG2	2277.72	-1.16	0.56	3.26E-03	2.96E-02
RERG	9645.10	-1.16	0.47	1.23E-03	1.52E-02
ZNF385C	60.80	-1.16	0.60	4.11E-03	3.50E-02
EXOC3L4	214.76	-1.16	0.44	7.62E-04	1.08E-02
ARID5B	18965.01	-1.16	0.41	4.94E-04	8.01E-03
B3GALNT2	1607.27	-1.17	0.27	1.72E-06	1.23E-04
FAM129A	13067.92	-1.17	0.52	2.08E-03	2.19E-02
MAN2A2	2672.15	-1.17	0.28	4.50E-06	2.52E-04
PLCE1	2327.53	-1.17	0.53	2.49E-03	2.50E-02
AMOTL2	12000.49	-1.17	0.43	6.21E-04	9.30E-03
EIF1	69322.48	-1.17	0.20	2.56E-10	7.71E-08
TNFAIP2	19769.01	-1.17	0.54	2.65E-03	2.61E-02
FANCE	345.32	-1.17	0.35	8.29E-05	2.19E-03
TMEM35A	463.30	-1.17	0.97	1.17E-02	7.02E-02
STXBP1	5150.53	-1.18	0.26	5.23E-07	4.72E-05
MAP3K7CL	2482.28	-1.18	0.87	1.01E-02	6.41E-02
RDH5	730.07	-1.18	0.36	1.05E-04	2.60E-03
PANK1	608.16	-1.18	0.69	6.21E-03	4.64E-02
LGALSL	3838.25	-1.18	0.35	6.59E-05	1.89E-03
HSFX1	66.08	-1.18	0.62	4.38E-03	3.67E-02
ADH1B	18131.37	-1.18	0.96	1.14E-02	6.91E-02
ZEB1	7667.85	-1.19	0.32	1.82E-05	7.06E-04
ABCA3	1178.26	-1.19	0.80	8.65E-03	5.81E-02
ACYP1	591.11	-1.19	0.33	3.33E-05	1.12E-03
GADL1	51.76	-1.19	0.38	1.68E-04	3.72E-03
TSC22D3	64679.80	-1.19	0.89	1.06E-02	6.59E-02
ADGRB3	174.75	-1.19	1.24	1.32E-02	7.61E-02
OR51E1	1389.21	-1.19	1.34	1.36E-02	7.81E-02
RAMP1	1600.73	-1.19	0.89	1.02E-02	6.44E-02
GCNT4	95.43	-1.19	0.94	1.06E-02	6.58E-02
CRIP1	29142.65	-1.20	0.39	2.28E-04	4.61E-03
ADIPOQ	39.65	-1.20	1.14	1.21E-02	7.18E-02
KIF1C	4591.13	-1.20	0.31	1.00E-05	4.57E-04
TPRG1	510.50	-1.20	0.36	8.56E-05	2.22E-03
FOSL2	25173.46	-1.20	0.56	2.71E-03	2.63E-02
JADE1	4714.54	-1.21	0.46	8.46E-04	1.16E-02
HDAC5	7025.70	-1.21	0.40	2.14E-04	4.43E-03
SLC38A1	4117.14	-1.21	0.90	9.96E-03	6.36E-02
ABCD2	288.76	-1.21	0.72	6.16E-03	4.62E-02
TNNI3K	97.19	-1.22	0.57	2.72E-03	2.64E-02
JUN	75011.85	-1.22	0.39	1.52E-04	3.43E-03
ADARB1	2090.09	-1.22	0.56	2.58E-03	2.56E-02
AFF3	1165.70	-1.22	1.02	1.12E-02	6.86E-02
FBLN5	33494.72	-1.22	0.93	1.04E-02	6.54E-02
C1orf167	268.32	-1.22	0.58	2.89E-03	2.73E-02
PROCR	12325.64	-1.23	0.75	6.78E-03	4.93E-02
LIMD1	1898.50	-1.23	0.40	2.09E-04	4.38E-03
HSPB1	54642.79	-1.23	0.23	1.39E-08	2.50E-06
PTCH1	522.45	-1.23	0.54	1.89E-03	2.05E-02
RIC3	184.22	-1.23	0.80	7.66E-03	5.37E-02
GLB1L2	89.31	-1.24	0.72	5.66E-03	4.35E-02
FRZB	8534.10	-1.24	0.96	1.01E-02	6.40E-02
HES1	4111.74	-1.25	0.59	2.71E-03	2.63E-02
C1QTNF1	13845.49	-1.25	0.69	4.92E-03	3.97E-02
OSBP2	211.80	-1.25	0.66	4.27E-03	3.59E-02
KLHL15	2410.42	-1.25	0.28	1.24E-06	9.66E-05
CCNI2	60.66	-1.25	0.93	9.29E-03	6.07E-02
CMTM5	67.52	-1.25	1.18	1.17E-02	7.05E-02
MYEF2	181.86	-1.25	1.10	1.13E-02	6.89E-02
TBC1D1	4772.53	-1.26	0.43	3.14E-04	5.87E-03
GPR88	93.19	-1.26	0.39	1.29E-04	2.99E-03
PPARGC1A	333.10	-1.26	0.61	3.07E-03	2.85E-02
SOD3	20062.03	-1.26	0.84	7.86E-03	5.46E-02
SLMAP	7103.93	-1.26	0.31	4.46E-06	2.51E-04
RGN	779.81	-1.26	0.74	5.91E-03	4.49E-02
DBNDD2	7040.72	-1.27	0.60	2.73E-03	2.64E-02
ECE1	8761.78	-1.27	0.39	1.13E-04	2.73E-03
WDR17	172.87	-1.27	0.50	9.50E-04	1.27E-02
GNAL	1264.29	-1.27	0.66	3.99E-03	3.44E-02
MARK1	2206.03	-1.27	0.41	1.88E-04	4.07E-03
CAB39L	3670.69	-1.27	0.64	3.49E-03	3.11E-02
FKBP5	18591.08	-1.27	1.03	1.04E-02	6.54E-02
NXT1	2482.93	-1.28	0.35	2.86E-05	1.01E-03
GRHL1	420.00	-1.28	0.90	8.41E-03	5.69E-02
HRH2	1787.43	-1.28	2.25	1.29E-02	7.50E-02
MAP2	2172.41	-1.29	1.10	1.10E-02	6.75E-02
CTH	640.99	-1.29	0.60	2.52E-03	2.52E-02
CHD1	5115.74	-1.29	0.40	1.13E-04	2.73E-03
GRK5	1911.89	-1.29	0.24	1.28E-08	2.37E-06
PXDC1	8090.84	-1.29	0.45	3.74E-04	6.63E-03
SYNE2	8365.78	-1.29	0.53	1.26E-03	1.54E-02
ABTB1	5464.94	-1.29	0.56	1.67E-03	1.88E-02

INMT	3129.75	-1.29	0.63	3.09E-03	2.86E-02
TEX29	10.76	-1.30	1.97	1.21E-02	7.20E-02
PDE1B	2446.26	-1.30	0.66	3.77E-03	3.30E-02
RGMA	1748.61	-1.30	0.73	5.10E-03	4.06E-02
FOLH1	418.97	-1.30	0.64	3.23E-03	2.95E-02
CNNM3	2679.90	-1.30	0.40	1.12E-04	2.72E-03
TP53I11	13323.02	-1.30	0.29	1.07E-06	8.50E-05
CELA2A	8.52	-1.30	1.13	9.95E-03	6.36E-02
NAF1	695.21	-1.30	0.41	1.43E-04	3.26E-03
SYN2	32.32	-1.30	0.79	6.08E-03	4.57E-02
TSC22D1	51547.95	-1.31	0.52	9.49E-04	1.27E-02
MEF2D	7386.18	-1.31	0.30	1.40E-06	1.05E-04
L3MBTL4	585.53	-1.31	0.74	4.98E-03	4.00E-02
COLCA2	454.92	-1.31	0.77	5.57E-03	4.31E-02
TMEM25	1538.73	-1.32	0.47	4.72E-04	7.77E-03
TBX2	8374.16	-1.32	0.72	4.70E-03	3.85E-02
NPR1	5891.75	-1.32	0.92	8.36E-03	5.66E-02
GPRC5A	9147.55	-1.32	0.83	6.69E-03	4.88E-02
PALM2	232.72	-1.33	0.81	6.25E-03	4.66E-02
ZNF141	1192.75	-1.33	0.50	7.24E-04	1.05E-02
EGR3	996.30	-1.33	0.67	3.37E-03	3.04E-02
LIMS2	6173.59	-1.33	0.48	5.28E-04	8.36E-03
SYNPO2	7927.81	-1.33	0.60	1.97E-03	2.11E-02
NNMT	29475.13	-1.34	0.45	3.19E-04	5.92E-03
TMC7	248.00	-1.34	1.16	1.05E-02	6.56E-02
CASP9	1501.82	-1.34	0.34	7.93E-06	3.89E-04
CAT	13847.76	-1.34	0.21	1.25E-11	6.40E-09
CECR2	30.13	-1.35	1.35	1.10E-02	6.74E-02
INAFM2	1428.10	-1.35	0.71	3.94E-03	3.41E-02
RBKS	1062.77	-1.36	0.98	8.35E-03	5.66E-02
PPP1R12C	3886.28	-1.36	0.33	3.71E-06	2.21E-04
FLNA	126394.79	-1.36	0.30	3.47E-07	3.56E-05
RYR2	691.81	-1.36	0.55	1.12E-03	1.42E-02
TLE2	2073.87	-1.37	0.47	2.99E-04	5.65E-03
PDE5A	6067.86	-1.38	0.63	2.24E-03	2.31E-02
MAP3K8	4977.76	-1.38	0.86	6.38E-03	4.72E-02
RND3	13010.46	-1.38	0.43	1.17E-04	2.80E-03
RASL12	7997.22	-1.39	0.53	7.56E-04	1.07E-02
ATOH8	1787.75	-1.39	0.54	8.27E-04	1.14E-02
CRYAB	14762.11	-1.39	0.49	3.60E-04	6.46E-03
SOX5	1446.14	-1.39	0.47	2.58E-04	5.11E-03
GP1BB	1276.58	-1.39	0.42	7.79E-05	2.11E-03
MOB3C	2896.61	-1.40	0.28	9.08E-08	1.18E-05
GADD45G	3704.91	-1.40	0.68	2.77E-03	2.66E-02
DSTN	67797.49	-1.40	0.40	4.38E-05	1.38E-03
ARHGEF10L	7544.51	-1.41	0.46	2.10E-04	4.39E-03
ANGPTL7	1352.09	-1.41	1.41	1.10E-02	6.76E-02
RDH10	3707.21	-1.41	0.92	7.01E-03	5.04E-02
DCUN1D3	3356.98	-1.41	0.19	1.19E-14	1.53E-11
PIP5K1B	467.78	-1.41	1.47	1.12E-02	6.84E-02
TMEM51	2193.47	-1.41	0.85	5.61E-03	4.32E-02
SLC4A11	192.05	-1.42	0.64	2.04E-03	2.17E-02
EIF4A3	15312.17	-1.43	0.55	8.13E-04	1.13E-02
BMP2	4277.89	-1.43	0.68	2.61E-03	2.58E-02
SLC25A18	469.70	-1.43	0.76	3.97E-03	3.42E-02
CCDC107	4213.59	-1.43	0.33	1.65E-06	1.20E-04
ITGA7	2891.64	-1.43	0.82	4.95E-03	3.98E-02
PIM1	5777.02	-1.43	0.71	3.03E-03	2.83E-02
THRA	4582.33	-1.44	0.53	5.64E-04	8.77E-03
SORL1	2252.97	-1.44	1.86	1.16E-02	6.98E-02
GPC4	4144.91	-1.44	0.59	1.12E-03	1.42E-02
KLF11	6269.07	-1.44	0.39	1.91E-05	7.38E-04
CPED1	7181.07	-1.45	0.44	1.09E-04	2.67E-03
OLAH	48.45	-1.45	1.04	7.73E-03	5.41E-02
ARVCF	2018.24	-1.45	0.63	1.66E-03	1.87E-02
GPD1L	2697.07	-1.45	0.37	9.78E-06	4.53E-04
CRIP2	25336.87	-1.45	0.54	5.77E-04	8.94E-03
NR3C2	551.01	-1.45	0.52	4.38E-04	7.36E-03
LTBP4	71836.00	-1.45	0.93	6.35E-03	4.71E-02
KLHL38	96.84	-1.46	1.46	1.06E-02	6.57E-02
BCL6	4956.38	-1.46	0.44	8.83E-05	2.28E-03
ADCY3	11434.48	-1.46	0.25	3.38E-10	9.78E-08
SLC16A8	130.60	-1.46	0.57	8.42E-04	1.16E-02
BHLHE40	15948.64	-1.46	0.50	3.27E-04	6.02E-03
S1PR3	7404.57	-1.46	0.30	1.74E-07	2.09E-05
HSPB7	3414.04	-1.46	0.71	2.66E-03	2.61E-02
GTF2B	4144.86	-1.47	0.34	2.09E-06	1.41E-04
RADIL	1223.69	-1.47	0.64	1.65E-03	1.86E-02
FADS3	5443.38	-1.47	0.50	2.62E-04	5.17E-03
RCAN1	10838.51	-1.47	0.63	1.53E-03	1.77E-02
ASPA	1490.05	-1.47	0.41	3.43E-05	1.15E-03
CYCS	17812.37	-1.48	0.82	4.29E-03	3.60E-02
CDHR3	201.27	-1.48	0.35	2.28E-06	1.50E-04
FOSB	30794.83	-1.48	0.57	7.89E-04	1.10E-02
TGFBR3	16366.17	-1.48	0.91	5.70E-03	4.37E-02
COL11A2	112.06	-1.49	0.81	4.08E-03	3.49E-02
LGI4	2782.60	-1.49	0.80	3.84E-03	3.34E-02
CSRP2	6864.32	-1.49	0.71	2.50E-03	2.51E-02
ANGPT1	3990.53	-1.49	0.42	3.88E-05	1.26E-03
TMEM38B	2777.46	-1.49	0.42	3.58E-05	1.19E-03
TRIM72	117.91	-1.49	0.65	1.53E-03	1.77E-02
NFKBIZ	24860.98	-1.50	0.66	1.64E-03	1.85E-02
TMCC2	373.68	-1.50	0.40	1.52E-05	6.17E-04
MYLK	25991.39	-1.50	0.64	1.40E-03	1.65E-02
GRTP1	299.96	-1.50	0.74	2.79E-03	2.67E-02
CBX7	3798.65	-1.50	0.63	1.28E-03	1.57E-02
CARNS1	400.12	-1.50	1.12	7.98E-03	5.51E-02
BVES	508.75	-1.51	0.55	5.02E-04	8.08E-03
MAP3K20	15079.18	-1.51	0.37	4.40E-06	2.50E-04
TACC1	23435.29	-1.51	0.22	3.73E-13	3.46E-10
BMP8B	208.68	-1.51	1.47	1.01E-02	6.39E-02
CD9	27230.32	-1.51	0.48	1.39E-04	3.19E-03

FOS	409588.11	-1.52	0.53	3.63E-04	6.50E-03
SERTAD3	3735.00	-1.52	0.33	3.10E-07	3.41E-05
TP53INP2	3125.79	-1.52	0.42	3.24E-05	1.11E-03
TCAP	19.01	-1.52	1.20	8.27E-03	5.64E-02
ACTA2	220958.22	-1.53	0.75	2.70E-03	2.63E-02
OLFML2A	3309.29	-1.54	0.48	1.25E-04	2.92E-03
B4GALNT1	1137.58	-1.54	0.57	5.97E-04	9.06E-03
ARHGEF39	693.80	-1.54	0.72	2.19E-03	2.27E-02
FGF1	703.49	-1.54	0.55	3.97E-04	6.91E-03
KLHDC9	195.51	-1.54	0.59	6.89E-04	1.01E-02
ABLIM1	12104.34	-1.54	0.85	4.25E-03	3.58E-02
GATM	1247.22	-1.54	1.06	6.99E-03	5.03E-02
SYDE2	863.29	-1.54	0.71	2.01E-03	2.14E-02
DNAJB1	66556.33	-1.54	0.66	1.40E-03	1.65E-02
CNGA3	19.90	-1.54	1.82	1.03E-02	6.48E-02
SPRYD3	9574.20	-1.55	0.31	5.24E-08	7.52E-06
ARL5B	5095.94	-1.55	0.46	6.33E-05	1.83E-03
ZFHX3	4629.71	-1.55	0.34	5.34E-07	4.76E-05
ADGRL3	1033.50	-1.56	1.03	6.43E-03	4.75E-02
CCNL1	18483.33	-1.56	0.45	4.24E-05	1.35E-03
KLHL23	2368.78	-1.56	0.80	3.23E-03	2.95E-02
PPFIA2	532.11	-1.56	0.59	6.23E-04	9.32E-03
SLC2A3	14786.98	-1.56	0.69	1.60E-03	1.82E-02
CSF1	21122.51	-1.57	0.50	1.40E-04	3.20E-03
CCNH	6071.03	-1.57	0.57	4.88E-04	7.94E-03
LAMA5	9787.06	-1.57	0.73	2.09E-03	2.20E-02
MTHFD2	4733.55	-1.58	0.47	7.78E-05	2.11E-03
EGFLAM	3037.51	-1.58	0.52	2.21E-04	4.52E-03
NOTCH3	62927.07	-1.58	0.60	6.77E-04	1.00E-02
NT5C1A	45.26	-1.58	2.45	1.04E-02	6.53E-02
PDK4	102881.26	-1.58	1.35	1.04E-02	6.52E-02
SVEP1	13163.67	-1.59	1.02	6.15E-03	4.61E-02
ATP8B4	888.81	-1.59	0.73	2.03E-03	2.16E-02
HEY2	2566.85	-1.59	0.76	2.35E-03	2.39E-02
SNN	2295.26	-1.59	0.39	3.88E-06	2.29E-04
PM20D2	2686.68	-1.60	0.69	1.39E-03	1.65E-02
TMC4	585.77	-1.60	0.66	1.12E-03	1.42E-02
TOB2	14322.06	-1.60	0.36	8.35E-07	6.98E-05
TPM2	58065.49	-1.60	0.53	2.24E-04	4.57E-03
KCNA2	71.17	-1.60	2.82	1.05E-02	6.56E-02
ITIH4	568.35	-1.60	0.94	4.73E-03	3.87E-02
SLC39A14	14888.45	-1.60	0.52	1.70E-04	3.75E-03
C8orf34	80.98	-1.60	0.42	1.06E-05	4.79E-04
JAG1	16700.91	-1.61	0.36	7.14E-07	6.18E-05
KLF10	23215.25	-1.61	0.41	7.73E-06	3.81E-04
JUND	85501.80	-1.61	0.42	1.11E-05	4.93E-04
ADPRH	1522.47	-1.61	0.35	4.36E-07	4.23E-05
SULT1C4	263.36	-1.62	0.76	2.16E-03	2.25E-02
ICOSLG	311.93	-1.62	0.87	3.60E-03	3.18E-02
TIPARP	6521.91	-1.62	0.45	3.16E-05	1.10E-03
LONRF2	771.92	-1.63	0.39	3.25E-06	1.98E-04
LDLR	2948.57	-1.63	0.47	5.22E-05	1.57E-03
JAKMIP2	52.02	-1.63	0.83	2.94E-03	2.77E-02
SUN2	13744.92	-1.64	0.27	1.48E-10	4.84E-08
PARD6A	304.37	-1.64	0.91	4.03E-03	3.46E-02
KRT222	631.27	-1.64	1.53	9.26E-03	6.06E-02
SPEG	3451.19	-1.64	0.80	2.53E-03	2.53E-02
MIDN	3559.55	-1.64	0.41	5.05E-06	2.75E-04
SNTA1	5291.33	-1.65	0.56	2.53E-04	5.02E-03
LONRF3	349.49	-1.65	0.43	1.15E-05	5.00E-04
LITAF	11616.17	-1.65	0.57	2.96E-04	5.61E-03
SYP	306.67	-1.66	0.70	1.21E-03	1.50E-02
PNRC1	67469.96	-1.66	0.45	2.16E-05	8.04E-04
TNS1	28250.23	-1.66	0.46	2.91E-05	1.02E-03
SLC25A21	72.62	-1.66	0.69	1.14E-03	1.44E-02
THBD	5998.46	-1.66	0.79	2.35E-03	2.39E-02
YBX3	34512.92	-1.67	0.20	2.51E-18	9.61E-15
WFS1	5060.97	-1.67	0.43	9.93E-06	4.55E-04
TRIM7	254.94	-1.67	0.62	4.99E-04	8.06E-03
MT1F	547.39	-1.68	0.47	3.28E-05	1.11E-03
ITPRIP	8547.82	-1.68	0.56	2.15E-04	4.44E-03
USP53	5032.66	-1.68	0.52	1.03E-04	2.58E-03
SPTB	353.68	-1.69	0.86	2.97E-03	2.79E-02
CBFA2T3	1198.22	-1.69	1.00	4.85E-03	3.94E-02
TMTC1	4242.92	-1.69	0.36	3.10E-07	3.41E-05
CNKSR1	45.01	-1.69	0.65	6.38E-04	9.52E-03
CEBPB	25129.08	-1.69	0.44	1.02E-05	4.61E-04
CITED4	1785.20	-1.69	0.58	2.73E-04	5.32E-03
MPPED2	417.24	-1.70	1.19	6.60E-03	4.84E-02
IER2	53583.29	-1.71	0.53	1.08E-04	2.66E-03
ASB16	86.99	-1.71	1.05	5.07E-03	4.06E-02
KLF9	4517.95	-1.71	0.52	7.84E-05	2.12E-03
TMPRSS9	34.57	-1.72	0.95	3.78E-03	3.31E-02
TINAGL1	31834.95	-1.72	1.01	4.61E-03	3.80E-02
CSPG4	10237.86	-1.72	0.53	9.94E-05	2.51E-03
MT1G	439.18	-1.72	1.34	7.65E-03	5.37E-02
SLC25A4	5465.67	-1.72	0.41	2.04E-06	1.39E-04
EBF3	763.17	-1.72	0.85	2.67E-03	2.62E-02
RGS7BP	931.89	-1.72	1.08	5.37E-03	4.22E-02
CORO6	1963.31	-1.72	0.70	9.55E-04	1.27E-02
NPNT	10286.47	-1.72	0.93	3.60E-03	3.18E-02
SPATA25	16.52	-1.73	0.74	1.13E-03	1.43E-02
RBPMS	12962.34	-1.74	0.41	2.15E-06	1.43E-04
INPP5A	7012.49	-1.75	0.49	3.20E-05	1.11E-03
PPM1L	1440.10	-1.75	0.70	8.66E-04	1.18E-02
WFDC1	2675.81	-1.76	1.02	4.45E-03	3.71E-02
FOXL1	485.89	-1.77	0.91	2.98E-03	2.79E-02
SMIM10L2A	105.13	-1.77	0.51	4.07E-05	1.31E-03
LIPH	37.49	-1.77	0.93	3.16E-03	2.90E-02
IL6R	1787.83	-1.78	0.61	2.73E-04	5.32E-03
TMEM110-MUSTN1	1653.51	-1.78	0.53	6.23E-05	1.80E-03
MAOA	2297.12	-1.78	0.61	2.68E-04	5.25E-03

SOX13	2659.43	-1.78	0.39	5.07E-07	4.60E-05
RASGRP2	1515.11	-1.79	0.94	3.14E-03	2.90E-02
FNDC11	28.11	-1.79	1.76	8.77E-03	5.85E-02
NIPAL1	104.33	-1.80	1.14	5.30E-03	4.18E-02
MOB2	3924.94	-1.80	0.45	5.91E-06	3.05E-04
CMKLR1	2372.37	-1.80	0.45	6.44E-06	3.29E-04
MAT2A	24016.81	-1.80	0.27	1.08E-12	8.69E-10
ADAMTSL2	358.67	-1.81	0.72	8.13E-04	1.13E-02
MAFK	649.49	-1.81	0.43	2.62E-06	1.67E-04
KLKB1	25.73	-1.82	0.81	1.49E-03	1.74E-02
ZSWIM5	224.69	-1.82	0.88	2.29E-03	2.35E-02
SERTM1	664.42	-1.82	0.75	1.04E-03	1.36E-02
NMUR1	94.70	-1.82	0.68	5.32E-04	8.41E-03
PPM1E	20.40	-1.83	1.11	4.70E-03	3.85E-02
HACD1	982.07	-1.84	0.74	9.22E-04	1.24E-02
PPP1R12B	14371.79	-1.84	0.72	7.87E-04	1.10E-02
PTH2R	490.21	-1.84	1.49	7.67E-03	5.38E-02
STOX1	139.34	-1.84	1.00	3.45E-03	3.08E-02
ZBTB21	3008.85	-1.85	0.37	7.88E-08	1.04E-05
RHOB	20561.48	-1.85	0.53	3.91E-05	1.27E-03
NUDT4	25519.19	-1.85	0.49	1.39E-05	5.81E-04
TIMP3	192626.55	-1.86	0.40	2.88E-07	3.24E-05
NTN4	9137.09	-1.86	0.72	7.31E-04	1.05E-02
DGKG	1050.63	-1.87	1.01	3.46E-03	3.09E-02
ABCC2	391.87	-1.87	0.95	2.74E-03	2.65E-02
BAG3	15542.97	-1.87	0.33	1.07E-09	2.74E-07
KHK	523.21	-1.87	0.64	2.63E-04	5.17E-03
KLHL26	993.51	-1.87	0.40	2.85E-07	3.24E-05
TMEM56	662.78	-1.87	0.57	8.51E-05	2.22E-03
CAP2	4142.98	-1.88	0.73	6.93E-04	1.02E-02
GLUL	65736.96	-1.88	0.56	6.48E-05	1.86E-03
LEFTY2	1381.96	-1.88	1.36	6.60E-03	4.84E-02
PTGES3L	128.28	-1.88	0.57	7.50E-05	2.06E-03
ELL2	6820.28	-1.89	0.62	1.71E-04	3.76E-03
PDE2A	2073.66	-1.89	1.62	8.16E-03	5.60E-02
MYL9	39536.88	-1.89	0.45	2.53E-06	1.64E-04
PYGM	2182.13	-1.90	0.72	5.79E-04	8.94E-03
ADCYAP1R1	394.73	-1.90	0.87	1.81E-03	1.99E-02
GSN	396161.88	-1.90	0.55	4.34E-05	1.36E-03
CRIM1	4510.05	-1.90	0.48	7.52E-06	3.73E-04
AOC3	28855.17	-1.91	0.54	3.62E-05	1.19E-03
PXDNL	834.36	-1.91	1.58	7.66E-03	5.37E-02
KANK1	6742.90	-1.91	0.24	1.07E-16	1.64E-13
CSRP1	46905.03	-1.91	0.44	1.34E-06	1.02E-04
BEND7	503.00	-1.92	0.40	1.46E-07	1.78E-05
MCAM	37592.16	-1.92	0.94	2.41E-03	2.44E-02
ADAMTS4	10416.63	-1.93	0.67	2.97E-04	5.63E-03
FHL5	5585.53	-1.93	0.98	2.79E-03	2.68E-02
GABRD	385.94	-1.93	1.29	5.69E-03	4.37E-02
SPRY2	9205.87	-1.94	0.43	4.41E-07	4.25E-05
CYP4X1	1103.60	-1.94	0.99	2.81E-03	2.69E-02
PRMT9	2973.07	-1.95	0.57	4.64E-05	1.44E-03
PROSER2	461.33	-1.96	0.32	5.70E-11	2.08E-08
SMOC1	41.82	-1.96	1.93	8.25E-03	5.64E-02
SLC25A25	3601.98	-1.96	0.40	1.07E-07	1.38E-05
GLDN	1193.03	-1.96	1.12	3.98E-03	3.43E-02
MB	70.79	-1.96	0.89	1.64E-03	1.86E-02
WTIP	2358.42	-1.96	0.58	5.76E-05	1.70E-03
ADAMTSL3	3876.07	-1.97	0.71	3.92E-04	6.85E-03
NTRK3	2153.08	-1.97	0.72	4.26E-04	7.23E-03
HEYL	8735.46	-1.97	0.46	1.46E-06	1.08E-04
CTNNA3	294.01	-1.97	1.06	3.25E-03	2.96E-02
ATP1B2	2836.55	-1.97	0.91	1.82E-03	1.99E-02
RPS6KL1	88.00	-1.98	0.74	5.39E-04	8.47E-03
NTRK2	19937.30	-1.98	1.02	2.98E-03	2.79E-02
TIMP4	681.90	-1.98	0.28	2.80E-13	2.86E-10
ADRA1A	289.45	-1.98	1.13	3.87E-03	3.36E-02
NET1	18447.82	-1.98	0.60	8.27E-05	2.19E-03
TRIM36	105.32	-1.99	1.34	5.66E-03	4.35E-02
PROB1	374.58	-1.99	0.47	2.02E-06	1.39E-04
KCNT1	140.83	-1.99	1.31	5.43E-03	4.25E-02
OXCT2	82.93	-2.00	0.63	1.10E-04	2.69E-03
NTRK1	303.02	-2.01	0.86	1.23E-03	1.52E-02
CHST7	1944.84	-2.01	0.92	1.77E-03	1.96E-02
FMO2	5068.50	-2.01	0.84	1.12E-03	1.42E-02
HES6	517.38	-2.02	0.71	3.13E-04	5.85E-03
NAP1L2	102.56	-2.02	1.05	2.88E-03	2.73E-02
ITGA3	2593.62	-2.03	0.75	5.17E-04	8.21E-03
OR51E2	473.70	-2.03	1.28	4.92E-03	3.97E-02
ZC3H12A	3209.63	-2.03	0.59	4.24E-05	1.35E-03
PIGA	1426.44	-2.04	0.58	3.59E-05	1.19E-03
ANGPT4	2369.25	-2.05	1.79	7.75E-03	5.41E-02
SLC2A4	289.85	-2.06	0.84	9.60E-04	1.27E-02
FSTL4	17.97	-2.06	1.12	3.08E-03	2.86E-02
PLD5	74.77	-2.06	2.62	8.63E-03	5.80E-02
ITPRIPL1	447.69	-2.06	0.90	1.35E-03	1.62E-02
ITPK1	4686.87	-2.07	0.50	2.78E-06	1.75E-04
GSTT2	23.75	-2.07	0.85	9.00E-04	1.22E-02
TSPAN19	8.53	-2.08	0.92	1.31E-03	1.58E-02
CFAP74	120.59	-2.10	0.88	1.10E-03	1.41E-02
SEMA3E	441.22	-2.10	0.92	1.36E-03	1.64E-02
APOD	53307.12	-2.10	1.18	3.66E-03	3.23E-02
SCN3A	627.50	-2.11	2.16	8.28E-03	5.64E-02
CAND2	1326.64	-2.11	0.59	2.71E-05	9.68E-04
NFASC	3550.77	-2.13	0.64	6.92E-05	1.96E-03
MT1E	10335.16	-2.14	0.54	6.72E-06	3.39E-04
CHST8	148.64	-2.14	1.64	6.56E-03	4.83E-02
ADIRF	29495.60	-2.14	0.73	2.44E-04	4.89E-03
FAM178B	99.57	-2.14	0.75	3.11E-04	5.83E-03
GNAZ	78.38	-2.16	0.52	2.59E-06	1.66E-04
CDC42EP4	8566.79	-2.16	0.47	3.41E-07	3.56E-05
MGLL	10791.98	-2.18	0.74	2.43E-04	4.88E-03

CABP1	295.51	-2.18	1.04	1.99E-03	2.13E-02
SCUBE3	455.05	-2.19	0.68	1.01E-04	2.53E-03
PTTG2	22.32	-2.19	2.64	8.41E-03	5.69E-02
ANGPTL5	795.92	-2.20	1.58	6.01E-03	4.55E-02
NKD1	710.28	-2.20	0.79	3.85E-04	6.76E-03
TAGLN	94742.85	-2.20	0.63	4.21E-05	1.35E-03
CNN1	15231.53	-2.20	0.59	1.73E-05	6.83E-04
ADCY1	653.09	-2.21	0.81	4.45E-04	7.46E-03
KIAA1671	2188.40	-2.21	0.41	5.04E-09	1.12E-06
SUSD5	4899.90	-2.22	0.80	4.12E-04	7.08E-03
CLMN	5914.40	-2.22	0.74	2.18E-04	4.48E-03
SNTB1	2352.50	-2.22	0.37	1.72E-10	5.51E-08
ACVR1C	55.73	-2.23	0.95	1.11E-03	1.42E-02
NOX4	1479.93	-2.23	0.77	2.80E-04	5.39E-03
BCAM	879.89	-2.24	0.68	7.29E-05	2.03E-03
WDR38	8.90	-2.24	1.09	2.04E-03	2.16E-02
CCDC69	2179.03	-2.24	0.51	9.30E-07	7.47E-05
SYPL2	555.99	-2.25	0.59	1.12E-05	4.96E-04
CYR61	92834.99	-2.26	0.37	6.90E-11	2.46E-08
LIFR	5233.03	-2.26	0.45	4.69E-08	6.86E-06
TRABD2B	465.10	-2.27	0.59	9.96E-06	4.55E-04
ADGRG2	1424.36	-2.28	0.83	4.19E-04	7.17E-03
SLC6A16	136.31	-2.29	0.86	5.45E-04	8.54E-03
NOL3	1990.13	-2.29	0.55	2.91E-06	1.80E-04
CCL8	1348.81	-2.30	1.34	3.95E-03	3.41E-02
SLPI	3288.11	-2.30	1.06	1.79E-03	1.97E-02
PPP1R15A	61690.21	-2.30	0.55	2.38E-06	1.56E-04
ABTB2	732.86	-2.31	0.77	2.11E-04	4.40E-03
LBH	14797.85	-2.31	0.66	3.56E-05	1.19E-03
KBTBD13	67.52	-2.32	1.20	2.73E-03	2.64E-02
DBNDD1	191.75	-2.33	0.95	8.83E-04	1.20E-02
PTP4A3	9623.31	-2.33	0.79	2.43E-04	4.88E-03
ZNF331	10289.15	-2.33	0.70	6.81E-05	1.94E-03
SORT1	5715.36	-2.34	0.74	1.24E-04	2.90E-03
DMPK	7520.88	-2.34	0.64	2.17E-05	8.05E-04
FABP4	6547.66	-2.34	0.90	6.11E-04	9.19E-03
RGL3	648.02	-2.34	0.78	2.09E-04	4.38E-03
SERPINI1	3764.76	-2.35	0.73	9.82E-05	2.49E-03
ACKR2	375.02	-2.35	1.04	1.40E-03	1.66E-02
CKMT2	856.21	-2.35	1.05	1.49E-03	1.74E-02
SGSM1	306.62	-2.35	0.85	4.02E-04	6.96E-03
NRIP2	2896.43	-2.36	0.82	2.95E-04	5.61E-03
ATF3	15031.33	-2.36	0.72	8.04E-05	2.16E-03
AGTR2	118.22	-2.36	2.00	6.74E-03	4.91E-02
HRC	1304.26	-2.36	0.98	1.00E-03	1.32E-02
AKAP1	5965.26	-2.37	0.58	3.92E-06	2.30E-04
MEF2B	684.42	-2.37	0.89	5.33E-04	8.41E-03
UAP1	25327.27	-2.37	0.63	1.37E-05	5.74E-04
ANKK1	6.65	-2.38	1.42	4.10E-03	3.50E-02
CCDC85A	630.19	-2.39	0.56	1.82E-06	1.27E-04
MYOM1	2627.71	-2.39	0.72	6.98E-05	1.97E-03
LDHC	21.19	-2.40	1.40	3.70E-03	3.26E-02
DNAJC6	275.18	-2.40	0.70	5.24E-05	1.58E-03
HUS1B	33.16	-2.42	0.56	1.41E-06	1.05E-04
MPP7	1302.80	-2.43	0.71	5.11E-05	1.55E-03
ADAMTS1	75681.91	-2.43	0.42	6.78E-10	1.79E-07
PER1	9349.85	-2.44	0.96	7.59E-04	1.07E-02
HOXD10	27.41	-2.44	1.89	5.98E-03	4.53E-02
MRGPRE	46.45	-2.45	1.01	8.96E-04	1.22E-02
TTPA	20.20	-2.46	1.15	1.77E-03	1.96E-02
PPP1R14A	4728.26	-2.46	0.81	1.87E-04	4.07E-03
TMEM52	58.20	-2.46	0.96	6.70E-04	9.94E-03
SMCO3	34.58	-2.47	0.46	9.47E-09	1.77E-06
NPY5R	177.89	-2.47	1.97	6.34E-03	4.71E-02
RANBP3L	354.06	-2.48	0.76	9.17E-05	2.34E-03
ARTN	273.97	-2.48	0.75	7.20E-05	2.01E-03
LDB3	1325.68	-2.49	0.93	5.13E-04	8.16E-03
STUM	972.70	-2.49	0.86	2.79E-04	5.39E-03
RXRG	412.54	-2.49	0.91	4.37E-04	7.36E-03
COCH	509.32	-2.50	0.50	5.07E-08	7.34E-06
IGSF9B	146.87	-2.50	0.79	1.22E-04	2.87E-03
SRL	1060.84	-2.51	1.05	1.07E-03	1.38E-02
NTF3	809.30	-2.52	1.19	1.92E-03	2.08E-02
JUNB	210392.23	-2.52	0.49	2.16E-08	3.64E-06
ITPKC	5773.75	-2.54	0.59	1.54E-06	1.13E-04
ADGRG4	43.86	-2.54	0.91	3.66E-04	6.53E-03
ZBTB7C	2023.58	-2.54	0.68	1.49E-05	6.09E-04
DMTN	486.88	-2.55	0.81	1.20E-04	2.85E-03
ZFAND5	81654.16	-2.55	0.71	2.60E-05	9.38E-04
ADRA1D	318.11	-2.55	0.83	1.56E-04	3.52E-03
PHLDA2	3749.19	-2.58	0.55	3.13E-07	3.41E-05
SAA2	22.17	-2.58	1.01	6.94E-04	1.02E-02
KLF2	23929.39	-2.58	0.70	2.08E-05	7.83E-04
ADRA2A	9919.30	-2.59	0.67	9.07E-06	4.27E-04
APOLD1	29743.05	-2.59	1.41	3.30E-03	3.00E-02
PITX1	515.72	-2.59	1.09	1.09E-03	1.40E-02
SNCG	4553.70	-2.59	0.89	2.59E-04	5.12E-03
PERM1	121.51	-2.59	0.99	5.74E-04	8.91E-03
HES4	6570.96	-2.60	0.65	5.42E-06	2.89E-04
NPY1R	2722.04	-2.60	0.93	3.80E-04	6.70E-03
CACNA1H	3681.30	-2.62	0.84	1.35E-04	3.11E-03
HIF3A	265.73	-2.62	0.65	4.46E-06	2.51E-04
WSCD2	1026.28	-2.64	0.78	5.58E-05	1.66E-03
HS3ST2	357.03	-2.65	0.94	3.58E-04	6.43E-03
SFRP5	57.47	-2.66	2.49	6.73E-03	4.90E-02
CASQ2	8318.75	-2.67	1.15	1.27E-03	1.55E-02
CEBPD	68995.24	-2.67	0.60	7.90E-07	6.69E-05
NFIL3	13665.60	-2.70	0.41	5.20E-12	3.19E-09
GPR20	1270.39	-2.70	1.07	7.46E-04	1.07E-02
PLN	14149.90	-2.70	1.00	4.84E-04	7.90E-03
DPF3	168.17	-2.71	0.62	1.26E-06	9.73E-05
GRID2IP	8.05	-2.71	1.96	4.96E-03	3.98E-02

TSPAN8	698.21	-2.71	1.04	6.24E-04	9.33E-03
DNER	409.34	-2.72	1.22	1.51E-03	1.76E-02
MRAP2	340.40	-2.72	1.15	1.07E-03	1.38E-02
SERTAD1	3878.94	-2.72	0.42	6.20E-12	3.66E-09
ATP1A2	1991.00	-2.73	0.98	3.79E-04	6.69E-03
PPP1R9A	310.29	-2.73	0.55	6.44E-08	8.98E-06
SOCS3	60716.32	-2.73	0.70	8.50E-06	4.07E-04
DYNC1I1	1045.56	-2.73	0.65	2.44E-06	1.59E-04
TACR1	67.47	-2.74	1.31	1.90E-03	2.07E-02
JPH2	2145.41	-2.74	0.66	3.35E-06	2.02E-04
PDE4C	644.23	-2.74	0.81	5.87E-05	1.72E-03
RASD1	39517.58	-2.75	1.29	1.89E-03	2.05E-02
BRSK2	194.75	-2.76	0.97	3.30E-04	6.06E-03
ERRFI1	17049.72	-2.77	0.69	5.21E-06	2.79E-04
RIMS3	345.33	-2.78	0.85	9.04E-05	2.31E-03
MYO3A	262.96	-2.79	0.64	1.25E-06	9.68E-05
LMOD1	12222.37	-2.79	0.76	2.20E-05	8.14E-04
IRF1	13852.23	-2.79	0.61	5.32E-07	4.76E-05
CSDC2	3067.68	-2.79	0.84	7.36E-05	2.04E-03
MAATS1	146.33	-2.80	0.63	7.66E-07	6.53E-05
KCNAB1	8391.14	-2.81	0.41	5.20E-13	4.43E-10
MYOZ1	642.58	-2.83	1.04	4.56E-04	7.60E-03
DRD1	636.98	-2.84	2.99	6.98E-03	5.03E-02
CASZ1	336.77	-2.84	0.62	4.56E-07	4.35E-05
EFHD1	14467.81	-2.85	0.53	7.83E-09	1.56E-06
KCNMB1	2571.63	-2.86	0.84	5.73E-05	1.69E-03
FOSL1	1434.34	-2.87	0.81	3.21E-05	1.11E-03
XIRP1	94.52	-2.90	1.50	2.54E-03	2.54E-02
DNAJC27	1923.83	-2.90	0.47	5.18E-11	1.97E-08
ASB2	779.75	-2.91	0.77	1.37E-05	5.74E-04
SLAIN1	327.17	-2.92	0.84	3.95E-05	1.27E-03
ANO3	140.21	-2.98	2.12	5.08E-03	4.06E-02
GREB1L	493.23	-2.98	0.79	1.30E-05	5.52E-04
FOXC2	2146.63	-2.98	0.94	1.18E-04	2.80E-03
EGR1	129789.44	-2.99	0.55	5.52E-09	1.21E-06
SORBS1	5982.13	-2.99	0.60	6.03E-08	8.49E-06
CX3CL1	3095.19	-2.99	0.80	1.77E-05	6.96E-04
MDGA2	31.52	-2.99	1.12	5.01E-04	8.07E-03
NRGN	872.11	-3.00	0.78	9.92E-06	4.55E-04
DACH2	104.45	-3.01	2.45	5.78E-03	4.41E-02
RBPMS2	3003.07	-3.03	0.65	2.96E-07	3.32E-05
HMGCLL1	451.76	-3.04	0.75	4.05E-06	2.34E-04
TC2N	2378.36	-3.04	1.08	3.52E-04	6.37E-03
MT2A	51186.83	-3.05	0.55	2.63E-09	6.11E-07
RERGL	8167.10	-3.05	1.48	2.09E-03	2.20E-02
PRDM16	823.52	-3.06	1.11	4.09E-04	7.04E-03
REEP1	1619.51	-3.07	0.80	1.14E-05	5.00E-04
SYT1	210.41	-3.07	1.04	2.39E-04	4.82E-03
ACTC1	1029.15	-3.09	1.10	3.53E-04	6.38E-03
AJAP1	605.11	-3.10	0.72	1.78E-06	1.26E-04
KCNQ4	511.14	-3.10	0.77	4.71E-06	2.59E-04
CLDN4	346.36	-3.12	1.00	1.47E-04	3.34E-03
C2orf40	2657.32	-3.12	0.88	3.26E-05	1.11E-03
HAS1	9405.39	-3.13	1.05	2.26E-04	4.60E-03
ZFP36	286522.75	-3.14	0.58	8.14E-09	1.58E-06
ADAMTS8	508.83	-3.14	0.77	3.72E-06	2.21E-04
MYC	39997.88	-3.15	0.49	1.46E-11	6.80E-09
NMNAT2	951.73	-3.15	0.83	1.20E-05	5.22E-04
CCDC3	23450.44	-3.16	0.97	9.31E-05	2.37E-03
USP2	833.39	-3.18	0.40	2.30E-16	3.21E-13
KCNK3	1059.80	-3.19	0.89	2.72E-05	9.68E-04
KCNJ3	28.64	-3.24	0.87	1.43E-05	5.89E-04
ADCY5	2379.84	-3.25	0.83	8.07E-06	3.94E-04
TRPV1	1675.05	-3.25	0.54	1.41E-10	4.69E-08
AVPR1A	12789.85	-3.28	0.95	4.91E-05	1.50E-03
VIT	3772.03	-3.29	1.42	1.24E-03	1.53E-02
SYNM	2959.14	-3.31	0.70	1.87E-07	2.22E-05
SGCA	2434.82	-3.32	1.04	1.13E-04	2.73E-03
CPXM2	12394.15	-3.33	0.75	9.27E-07	7.47E-05
CSRNP1	28760.89	-3.33	0.66	4.12E-08	6.09E-06
RRAD	8328.23	-3.34	0.82	4.54E-06	2.52E-04
SLC16A12	39.92	-3.34	0.83	4.81E-06	2.63E-04
DUSP8	2153.98	-3.35	0.56	2.41E-10	7.54E-08
KLHL30	417.94	-3.37	0.78	1.42E-06	1.05E-04
PCOLCE2	18545.93	-3.37	0.86	7.61E-06	3.76E-04
KLF15	226.44	-3.38	0.81	2.98E-06	1.83E-04
CDKN1A	88140.45	-3.39	0.54	4.13E-11	1.71E-08
KBTBD12	243.28	-3.41	0.69	6.99E-08	9.33E-06
GDA	58.74	-3.41	1.02	6.76E-05	1.93E-03
SBSPON	2480.37	-3.44	0.75	4.96E-07	4.56E-05
ART3	197.91	-3.45	0.75	3.62E-07	3.68E-05
SCN4A	188.69	-3.48	0.79	9.64E-07	7.66E-05
ADRB1	192.26	-3.49	1.68	1.91E-03	2.07E-02
ACADL	402.43	-3.50	1.15	1.88E-04	4.07E-03
FRMD1	75.33	-3.52	0.84	2.62E-06	1.67E-04
MYOCD	1516.99	-3.52	1.07	8.49E-05	2.22E-03
EPHA5	195.49	-3.54	1.17	2.00E-04	4.26E-03
CELA2B	6.48	-3.54	1.22	2.27E-04	4.61E-03
MYH11	125221.98	-3.57	1.00	3.25E-05	1.11E-03
FAM189A2	667.46	-3.60	0.81	8.37E-07	6.98E-05
GADD45B	76038.31	-3.60	0.59	1.03E-10	3.51E-08
FOXC1	1679.56	-3.62	0.61	3.87E-10	1.10E-07
GPX3	80743.42	-3.66	0.38	8.06E-23	1.24E-18
RGR	10.13	-3.70	0.99	1.59E-05	6.38E-04
CCL21	1680.38	-3.71	1.23	1.99E-04	4.25E-03
ITIH3	1654.40	-3.71	1.16	1.11E-04	2.70E-03
SORBS2	27041.88	-3.72	0.87	1.82E-06	1.27E-04
RGS6	1419.02	-3.72	0.95	8.27E-06	3.98E-04
ENTPD3	4465.91	-3.72	0.93	5.85E-06	3.03E-04
DEFB1	179.48	-3.74	1.62	1.21E-03	1.50E-02
LGR6	1127.92	-3.77	1.08	4.26E-05	1.35E-03
PIK3C2G	122.02	-3.77	1.77	1.67E-03	1.88E-02

TMEM179	5.27	-3.80	1.94	1.30E-03	1.58E-02
TNNC1	72.53	-3.86	0.99	8.74E-06	4.14E-04
CCL2	39711.81	-3.87	0.74	2.05E-08	3.50E-06
EVX1	28.90	-3.87	1.93	2.16E-03	2.25E-02
BTC	184.75	-3.89	0.81	1.44E-07	1.77E-05
LRRC10B	1543.68	-3.89	0.66	4.86E-10	1.36E-07
RGS16	16091.86	-3.93	0.83	2.43E-07	2.82E-05
SPRR2E	104.17	-3.95	1.47	4.85E-04	7.90E-03
KCNA5	3751.26	-3.98	1.06	1.78E-05	6.98E-04
AQP7	414.25	-3.98	0.85	3.11E-07	3.41E-05
MYH14	112.53	-4.03	1.25	1.02E-04	2.55E-03
ABRA	105.73	-4.04	1.14	3.73E-05	1.21E-03
DKK1	2512.65	-4.05	1.54	5.94E-04	9.06E-03
C11orf53	84.71	-4.08	2.33	2.98E-03	2.79E-02
MT1X	19955.58	-4.11	0.60	1.13E-12	8.69E-10
LSMEM2	415.92	-4.11	0.91	7.00E-07	6.10E-05
MAFF	12734.51	-4.13	0.82	6.71E-08	9.12E-06
PCP4L1	260.80	-4.13	1.55	5.28E-04	8.36E-03
GPM6A	259.39	-4.17	0.81	3.18E-08	5.02E-06
ADRA2C	382.37	-4.17	0.81	2.69E-08	4.34E-06
ZBTB16	10941.97	-4.32	0.99	1.38E-06	1.04E-04
SERPINA3	1835.90	-4.33	0.81	9.39E-09	1.77E-06
BMP5	708.55	-4.33	1.59	4.68E-04	7.73E-03
HBG2	23.12	-4.40	1.74	7.57E-04	1.07E-02
NR4A1	117239.70	-4.44	0.88	6.63E-08	9.12E-06
SLC19A2	6993.39	-4.45	0.51	2.42E-19	1.24E-15
C11orf96	7724.62	-4.46	0.53	9.89E-18	2.53E-14
BTG2	91008.99	-4.53	0.81	2.91E-09	6.66E-07
RXFP2	32.94	-4.54	1.33	4.46E-05	1.39E-03
PRG4	9624.19	-4.60	1.09	3.00E-06	1.84E-04
RBM24	2364.05	-4.61	0.81	1.79E-09	4.37E-07
IL6	12258.25	-4.80	0.92	2.59E-08	4.23E-06
PGPEP1L	18.66	-4.82	1.58	2.13E-04	4.42E-03
MT1M	11638.78	-4.83	0.55	1.49E-19	1.14E-15
CCK	37.51	-4.94	1.86	5.60E-04	8.73E-03
OPCML	104.32	-4.96	1.34	2.04E-05	7.78E-04
LGR5	711.63	-5.06	2.26	1.31E-03	1.59E-02
MYOC	5581.10	-5.21	1.33	1.09E-05	4.91E-04
PPP1R1A	720.16	-5.21	0.96	8.49E-09	1.63E-06
PCDH10	528.39	-5.33	0.76	3.83E-13	3.46E-10
CSF3	180.04	-5.39	1.23	1.41E-06	1.05E-04
KLHL40	16.13	-5.46	1.48	2.48E-05	9.06E-04
PROM1	35.67	-5.48	1.57	4.03E-05	1.30E-03
CYP4B1	1217.57	-5.59	1.09	4.13E-08	6.09E-06
TFF3	408.71	-5.69	1.57	3.22E-05	1.11E-03
MT1A	9262.22	-5.99	0.88	1.79E-12	1.31E-09
ALKAL2	761.60	-6.00	0.81	3.18E-14	3.76E-11
GPR182	29.10	-6.46	1.66	7.75E-06	3.81E-04
NMRK2	27.61	-6.71	1.67	7.36E-06	3.67E-04
CAV3	128.75	-6.89	1.55	1.11E-06	8.67E-05
SLC6A17	16.66	-7.16	1.80	4.32E-07	4.22E-05
SAA1	202.90	-7.77	1.41	6.51E-09	1.35E-06
