# Supplementary material for: Elucidating the fundamental fibrotic processes driving abdominal adhesion formation
Source: Nat Commun. 2020 Aug 13;11:4061. doi: 10.1038/s41467-020-17883-1 (PMC7426428; doi:10.1038/s41467-020-17883-1)
Supplement: Supplementary file 5 — Reporting Summary [file 41467_2020_17883_MOESM5_ESM.pdf]

nature research

Corresponding author(s): Michael Longaker, MD

Last updated by author(s): Jul 3, 2020

Reporting Summary

Nature Research wishes to improve the reproducibility of the work that we publish. This form provides structure for consistency and transparency in reporting. For further information on Nature Research policies, see Authors & Referees and the Editorial Policy Checklist .

Statistics

For all statistical analyses, confirm that the following items are present in the figure legend, table legend, main text, or Methods section.

n/a	Confirmed	
☐	☒	The exact sample size (*n*) for each experimental group/condition, given as a discrete number and unit of measurement
☐	☒	A statement on whether measurements were taken from distinct samples or whether the same sample was measured repeatedly
☐	☒	The statistical test(s) used AND whether they are one- or two-sided<br>*Only common tests should be described solely by name; describe more complex techniques in the Methods section.*
☐	☒	A description of all covariates tested
☐	☒	A description of any assumptions or corrections, such as tests of normality and adjustment for multiple comparisons
☐	☒	A full description of the statistical parameters including central tendency (e.g. means) or other basic estimates (e.g. regression coefficient) AND variation (e.g. standard deviation) or associated estimates of uncertainty (e.g. confidence intervals)
☐	☒	For null hypothesis testing, the test statistic (e.g. *F*, *t*, *r*) with confidence intervals, effect sizes, degrees of freedom and *P* value noted<br>*Give P values as exact values whenever suitable.*
☒	☐	For Bayesian analysis, information on the choice of priors and Markov chain Monte Carlo settings
☒	☐	For hierarchical and complex designs, identification of the appropriate level for tests and full reporting of outcomes
☐	☒	Estimates of effect sizes (e.g. Cohen's *d*, Pearson's *r*), indicating how they were calculated

Our web collection on statistics for biologists contains articles on many of the points above.

Software and code

Policy information about availability of computer code

Data collection	No code was used for data collection.
Data analysis	GraphPad Prism 6, ImageJ/FIJI v2.0.0, Imaris (Bitplane) 9.2, and FlowJo (TreeStar) 10 software packages were used to analyze the data in this research as indicated in the manuscript. For sequencing data analysis, Salmon v0.12.0 , DESeq2 v1.22.2, tximport v1.4.0, clusterProfiler v3.10.0, GSEA software provided by the Broad Institute, Cell Ranger (10X Genomics; v3.1), STAR v2.7.0, Seurat v3.1.1, SingleR v3.11, Monocle2 package in R v2.4.0, CytoTRACE package for R v0.3.2, and EnrichR v2.1 were used. These tools are referenced in the manuscript and are publicly available.

For manuscripts utilizing custom algorithms or software that are central to the research but not yet described in published literature, software must be made available to editors/reviewers. We strongly encourage code deposition in a community repository (e.g. GitHub). See the Nature Research guidelines for submitting code & software for further information.

Data

Policy information about availability of data

All manuscripts must include a data availability statement. This statement should provide the following information, where applicable:

- Accession codes, unique identifiers, or web links for publicly available datasets
- A list of figures that have associated raw data
- A description of any restrictions on data availability

Data to support the conclusions drawn in this manuscript can be found in the primary and supplemental figures. Source data are provided with this paper. All RNA-seq data discussed in this publication have been deposited in NCBI's Gene Expression Omnibus (GEO)51 and are accessible through GEO Series accession number GSE GSE153929 (https://www-ncbi-nlm-nih-gov /geo/query/acc.cgi?acc=GSE153929).

Field-specific reporting

Please select the one below that is the best fit for your research. If you are not sure, read the appropriate sections before making your selection.

- [x] Life sciences
- [ ] Behavioural & social sciences
- [ ] Ecological, evolutionary & environmental sciences

For a reference copy of the document with all sections, see nature.com/documents/nr-reporting-summary-flat.pdf

Life sciences study design

All studies must disclose on these points even when the disclosure is negative.

Sample size	A minimum sample size of 3 animals (range 3-10) was used for each time-point for each condition for all experiments completed throughout the duration of the study. Sample size was selected for each experiment based on pilot and published studies using the same model (PMID: 276855681). Animals were selected based on their genotype and randomly assigned to experimental groups. All sample sizes are reported in detail in the figure legends and/or Methods section.
Data exclusions	No data were excluded from our experimental analyses.
Replication	All data analyzed in the present study resulted from experiments that were performed with at least 3 biological replicates, and independent experiments were repeated at least 3 times. All attempts at replication were successful.
Randomization	Animals were randomly assigned to both experimental and control groups based on their genotype.
Blinding	All experiments with any potential for observer bias and whenever feasible (primarily quantitation of imaging data) were performed/analyzed in a blinded manner. Information was revealed post-analysis.

Reporting for specific materials, systems and methods

We require information from authors about some types of materials, experimental systems and methods used in many studies. Here, indicate whether each material, system or method listed is relevant to your study. If you are not sure if a list item applies to your research, read the appropriate section before selecting a response.

Materials & experimental systems

n/a	Involved in the study	
☐	☒	Antibodies
☐	☒	Eukaryotic cell lines
☒	☐	Palaeontology
☐	☒	Animals and other organisms
☐	☒	Human research participants
☒	☐	Clinical data

Methods

n/a	Involved in the study	
☒	☐	ChIP-seq
☐	☒	Flow cytometry
☒	☐	MRI-based neuroimaging

Antibodies

Antibodies used

Antibodies used for FACS included: Anti-phospho-JUN (Cell Signaling, s73d47G9, lot: 5, used at 1:200), anti-phospho-STAT5-PECy7 (BD Biosciences, 560117, lot: 8266820, pre-diluted and used at manufacturer's volume per test of 20ul), anti-FSP-1 (Ray biotech, 188-11191, lot: 1804128, used at 10ug/mL), IgG Pacific Blue (Thermo Fisher, p31582, lot: 1929717, used at 1 ug/mL), anti-PDGFR (Abcam, ab90967, lot: gr321324-2, used at 10ug/mL), IgG Alexa-Fluor 647 (Abcam, ab150159, lot: GR241187-2, used at 1:2000), IgG Alexa-Fluor 488 (Abcam, ab150077, lot: GR3224145-2, used at 1:2000), IgG Alexa Fluor 647 (Abcam, ab: 150075, lot: GR269275-2, used at 1:2000), IgG Pacific Blue (Thermo Fisher, P10994, lot: 2045342, used at 1ul/mL), anti-S100A4-PE (BioLegend, 370003; lot b286200, used at 1:20), anti-PDGFRa (Abcam, ab203491, lot: GR3226597-1, used at 1:50), anti-CD26 PECy7 (Biolegend, 302713, lot: B253866, used at 1:20), anti-CD45-FITC (Invitrogen, 11-9459-42, lot 4319940, used at 1:20), anti-Ter119-FITC (Invitrogen, 11-5921-85, lot :4322597, used at 2.5ug/mL), anti-CD31-FITC (Thermo Fisher, 11-0311-82, lot: B224877, used at 1:100), anti-Tie2 (Thermo Fisher, 14-5987-82, lot: 2072830, used at 1:100), anti-CD324 (Biolegend, 147302, lot: B228369, used at 10ug/mL), anti-CD326 (Biolegend, 118202, lot: B254013, used at 0.6ug/mL), 488 secondary (Abcam, ab150157, used at 1:2000), anti-CD45-eFluor 450 (Invitrogen, 48-0451-82, lot: 1936503, used at 5ug/mL), anti-Ter119-Pacific Blue (Invitrogen, 48-5921-82, lot: 1974934, used at 5 ug/mL), anti-CD31-eFluor 450 (Invitrogen, 48-0311-82, lot: 1982691, used at 2.5 ug/mL), anti-Tie2-biotin (Invitrogen, 13-5978-82, lot: 4304957, used at 5 ug/mL), anti-CD324-biotin (Invitrogen, 13-3249-82, lot:1916204, used at 2.5 ug/mL), eFluor 450-Streptavidin (Invitrogen, 48-4317-82, lot 1988686, used at 2.5 ug/mL), anti-CD326-eFluor 450 (Invitrogen, 48-5791-82, lot: 1984115, used at 10 ug/mL), anti-CD45-PECy7 (Thermo Fisher, MHCD4512, used at 1:100), anti-Ter119-PECy7 (Invitrogen, 25-5921-82, lot: 1994153, used at 5 ug/mL), anti-CD31-PECy7 (Invitrogen, 25-0311-81, lot 4318668, used at 5 ug/mL), anti-Tie2 (Invitrogen, 14-5987-82, lot: 2072830, used at 10 ug/mL), anti-CD326-PECy7, (BioLegend, 324221, lot: B266928, used at 1:20), and anti-CD324-PECy7 (Biolegend, 147310, lot: B255274, used at 2.4 ug/mL). Antibodies used for ICC and IF included: Anti-phospho-JUN (Cell Signaling, S63 (54B3), lot: 7, used at 1:100), anti-JUN (Abcam, ab31419, lot: GR306615-18, used at 1:50), anti-aSMA (Abcam, ab32575, lot: GR282976-32, used at 1:100), anti-FSP-1/S100A4 (Abcam,

ab41532 lot: GR3176834-1, used at 1:200), anti-COL3 (Abcam, ab7778, lot: GR3234897-1, used at 1:100), anti-COL1 (Abcam, ab34710, lot: GR3244041-2, used at 1:100), anti-MSLN (ABBiotec, 250519, lot: 15102712, used at 1:100), anti-CD26 (Abcam, ab222716, lot: GR3220836-1, used at 1:100), anti-vimentin (Abcam, ab11256, lot: GR236597-5, used at 1:20), anti-phospho-FAK (Thermo Fisher, 799255, lot: RG240925A, used at 1:100), anti-PDPN (Invitrogen, MA5-29742, lot: UB2724771, used at 1:250), anti-CD10 (Abcam, ab227640, lot: GR3227478-1, used at 1:100), anti-CD31 (Abcam, ab28364, lot: GR3247742-7, used at 1:50), anti-CD45 (Abcam, ab10558, lot: GR269008-1, used at 1:150), anti-phospho-Stat5 (Cell Signaling, 9314S, used at 1:200), anti-PDGFRa (Abcam, ab203491, lot: GR3226597-1, used at 1:200), IgG Alexa-Fluor 488 (Invitrogen, A32731, lot: SH251139, used at 1:1000), IgG Alexa Fluor 555 (Invitrogen, A32732, lot: SH251140, used at 1:1000), IgG Alexa Fluor 647 (Invitrogen, A32733, lot: SI231745, used at 1:1000).

All antibodies and concentrations used in experiments are also listed in the Methods section of the manuscript.

Validation

All antibodies used were validated by the suppliers they were acquired from and were used at the optimized conditions according to the manufacturer's recommendations. Additional validation information and relevant publications are available on the manufacturers' websites.

Eukaryotic cell lines

Policy information about cell lines

Cell line source(s)

Cell lines were acquired from the NIH and ATCC.

Authentication

All cell lines were acquired from the source, authentication provided with purchase.

Mycoplasma contamination

All cells lines were tested for Mycoplasma contamination and found to be negative by the vendor prior to shipping.

Commonly misidentified lines
(See ICLAC register)

Name any commonly misidentified cell lines used in the study and provide a rationale for their use.

Animals and other organisms

Policy information about studies involving animals; ARRIVE guidelines recommended for reporting animal research

Laboratory animals

All experiments were performed on eight to ten-week old mice in the following mouse strains: Black/6 (C57BL/6J), ROSA26mTmG (B6.129(Cg)-Gt(ROSA)26Sortm4(ACTB-tdTomato,-EGFP)Luo/J), PDGFRa-GFP (B6.129S4-Pdgfratm11EGFPSor/J), PDGFRa-CreERT2(B6N. Cg-Tg (Pdgfra-cre/ERT)467 Dbe/J), eGFP (C57BL/6J-Tg(CAG-EGFP)10sb/J), Actin-CreERT2 (Tg(CAG-cre/Esr1)5Amc/J), En1Cre (En1- tm2(cre)Wrst/J), Wt1CreERT2 (Wt1-tm2(cre/ERT2)Wtp/J), Rainbow mice (ROSA26VT2/GK3), and flp-in tetO-c-JUN. Mice were housed at the Stanford University Comparative Medicine Pavilion (CMP) and Research Animal Facility (RAF). The facilities provided light- & temperature-regulated housing for all animals. Mice were given rodent chow and water ad libitum.

Wild animals

Wild animals were not used in this study.

Field-collected samples

Field-collected samples were not used in this study.

Ethics oversight

All mouse experiments were conducted under the guidance and approval of Stanford University's IACUC/APLAC. All human abdominal adhesion and control specimens were obtained at the Stanford Hospital under Stanford University's IRB approval.

Note that full information on the approval of the study protocol must also be provided in the manuscript.

Human research participants

Policy information about studies involving human research participants

Population characteristics

In total, 34 patients were recruited to participate in this study (Supplementary Table 2). From this cohort, adhesion specimens were obtained from 24 patients and control peritoneal tissue was obtained from 10 patients.

Recruitment

Human abdominal tissue specimens were obtained from patient's undergoing abdominal surgery at the Stanford Hospital under Stanford University's IRB approval. Tissue specimens included only tissues that would otherwise have been discarded. Inclusion criteria for patients were as follows: For all patients, patients must be over the age of 18, surgery must be elective, and there must be no evidence of active inflammation or infection at time of operation. For adhesion specimens – patients must have had at least one prior abdominal surgery, for control specimens - patients must have had no history of prior abdominal surgery. The patients were approached in the pre-operative area by one of the manuscript authors. The aims of the study were discussed with the patient. Participation was entirely voluntary. Written, informed consent was obtained from the patient prior to surgery. Tissue specimens were collected by one of the authors on this manuscript from the primary surgeon in the operating room, placed directly into sterile saline, and kept on ice for transport. Tissue specimens were processed immediately. As all patients approached to participate in this research agreed to participate. As such, we do not suspect a significant role for self-selection bias. In addition, cases were selected at random for recruitment based on the research progress and minimizing any other potential selection biases.

Ethics oversight

Human abdominal adhesion and control specimens were obtained under Stanford University's IRB approval.

Note that full information on the approval of the study protocol must also be provided in the manuscript.

Flow Cytometry

Plots

Confirm that:

- [x] The axis labels state the marker and fluorochrome used (e.g. CD4-FITC).
- [x] The axis scales are clearly visible. Include numbers along axes only for bottom left plot of group (a 'group' is an analysis of identical markers).
- [x] All plots are contour plots with outliers or pseudocolor plots.
- [x] A numerical value for number of cells or percentage (with statistics) is provided.

Methodology

Sample preparation	Adhesion or control tissue from mouse or human specimens was minced on ice. The tissue was then digested for 60 minutes in a 37C water bath agitator in 2 mg/mL collagenase (collagenase type IV, ThermoFisher) digest buffer in Medium 199 (HyClone, GE Healthcare) consisting of 5% fetal bovine serum (Gibco FBS, ThermoFisher), DNase I (Worthington), Poloxamer 188 (Cat. P5556-100ML, Sigma), HEPES, and CaCl2. The digest was quenched with quench media (DMEM (Gibco DMEM, ThermoFisher) with 15% FBS), then centrifuged at 300 x G for 5 minutes at 4C, resuspended in quench media, and filtered through 100, 70, and 40m cell strainers (Falcon cell strainer, ThermoFisher). Red blood cell lysis was performed using Hybri-Max (Sigma) per the manufacturers protocol. Histopaque was performed using Histopaque-1119 (Sigma Aldrich), per the manufacturers protocol. Cells were counted and re-suspended in FACS buffer. Primary antibodies were applied, and cells were stained in the dark with gentle agitation for 30 minutes. Cells were then washed thoroughly in FACS buffer. Staining with secondary antibodies was conducted in the same manner.
Instrument	BD FACS Aria II system
Software	Data was imported from BD FACS Aria II into FlowJo and was analyzed as described in the "Materials & Methods" section.
Cell population abundance	Cell populations were isolated based on purity. Both morphological characteristics and functional capabilities were assessed depending on the experiment.
Gating strategy	Single cells were gated primarily using FSC and SSC parameters. Unstained cells were assessed for each experiment. Propidium iodine (PI, Thermofisher, Cat. P3566, lot: 1755970, 3 µg/mL) or DAPI (Thermofisher, Cat. 3571) were used as a viability marker. A panel of lineage-markers were used to isolate (and discard) non-fibroblast cells. Adhesion fibroblasts were further characterized by their immunophenotype and gating was performed to assess this depending on the experiment.

- [x] Tick this box to confirm that a figure exemplifying the gating strategy is provided in the Supplementary Information.
